# Candidate regulators of Early Leaf Development in Maize Perturb Hormone Signalling and Secondary Cell Wall Formation When Constitutively Expressed in Rice

**DOI:** 10.1038/s41598-017-04361-w

**Published:** 2017-07-03

**Authors:** Peng Wang, Shanta Karki, Akshaya K. Biswal, Hsiang-Chun Lin, Mary Jacqueline Dionora, Govinda Rizal, Xiaojia Yin, Mara L. Schuler, Tom Hughes, Jim P. Fouracre, Basel Abu Jamous, Olga Sedelnikova, Shuen-Fang Lo, Anindya Bandyopadhyay, Su-May Yu, Steven Kelly, W. Paul Quick, Jane A. Langdale

**Affiliations:** 1 Department of Plant Sciences, University of Oxford, South Parks Rd, Oxford, OX1 3RB UK; 2 International Rice Research Institute, Los Banos, 4030 Laguna, Philippines; 3 Institute of Molecular Biology, Academia Sinica, Taipei, 11529 Taiwan; 4 Baniyatar-220, Tokha-12, Kathmandu, Nepal; 5 Department of Biology, University North Carolina, Chapel Hill, NC 27599 USA; 6 Department of Biology, Heinrich Heine University, D-40225 Düsseldorf, Germany; 7 Department of Biology, University of Pennsylvania, Philadelphia, PA 19104 USA; 8 Ministry of Agricultural Development, Government of Nepal, Singhadurbar, Kathmandu Nepal

## Abstract

All grass leaves are strap-shaped with a series of parallel veins running from base to tip, but the distance between each pair of veins, and the cell-types that develop between them, differs depending on whether the plant performs C_3_ or C_4_ photosynthesis. As part of a multinational effort to introduce C_4_ traits into rice to boost crop yield, candidate regulators of C_4_ leaf anatomy were previously identified through an analysis of maize leaf transcriptomes. Here we tested the potential of 60 of those candidate genes to alter leaf anatomy in rice. In each case, transgenic rice lines were generated in which the maize gene was constitutively expressed. Lines grouped into three phenotypic classes: (1) indistinguishable from wild-type; (2) aberrant shoot and/or root growth indicating possible perturbations to hormone homeostasis; and (3) altered secondary cell wall formation. One of the genes in class 3 defines a novel monocot-specific family. None of the genes were individually sufficient to induce C_4_-like vein patterning or cell-type differentiation in rice. A better understanding of gene function in C_4_ plants is now needed to inform more sophisticated engineering attempts to alter leaf anatomy in C_3_ plants.

## Introduction

Genes that regulate developmental processes have traditionally been identified through mutant screens for phenotypic defects. This approach has been enormously powerful for the elucidation of genetic pathways that underpin a large number of developmental mechanisms in model organisms across the phylogenetic range. However, where redundancy or complex genetic interactions operate, such mutant screens have rarely been successful. The development of a characteristic leaf anatomy known as ‘Kranz’ is a case in point. Despite concerted efforts to identify mutations that disrupt Kranz patterning, only two mutants have been identified^1, 2^ and both of these cause very subtle defects.

Kranz anatomy is a feature of plants that carry out C_4_ photosynthesis (reviewed in ref. 3). Whereas C_3_ plants carry out photosynthesis in a single photosynthetic cell-type, most C_4_ plants compartmentalize photosynthetic reactions between two distinct cell-types known as mesophyll (M) and bundle sheath (BS). For the C_4_ metabolic pathway to be effective, M cells need to be in contact with BS cells. As such, whereas C_3_ leaves develop 5–20 M cells between each pair of veins, C_4_ leaves develop concentric wreaths of BS and M cells around each vein (V) so that each pair of veins is normally separated by four cells in a V-BS-M-M-BS-V unit. This anatomy led to the name Kranz, which is German for wreath^4^. Remarkably, Kranz anatomy has evolved on over 60 independent occasions^5^, but there is currently very little indication of the genetic mechanisms that were recruited to enable the transition from the ancestral C_3_-type leaf anatomy.

Over the last ten years, bioinformatics approaches have increasingly been used to interrogate genome and transcriptome datasets with a view to identifying novel regulators of plant development. In the context of understanding C_4_ physiology and evolution, many comparisons have been made between different developmental stages within C_4_ plants^6–12^, and between closely related C_3_ and C_4_ species^13–18^. However, the most focussed study in terms of understanding how Kranz anatomy develops exploited the fact that the C_4_ plant maize develops two type of leaves – those with Kranz anatomy (foliar leaves) and those without Kranz anatomy (husk leaves surrounding the female inflorescence)^19^. The most striking anatomical difference between the two leaf types is that pairs of veins are separated by four cells in foliar leaves and by ~15–20 cells in husk leaves^20^. Genome-wide comparisons of transcriptomes from developing foliar and husk leaf primordia of maize identified 283 genes as potential positive regulators of Kranz anatomy^19, 21^.

The power of bioinformatics to generate lists of candidate developmental regulators has been proven. However, gene function can only be validated experimentally. In some model organisms this is straightforward in that loss- and gain- of function manipulations can be carried out both precisely and rapidly (e.g. ref. 22). However, even in the era of genome editing, testing necessity of gene function in the current C_4_ model systems is not high throughput enough to tackle large gene lists. In the face of this challenge, candidate positive regulators of Kranz anatomy were tested for sufficiency to perturb leaf anatomy in the C_3_ plant rice. 60 maize genes, most of which encoded transcription factors, were individually transformed into rice under the control of the maize ubiquitin promoter. Phenotypic characterization of vein spacing in leaves of transgenic plants (as a primary indicator of a shift towards Kranz-like anatomy) revealed that none of the candidate genes specifically altered leaf anatomy when expressed constitutively. Indeed, constitutive expression of ~75% of the tested genes had no apparent phenotypic effect on rice growth. The remaining 13 genes conditioned phnetypes that may represent roles in hormone signalling and/or secondary wall formation, providing novel insights into gene function.

## Results and Discussion

### Constitutive expression of maize genes in rice

To determine whether genes that had been identified in maize as candidate regulators of Kranz anatomy could induce Kranz-like features in the leaves of rice, a systematic transgenic approach was adopted. Of the 283 genes that had previously been identified as putative regulators of Kranz patterning^19^, 60 genes were chosen for analysis. The main criterion for selection was a predicted regulatory role, and hence the majority of genes encoded transcription factors (36 genes) with another group encoding leucine rich repeat receptor like kinases (LRR-RLK) (14 genes). Sequences for each of the 60 genes were either amplified by reverse transcriptase polymerase chain reaction (RT-PCR), using RNA isolated from maize shoots comprised of the shoot apical meristem plus plastochron (P) 1–5 leaf primordia (a plastochron is the time interval between initiation of primordia at the shoot apex, with P1 primordia being the youngest and closest to the apex), or by PCR from maize genomic DNA. Amplified sequences (Supplementary Information S1) were ligated downstream of the maize ubiquitin promoter in transformation vectors and then constructs were transformed into either indica (IR64) or japonica (Kitaake) rice varieties. Positive transformants were validated for all 60 genes by genomic PCR or DNA blot analysis. Phenotypic analyses were carried out either on multiple independent T0 lines (29 genes) and/or on multiple individuals from 2–3 independent T1 lines (31 genes). The list of genes analyzed is summarized in Table 1.

**Table 1:** Summary of genes analyzed.

Lab ID	Gene ID	Gene Family	Rice variety	Lines examined	Phenotype in overexpression line
JL2	GRMZM2G028046	MYB-interacting (***ZmMIL1***)	Kitaake	T1 (2)	Normal (Supplementary Dataset S3 and Information S3)
JL3	GRMZM2G136494	MYB-interacting (***ZmMIL2***)	Kitaake	T1 (3)	Normal (Supplementary Dataset S3 and Information S3)
JL4	GRMZM2G045883	bHLH (***ZmSPL1***)	Kitaake	T1 (2)	Normal (Supplementary Dataset S3 and Information S3)
JL8	GRMZM2G131516	GRAS (***ZmSCR1***)	Kitaake	T1 3)	Normal (Supplementary Dataset S3 and Information S3)
JL13	AC215201.3_FG008	bHLH	Kitaake	T1 (2)	Normal (Supplementary Dataset S3 and Information S3)
JL15	GRMZM2G016477	LRR kinase	Kitaake	T2 (3)	Normal (Supplementary Dataset S3 and Information S3)
JL22	GRMZM2G480386	YUCCA	Kitaake	T0 (>20)	Defective regeneration (Fig. 1)
JL23	GRMZM2G069365	ZnF HD (***ZmHBa***)	Kitaake	T0 (2)	Spindly (Fig. 6)
JL24	GRMZM2G417229	ZnF HD (***ZmHBb***)	Kitaake	T0 (6)	Spindly (Fig. 6)
JL25	GRMZM2G425236	ZnF HD (***ZmHBc***)	Kitaake	T0 (6)	Spindly (Fig. 6)
JL26	GRMZM2G119359	Growth regulating factor	Kitaake	T1 (2)	Normal (Supplementary Dataset S3 and Information S3)
JL27	GRMZM5G893117	Growth regulating factor	Kitaake	T1 (4)	Normal (Supplementary Dataset S3 and Information S3)
JL28	GRMZM2G114893	Unknown	Kitaake	T0 (8)	Stunted growth (Fig. 4)
JL29	GRMZM2G178182	bHLH	Kitaake	T1 (2)	Normal (Supplementary Dataset S3 and Information S3)
JL30	GRMZM2G140669	GATA ZnF	Kitaake	T1 (2)	Normal (Supplementary Dataset S3 and Information S3)
JL31	GRMZM2G472945	TUBBY-like	Kitaake	T1 (2)	Normal (Supplementary Dataset S3 and Information S3)
JL32	GRMZM2G146688	AP2	Kitaake	T1 (4)	Normal (Supplementary Dataset S3 and Information S3)
JL33	GRMZM2G111045	R2R3 MYB	Kitaake	T1 (2)	Drooping leaf (Fig. 3)
JL34	GRMZM2G377217	WRKY (***ZmWRKY12***)	Kitaake	T0 (>20) T1 (2)	No regeneration/inducible mini-plant (Fig. 5)
JL35	GRMZM2G023051	Unknown	Kitaake	T1 (2)	Normal (Supplementary Dataset S3 and Information S3)
JL36	GRMZM2G109480	Unknown	Kitaake	T1 (2)	Normal (Supplementary Dataset S3 and Information S3)
JL38	GRMZM2G074032	Znf C2H2 (***ZmIDD16***)	Kitaake	T0 (>20)	No regeneration
JL39	GRMZM2G027068	bHLH (***ZmbHLH106***)	Kitaake	T0 (>20)	No regeneration
JL40	GRMZM2G140694	Znf C2H2 (***ZmHCA2***)	Kitaake	T0 (>20)	No regeneration
JL43	GRMZM2G098988	bHLH (***ZmSACL3***)	IR64	T0 (>20)	Defective regeneration (Fig. 1)
JL44	GRMZM2G123900	Znf DOF	IR64	T0 (19)	Normal (Supplementary Dataset S2 and Information S2)
JL45	GRMZM2G374986	MYB	IR64	T0 (35)	Normal (Supplementary Dataset S2 and Information S2)
JL46	GRMZM2G011463	Auxin SAUR (***ZmSAUR60***)	IR64	T0 (35)	Defective regeneration (Fig. 1)
JL47	GRMZM2G097275	SBP	Kitaake	T1 (2)	Normal (Supplementary Dataset S3 and Information S3)
JL48	GRMZM2G148467	SBP	Kitaake	T1 (2)	Normal (Supplementary Dataset S3 and Information S3)
JL49	GRMZM2G028643	LRR kinase	Kitaake	T1 (2)	Normal (Supplementary Dataset S3 and Information S3)
JL50	GRMZM2G163724	LRR kinase	Kitaake	T1 (2)	Slow growth and dwarfed (Fig. 2)
JL51	GRMZM2G178102	Class III HD-ZIP	Kitaake	T1 (2)	Normal (Supplementary Dataset S3 and Information S3)
JL53	GRMZM2G163975	AP2	IR64	T0 (20)	Normal (Supplementary Dataset S2 and Information S2)
JL54	GRMZM2G077219	Unknown	IR64	T0 (17)	Normal (Supplementary Dataset S2 and Information S2)
JL55	GRMZM2G082586	bHLH	IR64	T0 (10)	Normal (Supplementary Dataset S2 and Information S2)
JL56	GRMZM2G171365	MADS box	IR64	T0 (34)	Normal (Supplementary Dataset S2 and Information S2)
JL57	GRMZM2G098813	LFY	IR64	T0 (34)	Normal (Supplementary Dataset S2 and Information S2)
JL59	GRMZM2G139324	Unknown	IR64	T0 (35)	Normal (Supplementary Dataset S2 and Information S2)
JL60	GRMZM2G061314	LRR binding protein	IR64	T0 (28) T1 (2)	Normal (Supplementary Dataset S2 and S3, Information S2 and S2)
JL61	GRMZM2G151955	LRR-RLK	IR64	T0 (34)	Normal (Supplementary Dataset S2 and Information S2)
JL62	GRMZM2G159953	Lectin family RK	IR64	T0 (10)	Normal (Supplementary Dataset S2 and Information S2)
JL63	GRMZM2G039934	LRR-RLK (TDR/PXY)	IR64	T0 (35)	Normal (Supplementary Dataset S2 and Information S2)
JL64	GRMZM2G046316	LRR-RLK	IR64	T0 (35)	Normal (Supplementary Dataset S2 and Information S2)
JL65	GRMZM2G034155	LRR-RLK	IR64	T0 (35)	Normal (Supplementary Dataset S2 and Information S2)
JL66	GRMZM2G114276	LRR-RLK	IR64	T0 (35)	Normal (Supplementary Dataset S2 and Information S2)
JL67	GRMZM2G059117	LRR-RLK	IR64	T0 (34)	Normal (Supplementary Dataset S2 and Information S2)
JL68	GRMZM2G089819	LRR-RLK (Brassinosteroid)	IR64	T0 (35) T1 (2)	Normal (Supplementary Dataset S2 and S3, Information S2 and S2)
JL69	GRMZM2G344857	PIP kinase	IR64	T0 (35)	Normal (Supplementary Dataset S2 and Information S2)
JL70	GRMZM2G087243	Armadillo-like	IR64	T0 (34)	Normal (Supplementary Dataset S2 and Information S2)
JL71	GRMZM2G133716	Forkhead domain	IR64	T0 (29)	Normal (Supplementary Dataset S2 and Information S2)
JL72	GRMZM2G469304	Ternary complex factor MIP1	IR64	T0 (35)	Normal (Supplementary Dataset S2 and Information S2)
JL75	GRMZM5G850129	Growth regulating factor	IR64	T0 (5) T1 (2)	Normal (Supplementary Dataset S2 and S3, Information S2 and S2)
JL76	GRMZM2G061734	SBP	IR64	T0 (13) T1 (2)	Normal (Supplementary Dataset S2 and S3, Information S2 and S2)
JL77	GRMZM2G318592	ZnF C2H2	IR64	T0 (35)	Normal (Supplementary Dataset S2 and Information S2)
JL78	GRMZM2G095899	bHLH	IR64	T0 (10)	Normal (Supplementary Dataset S2 and Information S2)
JL79	GRMZM2G015666	bHLH	IR64	T0 (35)	Normal (Supplementary Dataset S2 and Information S2)
JL80	GRMZM2G126018	SBP	IR64	T0 (27) T1 (2)	Normal (Supplementary Dataset S2 and S3, Information S2 and S2)
JL81	GRMZM2G312419	R2R3 MYB	IR64	T0 (17) T1 (2)	Normal (Supplementary Dataset S2 and S3, Information S2 and S2)
JL82	GRMZM2G478876	Serine Threonine kinase	IR64	T0 (31) T1 (10)	Normal (Supplementary Dataset S2 and S3, Information S2 and S2)

### Constitutive expression of 47 candidate regulators of Kranz anatomy in maize caused no apparent phenotypic defects in rice

To establish a baseline against which perturbations to leaf anatomy could be quantitatively assessed, variation in vein number versus leaf width was first quantified in both IR64 and Kitaake rice varieties. In each case, a regression analysis was carried out using measurements of wild-type leaves at a number of different developmental stages, when grown in different environmental conditions, and in both T0 and T1 null segregants from transformation experiments (Supplementary Dataset S1). In both IR64 and Kitaake varieties, a linear relationship was revealed between leaf width and vein number, a relationship that was conserved regardless of developmental age or environmental growth conditions. Essentially, wider leaves have more veins across the mediolateral leaf axis and narrow leaves have fewer, with the distance between veins remaining roughly equivalent.

T0 lines transformed with 29 of the candidate genes (including 14 encoding transcription factors and 11 encoding receptor kinases) exhibited normal vein spacing patterns, as judged by regression analysis of leaf width versus vein number measurements (Table 1 and Supplementary Dataset S2 and Information S2). T1 lines transformed with a further 18 genes (including 13 encoding transcription factors) similarly exhibited normal vein spacing patterns (Table 1 and Supplementary Dataset S3 and Information S3). Qualitatively, these T0 and T1 lines were phenotypically normal throughout the lifecycle.

### *ZmIDD16*, *ZmbHLH106* and *ZmHCA2* may influence auxin signalling

Rice transformation protocols rely on the regeneration of plantlets from callus, using an excess of cytokinin to auxin in regeneration media to promote shoot growth. Constitutive expression of three genes prevented plantlets from regenerating under these conditions. Phylogenetic analysis revealed that these genes were: (1) *ZmINDETERMINATE DOMAIN (IDD16)* (an ortholog of *IDD14/15/16* in Arabidopsis^23^ and *LOOSE PLANT ARCHITECTURE1* (*LPA1*) in rice^24^) (Supplementary Figure S1); (2) an ortholog of *bHLH106* in Arabidopsis^25^ (Supplementary Figure S2); and (3) an ortholog of Arabidopsis *HIGH CAMBIAL ACTIVITY2* (*HCA2*)^26^ (Supplementary Figure S3). The maize genes are thus *ZmIDD16*, *ZmbHLH106* and *ZmHCA2* (Table 1). Notably, the Arabidopsis orthologs of *ZmIDD16* induce the expression of auxin biosynthesis and transport genes^23^, and although primarily characterized for the ability to confer salt tolerance, *AtbHLH106* also activates transcription of the auxin biosynthesis gene *AtYUCCA5* ^25, 27^. *AtHCA2* induces the formation of interfascicular cambium in Arabidopsis, a process known to be promoted by auxin^28^, with gain of function mutations leading to ectopic formation of vascular bundles in both stems and leaves^26^. Together these observations suggest that *ZmIDD16*, *ZmbHLH106* and *ZmHCA2* may promote auxin biosynthesis and/or signalling, such that constitutive gene expression in rice callus prevents plantlet regeneration by effectively reducing the cytokinin to auxin ratio. However, other explanations are possible and more direct evidence is needed to confirm the function of each gene.

### Constitutive expression of *ZmSAUR60*, *ZmSACL3* or a maize *YUCCA* gene perturbed shoot and/or root development in rice

**Figure 1:**
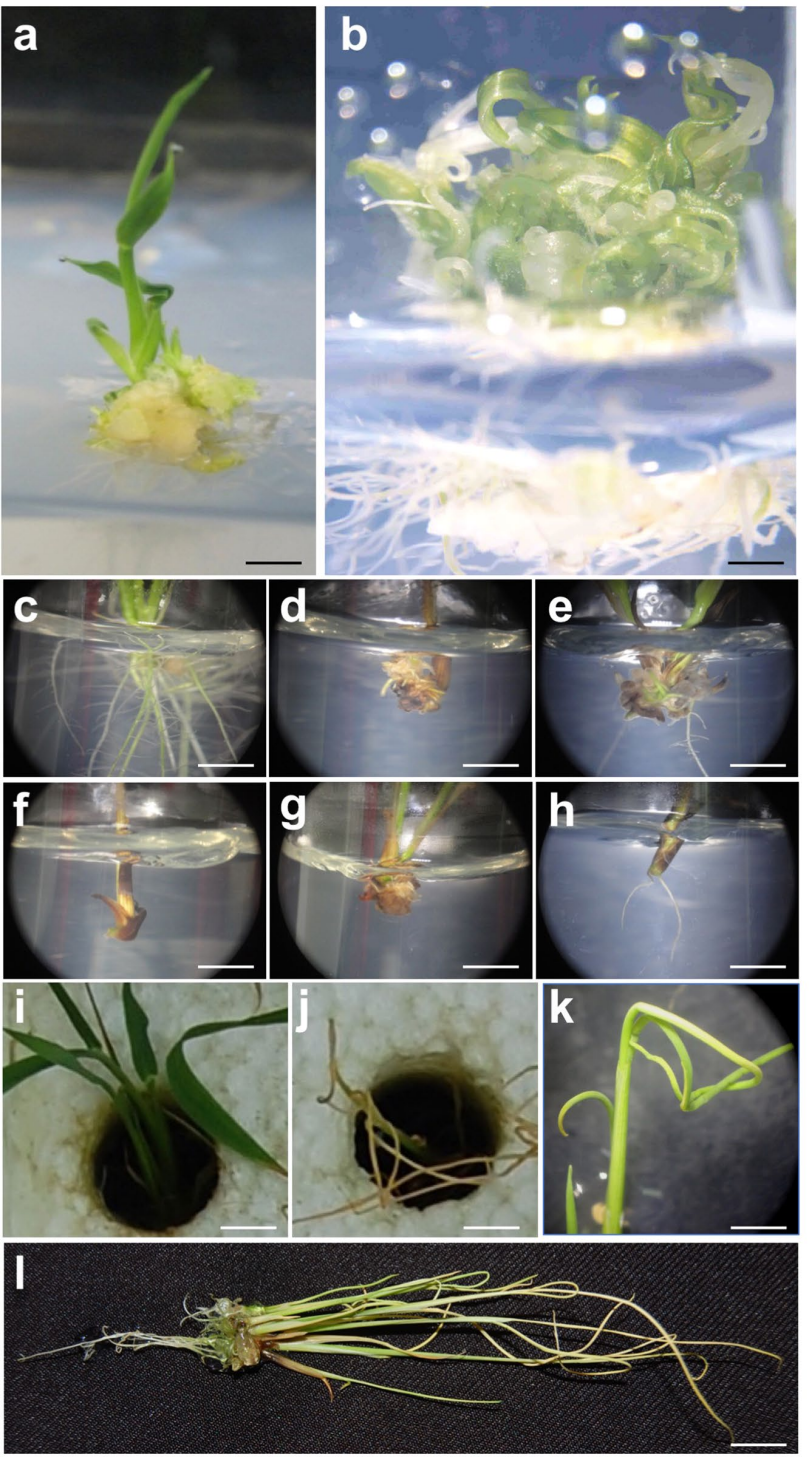
Phenotype of transgenic lines that fail to regenerate viable plants. (**a**,**b**) T0 plants of control (**a**) and transgenic (**b**) lines transformed with a maize *YUCCA* gene. (**c**–**h**) T0 plants of control (**c**) and transgenic (**d**–**h**) lines transformed with *ZmSAUR60*. (**i**–**l**) T0 plants of control (**i**) and transgenic (**j**–**l**) lines transformed with *ZmSACL3*. Scale bars = 0.5 cm (**a**,**c**–**h**); 0.2 cm (**b**); 1 cm (**i**,**j**,**l**); 0.8 cm (**k**).

Once transgenic plantlets regenerate from callus, endogenous developmental processes regulate cytokinin to auxin ratios in order to promote shoot and root growth, with cytokinin primarily promoting growth in the shoot meristem and auxin promoting growth in the root. Perturbed shoot and root phenotypes in multiple independent lines constitutively expressing each of three transgenes suggested that gene expression may interfere with cytokinin/auxin homeostasis (Fig. 1). Phylogenetic analysis demonstrated that one of the genes encodes a flavin monoxygenase-like enzyme of the YUCCA family (Supplementary Figure S4). *YUCCA* genes act in the auxin biosynthesis pathway and the phenotype of rice lines overexpressing the maize *YUCCA* gene (Fig. 1a,b) is consistent with excessive auxin levels. As previously seen in lines overexpressing rice *YUCCA* genes^29^, the maize gene caused a proliferation of curled leaflets and hairy roots (Fig. 1b). None of over 20 T0 plantlets survived transplantation to soil. The second gene had the opposite effect on root growth in that very few, if any, lateral roots were formed (Fig. 1c–h). Phylogenetic analysis revealed that the gene encodes a SMALL AUXIN UPREGULATED RNA (SAUR) protein (Supplementary Figure S5), previously annotated as *ZmSAUR60* ^30^. Although not the most closely related rice gene to *ZmSAUR60*, overexpression of *OsSAUR39* has been shown to negatively regulate auxin synthesis and transport in rice^31^. The phenotype of transgenic lines shown in Fig. 1f–j suggests that ZmSAUR60 may have a similar role, preventing auxin flow into the root and thus inhibiting primary root elongation and lateral root initiation. The third gene that severely perturbed growth in young plantlets was a bHLH transcription factor that is orthologous to *SACL3* in Arabidopsis (Supplementary Figure S6). This gene has thus been named *ZmSACL3* (Table 1). In Arabidopsis, SACL3 binds to the bHLH protein LONESOME HIGHWAY (LHW), preventing LHW from interacting with another bHLH protein, TARGET OF MONOPTEROS5 (TMO5)^32^. Given that LHW/TMO dimers activate cytokinin biosynthesis^33, 34^, competitive inhibition through an excess of SACL3 would lead to reduced levels of cytokinin, and an equivalent phenotype to that seen with increased auxin levels. Although the plantlets overexpressing *ZmSACL3* (Fig. 1i–l) reach a more advanced stage than those overexpressing the *YUCCA* gene (Fig. 1B), in both cases, the severely curled leaves might be explained by a decreased cytokinin to auxin ratio.

**Figure 2:**
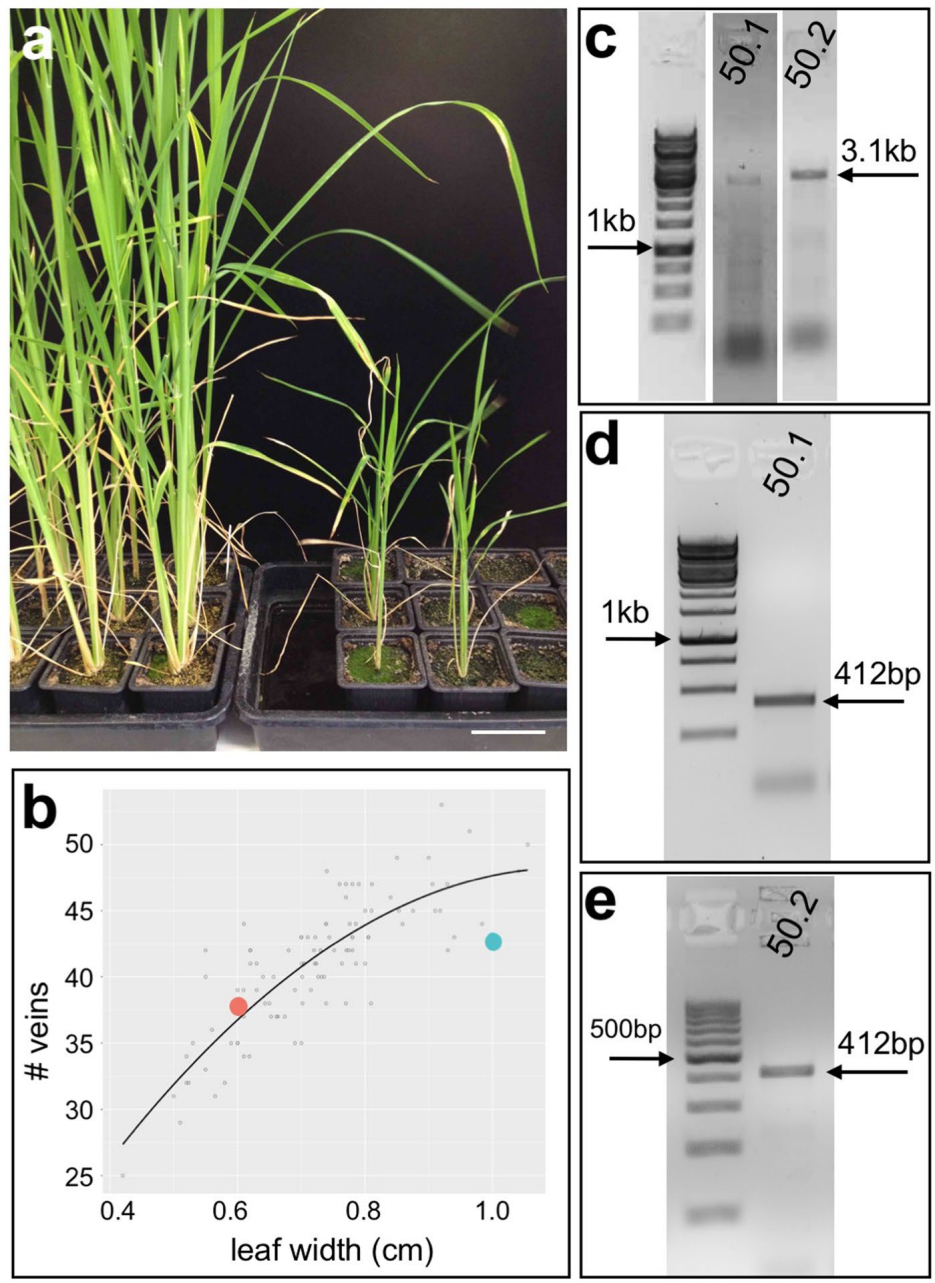
Transgenic lines that overexpress an LRR kinase are dwarfed. (**a**) Non-transgenic plants (left hand panel) are taller than transgenic plants (right hand panel). (**b**) Regression plot showing vein number versus leaf width for one individual from each of two independent T1 lines – 50.1 (orange spot) & 50.2 (blue spot). (**c**–**e**) Cropped gel images of genomic (**c**) and RT- (**d**,**e**) PCR reactions illustrate transgene presence and transcript levels in an individual from each T1 line. Scale bar = 5 cm.

Perturbations to auxin homeostasis may also underpin the dwarfed phenotype seen in lines expressing an LRR kinase (Table 1 and Fig. 2), but other possibilities cannot be excluded. Transgenic lines grew more slowly than non-transgenic controls but were fertile, exhibited normal vein spacing patterns, and were propagated to the T1 generation. Phylogenetic analysis revealed homology of the transgene to a clade of five genes from Arabidopsis (Supplementary Figure S7), loss of function mutations in which cause resistance to an auxin transport inhibitor (At2G23300 & At5G67280)^35^ or to abscisic acid (*AtRDK1*)^36^.

### A failure to differentiate abaxial sclerenchyma is associated with drooping leaves in rice lines expressing a maize R2R3 MYB gene

**Figure 3:**
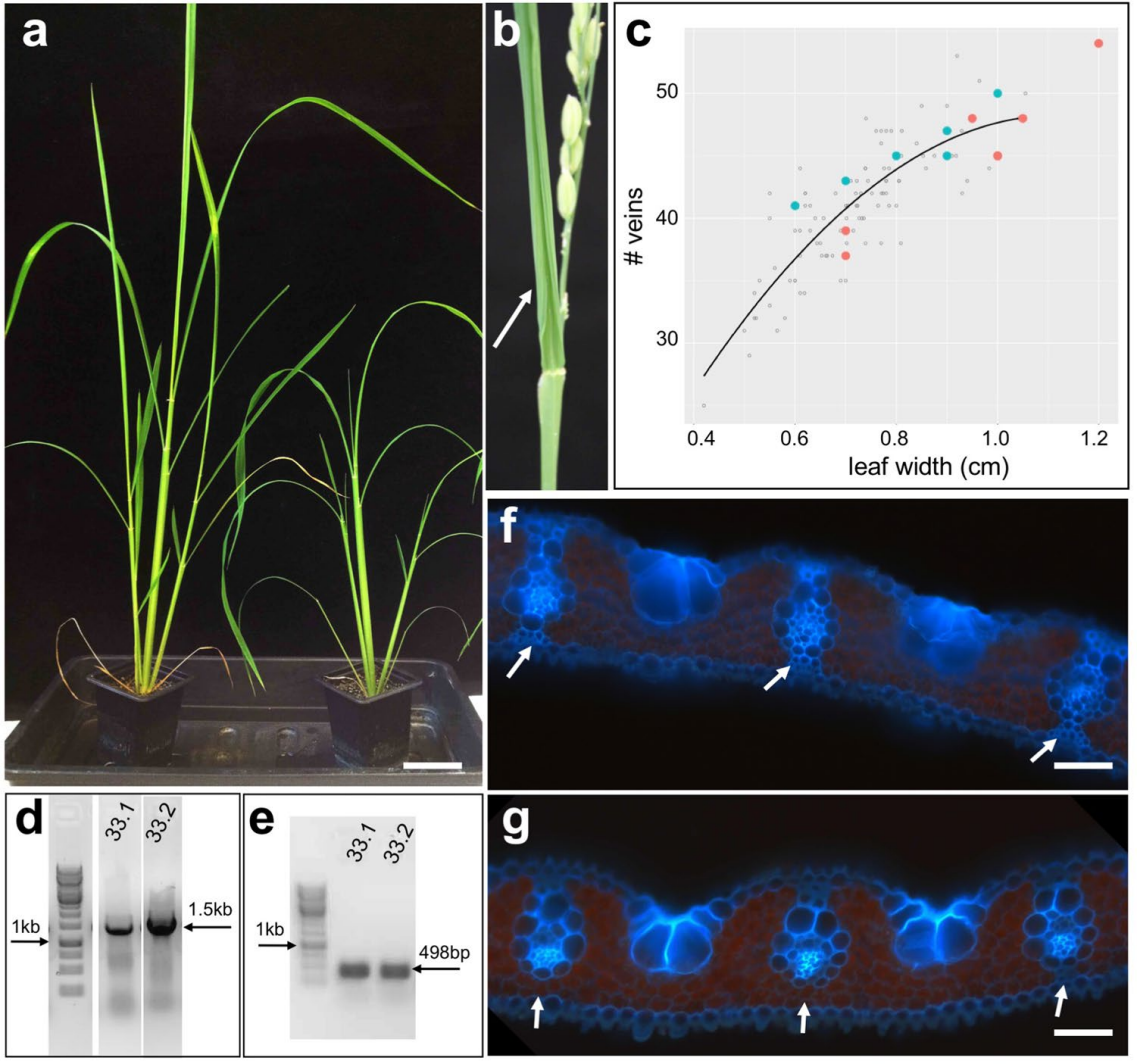
Transgenic lines overexpressing a maize R2R3 MYB gene exhibit drooping leaves and have fewer abaxial sclerenchyma cells than wild-type. (**a**) T1 transgenic plants (right hand panel) are shorter than non-transgenic plants (left hand panel), and exhibit a drooping leaf phenotype. (**b**) T0 plant showing abaxial curling of the flag leaf (white arrow). (**c**) Regression plot showing vein number versus leaf width for 6 individuals from each of two independent T1 lines − 33.1 (orange spots) & 33.2 (blue spots). (**d**,**e**) Cropped gel images of genomic (**d**) and RT- (**e**) PCR reactions illustrate transgene presence and transcript levels in representatives of each T1 line. (**f**,**g**) Representative transverse leaf sections from wild-type (**f**) and transgenic line 33.1 (**g**) plants, showing presence - white arrows in (**f**), and absence - white arrows in (**g**), of abaxial sclerenchyma. Scale bar = 5 cm (a); 50 µm (**f**,**g**).

Transgenic lines overexpressing a gene encoding an R2R3 MYB transcription factor exhibited drooping, abaxially curled leaves (Table 1 and Fig. 3). Vein spacing and number were unperturbed in the leaves of transgenic plants (Fig. 3c) but transverse leaf sections revealed that intermediate veins often lacked sclerenchyma cell connections to the abaxial epidermis (Fig. 3g). Given the structural role of sclerenchyma, this defect would be sufficient to cause abaxial leaf curling. Phylogenetic analysis revealed that the maize gene is orthologous to *LATE MERISTEM IDENTITY 2* (*LMI2*), a gene that regulates the vegetative to inflorescence transition in Arabidopsis^37^ (Supplementary Figure S8). No flowering time defects were observed in the transgenic rice lines, however, LMI2 is itself unusual, being nested in a clade that contains many positive and negative regulators of epidermal cell differentiation^38^. In this context, it is possible that the maize R2R3 MYB functions as a negative regulator of sclerenchyma development.

### Enhanced sclerenchyma formation around leaf veins infers a role for a novel gene in secondary cell wall formation

**Figure 4:**
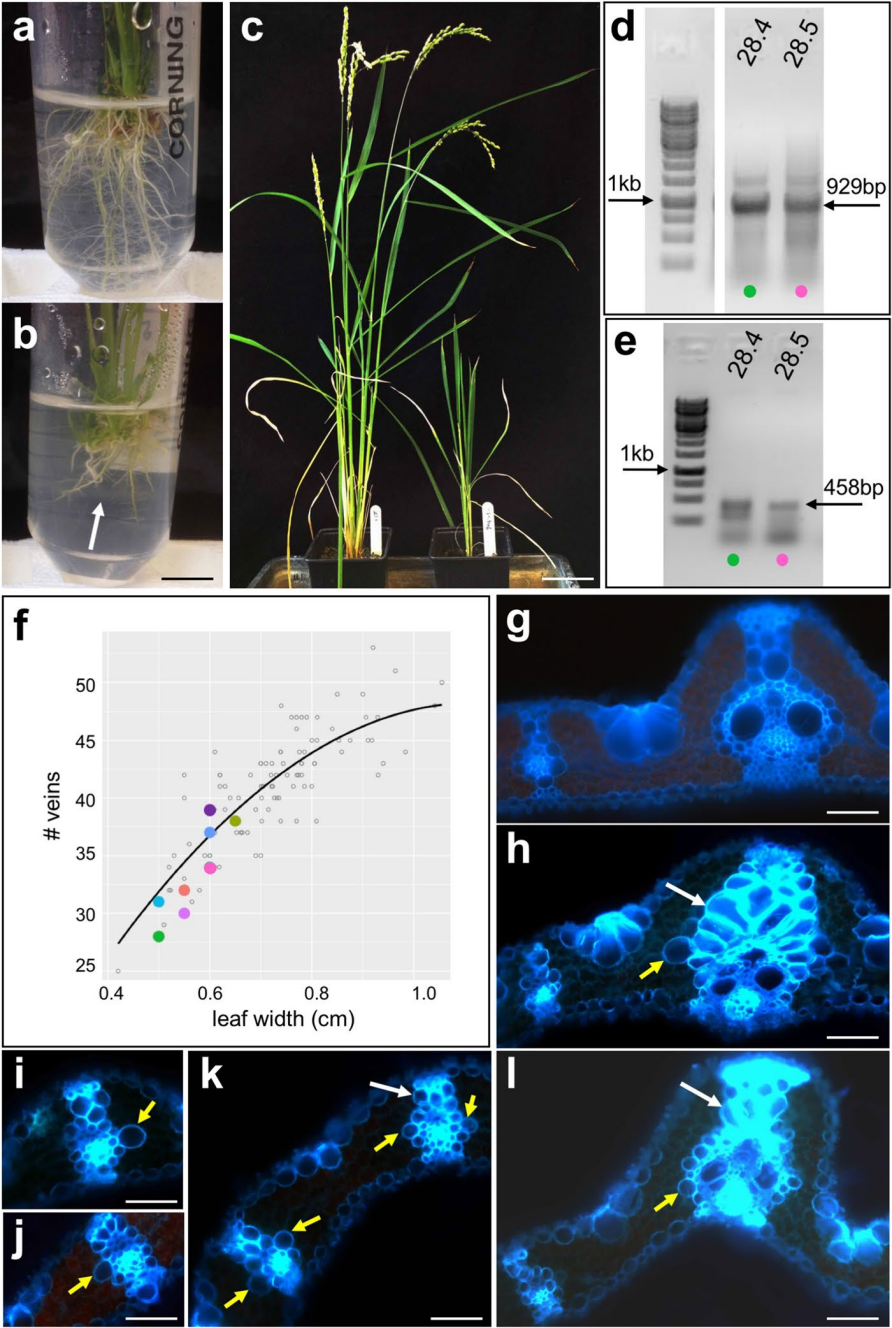
Transgenic lines overexpressing a protein of unknown function exhibit aberrant lignification around vascular bundles. (**a**–**c**) Compared to non-transgenic roots (**a**) and shoots (**c**) - left hand panel), growth of transgenic roots (**b**) and shoots (**c**)- right hand panel) is stunted. (**d**,**e**) Cropped gel images of genomic (**d**) and RT- (**e**) PCR reactions illustrate transgene presence and transcript levels in two representative T0 lines. (**f**) Regression plot showing vein number versus leaf width for 8 independent T0 lines. (**g**,**l**) Transverse cross sections of wild-type (**g**) and transgenic (**h**–**l**) leaves showing enhanced number of thicker walled sclerenchyma cells (white arrows) and enlarged and/or ectopic cells around veins (yellow arrows). Coloured circles in (**d**,**e**) illustrate corresponding datapoints in (**f**). Scale bars = 1 cm (**a**); 8 cm (**b**); 70 µm (**g**,**h**,**l**); 60 µm (**i**–**k**).

A novel gene with no functional annotation conditioned a striking phenotype in rice overexpression lines (Table 1 and Figs 4 and S9). Regenerating plants exhibited shorter roots with fewer laterals than non-transgenic siblings (Fig. 4a,b), and shoots were stunted and infertile (Fig. 4c). Vein spacing and number were unperturbed in the leaves of transgenic plants (Fig. 4f), however, transverse leaf sections revealed excessive secondary wall formation around vascular bundles (Fig. 4g–l). Specifically, more sclerenchyma cells (which have lignified cell walls) developed on the adaxial side of major leaf veins and the cells had thicker cell walls than in wild-type plants (Fig. 4g,h,k,l). In addition, one or two BS cells around each vascular bundle often appeared larger than the other cells (Fig. 4h–l).

### Inducible expression of *ZmWRKY12* inhibits lobing of mesophyll cells in riceN

**Figure 5:**
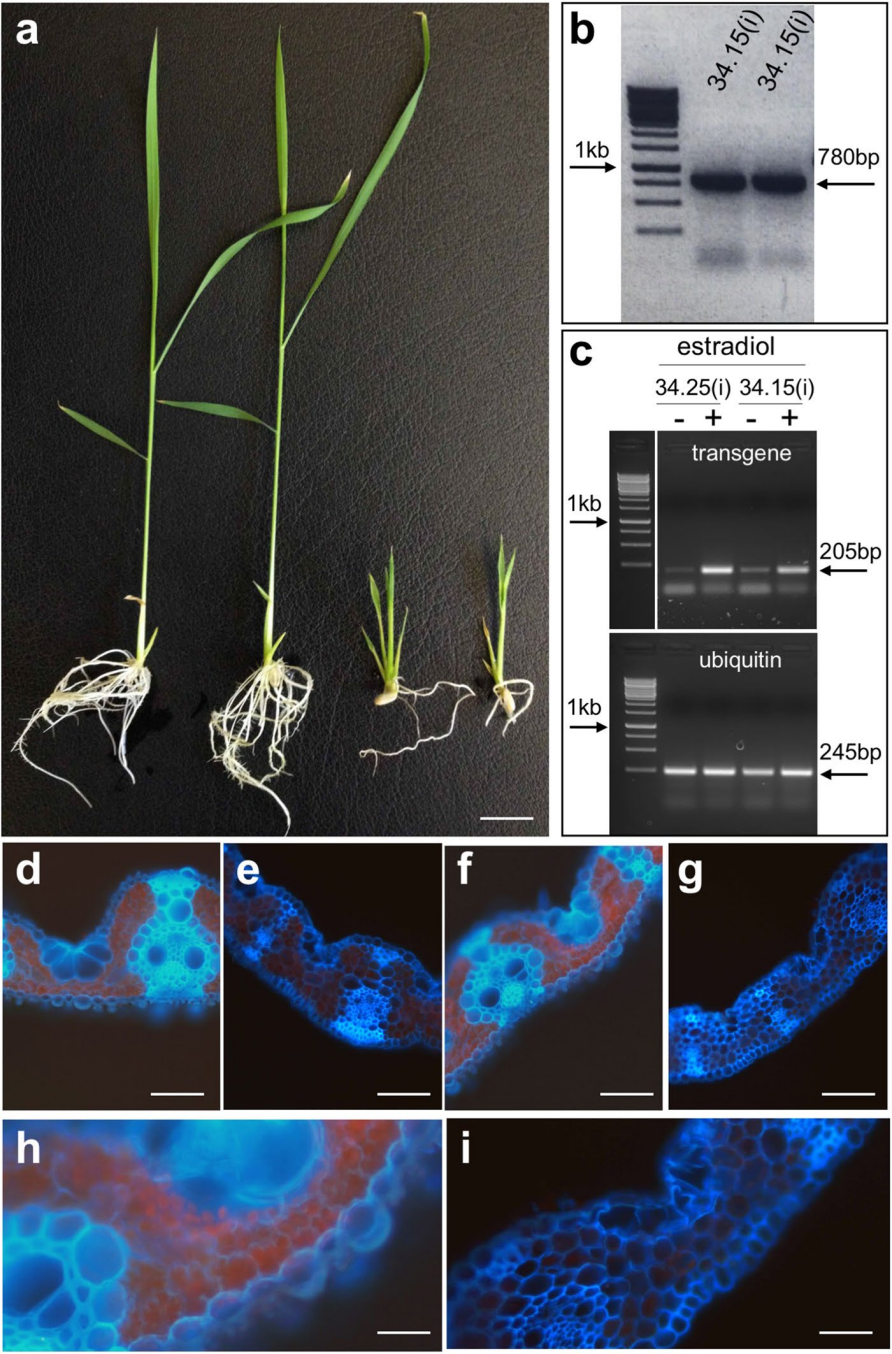
Expression of ZmWRKY12 inhibits cell wall growth in rice. (**a**) Non-transgenic plants (two left hand plants) are taller than plants in which expression of the transgene has been induced by germination on estradiol (two right hand plants). (**b**,**c**) Cropped gel images of genomic (**b**) and RT- (**c**) PCR reactions illustrate transgene presence in two individuals of T1 line 34.15(**i**) and enhanced transgene transcript levels (relative to ubiquitin) after exposure (+) of individuals from two independent T1 lines to estradiol. (**d**–**i**) Transverse cross sections of non-transgenic (**d**,**f**,**h**) and transgenic (**e**,**g**,**i**) young (**d**,**e**) and older (**f**–**i**); (**h**) and (**i**) are magnified images of (**f**) and (**g**) respectively) leaves show smaller, non-lobed mesophyll cells in transgenic plants. Scale bars = 1.5 cm (**a**); 75 µm (**d**–**g**); 25 µm (**h**,**i**).

Phylogenetic analysis of one of the candidate Kranz regulators revealed orthology to *AtWRKY12* in Arabidopsis (Supplementary Figure S10) and thus the maize gene was named *ZmWRKY12*. Rice callus transformed with *ZmWRKY12* driven by the constitutive ubiquitin promoter failed to regenerate, however, callus transformed with an estradiol-inducible transgene regenerated normally in the absence of estradiol and T1 seed were harvested. When T1 seed were germinated in the presence of estradiol, plants were severely dwarfed and had minimal root growth (Fig. 5a). This phenotype was consistent with increased *ZmWRKY12* transcript levels (relative to ubiquitin) as compared to untreated control plants (Fig. 5c). Quantitative analysis of vein number versus leaf width in estradiol-treated transgenic plants did not reveal any perturbations, however, transverse leaf sections revealed a mesophyll cell defect (Fig. 5d–i). Mesophyll cells in rice leaves are smaller than those in most other grasses but the cells have extensively lobed walls that effectively increase the surface area for CO_2_ diffusion^39^. Nothing is known about how this lobed shape is formed but mesophyll cells in the induced transgenic plants had no lobes (Fig. 5e,g,i). Given that *AtWRKY12* is a negative regulator of secondary cell wall formation in Arabidopsis^40^, it is likely that constitutive expression of *ZmWRKY12* prevented callus regeneration through an inability to develop vascular tissue (which requires the formation of secondary walls). More intriguingly, it appears as though the lobing of rice mesophyll cells may require secondary cell wall formation.

### Three maize ZnF homeodomain genes and an endogenous rice ortholog caused spindly growth and lodging when constitutively expressed

**Figure 6:**
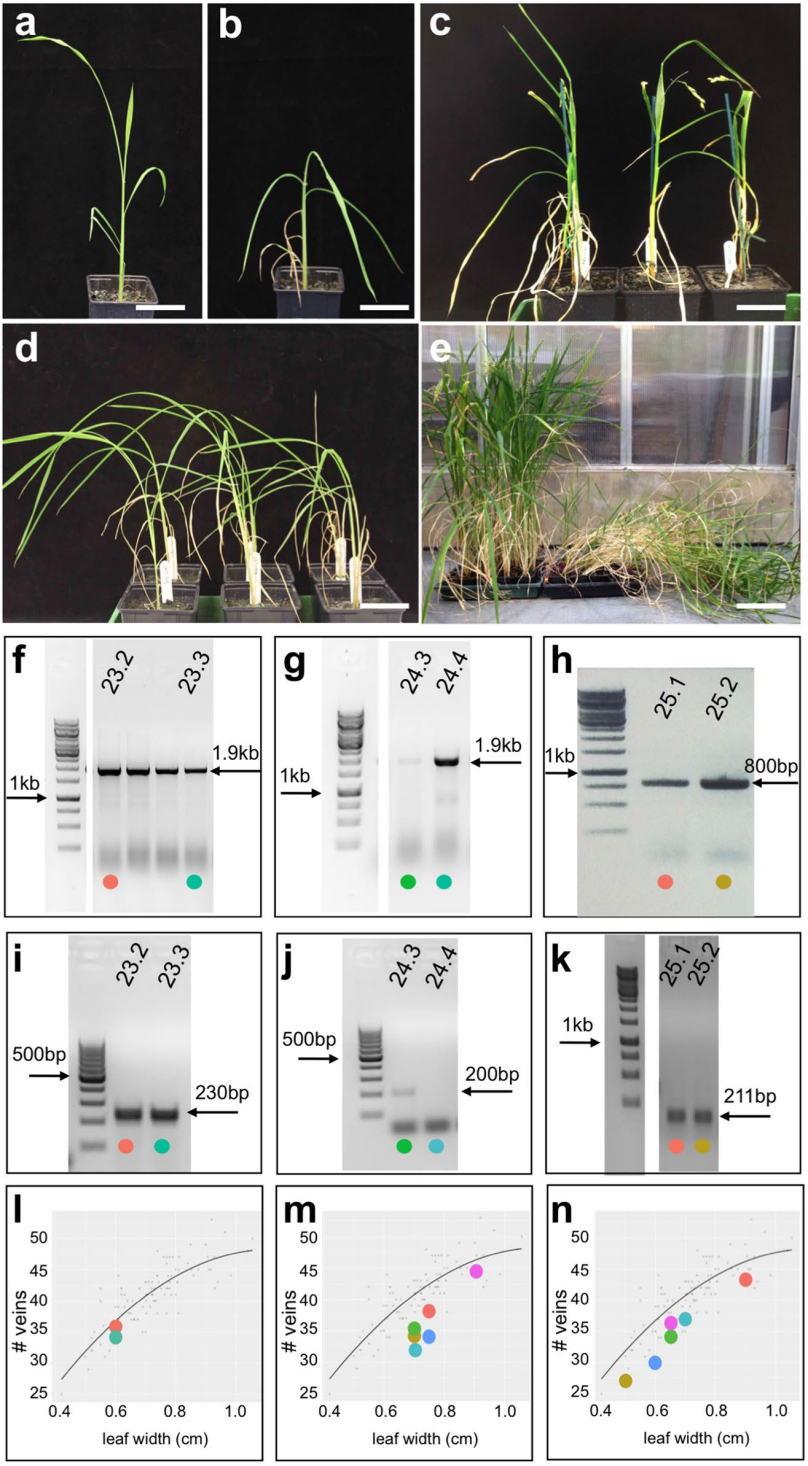
Ectopic expression of ZnF homeobox genes promotes spindly growth and lodging. (**a**–**d**) Transgenic plants expressing *ZmHBc* (**b**), *ZmHBa* (**c**) or *ZmHBb* (**d**) transgenes are spindly compared to non-transgenic controls (**a**). (**e**) Plants ectopically expressing the rice ortholog of *ZmHBc* exhibit severe lodging at maturity (right). Null segregants of the same age are on the left. (**f**–**k**) Cropped gel images of genomic (**f**,**g**,**h**) and RT- (**i**,**j**,**k**) PCR reactions illustrate transgene presence and transgene transcript levels in *ZmHBa* (**f**,**i**), *ZmHBb* (**g**,**j**) and *ZmHBc* (**h**,**k**) lines. (**l**–**n**) Regression plots showing vein number versus leaf width for two (**l** –*ZmHBa*) and six (**m** – *ZmHBb*; **n** – *ZmHBc*) individual T0 lines. Coloured circles in (**f**–**k**) correspond to datapoints in (**l**–**n**). Scale bars = 8 cm (**a**–**d**); 12 cm (**e**).

Lines that constitutively expressed any of three genes encoding closely related ZnF homeodomain (ZnF-HD) proteins exhibited drooping stems and leaves (Table 1 and Fig. 6a–d,f–k). None of the lines were strong enough to produce T1 seed but quantification of vein number versus leaf width in T0 plants revealed no differences from wild-type (Fig. 6l–n). A spindly shoot phenotype was also observed in an activation tagged line in which a related rice gene (Os03g50920) was ectopically expressed, but in this case T3 populations were obtained. Comparisons between homozygous lines with or without the activation tag revealed a dramatic lodging phenotype at maturity in plants that ectopically expressed the endogenous rice gene (Figs 6e and S11).

**Figure 7:**
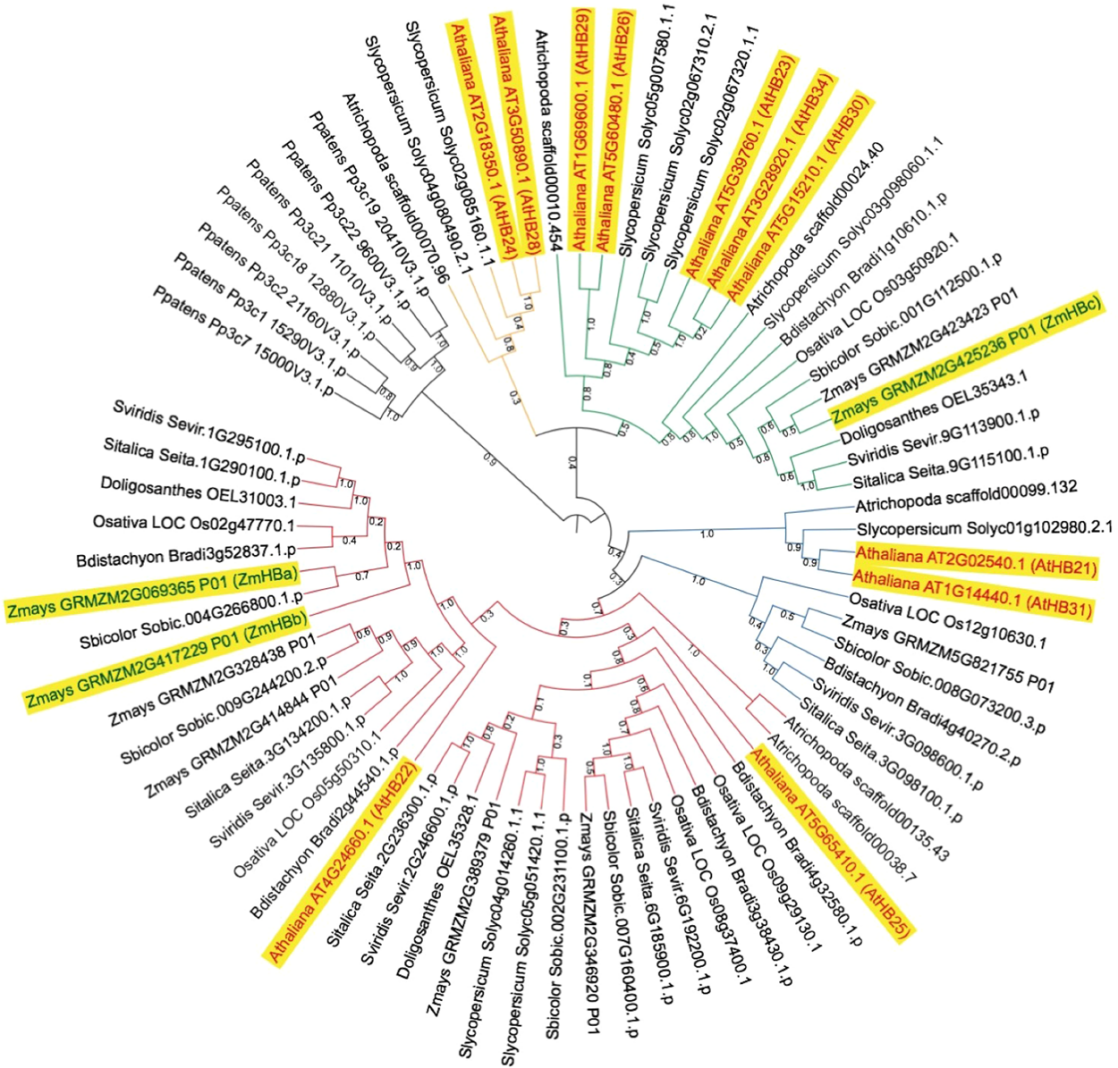
Phylogenetic tree of Zn finger homeodomain proteins. Maximum-likelihood tree of the ZnF-HD orthogroup (as defined by Orthofinder)^56^. Numbers at each node are support values based on the consensus tree of 10 bootstrap replicates. Sub-groups are denoted with different line colours. The Arabidopsis proteins (red text) plus the three maize proteins encoded by the genes analysed in this study (green text) are highlighted in yellow.

Phylogenetic analysis using a subset of monocot and eudicot species, and the moss *Physcomitrella patens* as an outgroup resolved five main clades of flowering plant ZnF-HD genes (Fig. 7) that were consistent with those previously reported for Arabidopsis genes in this family^41^. Two of the maize genes, referred to here as *ZmHBa* (GRMZM2G069365) and *ZmHBb* (GRMZM2G417229), were nested in a clade with *AtHB22* and *AtHB25* genes. Functional analysis in Arabidopsis revealed that *AtHB25* positively regulates *GIBBERLLIC ACID3-OXIDASE2* (*GA3OX2*), leading to increased levels of gibberellic acid (GA) in *AtHB25* gain of function lines, and that *AtHB25* and *AtHB22* act redundantly^42^. Increased GA levels would explain the spindly phenotype of transgenic rice lines overexpressing *ZmHBa* and *ZmHBb* genes (Fig. 6c,d), either through a direct effect of GA on cell expansion or through an inhibition of lignin content (as previously reported in *GA2OX* overexpression lines of switchgrass)^43^. The third maize gene, referred to here as *ZmHBc* (GRMZM2G425236), grouped with *AtHB23*, *AtHB26*, *AtHB29*, *AtHB30* and *AtHB34*. *ZmHBc* is orthologous to Os03g50920. Although the Arabidopsis orthologs of *ZmHBc* function in stress responses (where known)^44, 45^, perturbed GA levels could also explain the spindly phenotype of *ZmHBc* overexpression lines (Fig. 6b), and the lodging of lines ectopically expressing the corresponding rice ortholog (Fig. 6e).

### Summary

Constitutive expression of 60 maize genes in rice has revealed three phenotypic classes. The largest class was phenotypically indistinguishable from wild-type (Table 1 and Supplementary Information S1 and S3). More than 25 genes in this class encoded transcription factors which were expected to alter the expression of a number of downstream genes. The lack of phenotypic perturbation observed suggests either that plant developmental processes are buffered against changes in these particular transcriptional networks or that the maize genes cannot activate downstream targets in rice. Notably one of the genes in this class is *ZmSCARECROW1*, a gene which when mutated in maize causes subtle defects in Kranz anatomy^1^. Clearly *ZmSCR1* is not sufficient to induce Kranz-like traits in rice, at least not when expressed ubiquitously.

The second phenotypic class altered shoot and/or root growth in ways that inferred perturbed hormone signalling (Figs 1, 2 and 6), although other explanations are possible. In the six cases where gene expression either prevented callus regeneration (Table 1) or led to the regeneration of inviable plantlets (Fig. 1), the endogenous gene expression profile in maize was such that transcript levels were much lower in P5 leaf primordia than in younger P3/4 primordia^19^. This suggests that repression of gene expression at P5 is necessary for normal maize leaf development. Constitutive expression in transgenic rice plants might thus be expected to perturb shoot development. Notably a number of genes in this class are orthologs of genes involved in auxin biosynthesis or signalling in Arabidopsis, and the phenotype of transgenic lines is consistent with a similar role for the maize genes. Loss of function mutants need to be identified to confirm or refute this suggestion.

Phenotypes in the final class were associated with perturbed secondary wall formation (Figs 3, 4 and 5). Given that secondary wall formation is crucial during early leaf development it is not surprising that such genes were identified as potential regulators of C_4_ patterning. However, the gain of function phenotypes resulting from constitutive expression raise interesting questions about potential roles in regulating cellular differentiation more broadly. For example, the gain of function phenotype for one of these genes, *ZmWRKY12*, revealed a role for secondary cell walls in the formation of lobed mesophyll cells in rice (Fig. 3). This trait is characteristic of rice mesophyll cells but until now nothing was known about how lobing was achieved. Just as *ZmWRKY2* inhibited mesophyll cell lobing, constitutive expression of an R2R3 Myb gene suppressed sclerenchyma formation around veins (Fig. 4). A third gene defined a novel gene family which is monocot-specific. Gain of function in this case led to the formation of ectopic cell-types around veins (Fig. 5). These cells resembled extra sclerenchyma, but could possibly be bulliform-type cells. Unfortunately, there are no markers to distinguish these two cell-types. Further functional insight into the role of all three genes in cell-type differentiation requires the generation of loss of function mutants.

The development of Kranz anatomy in C_4_ plants is complex, minimally requiring altered leaf venation and cell-type differentiation in comparison to leaf development in ancestral C_3_ plants. Despite this complexity, the trait has evolved on over 60 independent occasions^5^. In this study we tested whether potential regulators of Kranz anatomy in maize were individually sufficient to alter leaf anatomy in rice when constitutively expressed. Although none of the genes elicited a shift towards Kranz-type venation patterning, in some cases cellular differentiation was perturbed. In other cases, phenotypic perturbations were so severe that potentially more subtle effects were obscured by the coarse nature of constitutive expression experiments. In these cases, further analyses of gene function need to be carried out (both necessity and sufficiency), exploiting different promoters for transgenic experiments (both inducible and cell-type specific), and also analyzing combinations of gene function. Importantly, as efforts continue to try and engineer rice leaf anatomy, 47 genes can be eliminated from further study because even with constitutive expression, no impact on rice leaf development was observed.

## Methods

### Rice germplasm

Transgenic lines were generated using *Oryza sativa* indica cultivar IR64 or japonica cultivar Kitaake. Activation tagged lines were previously generated in Tainung 67 and are part of the Taiwan Rice Insertional Mutant (TRIM) collection http://trim.sinica.edu.tw/ ^46^.

### Plant growth conditions

Kitaake and Tainung 67 lines were grown in soil (John Innes Compost No.2) in a transgenic greenhouse in Oxford. Day/night temperature was maintained at 30 °C/22 °C ± 3 °C with a diurnal light regime of 16 h light (supplemented to ~300 μM m^−2^ sec^−1^) and 8 h dark. IR64 lines were grown in soil in 7 L pots in a transgenic screenhouse under natural light conditions (maximum light intensity up to 2000 μM m^−2^ sec^−1^ on a sunny day) at the International Rice Research Institute, Los Banos, Philippines where day/night temperatures were 35 °C/28 °C ± 3 °C.

### DNA and RNA extraction

Genomic DNA from maize B73 or Kitaake rice lines was isolated using a modified CTAB method^47^. Genomic DNA from IR64 rice lines was isolated using a potassium acetate method^48^. Rice leaf RNA was isolated using TRIZOL reagent (Invitrogen) and maize leaf RNA using either TRIZOL or a mirVana™ miRNA isolation kit (Applied Biosystems), as described in ref. 19.

### Generation of transformation constructs

Coding sequences corresponding to each of the candidate genes was isolated by PCR amplification using Phusion High-Fidelity DNA Polymerase (Thermo Scientific) and Gateway® compatible primers (Supplementary Dataset S4). The template for all reactions was maize cDNA that had been generated using RNA isolated from P1-5 leaf primordia and a Transcriptor High Fidelity cDNA Synthesis Kit (Roche). For some genes, the same PCR conditions were used to amplify sequences from genomic DNA. The amplified sequences were subcloned into the Gateway® donor vectors pDONR™ 207 or pENTR/D-TOPO in a BP reaction. The resultant entry clones were sequenced, and the target sequences were then cloned in a LR reaction downstream of the maize ubiquitin promoter (*ZmUbipro*), into a destination vector modified from pVec8-GFP^49^ or into the destination vector pSC310. pSC310 vector was created by Sarah Covshoff and kindly gifted to us by Julian Hibberd (University of Cambridge, Cambridge, UK). Estradiol inducible constructs were generated by an LR reaction between entry clones and the destination vector pMDC7^50^. All constructs were given a construct ID and a ‘JL’ gene ID.

### Rice transformation

Callus induced from mature rice seeds was used for transformation of the Kitaake cultivar with *Agrobacterium tumefaciens* strain EHA105 carrying the construct of interest. Callus induction, transformant selection and seedling regeneration were performed at 32 °C under continuous light according to a protocol modified from^51^ (available to download from https://langdalelab.files.wordpress.com/2015/07/kitaake_transformation_2015.pdf). Hygromycin resistant T0 seedlings that confirmed positive for transgene presence by PCR screening (see below) were transplanted into soil in 0.73 L pots. For IR64, immature embryos were used for agrobacterium mediated transformation using *Agrobacterium tumefaciens* strain LBA4404 carrying the construct of interest. Callus induction, selection of transformed callus and plantlet regeneration were performed at 30 °C under continuous light according to a protocol modified from^52^.

### Estradiol induced gene expression

For transgene expression analysis, 4^th^ leaves from plants of the inducible lines JL34.15 and JL34.25 were detached and allowed to take up 2 μM β-estradiol or DMSO mock solution by transpiration. Total RNA was extracted after 24 h of treatment at 28 °C in the light, and RT-PCR carried out (see below). For phenotypic characterization of estradiol inducible lines, seeds were germinated alongside wild-type controls on filter paper wetted with 2 μM β-estradiol solution. After germination, plants were cultured in 1/2 MS liquid medium with the same β-estradiol concentration, and images were taken 10 days after germination. Liquid cultures were grown in a growth cabinet with cycles of 28 °C/16 h light and 23 °C/8 h dark.

### Genomic PCR screening and RT-PCR

For Kitaake lines, regenerated T0 plants and T1 seedlings (2–3 weeks after germination) were subjected to genomic PCR using primers specific to the cloning vector: pVec8F (TTTAGCCCTGCCTTCATACG, located in the *ZmUBI*_*pro*_), and pVec8R (ATTGCCAAATGTTTGAACGA, located in the *nos* terminator). PCR amplification was performed in a total reaction volume of 10 µl containing 5 ul 2xGoTaq® mix (Promega) and 2.5 ul 4 M betaine. PCR conditions were: 95 °C for 5 min; 28 cycles of 95 °C for 30 s, 55 °C for 40 s, 72 °C for 2.5 min; and 72 °C for 5 min. For IR64 lines, regenerated T0 plants and T1 seedlings (2 weeks after germination) were subjected to genomic PCR using gene specific primers (Supplementary Dataset S4). PCR amplification was performed in a total reaction volume of 10 µl containing 5 µl 2xKAPA Plant PCR buffer, 0.1 µl KAPA3G Plant DNA polymerase (KAPABIOSYSTEMS Inc.) and 3.1 µl distilled water. PCR conditions were: 95 °C for 5 min; 32 cycles of 95 °C for 20 s, 60 °C for 15 s, 72 °C for 1 min; and 72 °C for 1 min.

Unless otherwise noted, gene expression analysis was carried out using RNA extracted from fully expanded 4^th^ leaf tissue. Total RNA was treated with RQ1 RNAse free DNAse (Promega, USA) and cDNA was synthesized using Superscript® III reverse transcriptase (Invitrogen) according to the manufacturer’s instructions. Primers specific to the gene of interest (Supplementary Dataset S4) and 2xGoTaq® mix (Promega) were used in a 10 µl PCR reaction volume to detect the expression of transgenes and/or endogenous rice genes. All PCR products were detected by agarose gel electrophoresis using standard protocols^53^.

### DNA gel blot analysis

For each sample, 6–8 µg of genomic DNA was digested with a restriction endonuclease that had a single cut site in the construct (37 °C for 12–16 h). Digested DNA samples were electrophoresed overnight at 25 volts on a 0.8% agarose gel in 1X TAE buffer and then blotted onto Hybond N^+^ membrane (GE Healthcare, UK) overnight using 20x SSC as transfer buffer. Blots were hybridised with a digoxigenin (DIG) labeled fragment of the *ZmUbi* promoter that was synthesised using the PCR DIG Probe Synthesis Kit (Roche Diagnostics, Germany). DNA hybridisation signals were detected using CDP-*Star* (Roche Diagnostics, Germany).

### Quantitative RT-PCR

PCR amplification was carried out using GoTaq Hot Start polymerase (Promega) and amplification detected with 1/60,000 SYBR Green II (Sigma-Aldrich) and the Mx3000 P QPCR System (Agilent). The thermal profile ran as follows: 95 °C, 10 minutes; (95 °C, 15 seconds, 60 °C, 30 seconds, 72 °C, 30 seconds) × 45 cycles. Three technical replicates were carried out per sample and transcript abundance was normalized to the endogenous rice *UBIQUITIN* gene (Os03g13170).

### Phenotypic analysis

Phenotypic analysis was carried out on the expanded 6^th^ leaf of the first tiller. Leaf width and length plus stomatal number on adaxial and abaxial leaf surfaces were first measured and then fresh segments were hand cut from the middle of the leaf blade. For Kitaake lines, segments were embedded in 5%(w/v) agar and sectioned at 70–80 μm with a Vibratome Series 1000 Sectioning System. Sections were viewed and photographed with a Leica DMRB microscope. For IR64 lines, segments were fixed in 2.5 glutaraldehyde, cleared and then stained with Toluidine blue. Hand sections were captured using an Olympus BX51 microscope with a DP71 camera. The cell number between a pair of intermediate veins positioned between the second and third lateral vein in from the leaf margin was recorded for each sample, and the presence or absence of large BS chloroplasts and of M cell invaginations was also noted.

### Quantification of vein spacing

The number of veins across the medio-lateral leaf axis was counted either using the Leica DMRB microscope and transverse cross-sections, or using a stereomicroscope and photomicrographs of the leaf surface. 2–5 leaf samples were quantified for each line. Least-squares regression lines of leaf width versus vein number were plotted for both the Kitaake and IR64 datasets using the R stats package^54^. To maximize the R-squared value, a second order polynomial line was fitted to the Kitaake data, whilst a linear model was used for IR64 data. Data and regression lines were visualized using the R ggplot2 package^55^.

### Phylogenetic analysis

OrthoFinder^56^ was run over proteome-wide protein sequences of eleven species to identify the groups of orthologous genes (OrthoGroups (OGs)) across them. Species included the eudicots *Arabidopsis thaliana* and *Solanum lycopersicum*, the monocots *Zea Mays*, *Sorghum bicolor*, *Setaria italica*, *Setaria viridis*, *Dichanthelium oligosanthes*, *Oryza sativa*, *Brachypodium distachyon*, the basal angiosperm *Amborella trichopoda*, and the moss *Physcomitrella patens*. The OGs which contained the maize genes of interest were selected, and then protein sequences were aligned by MAFFT-lins^57^. For the ZnF HD phylogeny (Fig. 7), three sequences were removed from the dataset before alignment (*D*. *oligosanthes* OEL17202.1 & OEL35852.1, plus A. *trichopoda* scaffold00011.170) because the genome annotation appeared spurious. Maximum Likelihood phylogenetic trees for each OG were produced from these alignments using IQ-TREE software^58^, and then consensus trees were finally generated by SUMTREES^59^ with the Maximum Clade Credibility Topology (MCCT) algorithm.

### Accession codes

All accession codes are included in Table 1, and complete sequences are included in Supplementary Information S1.

## Electronic supplementary material

Supplementary Information

Supplementary Dataset 1

Supplementary Dataset 2

Supplementary Dataset 3

Supplementary Dataset 4

## References

[1] Slewinski TL, Anderson AA, Zhang C, Turgeon R (2012). Scarecrow plays a role in establishing kranz anatomy in maize leaves. Plant Cell Physiol..

[2] Slewinski TL (2014). Short-root1 plays a role in the development of vascular tissue and Kranz anatomy in maize leaves. Mol. Plant.

[3] Langdale JA (2011). C_4_ cycles: past, present, and future research on C_4_ photosynthesis. Plant Cell.

[4] Haberlandt, G. *Physiologische Pflanzenanatomie*. (Wilhelm Engelman, 1896).

[5] Sage RF, Christin P-A, Edwards EJ (2011). The C_4_ plant lineages of planet Earth. J. Exp. Bot..

[6] Wang L (2014). Comparative analyses of C_4_ and C_3_ photosynthesis in developing leaves of maize and rice. Nat. Biotechnol..

[7] Li P (2010). The developmental dynamics of the maize leaf transcriptome. Nat. Genet..

[8] Pick TR (2011). Systems analysis of a maize leaf developmental gradient redefines the current C_4_ model and provides candidates for regulation. Plant Cell.

[9] Liu W-Y (2013). Anatomical and transcriptional dynamics of maize embryonic leaves during seed germination. Proc. Natl. Acad. Sci..

[10] Li, Y. *et al*. Developmental genetic mechanisms of C_4_ syndrome based on transcriptome analysis of C_3_ cotyledons and C_4_ assimilating shoots in Haloxylon ammodendron. *PLoS One* **10** (2015). 10.1371/journal.pone.0117175 PMC4313948 25643361

[11] Yu CP (2015). Transcriptome dynamics of developing maize leaves and genomewide prediction of cis elements and their cognate transcription factors. Proc Natl Acad Sci USA.

[12] Lauterbach M (2017). C_3_ cotyledons are followed by C_4_ leaves: intra-individual transcriptome analysis of Salsola soda (Chenopodiaceae). J. Exp. Bot..

[13] Brautigam A (2011). An mRNA blueprint for C_4_ photosynthesis derived from comparative transcriptomics of closely related C_3_ and C_4_ species. Plant Physiol..

[14] Gowik U, Brautigam A, Weber KL, Weber AP, Westhoff P (2011). Evolution of c4 photosynthesis in the genus flaveria: how many and which genes does it take to make c4?. Plant Cell.

[15] Aubry S, Kelly S, Kumpers BM, Smith-Unna RD, Hibberd JM (2014). Deep evolutionary comparison of gene expression identifies parallel recruitment of trans-factors in two independent origins of C_4_ photosynthesis. PLoS Genet..

[16] Rao X (2016). Comparative cell-specific transcriptomics reveals differentiation of C_4_ photosynthesis pathways in switchgrass and other C_4_ lineages. J. Exp. Bot..

[17] Ding, Z. *et al*. Identification of photosynthesis-associated C_4_ candidate genes through comparative leaf gradient transcriptome in multiple lineages of C_3_ and C_4_ species. *PLoS One* **10** (2015). 10.1371/journal.pone.0140629 PMC4605685 26465154

[18] Kümpers BMC (2017). Shared characteristics underpinning C_4_ leaf maturation derived from analysis of multiple C_3_ and C_4_ species of Flaveria. J. Exp. Bot..

[19] Wang P, Kelly S, Fouracre JP, Langdale JA (2013). Genome-wide transcript analysis of early maize leaf development reveals gene cohorts associated with the differentiation of C_4_ Kranz anatomy. Plant J..

[20] Langdale JA, Rothermel BA, Nelson T (1988). Cellular patterns of photosynthetic gene expression in developing maize leaves. Genes Dev..

[21] Fouracre JP, Ando S, Langdale JA (2014). Cracking the Kranz enigma with systems biology. J. Exp. Bot..

[22] Costanzo, M. *et al*. A global genetic interaction network maps a wiring diagram of cellular function. *Science* (*80-*.*)*. **353** (2016). 10.1126/science.aaf1420 PMC5661885 27708008

[23] Cui, D. *et al*. The Arabidopsis IDD14, IDD15, and IDD16 Cooperatively Regulate Lateral Organ Morphogenesis and Gravitropism by Promoting Auxin Biosynthesis and Transport. *PLoS Genet*. **9** (2013). 10.1371/journal.pgen.1003759 PMC3764202 24039602

[24] Wu X, Tang D, Li M, Wang K, Cheng Z (2013). Loose Plant Architecture1, an INDETERMINATE DOMAIN protein involved in shoot gravitropism, regulates plant architecture in rice. Plant Physiol..

[25] Ahmad, A. *et al*. BHLH106 integrates functions of multiple genes through their g-box to confer salt tolerance on Arabidopsis. *PLoS One* **10** (2015). 10.1371/journal.pone.0126872 PMC4433118 25978450

[26] Guo Y, Qin G, Gu H, Qu L-J (2009). Dof5.6/HCA2, a Dof transcription factor gene, regulates interfascicular cambium formation and vascular tissue development in Arabidopsis. Plant Cell.

[27] Woodward C (2005). Interaction of auxin and ERECTA in elaborating Arabidopsis inflorescence architecture revealed by the activation tagging of a new member of the YUCCA family putative flavin monooxygenases. Plant Physiol..

[28] Suer S, Agusti J, Sanchez P, Schwarz M, Greb T (2011). WOX4 imparts auxin responsiveness to cambium cells in Arabidopsis. Plant Cell.

[29] Yamamoto Y, Kamiya N, Morinaka Y, Matsuoka M, Sazuka T (2007). Auxin biosynthesis by the YUCCA genes in rice. Plant Physiol..

[30] Chen Y, Hao X, Cao J (2014). Small auxin upregulated RNA (SAUR) gene family in maize: Identification, evolution, and its phylogenetic comparison with Arabidopsis, rice, and sorghum. J. Integr. Plant Biol..

[31] Kant S, Bi YM, Zhu T, Rothstein SJ (2009). SAUR39, a small auxin-up RNA gene, acts as a negative regulator of auxin synthesis and transport in rice. Plant Physiol..

[32] Vera-Sirera F (2015). A bHLH-Based Feedback Loop Restricts Vascular Cell Proliferation in Plants. Dev. Cell.

[33] De Rybel B (2014). Plant development. Integration of growth and patterning during vascular tissue formation in Arabidopsis. Science.

[34] Ohashi-Ito K (2014). A bHLH complex activates vascular cell division via cytokinin action in root apical meristem. Curr. Biol..

[35] ten Hove CA (2011). Probing the roles of LRR RLK genes in *Arabidopsis thaliana* roots using a custom T-DNA insertion set. Plant Mol. Biol..

[36] Kumar, D. *et al*. ARABIDOPSIS THALIANA RECEPTOR DEAD KINASE1 Functions as a Positive Regulator in Plant Responses to ABA. *Mol*. *Plant* doi:10.1016/j.molp.2016.11.011. 10.1016/j.molp.2016.11.011 27923613

[37] Pastore JJ (2011). LATE MERISTEM IDENTITY2 acts together with LEAFY to activate APETALA1. Development.

[38] Brockington SF (2013). Evolutionary analysis of the MIXTA gene family highlights potential targets for the study of cellular differentiation. Mol. Biol. Evol..

[39] Sage TL, Sage RF (2009). The Functional Anatomy of Rice Leaves: Implications for Refixation of Photorespiratory CO_2_ and Efforts to Engineer C_4_ Photosynthesis into Rice. Plant Cell Physiol..

[40] Wang H (2010). Mutation of WRKY transcription factors initiates pith secondary wall formation and increases stem biomass in dicotyledonous plants. Proc. Natl. Acad. Sci. USA.

[41] Tan QK-G, Irish VF (2006). The Arabidopsis zinc finger-homeodomain genes encode proteins with unique biochemical properties that are coordinately expressed during floral development. Plant Physiol..

[42] Bueso E (2013). ARABIDOPSIS THALIANA HOMEOBOX 25 uncovers a role for gibberellins in seed longevity. Plant Physiol..

[43] Wuddineh WA (2015). Identification and overexpression of gibberellin 2-oxidase (GA2ox) in switchgrass (Panicum virgatum L.) for improved plant architecture and reduced biomass recalcitrance. Plant Biotechnol. J..

[44] Choi H (2014). The homeodomain-leucine zipper ATHB23, a phytochrome B-interacting protein, is important for phytochrome B-mediated red light signaling. Physiol. Plant..

[45] Tran LSP (2007). Co-expression of the stress-inducible zinc finger homeodomain ZFHD1 and NAC transcription factors enhances expression of the ERD1 gene in Arabidopsis. Plant J..

[46] Lo S-F (2016). Genetic resources offer efficient tools for rice functional genomics research. Plant. Cell Environ..

[47] Murray MG, Thompson WF (1980). Rapid isolation of high molecular weight plant DNA. Nucleic Acids Res.

[48] Guillemaut P, Maréchal-Drourd L (1992). Isolation of plant DNA: a fast, inespensive, and reliable method. Plant Mol. Biol. Report..

[49] Kim, C. M. & Dolan, L. ROOT HAIR DEFECTIVE SIX-LIKE Class I Genes Promote Root Hair Development in the Grass Brachypodium distachyon. *PLoS Genet*. **12** (2016). 10.1371/journal.pgen.1006211 PMC4975483 27494519

[50] Curtis MD, Grossniklaus U (2003). A gateway cloning vector set for high-throughput functional analysis of genes in planta. Plant Physiol.

[51] Toki S (2006). Early infection of scutellum tissue with *Agrobacterium* allows high-speed transformation of rice. Plant J..

[52] Hiei Y, Komari T (2006). Improved protocols for transformation of indica rice mediated by *Agrobacterium tumefaciens*. Plant Cell. Tissue Organ Cult..

[53] Sambrook, J., Fritsch, E. F., Maniatis, T. & Rich, E. F. *Molecular cloning: a laboratory manual*. (Cold Spring Harbor Press, 1982).

[54] Team, R. R Development Core Team. *R A Lang*. *Environ*. *Stat*. *Comput*. **55**, 275–286 (2015).

[55] Wickham, H. *ggplot2*. *Elegant Graphics for Data Analysis* doi:10.1007/978-0-387-98141-3 (2009).

[56] Emms DM, Kelly S (2015). OrthoFinder: solving fundamental biases in whole genome comparisons dramatically improves orthogroup inference accuracy. Genome Biol..

[57] Katoh K, Standley DM (2013). MAFFT multiple sequence alignment software version 7: Improvements in performance and usability. Mol. Biol. Evol..

[58] Nguyen LT, Schmidt HA, Von Haeseler A, Minh BQ (2015). IQ-TREE: A fast and effective stochastic algorithm for estimating maximum-likelihood phylogenies. Mol. Biol. Evol.

[59] Sukumaran J, Holder MT (2010). DendroPy: A Python library for phylogenetic computing. Bioinformatics.

