# Supplementary material for: Candidate regulators of Early Leaf Development in Maize Perturb Hormone Signalling and Secondary Cell Wall Formation When Constitutively Expressed in Rice
Source: Sci Rep. 2017 Jul 3;7:4535. doi: 10.1038/s41598-017-04361-w (PMC5495811; doi:10.1038/s41598-017-04361-w)

SUPPLEMENTARY INFORMATION FILE

CANDIDATE REGULATORS OF EARLY LEAF DEVELOPMENT IN MAIZE PERTURB HORMONE SIGNALLING AND SECONDARY CELL WALL FORMATION WHEN CONSTITUTIVELY EXPRESSED IN RICE

Peng Wang, Shanta Karki, Akshaya K. Biswal, Hsiang-Chun Lin, Mary Jacqueline Dionora, Govinda Rizal, Xiaojia Yin, Mara L. Schuler, Tom Hughes, Jim P. Fouracre, Basel Abu Jamous, Olga Sedelnikova, Shuen-Fang Lo, Anindya Bandyopadhyay, Su-May Yu, Steven Kelly, W. Paul Quick and Jane A. Langdale

LEGENDS

Dataset S1 (xls). Data used for regression analysis of vein number versus leaf width (cm). Corresponding non-transgenic control samples are listed as 'regression' in each of 4 datasets (Kitaake T1 lines - leaf 6 & 8; IR64 T0 lines - leaf 6; IR64 T1 lines - leaf 6; IR64 T0 lines - penultimate leaf).

Dataset S2 (xls). Phenotyping data for IR64 T0 lines, showing measurements of vein number, and leaf width. Transgene presence as determined by PCR is indicated.

Dataset S3 (xls). Phenotyping data for Kitaake & IR64 T1 lines, showing measurements of vein number, distance between veins, cell number between veins and bundle sheath cell size. Transgene transcript level as determined by PCR is shown where measured.

Dataset S4 (xls). Primer sequences used for cloning and for RT-PCR.

Information S1. Sequences of all 60 genes transformed into rice.

Information S2. Individual datasheets for T0 rice lines summarized in Dataset S2. Each page shows the gene expression profile in maize foliar and husk leaf primordia as reported in [1] (green circles refer to relative expression level in samples of the shoot apical meristem plus plastochron 1/2 leaf primordia, orange circles to levels in P3/4 leaf primordia and blue circles to levels in P5 leaf primordia); construct composition and ID; number and ID of independent transgenic rice lines. Cropped agarose gel images show PCR products of representative genomic PCR reactions (detecting presence of transgene). Linear regression plots illustrate number of veins versus leaf width in each line (x axis = leaf width in cm; y axis = vein number). Representative leaf samples are shown with views of the adaxial leaf surface taken using a hand-held microscope.

Information S3. Individual datasheets for T1 rice lines summarized in Dataset S3. Each page shows the gene expression profile in maize foliar and husk leaf primordia as reported in [1] (green circles refer

to relative expression level in samples of the shoot apical meristem plus plastochron 1/2 leaf primordia, orange circles to levels in P3/4 leaf primordia and blue circles to levels in P5 leaf primordia); construct composition and ID; number and ID of independent transgenic rice lines. Where available, cropped agarose gel images show PCR products of representative genomic PCR reactions (detecting presence of transgene) and RT-PCR reactions (detecting transgene transcripts). qRT-PCR data are shown for one construct. Polynomial (Kitaake lines) or linear (IR64 lines) regression plots illustrate number of veins versus leaf width in each line. Representative leaf samples (either hand cut transverse leaf sections viewed by fluorescence microscopy or views of the adaxial leaf surface taken using a hand-held microscope) are shown in each case. Fluorescence images are visualized through autofluorescence of chlorophyll (red) and lignin (blue).

Figure S1. Phylogenetic tree of GRMZM2G074032 (*ZmIDD16*). Maximum-likelihood tree of the GRMZM2G074032 orthogroup (as defined by Orthofinder [2]). Numbers at each node are support values based on the consensus tree of 10 bootstrap replicates. Arabidopsis and rice proteins, plus the maize protein encoded by the gene analysed in this study are highlighted in yellow. Branch lengths reflect the number of amino acid substitutions per site.

Figure S2. Phylogenetic tree of GRMZM2G027068 (*ZmbHLH106*). Maximum-likelihood tree of the GRMZM2G027068 orthogroup (as defined by Orthofinder [2]). Numbers at each node are support values based on the consensus tree of 10 bootstrap replicates. Arabidopsis proteins, plus the maize protein encoded by the gene analysed in this study are highlighted in yellow. Branch lengths reflect the number of amino acid substitutions per site.

Figure S3. Phylogenetic tree of GRMZM2G140694 (*ZmHCA2*). Maximum-likelihood tree of one of the sub-groups of the GRMZM2G140694 orthogroup (as defined by Orthofinder [2]). Numbers at each node are support values based on the consensus tree of 10 bootstrap replicates. Arabidopsis HCA2, plus the maize protein encoded by the gene analysed in this study are highlighted in yellow. Branch lengths reflect the number of amino acid substitutions per site.

Figure S4. Phylogenetic tree of GRMZM2G480386 (YUCCA). Maximum-likelihood tree of the GRMZM2G480386 orthogroup (as defined by Orthofinder [2]). Numbers at each node are support values based on the consensus tree of 10 bootstrap replicates. Rice YUCCA8 plus the maize protein encoded by the gene analysed in this study are highlighted in yellow. Branch lengths reflect the number of amino acid substitutions per site.

Figure S5. Phylogenetic tree of GRMZM2G011463 (*ZmSAUR60*). Maximum-likelihood tree of the GRMZM2G011463 orthogroup (as defined by Orthofinder [2]). Numbers at each node are support values based on the consensus tree of 10 bootstrap replicates. Rice and Arabidopsis proteins, plus the maize protein encoded by the gene analysed in this study are highlighted in yellow. Branch lengths reflect the number of amino acid substitutions per site.

Figure S6. Phylogenetic tree of GRMZM2G098988 (*ZmSACL3*). Maximum-likelihood tree of the GRMZM2G098988 orthogroup (as defined by Orthofinder [2]). Numbers at each node are support values based on the consensus tree of 10 bootstrap replicates. Arabidopsis SACL3, plus the maize protein encoded by the gene analysed in this study are highlighted in yellow. Branch lengths reflect the number of amino acid substitutions per site.

Figure S7. Phylogenetic tree of GRMZM2G163724 (LRR kinase). Maximum-likelihood tree of the GRMZM2G163724 orthogroup (as defined by Orthofinder [2]). Numbers at each node are support values based on the consensus tree of 10 bootstrap replicates. Arabidopsis RDK1, plus the maize protein encoded by the gene analysed in this study are highlighted in yellow. Branch lengths reflect the number of amino acid substitutions per site.

Figure S8. Phylogenetic tree of GRMZM2G111045 (R2R3 MYB). Maximum-likelihood tree of the GRMZM2G111045 orthogroup (as defined by Orthofinder [2]). Numbers at each node are support values based on the consensus tree of 10 bootstrap replicates. Arabidopsis LMI2, plus the maize

protein encoded by the gene analysed in this study are highlighted in yellow. Branch lengths reflect the number of amino acid substitutions per site.

Figure S9. Phylogenetic tree of GRMZM2G114893 (Unknown). Maximum-likelihood tree of the GRMZM2G114893 orthogroup (as defined by Orthofinder [2]). Numbers at each node are support values based on the consensus tree of 10 bootstrap replicates. The maize protein encoded by the gene analysed in this study is highlighted in yellow. Branch lengths reflect the number of amino acid substitutions per site.

Figure S10. Phylogenetic tree of GRMZM2G377217 (*ZmWRKY12*). Maximum-likelihood tree of one of the sub-groups of the GRMZM2G377217 orthogroup (as defined by Orthofinder [2]). Numbers at each node are support values based on the consensus tree of 10 bootstrap replicates. Arabidopsis proteins plus the maize protein encoded by the gene analysed in this study are highlighted in yellow. Branch lengths reflect the number of amino acid substitutions per site.

Figure S11. Details of activation tagged line M0094935. **a)** Schematic of rice chromosome 3 showing insertion site of T-DNA (red arrow) upstream of Os03g50920 in line M0094935. **b)** Gel images of RT-PCR reactions showing transcript levels of 03g50920 relative to actin in 21 day old seedling shoots of wild-type (WT) and the insertion line (M0094935).

REFERENCES

1. Wang, P., Kelly, S., Fouracre, J. P. & Langdale, J. A. Genome-wide transcript analysis of early maize leaf development reveals gene cohorts associated with the differentiation of C4 Kranz anatomy. *Plant J.* **75,** 656–670 (2013).
2. Emms, D. M. & Kelly, S. OrthoFinder: solving fundamental biases in whole genome comparisons dramatically improves orthogroup inference accuracy. *Genome Biol.* **16,** 157 (2015).

Information File S1. Sequences of all 60 genes transformed into rice.

>JL2__GRMZM2G028046
ATGAGCAGAGGAGCTGAGAAGAAGAATTGGCCGCCGTCTCGGTCTTCTTCCTCCTCCTCTTCTT
CACCGACGTCGTCGTGGGTCGCCAGCAGGTCCATGGCGGGGTTCTACCGCTACCCGTCCTCCA
CTCGCACCTCCGGCAAGGACAGAACCCAGCCGCCGCCAGCACCAGCTGCACCGACACAACAA
CAAGGCGACAAGAGCAGCAGCAAGAAGAAGAGGAGGAGTATCAGCATCGGCCGGAGCATCAC
GTGCGCGGGCTCCATCTGCAGTACCAAGGAGAGCTCGGTCATGAGCCGGGACCGCCGCGGCG
CCTCCAGCAGGTCGCTCAGGGCACCCTACGTCGACGTCGACGTCAACGACGCCTCTGCCGCC
GCCATCTCCGCCACGTCGTCCTTCAACTCGGAGACCACCGTGGCGTCGACGACCACCGTCACG
TCCTCGTCCTCGTCCTCGTCCGTCGGGACCTCCTTCAGGGGCGTGCACATCAGGAAGCTCTCC
GGCTGCTACGAGTGCCACTCCGTCTTCGACCCCAGGAGCTTCGGCGCCGCCGCCTTCCCCTGC
GCGGACTGCGACGAGGTGTTCGCCAAGGCAGAGTCGCTAGAGCTCCACAGAGCAACCAGGCA
TGCAGTTTGGGAGCTGGGCCCCGAGGACACGAGCAGGAACATCGTGGAGATCATCTTCCAGTC
CAGCTGGCCGGCCGGGAAGCAGCAGCAGGCGCCGGTCTGCAAGATCGAGCGGGTACTCAAGG
TGCAGAGCAGCGACAGGACCCTGGAGAGGTTCGAGCAGTACAAGGAGAGCGTCAGGGAGAGG
GCGAGCAGCGACCGGGGCAGGAAGAACAACCCTCGGTGCGTCGCCGACGGCAACGAGCTCCT
CAGGTTCCACTGCACCACCTTCACCTGCTCCCTCGGCCTCGCCGGCAGCACCGCGCTGTGCCG
GGCGCCGCCGTGCAAGCTGTGCGCCATCGTCAGGGACGGTTTCAGGGTCGACGACGACGGTA
AGATTGCGACGATGGGCACCAGCGGGCGCGCCCACGACATGGCGCAGGCGGTGCTGTCGGA
GTCGGACGGTGACAAGAGAGCAATGCTCGTGTGCAGGGTCGTCGCGGGGAGGGTGAAGAAGA
AGGCCAGCGATGAGAAGAAGCCTTCAGAGGACTCTGGCTTTGACTCTGTCAGCCCCAGCACAG
AAGGCGTCTGCTCGGATTTGGATCAACTCTTTGTCTTCAATCAGAGAGCTATACTGCCCTGCTTT
GTAGTCATATACAGTGGCTATTAG

>JL3_GRMZM2G136494
ATGGTGGAACGGCCGGGCAGATCGGCAGGCGTCAGGGAGGCCAATAAGACTTCCGCTGCCGC
CATGGTGAAGACGACGACGACGACCAATAAGGAGGAGCTGGAGAGCCCGTCCTACAGGTTAGC
AGCGTTCAAGAGCCTCTTCTCGTGCAGGAACAGCCACGCGCGGCCGCGGGCGCCGGACACCG
GCACCGGCAGCAGGAGCAAGAGGCTGGGCTGCTCCGCGCCGTCCATCTGCAAGCTCAAGGAC
GACAGCGTGCCCGTGCCGCCCAAGGAAACCACGGGCACGGCGTCGGCAGGCGAGCCCTGCA
AGCGCCGCGCGTCCGTGAGCGGGAGCAGCGATCGGTGCGTGAAGAAGCCCCAATGTGGCGCT
GGCAGCAACAAGCAGCTGCAGCGTGGCGGGTCGTCGTCGTCGTCGGGCGGGTCGTCGTTCAG
GGCCGGCATGCAGCTGAGGCGGCTTTCGGGGTGCTACGAGTGCCACATGGTGGTGGCGGGCA
GCGCCTCCATGAGGGCCACCACCATCTGCCCCTGCCCGGACTGCGGCGAGGTGTTCGTCAGG
CAGGAGAGCCTCCAGCTCCACCAGTCCATCAGGCACGCAGTGTCCGAGCTTGGCACTGATGAC
ACGAGCCGCAACATCATCGAGATCATCTTCCAGTCCAGCTGGCTCAAGAAGCAGAGCCCCGTG
TGCAAGGTCGACCGCATCCTCAAGGTGAACAACACGCCCAGAACGCTGGCCCGCTTCGAGGAG
TACCGTGACGCTGTCAAGGCCAGGGCGGGCGCCGAGCAGCTGGCGGTAGCGGCGGCCAACA
GCAACAGGCACCCGCGCTGCACCGCCGACGGCAACGAGCTGCTCCGGTTCCACTGCGCCACC
CTGGCCTGCTCGCTCGGCCTCAACGGCGCCACGCACCTCTGCGACGCCGCGGGGACCTGCGC
AGCGTGCGGCATCATCCGCGACGGCTTCAGCAGCGGCGGGGTGCTGACCATGGCCACGAGCG
GCCGCGCGCACGACGCTGCGGAGGCTGGCGCCGGACTGGATGAGGAAACCACTCCAAGGGC
GATGCTGGTGTGCCGGGTCATCGCGGGAAGGGTGAAGCGGCCCAAAGAAGCGGTACCAGAGG
AGGAGGCCGTCGCCGACGCCGCCGAGGAGTCAGAGTACGACTCGGTTGCGGGGTCGGCCGG
GGTGTACTCCAACCTGGAGGAGCTGCTGGTGTTCAACCCCAGGGCCATCCTCCCATGCTTTGT
CGTTGTCTACAAGGCTTAG

>JL4_GRMZM2G045883
ATGGCGCTCGAAGCTGTGGTGTTCCCGAAGGAGCACCCGGGGTGTACGGCGGTCAAGGCGCG
CCCGGCGTCGCTGGGCTGCGGCTTCCACACCGAGGAGCTCGAGGTCGAGGACAAGGGCGGC
GTGGTTCTGCAAGGAGAGGCCGCCGACGCGCTGCCGCCGCCCGGTGGTGCCGCCACCACGT
GGGACTGGGACGCGGTGCTCTGCCCGTGCTCCACTGCACCAGCTGCAGGCGCCGTGGAGGAG
TGCTGGGACGTGCAGCAGCAGCAGCATCAGCTCCTGTCCGTGTCCCCGCCGCCCTCGTCCGC
CGTCGCAGCGTCGTCGTCTGGGCGCCGCAAGGCTGCCGCGTCGGAGGCGGCGCGGCGACGG
CGGCGACGCCCCAAGGCGGTGAGGAACACGGACGAGATGGAGAGCCAGCGCCGCAACCACAT
TGCCGTGGAGCGCAACCGGCGGCGGCAGATGAACGAGTACCTCTCCGTGCTCCGCTCCGCCA

TGCCGCCTTCCTACACGCAGCGGGGTGACCAGGCGTCGATCGTGGCTGGTGCCATCAACTTCG
TCAAGGAGCTGGAGCAGCTGCTGCAGTCGCTGGAGGCGCAGAAGCGGTGCACCGAGCCACCG
GCGCCGGCGCCGTTCGCCGGCTTCTTCATCTTCCCTCAGTACTCCACCACCGCCGCGACGGGT
GGTGCGGTTGGCTCTAGTAGCGACAGCGCTGGCTCCGGCGGCGATCAGAGTGGCGGCGGCG
GCGGCTGCGCGGGAGCGCGGCGAGGCGTGGCCGACATCGAGGTGTCCCTGGCGGAGAGCCA
CGCCAACGTGAAGGTGCTCGCGGCGCGGCGGCCGCGGCAGCTGCTGAGGATGGTGGTGGCG
CTGCAGTGCCTCGGCCTCACCGTGCTCCACCTCAACGTGACCACCACCGCCGACCACCTGGCC
TTGTACTCCCTCAGCCTCAAGATGGAGGACGAGTGCCGGCTGTCGCCGTCGGTCGACGACATC
GCCGCCGCGGTGAACGAGATACTCGCCGATATCTCCGACGAGTGCGCCCGCGGGGGGCCAGG
CCACTGA

>JL8_GRMZM2G131516
ATGCCACCTCCGCCACCACCACCCCCTCTCACACCATATTGCCGCCGCTGTCCTCCACCGCAC
CTGCCTCCGCCCCCGCCGTCGTCCCCAAATCACTTTTTGTTGCACTATCTTCATCAGTTGGATCA
TCAAGAAGCAGCTGCAGCAGCTATGGTTAGAAAGAGACCAGCGTCTGACATGGACTTGCCGCC
CCCCAGACGTCACGTGACTGGCGACCTCTCAGATGTTACTGCAGCTGCAGCTGCAGGAGTTGG
CGGCAGTGGCGCTCCCAGCTCCGCTAGCGCACAGCTGCCCGCTCTTCCGACTCAGCTGCACCA
ACTGCCTCCAGCGTTTCAACACCACGCCCCCGAGGTGGACGTGCCCGCGCATCCGGCCCCCG
CAGCCCATGCCCAAGCGGGAGGGGAAGCCACAGCGTCAACCACCGCTTGGGTGGACGGAATC
ATCCGTGACATAATCGGGTCCTCTGGAGGGGCCGCGGTGAGCATTACTCAATTGATCCACAATG
TGAGGGAGATAATCCACCCCTGCAACCCTGGACTTGCAAGCCTCCTCGAGCTGCGCCTTAGGA
GCCTGCTGGCAGCTGACCCCGCACCTTTGCCACCTCCCCCTCAGCCCCAGCAACACGCCCTGC
TGCACGGGGCGCCAGCTGCAGCTCCGGCGGGCTTGACTCTTCCACCACCACCCCCACTGCCG
GATAAGAGGAGGCATGAGCACCCGCCTCCATGCCAGCAGCAGCAGCAAGAGGAGCCTCACCC
CGCACCCCAATCGCCAAAAGCTCCAACGGCGGAGGAAACAGCAGCAGCTGCTGCTGCTGCTCA
AGCAGCAGCAGCTGCTGCTGCTAAGGAGAGAAAGGAGGAGCAGCGCCGGAAACAAAGGGATG
AGGAAGGCCTGCACCTCCTCACCCTGTTGCTCCAATGCGCTGAGGCTGTCAACGCTGATAATCT
GGATGACGCACACCAGACCCTCCTTGAGATTGCTGAACTGGCGACTCCATTTGGCACGAGCAC
ACAGCGTGTCGCAGCGTACTTCGCCGAAGCTATGAGCGCCAGACTCGTTAGCAGCTGCCTCGG
CCTTTACGCTCCGCTCCCACCTGGTTCCCCCGCGGCCGCAAGATTGCATGGTCGCGTTGCTGC
AGCTTTTCAAGTGTTCAATGGTATCAGCCCTTTTGTGAAGTTTTCACACTTCACAGCGAACCAGG
CCATCCAAGAGGCGTTCGAGCGCGAGGAGAGAGTCCATATTATTGACCTGGACATTATGCAGG
GTCTCCAGTGGCCGGGCCTTTTTCACATCCTCGCTTCAAGGCCCGGAGGTCCTCCTCGGGTCC
GCTTGACAGGCCTGGGGGCCTCAATGGAGGCCCTGGAAGCGACGGGCAAAAGGCTTTCTGAC
TTCGCGGACACGCTTGGGTTGCCGTTCGAATTCTGCGCTGTCGCGGAAAAGGCAGGTAACGTC
GATCCGGAAAAACTGGGTGTCACCAGGCGTGAAGCAGTCGCTGTGCACTGGCTCCATCACAGC
CTCTACGATGTGACCGGTTCAGATTCAAATACCCTCTGGCTTATTCAGCGCCTGGCACCGAAGG
TCGTCACAATGGTGGAACAGGACTTGAGTCATAGTGGCTCTTTTTTGGCGCGTTTCGTGGAAGC
GATACATTACTATTCGGCCCTGTTCGACAGCCTTGACGCCTCGTACGGTGAGGATTCCCCCGAG
AGACACGTCGTTGAGCAACAACTGCTTTCAAGAGAAATTAGGAACGTCCTGGCCGTGGGAGGC
CCTGCCAGGACAGGCGACGTCAAGTTCGGCTCCTGGAGAGAGAAATTGGCCCAATCGGGCTTT
CGTGCTGCATCACTGGCAGGGAGCGCGGCAGCACAGGCCAGTCTGCTCTTGGGCATGTTCCC
GTCGGATGGCTACACCCTCGTGGAAGAAAACGGCGCATTGAAGTTGGGCTGGAAGGACCTCTG
TTTGCTCACCGCCAGCGCGTGGCGGCCGATCCAGGTGCCACCGTGCAGGTGA

>JL13_AC215201.3_FG008
ATGGTTTCCAAGGAGCAGAATAAGCGAGGAGCTTTGCATGAGAAGCTCAAGATTCTCCGCAGCG
TTACTCACTCTCATGCGGTAACTGTCGTCATCTCCTTTTCTCTTTCACATGTTGTTCATTCTTCTT
CTTGATGATTACTCATCCATCGACCTCTTGTTATGTACGTGAACACGAATTCTCGTTTTCTCTCTC
TCTCTCTCTCTCTCTCTGAAAGAACACTCATCGTGCTCTAGCTAGTTATGTAGGTGAAAGAATCT
CAAGCTAATAATAGCTAGTGTGTCATGCACGGTTAACTGTTTTTCCTTCAGTTGTTGTTTTCTTTC
ACGTATGAGGCAATGATCGTCGTTTACATTCATCATTTCATCAGTTTGTTTATCCACACAGCCATT
CATGCCTTGCGTATGTTTTCTCTGAAAGAACAGATGCTTTCGTACCAATATTATTCACATATCAGT
CATGCTGTGTGTGTATATCTACGTACCATTCACCTTTCAGACCATTCCGATGCTGTCAAGTTATAT
GCAACACGTACGTGTGTGTCACGCAGGGGGATAAAGTGTCGATAATAGCGGACGCGTCATCGT
ACATCAAGGAACTAAAGCAGAAGATCGCGAAGCTAAGCCAAGAGATGGCATCGTCGTCTCCGC
AGCACGCCACTACTGGTGTCTGCCAACAACAACCGTCGTCGTCGTCGGTGAGTGTGGGAGTCC
TGCTGGACAAGAAGGGCCGTTTCCTCGTCAGTGTGTTCATGGACGAGAGCTGCGGCCCGCCGC

CGGCCGGGCTACTCGCTTCCGTCCTCGAGGCCTTCGACGACATCGGACTCACGGTGCTCGAG
GCTAGGGCTACCTGCGCTGGTTCCTTTCGGCTAGAAGCTGTAGGAGAGGTGCGATGCGACTGC
GAACGAGTACACATTATTACACCGCGCGTACCGCCGCAAACGCGGTAGTACGCATGCATGATTT
AATCACTTCTTTGTCTTGCTTCTTTAATCTCTCTTGGTTTATTTGCTGCTTTGTTTGTTGTCATTCC
AGGAAGTAGTAGTAGATGGGGGCCTCATCATCGACGCGCACGCGGTGGAGCAAGCAGTAGTTC
AGGCAATCAAGAATTGCCCTGCAAATTAA

>JL15_GRMZM2G016477
ATGCCGCTGTCGCGGTCGCTGTTCCTGCTGCTCATGCTCGCGCTGGCGCCGCTTGCGTCCCTG
GCCGCCCCGGCGCCGGCGCCGCCGGTGGCGAGGACGGCCGGGGTGCAGGCGGAGATCGAC
GCGCTCCTCGCGTTCCGCCGCGGCCTGCGCGACCCCTACGGCGCCATGTCCGGATGGGACGC
GGCCTCCCCCTCCGCGCCCTGCTCCTGGCGCGGCGTCGCGTGCGCGCAGGGCGGCGCCGGC
GGGCGCGTCGTCGAGCTGCAGCTCCCGCGGCTCCGCCTCTCGGGCCCCATCTCGCCGGCGCT
CGGCTCGCTGCCGTGCCTCGAGCGGCTCGGCCTCCGGTCCAACGACCTCTCCGGCGCCATCC
CGGCGTCGCTGGCTCGCGTCACCTCCCTGCGTGCCGTCTTCCTCCAGTCCAACTCGCTCTCCG
GACCCATTCCCCCGTCGTTCCTCGCCAACCTCACCAACCTCGACACTTTCGACGTGTCCGGCAA
CCTCCTGTCCGGCCCCGTCCCCGTCTCATTTCCTCCCGGCTTGAAGTACCTCGACCTCTCCTCC
AATGCGTTCTCCGGAACCATCCCGGCGAACATTGGCGCATCCATGGCGAACCTCCAGTTCTTGA
ACCTGTCCTTTAACCGGCTCCGGGGTACCGTGCCGGCGTCCCTGGGCAACTTGCAGAACCTGC
ATTACCTCTGGCTGGACGGGAACCTTCTCGAGGGGACCATCCCGGCGGCGCTGGCCAACTGCT
CGGCGCTGCTCCACCTGAGCCTTCAAGGGAACTCGCTCCGCGGCATCCTGCCGTCTGCGGTAG
CCGCGATACCCACGCTGCAGATTCTCTCGGTGTCGCGGAACCAGCTCACCGGTACCATCCCGG
CCGAGGCGTTCGGCGGCCAGGGGAACTCGTCTCTGCGCATCGTGCAGCTCGGCCGCAACGAG
TTCTCTCAGGTGGACGTGCCGGGAGGTCTCGCCGCGGATCTTCGGGTGGTGGACCTTGGCGG
CAACAAGCTCGCTGGTCCGTTCCCGACTTGGATTGCTGGGGCGGGAGGGCTGACGCTGCTCG
ACCTCTCGGGCAATGCGTTCACAGGCGAGTTGCCGCCAGCGGTCGGGCAGCTCAGTGCGCTG
CTTGAGCTGCGCCTCGGCGGCAATGCTTTCGCTGGCGCCGTGCCTGCGGAGATTGGCAGATG
CAGCGCACTCCAGGTGCTTGATCTCGAGGACAACCACTTCACCGGCGAGGTGCCATCAGCTCT
AGGTGGTCTCCCGAGGCTCAGGGAGGTCTATCTTGGTGGGAACACTTTCTCCGGCCAGATTCC
GGCGACCTTGGGGAATCTGGCGTGGCTAGAGGCATTGTCCATACCCAGGAACAGACTCACTGG
TCGCCTTTCTCGTGAGCTCTTCCAGCTGGGAAACCTGACGTTCCTGGACCTATCCGAGAACAAC
CTCACCGGAGAGATCCCTCCGGCCGTCGGCAATTTGTTGGCTCTCCACAGTTTAAATTTGAGCG
GCAATGCCCTTTTTGGCCGCATTCCAACGACCATTGGCAACCTCCAGAACCTGCGAGTTCTTGA
TCTCTCCGGGCAGAAGAACCTCTCTGGCAATGTCCCGGCGGAGCTTTTCGGCCTACCGCAGCT
GCAGTACGTGTCGTTCTCGGACAACTCCTTCTCTGGCGATGTTCCTGAAGGTTTCAGCAGTCTC
TGGAGTCTGCGCAATCTCAATCTCTCCGGCAATTCTTTCACTGGGTCAATACCGGCGACGTATG
GGTACTTGCCGTCACTTCAAGTGTTGTCAGCCGCACACAACCATATTTCTGGCGAGCTACCAGC
CGAGCTCGCCAATTGTTCCAACCTCACTGTGCTTGAGCTCAGTGGCAACCAGCTGACCGGCTC
CATCCCGAGGGACATCTCACGCCTTGGTGAATTGGAGGAGCTTGACCTCAGCTACAATCAGCTT
TCAGGCAAGATACCACCAGAGATCTCCAACTGCTCATCACTCACCCTTCTCAAGCTTGACGACA
ATCATTTTGGCGGAGACATACCAGCCTCTGTTGCCAGCCTCTCGAAGCTACAGACGCTTGACCT
GTCGTCCAACAATCTTACCGGCAGCATCCCTGCTTCATTGGCTCAGATTCCTGGCCTTTTATCAT
TCAACGTGTCACACAACAAGCTTTCCGGCGAGATACCGGCGATGCTCGGCTCCCGCTTTGGCT
CTTCGTCAGCATATGCTTCGAACTCAGACTTGTGCGGTCCGCCGTCGGAGAGCGAGTGCGGCG
TGTACCGGCGACGGCGGAGGCGGCAGAGGGTGCAGCGCCTGGCTCTGCTTATCGGTGTGGTG
GCTGCTGCGGCGTTGCTCGTTGCGCTGTTCTGCTGTTGCTGCGTGTTCAGCTTGATGGGATGG
AGGCGTCGGTTCGTTGAGAGCCGTGACGGTGTCAAGAAGAGAAGACGCAGCCCAGGACGCGG
CAGCGGGTCGAGCGGCACGAGCACGGAGAACGGCGTCAGCCAGCCGAAGCTGATCATGTTCA
ATTCAAGGATCACTTACGCTGACACGGTTGAGGCGACCCACCAGTTCGACGAGGAGAACGTGC
TCAGCCGAGGCCGCCACGGCCTCGTGTTCAAGGCTTGTTACAGTGACGGCACCGTGCTGGCGA
TTCTGCGGCTCCCGTCCAGGTCTGCCGATGGCGCCGTGGTGATCGACGAGGGATCGTTCAGGA
AGGAGGCCGAGTCTCTCGGGAAGGTGAAGCACAGGAACCTCACGGTGCTCCGTGGCTACTATG
CCGGGCCTCCGCCGGACGTCCGGCTGCTGGTCTATGACTACATGCCCAACGGCAACCTCGCCA
CGCTGCTGCAGGAAGCGTCACACCGGGACGGCCACATTCTCAACTGGCCGATGAGGCACCTCA
TCGCGCTCGGCGTCTCGCGCGGTCTTGCGTTCCTGCACCAGTCCGGGGTGGTGCACGGCGAC
GTCAAGCCGCAGAACATTCTCTTCGACGCCGACTTCGAGCCGCACCTGTCTGATTTCGGCCTCG
AGCCGATGGTGGTGACTGCGGCCGCCGCCGCCGCGTCGACATCGGCTGCAACTGCAACACCA
CCCGTGGGCTCCCTCGGCTACGTGGCCCCCGACGCGGCCGCTGCCGGGCAGGCCACGAGGG

AGGGCGACGTGTACAGCTTCGGCATCGTGCTGCTGGAGCTCCTCACGGGTCGGCGCCCCGGC
ATGTTCGCCGGGGAGAAGGAGGAGGAGGAGGAAGAGGACATCGTGAAGTGGGTGAAGCGGCA
GCTGCAGCGCGGCGCCGTGGCGGAACTGCTGGAGCCGGGCCTGCTGGAGCTGGACCCGGAG
TCGTCCGAGTGGGAGGAGTTCCTGCTGGGCATCAAGGTCGGGCTGCTGTGCACCGCGTCCGA
CCCGCTGGACCGCCCGGCCATGGGCGACGTGGTGTTCATGCTGGAGGGCTGCCGCGTCGGCC
CGGACATCCCGTCCCCGTCCTCCGCCGACCCCACCTCCCAGCCGTCGCCGGCCTGA

>JL22_GRMZM2G480386_CDS
ATGCAGGGGCAAGTTGCTGGTGACGACGCAGGTGGTGCAGGCAGCCCTAGCAGGCGGCGGGA
GAGCTCGGCCGCCGCGCGCTTGGTGCGTGGCCCCATCATCGTCGGCGCGGGGCCGTCCGGG
CTGGCCGTGGCGGCGACGCTGAGCCGGCACGCGGTGCCCTTCACGGTGCTGGAGCGTTCCGA
CGGCATCGCCGACCTGTGGACGAACCGCACGTACGACCGGCTCCGGCTGCACCTCCCCAAGG
TGTTCTGCGAGCTCCCGCACGCCCGCTTCCCGGCCGACTTCCCCACGTACCCGACGAAGCACG
ACTTCCTCCGGTACCTCCGCTCCTACGCCGCGCGCTTCGCCGTGGCCCCGCTGTTCGGGCGCA
CGGTGACCTGCGCGCGGTACGACGCGGAGGCGTCGCTCTGGAGGGTCACGGCGGTGTCGTC
GTCGTCGACTGCCGCGGGCGGCGGCGCGGCGGCGGAGACGACGACTGAGACCGAGTACGTG
TCGCCGTGGCTGGTGGTGGCCAGCGGGGAGAACGCGGAGGTGGTGGTGCCCACGGTGAAGG
GGAGGGAGAGGTTCGGCGGGGAGGTGCTGCACTCCAGCACGTACAGGAGCGGGGAGAGGTT
CAAGGGCATGAGGGTCCTGGTCGTGGGCTGTGGCAACTCCGGCATGGAGATGTGCTTGGACTT
GTGCGAGCACGGTGCCATGCCCTTCATGTCCGTCAGGAGCGGGGTGCACGTTCTGCCGAGAG
AGATGTTCGGGGCCTCAACGTTCGGCATCGCCATGAAACTGCTCCGATGGCTGCCGATCAAGA
TGGTGGACAGGCTCCTGCTCCTGGTGGCGAGGATGGTCCTGGGTGACACCGAGAAGCACGGG
CTGAGGAGGCCCAAGCTAGGCCCGCTGGAGATCAAGAACGTCACCGGCAAGAGCCCTGTTCTG
GACGTGGGAGCATGGTCGTTCATCAAATCTGGAAACATCAAGATCGTGCCAGAAGTGGAGTCAT
TCACCGGCAGCAACGGGGTGAGGTTCGTGAACGGCGACGAGATGGCCTTCGACTCTGTCATCT
TCGCCACCGGCTACAGAAGCAACGTCCCTTGCTGGCTCAAGGATGACGGCGAGCTGTTCACCG
AGGACGGAAAGGCGAAAGCTGAGCAGCCGAGCGACGACGACAGGTGGAGGGGTCCCAACGG
GCTGTACCGCGTGGGGTTCTCCGGCCAGGGCTTGCTCGGCGCTGGCGCGGACGCGCTGAGG
GCCGCCGCCGACATCGCCGGGAGGTGGCAGGCGGAGGTGGCAGCAGCGGTGGCTGGAGCCA
AGGTTTCCTCGGCCTCGGTGTAG

>JL22_GRMZM2G480386_GENOMIC
ATGCAGGGGCAAGTTGCTGGTGACGACGCAGGTGGTGCAGGCAGCCCTAGCAGGCGGCGGGA
GAGCTCGGCCGCCGCGCGCTTGGTGCGTGGCCCCATCATCGTCGGCGCGGGGCCGTCCGGG
CTGGCCGTGGCGGCGACGCTGAGCCGGCACGCGGTGCCCTTCACGGTGCTGGAGCGTTCCGA
CGGCATCGCCGACCTGTGGACGAACCGCACGTACGACCGGCTCCGGCTGCACCTCCCCAAGG
TGTTCTGCGAGCTCCCGCACGCCCGCTTCCCGGCCGACTTCCCCACGTACCCGACGAAGCACG
ACTTCCTCCGGTACCTCCGCTCCTACGCCGCGCGCTTCGCCGTGGCCCCGCTGTTCGGGCGCA
CGGTGACCTGCGCGCGGTACGACGCGGAGGCGTCGCTCTGGAGGGTCACGGCGGTGTCGTC
GTCGTCGACTGCCGCGGGCGGCGGCGCGGCGGCGGAGACGACGACTGAGACCGAGTACGTG
TCGCCGTGGCTGGTGGTGGCCAGCGGGGAGAACGCGGAGGTGGTGGTGCCCACGGTGAAGG
GGAGGGAGAGGTTCGGCGGGGAGGTGCTGCACTCCAGCACGTACAGGAGCGGGGAGAGGTT
CAAGGGCATGAGGGTCCTGGTCGTGGGCTGTGGCAACTCCGGCATGGAGATGTGCTTGGACTT
GTGCGAGCACGGTGCCATGCCCTTCATGTCCGTCAGGAGCGGGGTAAGTTACCAGCAGCTAAG
CTAGCCCTCTTTTTCCATCTTCTTCCTTCCTATATATATTAATCCTCTAGCTACTAGCTGCTTAATT
ATATATCAGCTTGCTATATACATAATACTGTTGGACACACTTGTTGATCCGAGTTTCATGTACTCC
GACATACATTGTTGCCATTTATCTTACTTGATCACGTCATATCTCTAGTCGCTATCTATTTTTAAGA
AATTTAAAAAATGCAGTAACCTTTCAGTTTTCTAGCTATATAGAACTGTGATATTTATATATAATAT
GTTGCATTGCTAGTCATTAATATATAATTCCATGAAGAAGTGAATAGGTTCTATATATGTGTGTGT
GTATGGCTTTTATTACTACTGTGTACTGGAGCTTAATCATATATGGACCAATAATGCGCATCGTAT
GTCGTGCTTATGAAACGAAACAGGTGCACGTTCTGCCGAGAGAGATGTTCGGGGCCTCAACGT
TCGGCATCGCCATGAAACTGCTCCGATGGCTGCCGATCAAGATGGTGGACAGGCTCCTGCTCC
TGGTGGCGAGGATGGTCCTGGGTGACACCGAGAAGCACGGGCTGAGGAGGCCCAAGCTAGGC
CCGCTGGAGATCAAGAACGTCACCGGCAAGAGCCCTGTTCTGGACGTGGGAGCATGGTCGTTC
ATCAAATCTGGAAACATCAAGGTGAGGGCTGCTGTCAAAGGGTCACACTGAAATAAAGAAGAGT
GACGTCGTTCGTGCATGGGAATGGAGGAGTGTACTAGGAGTGCATCCATCCATGCATCTTTCAT
TCATTCCTTCCTGTTGCTTCCTTTCGCATGTCATGCCAACGCTAGCTTTCACTTTTGAGGTTCATA
CAACATTCTTTTCCCTATCCCTTTGTTGTTGGTGGTAGTGTGTGCGTGGGCTGATGAGTGTGAAA

ACTACTAGTTTTTACAGTTTTATCCGCATGCATGCATGCCTCTTTGCATGGACCATGGCTTTATTA
TTATATATTTTTTACACCCTCCCCGTCCCCGGTCTCTTCTTGCTCTACTAGCTAGGGTGCCTGCC
TGGTGCATGCAGATGCAGGCGTCTGTACTTGGGCGTATAGCATCATGCTTGCATGACAACAAAA
TAACTGCTGGCTGAACAAAAATGGCATCTCCATGGACCATTGTCCGATGCCCTTATTTGCGTGCT
GATGATGGCTAAGCTAGCAGCTAACTTTGTGGCAAAATGTGCAATCATGTGATGGTGGTCCGAT
GTATATATATTCCATTTCACCTAATTTCTGCGTTCAAACATGCATTTTATTTGTCCATACACATAAC
ATATTTATGTGTGCACTTGTGATGATGCAGATCGTGCCAGAAGTGGAGTCATTCACCGGCAGCA
ACGGGGTGAGGTTCGTGAACGGCGACGAGATGGCCTTCGACTCTGTCATCTTCGCCACCGGCT
ACAGAAGCAACGTCCCTTGCTGGCTCAAGGCACGTACGTCGTCGCCGGCCGCGCGCCGCGCC
TATAGCTAGCTAACTGATCGACACTCAGCTCCTGTTACTGATCGGTACTACTACTACCTACGTGG
TGGGCGTGCACATGTATGCAGGATGACGGCGAGCTGTTCACCGAGGACGGAAAGGCGAAAGC
TGAGCAGCCGAGCGACGACGACAGGTGGAGGGGTCCCAACGGGCTGTACCGCGTGGGGTTCT
CCGGCCAGGGCTTGCTCGGCGCTGGCGCGGACGCGCTGAGGGCCGCCGCCGACATCGCCGG
GAGGTGGCAGGCGGAGGTGGCAGCAGCGGTGGCTGGAGCCAAGGTTTCCTCGGCCTCGGTGT
AG

>JL23_GRMZM2G069365
ATGGAGTACAAGAGATCATCGCATGTGGAGGATGAAGACGAGGATGAGCTGGAGGAGGAGGA
GGAAGAAGACGAGGAAGAGGAGGAGGACGAGGATGAGGAGGCCAGAAGCCATCGCTACACCA
CGGCGGCGGCGGCACCCTTAGGCACTCCACAGCAGCAGGAGGTGCAAGCCCAAGCTCTTGGG
CTCGGCTCGCACGCCTCCCTGATGGACTCCGCCGCCTTCTCGAGGCCGCTCTTGCCTCCCAAC
TCATCTCTGGTGACGCAGCCACCGCTGCCTCCACCAGGCTTCCTGCCGGCTCACCGCCAGCCG
CCGCAGCTCCATCCGAGGAAAGCGGAGAGGGAGAGGGAGAGGGACAGAGCCGCGGGAGCGC
AGCAGCCCCAGCTCCGGCGCCACCAAGAACCAGTGAGGAATAATAATGGCGTTCTCGGCGGCG
GCGGTGGCGCCGCGCCCCCATCGGCGGCCTCTTCTACGTTCGGCCTCGCGTCCGTGCGGCCC
GTGGTGGAGGCACCCCAGTGGCGGTACCGTGAGTGCCTGCGCAACCACGCGGCGCGGCTTGG
CGCGCACGTGCTCGACGGCTGCTGCGAGTTCATGCCGTCCGGGAGCGACGGGGCCGCCGCG
CTGGCCTGCGCCGCCTGCGGCTGCCACCGCAGCTTCCACCGACGCGAGGCGATCCCCGGCG
GCGTTGCCGCCGCGGCGGCGGTGGTATCGCCCTCGGCGGTGCCGGTGGTGACCCCCACAGC
CCGCGCCAGCGCCAACTCGTCCCGTGTCATGCCGCTGCTCATGGCCCCGCCGCACATGCATAC
GCGCCCGCCGCACGTCCCGGCGTCCCCGGCGTCCGCGCCCGCCGCGCTGACCGAGTCGTCG
AGCGAGGAGCTGCGAGGCCACGCGCCCGCGCACCCGGCCGCGCCGCCGCCGCCGCCGCCAC
ACGCGCAGGTGGTGGCCCTAGCGGGGTCGGCTTCCGCTCCCCCGGCGCCGAGCAAGAAGCGT
TTCCGGACCAAGTTCACGGCGGAGCAGAAGGACCGCATGCGCGAGTTCGCGCACCGCGTCGG
CTGGCGCATCCACAAGCCCGACGCCGACGCCGTGGACGTCTTCTGCGCCCAGGTCGGCGTCT
CCCGGCGCGTCCTCAAGGTGTGGATGCACAACAACAAGCACCTCGCCAAGGTCCCGCCCTCGC
CCCCATCCTCCCAGGCCCAGGCCCAGGCCCAGCCCCCGCCGCCGCCGCAGCAGCATCGCCAC
CACAACCACCACCCACCTCCACCTCCGCCGCACCACATCCACCATCACCACCACCCGCCGCCG
CCGCCGCAGCAGGAGCAGCAGCATGATATGCATGAGTGA

>JL24_GRMZM2G417229
ATGGAGTACAAGAGATCATCGCATGCGCAAGAGGAAGAAGAGGACGAGGAGGAGGAGGAAGA
AGAAGACGACGAGAACGAGGAAGAAGAGGAGGATGAGGAGGAGGCCAGAGGCCTTCGCTACA
CCACGGCGGCGGTGGCACCCGTAGGCGCTCCACAGCAGCAGCAGGTGCACGCCCAAGCTCTT
GGCCTCGGCTGCTCACACCCCTCCCAGCTGATGGATTCTGCCGCCTTCTCGAGGCCGCTCTTG
CCTCCCAACTCATCTCTGGTGATGCAGCCACCGCTGCCTCCACCAGGCTTCCCGCCGGCTCAC
CGCCAACAACCGCCGCATGCGCAGCTCCATCCGAGGAAAGCGGAGAGGGAGAGGGACAGAGC
CGCGGGAGCGCAGCCCCATGTCCGGCGCCACCACCAAGAACCAGCCAAGAATGGCGTTCTCG
GCGGTGGCGCCGCGCCAACGTCGGCACCCTCTACGCTCGGCCTCGTGCCCGTGGTGGAAGCG
GCCCAGCTGTGGAGGTACCGGGAGTGCCTGCGGAACCACGCGGCGCGGCTTGGCGCGCACG
TGCTCGACGGCTGCTGCGAGTTCATGCCGTCCGGGGGCGACGGGGCCGCCGCGCTGGCCTG
CGCCGCCTGCGGCTGCCACCGCAGCTTCCACAGACGCGAGGCGGTCCCCGGCGGCGGCGTG
GCGGTATCGCCCTCGGCGGCGGTAACTCCCACCCCCACCGCCGGCGCCAACTCCTCCCGCGC
CATTCCGCTGCTGCTGGGCCCCGCCGCACATGCACACGCGCCCGCAGCACCACGTGCCGGCG
TCCCCGGGTGTCCGCGCCCGCCGCGCTGGCCGAGTCGTCCAGCGAGGAGCTGCGCGGCCCC
GCGCCCGCGCCCGCGCCGCCGACGCACGCGCAGGTGGCCGTAG

>JL25_GRMZM2G425236

ATGGATCAACAGCAGCACCAGGAGCGGCCGTGGGAGGTGTACCGGGAGTGCATGCGCAACCA
CGCGGCGAAGCTCGGGACATACGCCTCCGACGGATGCTGCGAGTACACCCCCGACGACGGCC
AGCCCGCAGCGATGCTCTGCGCCGCCTGCGGCTGCCACCGCAACTTCCACCGGAAGGCGTTC
CTCGCCGACGCTGCGGCGGGAGCGCACGGCGGCGCGCACGGGGCAATGCTGCCCTCTCCCG
GCGCGTCCCCGGGCTACGGCATGCACCACATGGCGATTGCTGCGGCCGGCATGGGCGGTGAC
GCCGGCGCGCACGGCTCCGGGTCCGGGGGCAGGCGCCGGACGCGGACCAAGTTCACGGACG
AGCAGAAGGAGCGCATGGCGCGGTTGGCGGAGCGGCTCGGCTGGCGGATGCCCAAGCGGGA
GCCCGGCCGCGCGCTGGGGGACGACGAGGTGGGCCGCTTCTGCCGGGAGATCGGGGTCACC
AGGCAGGTCTTCAAGGTATGGATGCACAACCACAAGTCCGGTGGTGGCGGCAGCCAGGGCGA
GGGCGCCAGCGCTGGCGCTGCCGGAGCCGCTCAGACGACGTCGTCGTCCACGACGCGTGGC
GGCGGCGGCGGCGGCGTTGTTGGAGGAGGCGGGAGCATGTCCACGGCGATGATGGGCGCCG
ACGTCGAGGACGACGAGGAGGTGAGGGGCAGCGAGATGTGCATGTAG

>JL26_GRMZM2G119359
ATGCACCGTGGCCGCAACCGTTCAAGAAAGCCTGTGGAATCCAAGACCGCCTCCTCGCCGCCC
CAGCTGTCCACCGTCGTCACCACCACCACCACCCGGGAGGCCGCCGCCGCGACGCCCCTTGA
GTCCCTCGCGGGGGCGGGGGGTAAGGCTCACGGCCTGTCCCTCGGCGGCGGGGCTGGCTCG
TCGCACCTCAGCGTCGACGCTTCGAACGCTCACTTTCGCTATGGCAGCAAGTATGGTTACATTC
GCAGTTCCCTTTTTTGTGGTTCTAGCTGTTGTTGCTATCGAATGGCCATGATGCTATAGTTTTCG
TTGGTTCTACACTATTATCGCAAAAGGAGAATCACACCGACTCACTCACTCTTCAACGGCAAATG
CACTTCTGCCACTGGCTTCTTGCAGTATTAGCCCTTCTACTGTACAATATAGAAATCTTTTGCAGT
TCTGTTGTATTAAGTGAACTGCCGTGCCTGGTATGCCCTGGGCCCTGGCTGCTTCTGCTATCTT
AGATGTCTATAAAATTTTATATATTTGCATGCTCTGAACAACTTGGGCTGGCGGATGCCCATGTC
CATGCCGCTACACAACTTAGGTCTGTACTAGTGTTTCAAACAGATTTTCTTATTGGCCCCCTATTT
TGTACTCCGTTTCTTTCGGATTCATTGTTTTTGTGGGTTTGAATTTCTATAAGGATTTGCGCATCA
AATATTGATATGACTTCAAGACAATATTGCAGTTCATGTCCTGAATTTGCATCCTGAGCTTGATTT
TCTGTCGAGTTGACTTTAATAGCTATTTATTTGTCACTTGTACATCTATTCCACTTCATACCAAACA
AACCCACCATGTCTTGCCGGTTCCTGTAGGCAGAAATATGACACTAGATAGACTCCAAAGCTATA
GTGCCAGGCTGACAAGTGTTCTGTCAGTTCATAATACTTTCGTTGGCAACTTGTGATTGTCACAT
ACTATCCATAACTAATTGACTGTGCAGGTACCCTCTTGGAGCTAAATCCGATGCTGGCGAGCTG
AGCTTCTTCTCAGGAGCACCAGGGAACTCCAGGGGCTTCACCATTGATTCTCCAGCAGATAACT
CTTGGCACTCCCTGCCATCCAACGTGCCCCCGTTTACACTGTCCAAGGGCAGAGACTCTGGCC
TCCTGCCCGGTGCCTACTCCTACTCCCACATTGAACCACCGCAGGTGCTCGGCCAGGTCACCA
TCGCCTCGCTGTCACAGGACCAGGAGCGCCACCAGTCGTCGTTCAGCAGCGGCGGGGCCGGC
GCTGGTGGATTGCTGGGGAGCGTGAAGCAGGAGAACCAGCCGCTGAGGCCCTTCTTCGACGA
GTGGCCTGGGACGCGGGACTCGTGGTCGGAGATGGACGACGCGAGGTCCAGTAGGACCTCCT
TCTCGACGACCCAGCTCTCCATCTCCATTCCGATGCCCAGATGTGAGTTGTTTCATACTGATCTC
ATCTTAGGAACCCGCAGTCCTTCTGTGTGCTTCCCCAATCGTGCTTGGATGTGTTCTGATGACA
GCAGCAATCACAATTGTTTCTGCAGGTGATTGA

>JL27_GRMZM5G893117_CDS
ATGATGATGATGAGCGGTCGAGCGGCCACCGCGGGGCGGTACCCGTTCACGGCGTCGCAGTG
GCAGGAGCTGGAGCACCAGGCGCTCATCTACAAGTGCCTGGCGTCCGGCAAGCCCATCCCGT
CCTACCTCATGCCACCGCTCCGCCGCATCCTCGACTCCGCCCTCGCCACGTCGCCGTCGCTCG
CCGCCTTCCCGCCGCAACCCTCGCTGGGGTGGGGGGGCTGCTTCGGGATGGGCTTCAGCAGG
AAGCCCGCCGACGAGGACCCGGAGCCCGGGCGGTGCCGGCGCACGGACGGCAAGAAGGCGC
TGCTCCAAGGAGGCGTACCCGGACTCCAAGTACTGCGAGAAGCACATGCACCGGGGCAAGAAC
CGTTCAAGAAAGCCTGTGGAAATGTCCTTGGCCACGCCGGCGCCGGCCTCCTCCGCCACAAGC
GCCGCCGCCGCCGCCACCTCCTCGTCCCAGGCGCCGTCCTACCACAGCCCGGCCCCCGCCGT
GCCGTACCACGCGCCCTACGGCGCCGCGTACCATCACACGCAGACGCAGGTGACGATGAGCC
CCTTCCACCTCCTCCACCTCGAGACCACCCACCCGCACCCGCCGCCGCCGCCGCCGCCGCCC
TACTACTACGCGGACCAGAGGGACTACGCCTACGGCAAGGAGGTCGGCGAGCGCGCCTTCTTC
TCCGACGGCGCGGGCGAGAGGGACCGGCAGCAGCAGGCCGCGGGGCAGTGGCAGTTCAAGC
AGCTCGGGACGATGGAGGCGACGAAGCAGCCGTGCACCACGCCGCTGCTCGTCCCCGCCGCC
GGGTACGGCCACGGCGCGGCGTCGCCGTACGGCGTCGGTCAGGCCAAGGAAGACGAGGAGG
AGGAGGAAACGCGGCGGCAGCAGCAGCACTGCTTCGTTCTTGGCGCCGACCTGCGGCTGGCG
GAGCGGCCGTCGGGGGCACATGA

>JL27_GRMZM5G893117_GENOMIC
ATGATGATGATGAGCGGTCGAGCGGCCACCGCGGGGCGGTACCCGTTCACGGCGTCGCAGTG
GCAGGAGCTGGAGCACCAGGCGCTCATCTACAAGTGCCTGGCGTCCGGCAAGCCCATCCCGT
CCTACCTCATGCCACCGCTCCGCCGCATCCTCGACTCCGCCCTCGCCACGTCGCCGTCGCTCG
CCGCCTTCCCGCCGCAACCCTCGCGTACGCGACTACTGTCTCTCTAATCTATGCGGCCGCCAT
GTGTCTTTATTTCCTGTATATTCTGGTTCACGTCCGCGTTGTTGGTTGCCTTTGCATGCAGTGGG
GTGGGGGGGCTGCTTCGGGATGGGCTTCAGCAGGAAGCCCGCCGACGAGGACCCGGAGCCC
GGGCGGTGCCGGCGCACGGACGGCAAGAAGGCGCTGCTCCAAGGAGGCGTACCCGGACTCC
AAGTACTGCGAGAAGCACATGCACCGGGGCAAGAACCGTTCAAGAAAGCCTGTGGAAATGTCC
TTGGCCACGCCGGCGCCGGCCTCCTCCGCCACAAGCGCCGCCGCCGCCGCCACCTCCTCGTC
CCAGGCGCCGTCCTACCACAGCCCGGCCCCCGCCGTGCCGTACCACGCGCCCTACGGCGCCG
CGTACCATCACACGCAGACGCAGGTGACGATGAGCCCCTTCCACCTCCTCCACCTCGAGACCA
CCCACCCGCACCCGCCGCCGCCGCCGCCGCCGCCCTACTACTACGCGGACCAGAGGGACTAC
GCCTACGGCAAGGAGGTCGGCGAGCGCGCCTTCTTCTCCGACGGCGCGGGCGAGAGGGACC
GGCAGCAGCAGGCCGCGGGGCAGTGGCAGTTCAAGCAGCTCGGGACGATGGAGGCGACGAA
GCAGCCGTGCACCACGCCGCTGCTCGTCCCCGCCGCCGGGTACGGCCACGGCGCGGCGTCG
CCGTACGGCGTCGGTCAGGCCAAGGAAGACGAGGAGGAGGAGGAAACGCGGCGGCAGCAGC
AGCACTGCTTCGTTCTTGGCGCCGACCTGCGGCTGGCGGAGCGGCCGTCGGGGGCACATGA

>JL28_GRMZM2G114893
ATGGCGACGGCGTGGGTGCGCTCGCTCAGCTGCAGGTCCTCCTACGCCGTCACGGACGCCGC
CATCGCGCCGTCACCGGCCAAGAAGCCGCCGCCGCAGCCGCTCCCCTTCTCCTGCGCCAGCG
CCACTGCGGCGGCGGCGGACGTGATGGACGCCGTGGCGTACGCCCATCATCAGGCCAGGCG
GAAGAAGCTGGGGAGGGAGAGGGAGAGGCGGAAGGAAACGCGAGAGCCGCGGCCACGGCCC
AAGAAGAAGCCCAAGCCCATGGCGACGAAGGACGCGGCCGCCGCCGCCGGGGCGCCGCTGT
CGTTCATGCCGTCGCCGGCGCCGGGCCCGCCCGTGAGCTCGGCGTTCCTCACGATGGCGGAG
CTGCCCGAGGGCCACTCCTCGCGGCGTGTCGTGGAGCTCATCTTCGCGTCCGGGTGGGCCGA
CGCGGCGGGGCGCCCGTCGCCGTCGCCGTCGCCGGCGGTGGAGGCGCTGTTCCGCGTGCAC
AGCGCGCCGCGCGCGGTGGCGCGGTTCGAAGAGGCGCGCCAGGCGGCCCGCGCGCGCGGC
GCCGCGGCGCGGTGCGGCGCCGACGGCAACGAGATGATGCGGTTCCAGTGCCGCGCGCCCG
GCGGCGGGGTGTTCGGCGCGGGCGTCGCTACCTGCCGCCTCGGCGCGTCGGTCTCTGCAGT
GCGCACGTTCGCCTGCAGCGGCGCCGCGCACGCCAGCGCCGCCGCCGCCACCGCCACCGCC
GGCCGGCGGGCGATGCTGGTGTGCCGCGTCATCGCCGGCCGCGTCTGCCCCGCCAACCACC
CGTCCTCGCGCCACGCCCACGGATCCGCTGCGACCGCCTATGACTCCGTGGACATGGGCAAC
GGCGAGCTGGTGGTCCTGGACAGCCGCGCCGTGCTCCCCTGCTTCCTCATCATCTACAAGGTC
TAG

>JL29_GRMZM2G178182
ATGGCAAGCTTATTCCCACAGCTCGCGCAGATGGAGCACGGCGTAGCCGGCGCCTCCCATGGC
GCGCCGAGCTTCCTGTTCTGCAATGGCGCAGGTGCAGCCACGGCAGACTCAGCCTCGCTAGAG
ACCTCGTCCGGTGTTCTCGACACCTCCCCACGAGGCGGAGGCGCTGATTCTGGCGGTAGGAAG
GCCCCGACGAAGCCCAGGGAAGACAGCGCCAGCGCCAGCGCCAGCTTCAGCTTCAGCTCTGC
GCACTCCAAGGACTCGAGCTCGAAGGGAAGCACCAAGAATAGGGGAGGGAAAAGAGGCAGAA
GCAGCAAGGAGGTAGACGAGGAGGAGGAGCCCAAGGGGTACATACACGTCAGGGCAAGGAGA
GGGCAAGCCACGGACAGCCACAGCCTTGCGGAGAGGGTGAGGAGGGAGAGGATCAGCGAGA
GGATGAGGGTGCTGCAGGCCCTGGTCCCTGGCTGTGATAAGGTCACTGGAAAAGCCCTCGTTT
TGGACGAGATCATCAACTACGTGCAGTCCTTGCAGAACCAAGTCGAGTTCCTTTCCATGAAGAT
CGCTTCACTGAGCCCTGTGCTGTATGGCTTTGGCATGGACACTACTGACGCTTTCAGCACTGAC
CATACTACTCAAAAAATCGAAGGGATGCTCCAGCTCCACCACGAGGCGCTTGCGGTGCCTAGCT
CTGCCCTGAGCAGACCTCCATTCGAGGCTATCGCGGACAGTACGCACGCCACCGCCTCTGCCT
CCTCCCCCCGGTCCTACGAGCTCCACGGCGATGGCGGCGGCGGCGGCGCCAGCATCTCTTTC
CCGCAGGACGGCGGCGGCTACATGGCGCAAACAGTAGGTGGGCCAAGGCCAGAGCTGTTCAA
CCAAGTGGTGTTCAGTAACCACATGTGCTCTTTCCAGTAG

>JL30_GRMZM2G140669
ATGGCGTCCTTCGTCGTGCACCACCACGGTTCGCCGCTGGACAGGGAGGGCAAGGTCAAGAT
GTCCGCCCTGGCCCTGAGGAGTAGCCTCAGGCCGTGCGAGGCCGCCGAGGAGGTCGGGGCC
GGGCAGGCGTGGTGCAGAGGCGCGGACATGGGCATCGCCGCCTTTGCCGATGAGTTCTCCGT

GGAGGACTTGCTGGACCTCGAGGACCTCTGCGAAGTTGACAAGGACTGCGCCGAGCTGGGCG
ACACGGTGCCGGCGGCGGAGTCTGTGCAGGAAGAGGACAAGGTGTCCAGTGACTCCCACGGG
TCGTCGGTGCTGTCCTACGAGCTCATGACGCTCCCGGTTCCGCACCCGATGATTGACCTCCCG
TTGCCGGCCCATGACGCCGAGGAGCTGGAGTGGGTGTCCCGTATCATGGACGACTCGCTCGC
CGAGCTCCCGCCGCCACCGAAGCTACCCGCGGCGCCGTTGGGAGCTGCGCGGCGGCCGCTG
GAAGGCGCAGTGGCAGGCCCTATGCGGAGCCCGACCACGACCATATGCGCGCTGTCGACGGA
GGCGCTAGTGCCGGTGATGAGGGCGAAGCGCAGCAAGCGCTCGCGTGGCTCCGTGTGGTCGC
TGTCCGGCGGAGCGCCCCTGTCGGAGTCGACATCGTCGTCTTCCACGGCCACCACGTCGTCCT
GCTCCTCGTCCGCCTCTTTCTCGCCGCTGTTACTCCTTCCGGCGGACCCGTCCCTCGTCCTGTC
CTCGCACCTCCTCGACGAGACGCCGCGGCCCAAGAAGAAGTCCAAGCACGGGAAGCATGGCA
AGAGCGGCAAGCCCAAGAAGCGCGGCCGCAAGCCGAAGCACCACCCCAAGCCGCCCCAGTTC
TCGGGCGGCGCCGCCTACGCGCCGGCCCAGGGCGACCGGCGCTGCAGCCATTGCGGCGTGC
AGAAGACGCCGCAGTGGCGCGCGGGCCCCGAGGGCGCCAAGACGCTGTGCAACGCGTGCGG
GGTCCGTTACAAGTCCGGGCGGCTGCTCCCGGAGTACCGTCCGGCTTGCAGCCCCACCTTCGT
GAGCAGCATACACTCCAACTCCCACCGCAAGGTGCTGGAGATGCGCCGCAAGAAGGATGGCGA
CCTTGGCCAGCTCCCGCTCCCAGTCCCGGCCGCGCTTCTGCTTGGGCCGGCCGTCGAGTCGT
TCTAG

>JL31_GRMZM2G472945
ATGGCGACCGTCCCAAACCCCAAGACGACCAGGGCCAGGAGGGAGCCGGCGCTGCGTCCCCG
AAGCAACAACGTCGCGGCGGCTCCGGCTCCGGCTCCGGCCGTGGCGAGCGTCAGGCGCGGG
CCGGCGGCATCCGCCGAGAAGGAGAACCAGCCGCCCAAGAGCCTCGGCCACGGGAAGGCGG
CGGCTGCGGCGACGGCCGCCGAGCCGCCGAAGCCCGCGGAGCCGGCGCCGGTTGTAGCGCC
ACCTCCGCCGCCGCTGAGGCCGTCGTCGCTGCAGCTCCGCATGAAGGACGAGGCGGCGGCG
GCGGCGCTGCCCGCGTTCGTCGTCGGGCCCAGGGGCAGGGAGCTGCTCCCGCCGCCGTCCT
CCAACTACGAGGCGTGGGACCTCTCCGACAGCGAGTCCGCGCCGGCCTCCTCCTGGGCCACG
CTCCCCAACAGGGCGCTGCTGTGCCGGCCTCTGCCTCAGGACGTCGGACGGTGCACGTGCGT
CATCGCCAGGGAGGCGGCCACCGGCGCCAGAGGCGTCGCGCTCTACTCCCTCTATACCAATG
AGGGGCAGGGGCGGCAGGACCGCAAGCTGGCGGTGGCGCGGCACCGGAGGAGGAGGGGCA
GGTCCGAGTTCGTCGTGGCGCAGAACCAGGACGGCATCTTCTGCACCGCCGACACCAGCTTCC
TCGGCGCGGTGCGCGCCAACCTGGTCGGATCCAAGTACCGAATCTGGGGCCAGGGTGACGAG
CTCAAGAACCAGTCGAAGCGGCTCCTTGCCGTTGTCGCGTTTGCCCCCACCATCACCACGCTC
ACCGGGAGTTTCAGGAGCTTGAGGGCATGGATCCCCAAGAACCAGTCCATGCAGCTCAAGGCA
AACAGTTCTGCTCAGATCCAGCATGTCAGTGGGCTCCCAAAGGATTGGCAGGAGAAGAAGAGC
AGAGCTGACCAGTTGTGCTCAAGAGCCCCGTTCTACAACCATATGACAAAGCGCTACGAACTGG
ACTTCAGAGAAAGGGCAGGAAGGATGGGGTACAAGGTGCAAACATCGGTCAAGAATTTTCAGAT
GACTTTGGAGGAAAATGGGAGGCAGACGGTGTTGCAGCTTGGTAGGGTTGGCAAATCCAAGTA
CATAATGGACTTTAGATACCCGCTGACCGGCTACCAAGCGTTCTGCATCTGCCTGGCATCGATC
GACGCGAAGTTGTGCTGCACCCTGTGA

>JL32_GRMZM2G146688_CDS
ATGACCAACAACAACAACGGCGGCAATGACACGAACGCCGCGGCGAGCGGCTGGCTAGGCTT
CTCGCTGTCTCCTCACATGGACGAGCACCAGCTCCAGCTCCAGCAGCAGCAGCAGCATGGCCT
CTTCTTCCCTTCCGTCACTGCGGGCGCCGCCGCCTCGTACGGCCTCGGCGGCGGCTTGGCCA
CCAGCGCGTCGCCGTACTACACGCCGCAGCTGGCGTCCATGCCGCTCAAGTCCGACGGCTCC
CTCTGCATCATGGAGGCGCTCCGGAGGAGCGATCAGCAAGACCACCACGGGCCCAAGCTCGA
GGACTTCCTTGGCGCGGCGGCGGCGCAGTCGCAGGCCATGGCGCTGAGCCTGGACAACCCG
GCGGCCGCCTCCAGCTTCTACTACTACTACTACGGAGGCGGAGGCGGCGGCGGGCCGGGGCA
CCACCACGGGTCGTTCCTGCAGCCGCCGTGCGTGGACCTGTACGGCGGGGCCTCGGCCGGGT
CCCAGCTGGTGGCGGACGACGAGGCCGCCGCGACGGCGACGGCGATGGCGAGCTGGGTGGC
GGCGCGCGCCGAGAACGGCGCACTGCTGGCCGCCGCCGGGCAGCCGCACCAGCACGCGCTG
GCCCTGTCCATCAGCTCCGGGTCGTCGCCGTCGAGCTGCGTGACGGCGCACCCCAGCTCCGA
GTACGGCGCGGCGGCGGCGGCGTCGATGGACGGCGGGCGCAAGCGCGGCGGCGCGGCGGG
GCAGAAGCAGCCCGTGCACCACCGCAAGTCCATCGACACGTTCGGGCAGAGGACGTCGCAGT
ACCGCGGCGTGACGAGGCATAGGTGGACGGGGAGGTATGAGGCGCACCTGTGGGACAACAGC
TGCAAGAAGGAAGGCCAGAGCAGGAAGGGCCGGCAAGTTTATCTCGGCGGGTACGACATGGA
GGAGAAGGCGGCGAGGGCCTACGACCTGGCGGCGCTCAAGTACTGGGGCCCTTCCACGCACA

TCAACTTCCCGCTGGAGGACTACCAGGAGGAGCTGGAGGAGATGAAGAACATGACACGGCAGG
AGTACGTGGCGCACCTCAGGAGGTACGTACGTACGTCGTTTCACTTCACCAAATGA

>JL32_GRMZM2G146688_GENOMIC
ATGACCAACAACAACAACGGCGGCAATGACACGAACGCCGCGGCGAGCGGCTGGCTAGGCTT
CTCGCTGTCTCCTCACATGGACGAGCACCAGCTCCAGCTCCAGCAGCAGCAGCAGCATGGCCT
CTTCTTCCCTTCCGTCACTGCGGGCGCCGCCGCCTCGTACGGCCTCGGCGGCGGCTTGGCCA
CCAGCGCGTCGCCGTACTACACGCCGCAGCTGGCGTCCATGCCGCTCAAGTCCGACGGCTCC
CTCTGCATCATGGAGGCGCTCCGGAGGAGCGATCAGCAAGACCACCACGGTACGTACGTACGT
GCGCCACATCCATACTGAGCCGATGGCCTCGCGTCGACCGGCCCGCTGGTCGTTGTTGTTGAT
CGATCGGCGTCATTGTTGGTTCACTCAGGGCCCAAGCTCGAGGACTTCCTTGGCGCGGCGGCG
GCGCAGTCGCAGGCCATGGCGCTGAGCCTGGACAACCCGGCGGCCGCCTCCAGCTTCTACTA
CTACTACTACGGAGGCGGAGGCGGCGGCGGGCCGGGGCACCACCACGGGTCGTTCCTGCAG
CCGCCGTGCGTGGACCTGTACGGCGGGCCTCGGCCGGGTCCCAGCTGGTGGCGGACGACG
AGGCCGCCGCGACGGCGACGGCGATGGCGAGCTGGGTGGCGGCGCGCGCCGAGAACGGCG
CACTGCTGGCCGCCGCCGGGCAGCCGCACCAGCACGCGCTGGCCCTGTCCATCAGCTCCGGG
TCGTCGCCGTCGAGCTGCGTGACGGCGCACCCCAGCTCCGAGTACGGCGCGGCGGCGGCGG
CGTCGATGGACGGCGGGCGCAAGCGCGGCGGCGCGGCGGGGCAGAAGCAGCCCGTGCACCA
CCGCAAGTCCATCGACACGTTCGGGCAGAGGACGTCGCAGTACCGCGGCGTGACGAGGTACG
TACGTACACGCCGCCGTGTCGAGCTAGTTGACTGGTCCGTCCGCCCGTCCGTCCATGGAGCTG
AAGCTGAAGTTGAAACTAAAACTAAAACTGAAAGTAATTAAGCAGGCATAGGTGGACGGGGAGG
TATGAGGCGCACCTGTGGGACAACAGCTGCAAGAAGGAAGGCCAGAGCAGGAAGGGCCGGCA
AGGTAACAACAAATTGATGAAATTTCAGCAAATTTATCTATCTATCTATCTATCTAAATCTAAATGG
GTGTTTTTTTGGCTGTGGGGACGATGGCGGACTGCCTGTCTGTCTGTCACGCTGCAGTTTATCT
CGGTAAGCACTCTTTGCTGCACATAAGCACATTGATGGTGAAAAAGTAAAAAAAAAACTGCGAG
CTGAGCTGACTGCTGAGAGCGTGGTCGGTATTGTATATTTTTAGGCGGGTACGACATGGAGGA
GAAGGCGGCGAGGGCCTACGACCTGGCGGCGCTCAAGTACTGGGGCCCTTCCACGCACATCA
ACTTCCCGGTAATAATTTTTTTTTTGCTTTTGTTTTACCGTGGTGCATCATGGTAAAAATCCTACC
ATTGCTGCGCTGATACTGTGCTGCGTGTGTGCCTGCAACCTGAATGAAGCTGGAGGACTACCA
GGAGGAGCTGGAGGAGATGAAGAACATGACACGGCAGGAGTACGTGGCGCACCTCAGGAGGT
ACGTACGTACGTCGTTTCACTTCACCAAATGA

>JL33_GRMZM2G111045_CDS
ATGGGAAGGGCACCCTGCTGCGACAAGAACAAGGGCCTCAAGAAGGGGCCCTGGACGCCGGA
GGAGGACAAGCTCCTCGTCGACTACATCCAGACCAACGGCCATGGCAGCTGGCGCCTGCTCCC
CAAGCTCGCAGGGCTGAACCGGTGCGGCAAGAGCTGCCGACTGCGGTGGACCAACTACCTCC
GGCCGGACATCAAGCGCGGGCCCTTCACAGCCGAGGAGGAGAAGTCCATCGTCCAGCTCCAC
GCCATCGTCGGCAACAAGTGGTCCATGATCGCGGCGCAGCTCCCAGGCCGGACGGACAACGA
GATCAAGAACTACTGGAACACGCACCTCAAGAAGCAGCTGCGCCGGATGGGCCTCGACGAGCC
CCCGCCCGGCCCGGCCGCCGGCTGCCCCGCCGCGCGCCACATGGCGCAGTGGGAGACGGCC
AGGCTCGAGGCCGAGGCGCGCCTCTCCCTCCTCGCCGCCACCGCCGCCTCCTCCGGCACCAC
CGCGACCACCACAGCCACCACCACCACGTCTGCAGCCTCCTCGTCCTCCACCACCGTCGCTGG
CGCGGCCGACAACAAGGCCTCCAACCCCGCGGACATCTTCCTGCGCCTGTGGAGCTCGGACAT
CGGGGACTCCTTCCGCCGCAAGACGGACACGACGCCAGCGGTGCCCGCGCTTCCGGCGCCC
GTCGTCAAGAGGAAGGACACGGCGATCAAGCAGGAGCCGCGGGCGCTGCTGCTCGGCCTCAG
GGACGACTCCTCGGCGGCGTCCAACGACAACGAGACGGAGGTGGCGGAGGCGCTGGAGGCG
AAGTACCAGATGTTCCTCGACTTCGCGGGCGAAGAGCTGGGATTGTTCCATGGCCGGCACGGC
GGCTTCTCGCTGTTCCCGCCGCTCGACGTGCTCGCTGAGGCGTCCCTGGACTCGGCTTTCAAG
TGA

>JL33_GRMZM2G111045_CDS
ATGGGAAGGGCACCCTGCTGCGACAAGAACAAGGGCCTCAAGAAGGGGCCCTGGACGCCGGA
GGAGGACAAGCTCCTCGTCGACTACATCCAGACCAACGGCCATGGCAGCTGGCGCCTGCTCCC
CAAGCTCGCAGGTCAGAATTCCTCTCTTTATTAGTCCCTTCTCTTCGCGGTTTCCACCGTCGTCG
TCTTAGCTTAGTTCTGCCCGACCATTGGTGTCGCACTCGCACTGGCCTAAAATGCCGACCAAAT
TCCTCGCCGGAGTGAACGATGCTTACTCTATTTCTGGGATGGCAATGGCATGCAGGGCTGAACC
GGTGCGGCAAGAGCTGCCGACTGCGGTGGACCAACTACCTCCGGCCGGACATCAAGCGCGGG
CCCTTCACAGCCGAGGAGGAGAAGTCCATCGTCCAGCTCCACGCCATCGTCGGCAACAAGTAA

GAAAACTTTTTTGCCGAGCCGAGGTAGCAACTGCCTGCCCGTCCCATTCGATCTGTGGCACCG
GCAGAGAGCAGCACGTTTCCTCTCTCGTCATGCTCTCATCTCACTCACTCATCATAGCTTAGAGA
GCTCGCACTGCCGGTGTACTGCTCGGCGCAGCGCCTCACTGTCGCAAAGCATCGCGTCGCATT
GAATGATGCCACCTCCGTCCAAGGCATCGCGTCCAAAACCTGGCGCCTTCAGCATGCCGGATG
TGTATTGCTATTGCCTCGCTCTCGTAGACGTAGTACTCCTATTTACTAGAGCCCTGTCTGCTCAT
ATTTCTGCCACGGCAATTGGTGATTGGCTGATTGCTATTGGCAGGACAACTCTCTCCCTCTCCG
GAAAAAAAGTTTGCAAACTATTCTCGCTGCTTGAAGAGTCAAACGGTCTAAACTTCAACTAAATTT
ATATAAAAAAATACAAACATTTTCAGTACAAAAAGAAGTGTCATTAAATTAGGTATACACACGCTG
GTGCTAATTACTATAAACTTGGACAGTTTGATTGTCATAAATAATATTGTTGCATTCTCTCGAGAA
CGAACGGCATACGTTGACGGTTTTTTTTATCTGGTGTTGATCCTGGTACCTCAAGGTGGTCCATG
ATCGCGGCGCAGCTCCCAGGCCGGACGGACAACGAGATCAAGAACTACTGGAACACGCACCTC
AAGAAGCAGCTGCGCCGGATGGGCCTCGACGAGCCCCCGCCCGGCCCGGCCGCCGGCTGCC
CCGCCGCGCGCCACATGGCGCAGTGGGAGACGGCCAGGCTCGAGGCCGAGGCGCGCCTCTC
CCTCCTCGCCGCCACCGCCGCCTCCTCCGGCACCACCGCGACCACCACAGCCACCACCACCA
CGTCTGCAGCCTCCTCGTCCTCCACCACCGTCGCTGGCGCGGCCGACAACAAGGCCTCCAACC
CCGCGGACATCTTCCTGCGCCTGTGGAGCTCGGACATCGGGGACTCCTTCCGCCGCAAGACG
GACACGACGCCAGCGGTGCCCGCGCTTCCGGCGCCCGTCGTCAAGAGGAAGGACACGGCGAT
CAAGCAGGAGCCGCGGGCGCTGCTGCTCGGCCTCAGGGACGACTCCTCGGCGGCGTCCAACG
ACAACGAGACGGAGGTGGCGGAGGCGCTGGAGGCGAAGTACCAGATGTTCCTCGACTTCGCG
GGCGAAGAGCTGGGATTGTTCCATGGCCGGCACGGCGGCTTCTCGCTGTTCCCGCCGCTCGA
CGTGCTCGCTGAGGCGTCCCTGGACTCGGCTTTCAAGTGA

>JL34_GRMZM2G377217
ATGGAGGGAGGCCAGCTGGGGACTTGCCTTCCTAGCTTCCTCGTGCCGGATCACTACGCCTTC
CCTCTTCCGCTCCCGCCGCTACAACTTCCTAGTAGCCAACCGAACAACAAGCCTTTCCAGATGC
CGTTTGACGACGTCCAGGAAGAGGCCGGGAACCACGCCGGGATGCTCTCCTCCTCCGACCACT
GTGGACTATACCCGCTGCAGTCACTGCCCTTCGGCAGTAGCGCCTCCGGCGGCGCCGGCGCG
GCAGCGTGCGGTGGGAAGCCTACGGCCGTCGGTTTCATGCCCAGCTGTAGTGCTGTCGGCGC
TGAGGAGGTCTGCACCTCGGTGGCGCCTACTACTAATGGTTGCAACGAGAGTAATAGCGCATG
GTGGAAGGCCTCAGCAGCGGAGAGAGGGAAGATGAAGGTGAGGAGGAAGATGAGGGAACCGA
GGTTTTGCTTCCAGACCAGAAGCGACGTGGATGTACTGGATGATGGCTACAAGTGGAGGAAGT
ACGGCCAGAAGGTTGTCAAGAACAGCCTCCATCCAAGGAGCTATTTCCGGTGCACTCACAGCAA
CTGCCGCGTGAAGAAACGGGTGGAGCGGCTGTCGACGGACTGCCGCATGGTGATGACCACGT
ACGAGGGCCGCCACACGCACTCTCCCTGCAGCGACGACGCTTCCTCCGGCGACCACACCGATT
GCTTCAGCTCCTTCTGA

>JL35_GRMZM2G023051
ATGGAGGAGGACCCGCTCATCCCGCTGGTGCACGTCTGGAACAACGCCGCCTTCGACCATGCC
TCCGCCTCCGCCTCCGCGTGGCACGCCCATTCCCCTGCGCCCGCGAGCGCCCGCGAGGGGG
ACAAGGAGAACCACCGCCCCGAGCCCGACGTTGAGGCGGAGGTCGACGACGTTGAGGCCGAG
ATTTACCACATCGAGGCGGAGATCCTGCGCCTGTCTTCCCGCCTCCACCACCTCCGCACCTCCA
AGCAGACGGAGCCGCCCAAGCGTGGACAGGTCGCGCGGACGCGGATGCGGGGGCTCAGTCT
GGGCCAGCTCGACGTCGCCGCTGCCGTCGACTCCGACCCGCTCACCGACAAGCAGCAGCCGA
GCGCCGCGCAGGGCTTGAAAAAGCCTATCAAGCAGGCCCCGGCGGCGCCGCGAGGCCGGAG
CTCCAGCCTCGGGCCCCTCGAGATCGTCGCCACGAACCCTAGGGTCCTCCGCTCCTCCGCCG
CCGCCGCTGCCGCACCGAAGAGGAAGATTCAGGGCGAGGGCGGCGCCGCGCGGCCGATCCT
CAGGCCGATCAAGGAGCCTCCCGTGCAGCGGCGCCGGGGCGTCAGCCTCGGGCCCTTGGAG
ATCCACCACGGCGTCGGCAGCAAGCCCGGGGCGGCGGCGCGGGTCAAGCACTTCTCCAACAA
CAGGCTCGGTGCCGTTCGAGAGGAGGAAGGCCAGCGTTCCAAGGAGCACGCCGTCCCCGCCA
GACCGTGGCCATCCAGCAATGCCAGGCACCCACTGGATGCCAGGCAAGGCACCGCAGCAAGC
AGAGCCAAGGCGAGGAGCGGGAGCATAAGCCCCAGCAGGTTCAGGAGGCAGTCCACTTCCAA
GGCTGCCGAGACAAGAGCGGGAAATGCCAAGCCTACAGAGGCGACGAGGGGAGGGAGCGAA
GCGGTCAATCACACCAGCAATGTAGCCACGACGAAGAGGCCGGCGGGGAGCTCCAAGGTCAG
GGTTGTCCCGAGCCGCTACAGCATCCCACCTGGCTCCTCCCTAGCAGCTGTGACACAAGGCAA
CCGATGCAAGCAGTCTCTCCCAGGATCGGCTACTGAGACCAGAGTAAATCTCACTGAGCCGCC
GAACGACGAGTTGTCTCCTGAAGAACTTGCCAAGGTTGCAGAGCTGCTCCCAAGGATTAGGAC
CATGCCGCCTTCTGATGAGAGCCCGCGTGACTCGGGATGTGCCAAGCGTGTTGCTGATTTGGT

CGGGAAGCGATCCTTCTTCACTGCTGCAGGGGACGATGGCAATCTCGTTACGCCCTACCAGGC
ACGGGTGGTTGAACTTGAATCACCCGAGGCAGCAGCAGAAGAAGCAGAAGCTTGA

>JL36_GRMZM2G109480
ATGGAGGCGGCAGTCGCGGAGACGCAGGGGATGTCGCCGTACGAGGCGGCGCGGGAGCGGA
ACCTGCAGGAGAACAAGCGAAAGATGGAAGCCCTCAACCTGCGCCACCTCTCCGCCGTCATCA
AGGAGGCCCCCAAGACCCCTTCCCCCATGAAACCGAAGAGGCGCAGGATCATCGAGGACGCG
GTCGTGGTTCCCTCGCCTCCCAGGAGGTCCCGCCGGCTTGCCAATGTCCCTGAGGTCAAGTAC
GCAGAGATAGCACCACACCGTGCAGATAGAATGACAAGGTCTCCTAGAAGACCAGCTGACCTC
ATCTACTTGGCAAGACGCGGATCAATTTCCATGAAAGCTAGGCTGGAGGCAACAAGGAAGGCT
GAGGAGCTAGAATCACAACTCGATCCTGAAATCCCATCCTTCGTGAAGGCGATGTTGCATTCGC
ATGTGGTTCGAGGCTTTTGGCTGGGTCTCCCGAGCCATTTCTGTGACACTTACATGCCGAAGCA
AGACGCCACTATCACCTTGGTGGATGAGAAGGATGAAGAATTCGACACCAACTACCTTGCCTAC
AAGAAGGGGCTAAGTGGCGGGTGGGCTGGTTTTGCGCTATACCATGCGATTCAGGATGGAGAT
TCAACTGTTTTTCAGTTGATTAAGCCCACGACCTTCAAGGTGCATATCGTCCGAGCAGCTACTGA
TGATAGCAGTGAAGTAGATGAGTGA

>JL38_GRMZM2G074032
ATGGCACTACTGGTCAAGAGCCACCAGCAAATGTTGGTCTCCTCCTCCACCACCTCCTCGTCGT
CCCCTTCGTCGGCGTCCCAGCAGCGGCACCCGCCGCCACCTCCTCCTCCCTCCTCCTCCTGCC
TCACCGACCAGCAACAGCCGTCCCCAGCCAAGCGCAAGAGGCGCCCTCCCGGCACGCCGGAC
CCCGATGCGGAGGTGGTGGCGCTGTCGCCGCGGACGCTGCTGGAGTCGGACCGGTACGTGTG
CGAGATCTGCGGGCAGGGGTTCCAGCGCGAGCAGAACCTGCAGATGCACCGGCGGCGGCACA
AGGTGCCGTGGCGGCTCGTGAAGCGGGCGCCGCCGCCTGCTGGCGGCGGCGAGGACAGCGC
CGGCGCTAATAATAGTAGCACAGCTGGTACAGGCGTTGGCGGCGGCGGCGGCGGCCCCCGGA
AGCGCGTGTTCGTGTGCCCCGAGCCGAGCTGCCTCCACCACGACCCCGCCCACGCGCTGGGC
GACCTGGTGGGCATCAAGAAGCACTTCCGGCGCAAGCACGGCGGGCGGCGGCAGTGGGTGTG
CGCCCGCTGCGCCAAGGGCTACGCCGTGCAGTCCGACTACAAGGCGCACCTCAAGACCTGCG
GCACCCGCGGCCACTCCTGCGACTGCGGCCGCGTCTTCTCACGGGTGGAGAGCTTTATCGAG
CACCAGGACGCGTGCAACTCCAGCCGGATACGCGGCGAGGCCGTGGCGGTAGCCGTGCCGG
CGCCGCCGTCGGCGCTCCCGGTCATCCGCCCCGCCGTCCCGCGGCACCAGCCCACGGTGGC
ACCGGCACCGCCGCCACCGGAGCTCCAGCTCCTCCCCGCCTCCGCGGCGGCATCAGCGCCGC
TGGCTAGCACACCGACCGCCTCTCTGTCCACCACCACAAACGCGTCACAGGAGCAAGAACCCC
ACGCGGCGGCGGCGGCGACGACGACGACGAAGCTGCAGCTCTCCATCGGCCCCACCGTCATC
GCGCCGGGCGCCGACGCTGCTGCGGCGACCGCCTCCGCCGTCGGCGAGGAGGACGACTCCG
AGGCGAAGGAGCTGCGGCGCGCGATGAAGGAGAAGGCCGCAGCGGACGCGGCGCGGGCGC
GGGCGCGCGGGGAGGCCGCCGCGGCGGAGCGCGCGCTGGACGAGGCCCGCCGCGCCAGGC
AGCGCGCCCGGGCCGACCTCGAGAAGGCGTGCGCGCTGCGGGACCACGCCGCGCGCCTGCT
CGCGCAGGTCACCTGCCACGCGTGCCGCCAGCGCTCGCTCGCCGTGGCCGGCGGCCCCGCG
GTGGCCTGCGACTCCATTATCAGGGGAGGAGGAGGAGAAGCAGGCATCTAG

>JL39_GRMZM2G027068
ATGTTCCCGGTCGAAGTGTCGGCGCTCGCGGCGGGCGGTGCGGGAGCGGGGCGTCAGGCTG
CCGGCGACGCCGCCGGGACGATGCTGCCGCCCTTTTTCATGGGCTCCATCTGGCCCGCGACC
GCTGGCGCCGCTGGCTCCGAGGAGGACGAGGCGGCGGCCGCCGCCGCCGCCCACGACCGC
GCGCTCGCCGCGTCGCGCAACCACCGCGAGGCCGAGAAGCGCCGTCGCGAGCGCATCAAGTC
GCACCTCGACCGCCTCCGCAACGTCCTCGCCTGCGACCCCAAGATAGACAAGGCTTCGCTGCT
GGCGAAGGCGGTGGAGAGGGTGCGGGACCTGAAGCAGCGGGCGGCGGGCGTCGGGGAGGC
AGCGCCGGCGCACCTGTTCCCGACGGAGCACGACGAGATCGTTGTGCTCGCGTCGGGCAGCG
GCGCCGTCTTCGAGGCCTCCGTCTGCTGCGACGACCGCTCCGACCTCCTCCCGGACCTCATCG
AGACGCTGCGCGCGCTCCGCCTCCGCACGCTCCGCTCCGAGATGGCCACCCTCGGCGGCCGC
GTCCGCAACGTGCTCGTCCTGGCGCGCGACGTCGTCGACGGCGGCGGCGGTGCCGTCGCGG
GAGGTGATGACGGATACGGTGGACGCGCTGCTGACTCGGCGGGCGGCGCCACGGACGGCGG
CGGCGGCGACTTCCTCAAGGAAGCCCTGCGGGCTCTGGTAGAGAGGCCAGGCGCCGCCGGA
GACCGGCCCAAGAGGCGACGGGTCTCGGACATGAACATGCAAGCGGCAGCATAG

>JL40_GRMZM2G140694

ATGATGGCCGGGGCACCGCCGATGCACATCTGCCTGGACTCGGACTGGCTCAAGGGCATCGTT
CCCGAGGAGCCTGGGATGGGATCGTCGTCACCGTCAGCGGAGCTGATCGCGTGCCCTAGGCC
AATGCACGCCACGGCTGCCGCGGCGGACCGGCGTCTGCGCCCGCAGCACGACCAGCCACTCA
AGTGCCCGCGATGCGAGTCCACGCACACCAAGTTCTGCTACTACAACAACTACAGTCTCTCCCA
GCCGCGCTACTTCTGCAAGACGTGCCGCCGCTACTGGACCAAAGGCGGATCGCTCCGCAACGT
CCCGGTTGGTGGGGGATGCCGCAAGAACAAGCGCGCCAGCGCCAAGAAACCACCCGCGCCAC
CGATGCAGCCGCCGCACATGTCGGAGACGGGCCTCCACCTGTCCTTCTCCGGCGTGCAGCAG
CTGCCGCCGGCCGATCCGCTTTGCAGCCTCGGCCTTTTGGACTGGAAGTATGACCCGATCCTC
ACAGGTTCCGGTGGCGTCGCTGGCTCGTTGGATGGCGCCAGCTCCGAGACGCACTTCGCAGG
GACGGGCATGCTGGGCATCCCAGGTGGCAGCGGGGGCGGCGAGTGCCACGCGCTGAGCGCG
CTCCGGTACGCGGCCGGCCTGGGCGAGAACCTGCAGGTGCAGGGTCTCCCGTTTGGGGCGCG
CGCTGAGCACGACGCGATGGAGATGAAGCCTGCGGCGACGGACAGGCTGCTCTCGCTGGACT
GGTACAGCGAGACAAGCCGCGCGCCAGAGAGCGCCATCAGCTCGTTGGGCGCGCTGGGCCTG
TGGAGCGGCATGCTCGGCGGTGCGCACCAGCACCATGGCTCGTCGGCGGCCATCTAA

>JL43_GRMZM2G098988
ATGCAGGGAGACCCCGGTTACGGGTACGGTGGCTACGGCGCCGGCGCCGGCGGCTACGGCT
ACGACTACGACGTCGCCGCCAACTGCGCCGGAGGCGGCTACTACTCGGCGGCGAACGACCGG
TACCCTGCCGCGCCTCCGCCTGCTGCAGCCTATGAGGATCCGCTCGGTGGTCGGAGGCAGCA
CGACTTCCCGGCGCCGCTCACGGGGCTGGAGTTCCAGCCGTCGGACACCTGTCCCAAGAACTA
CGTCATCTTCGACCAGACGTACGACCGGAGCCGGGTGATGTTCCACCCCTCCCTGGCCAACAA
CTTCGGTTCCTCTGCCGGCGGATACGACTACGGCAACCACTGCTACGGCTACGACCAGACCTA
CCCCGGCGAGAGCGCCTACCACGGCTGCGGCGACGACGCCGGCGCCGGCGCCGGCGGCGTC
TCGGTCCGGCAGAAGGAGGACACCGACGAGATCGACGCGCTCATGAGCACGGAGGAAGGCGA
CGAAGAGGACGACGTGCTCAGCACCGGCCGCACACCCGGATGCCGCGCCGGCGGCTCCCCG
GATTCCACGTGCTCATCCGGCGGGTACGCTGGGAACAGCAGCGGCGGCCGGAAGCACGAGAC
GGGCGGCGGTGAGAAGAAGAAGGAGCGGATGAAGAAGATGGTGCGGACGCTCAAGGGGATCA
TCCCCGGTGGCGACCGGATGGACACGCCGGCCGTGCTCGACGAGGCCGTGAGGTACCTCAAG
TCGCTCAAGGTGGAGGTCAAGAAGGCCGGCGCGCGTGGGTCAAGCAGCTAG

>JL44_GRMZM2G123900
ATGTTCTTGAATATACCGTGCTTTGAATTCCAGCCTTTATTAATTGATTCGTTATACATACAGATG
CAGATGCAGATGCAGCAGCAGTCACCGCTCCAGTGTCTCCTCGGCAGCGGCGGTGGCAGCGA
CCACCACCACCTCATGCCTCCTCCGTCCGGCCTGGCGCCGCTGCCGGGGGGTCCTGCTGACA
CAGCGGCGAGCGGTCCGGCGGGAGGCGGCTCGTCCACCTCGGCCTCAGTGCAAGCCGCGGC
GGGAGCGGGGGCAGGGGCGCAGCCTCGCCCCGTGGTGTCGATGGCGGAGCGCGCCCGGCTC
GCGCGCGTGCCGCTGCCGGAGCCCGGCACGCTCCGATGCCCGCGCTGTGACTCGACCAACAC
CAAGTTCTGCTACTTCAATAACTACTCGCTGTCGCAGCCGCGCCACTTCTGCAAGGCGTGCCGC
CGCTACTGGACCCGTGGCGGCGCGCTCCGCAACGTGCCCGTCGGCGGCGGGTGCAGGCGCA
ACACCAAGCGCTCCAGCAAGAAGTCGTCCCGCGGCGGCGGCGCGGGCGCCACGGCGGCAAC
CTCCTCGTCCTCGACCACCTCCACCTCCACCACGGCCACTACCACCACCACCACGAGCGCGGC
CATGGCGGCGGCCGAGGCCATCGCCGGCATGCAGGCGCAGCTGCCCCACCTCGGCCTCCCG
CCCGCAGCGGCCGCCGCGGCGCTTGAGGCCTCGCTGGAGGGCTACCACCACTACCTCCCGCT
CCAGATGCAGCCGCAGTTCCTGCAGCAGGCTGGCCTGCACGGCTACCATTTCGCCGACGACG
GCACCGGCGTCCTCGCAGCTGACGGGTTCCCGAGGGGCGTCGTCGCCTCGGGGCTGCTCGC
GCAGCTCGCGGCGGTGAAGATGGAGGAGCACGGCAGCAACGGCGGAGGTGCCATCGCGGCG
CATCATGAGCAGCAGTCCTACTGGCCCGGCAGCACCGGCGGTGGCGGTGGGTGGCCGGTGGA
GTTCTTGTCGGGGTTCAGCTCATCCTCGTCGGGGAATGTGTTGTGA

>JL45_GRMZM2G374986
ATGGTGAGGACGACGGTGGTCGGCAGGGAGAGGAGCGGCGGCGTGAGGCAGTACAACAGGT
CCAAGGTGCCTCGCCTCAGGTGGACGCCCGATCTGCACCACTGCTTCGTCCACGCCATCCACA
AGCTCGGAGGCCCGGACAGGGCTACGCCGAAGCGAGTGCTGCAGCTGATGGGGGTGGGAGG
ACTCACCATATCTCATGTCAAAAGCCATCTGCAGATGTACAGGAACATGAGAAATGATCTTGGAA
TGCAAGGGACGGAGGATCAGGAGCACGTATACGTAGGTGGAGGAGGCATGCATACCAATATAC
AGCAGCAGCGGGACCATGGGTGTGGCGATGCCCCCTGCTGCATCTGCTGCCGCCACTCGCCA
AATAAGCCTGCAAAGGAGGAGGAGGAGGCGACGCTGTTCCAACGCCAACTGAAAAGGTCGAGG
ACGGAGACGAGGCGTGGGCGTGGCCAGCGGGAGGAGATCGATGATGGGAGCGCGAGCCCCA

AGAGGCGGCTGCTGCTGCTGCGAGGAGGGCGAGGCCGCCCAGGAATATGTGAGAGCGACGG
CTCAGGCTCACCCAGCAGCGGCACGCTCGCAAGTGGCTACGACAACTACATGCGCATGATGCA
AGCGGCCATGGGCGTGGCTGCCGCTGCCGGCGCTGCTGCTGCTGCTGCTCCTCATCATCATCA
TCATCATCACGCGGAGACGGTGGAGCCCGGTCGAGGGCCGAGAATAAAGCAGCACTTGGCGC
GGCCCGGCGCAGACGGCGGCGAACACGCTCCTCCCAGCAGCAGGCAGCTCTTCACGTTCTTG
GGCTTCGTGGTGGCGCCGGGGCCGCCGCCTCCTCCCTGCTGCAGCCGTGACCACCCTTTCGA
GATGGTCGGCACGGCTCTCACCCACCGCAGCCTCCAGACACCGCCCACCAAGGCCGCCGTGG
CCCAACAAGTCGGTGGCGATCCCTGCTGGTCGTCGGCGGCCGCTTCCGAACACGACGACCAC
CACCACGACCGCGACCGCGACGGCTGCTGGCTCTCGCTGTCCCTTGCGCTGGAGACGAGAAG
CAGCCGCAGCGCCGACGAGGGCGGCCGGCTCTCGCCGACGGGGAGCCGGATCAGCCTCGAC
CTATCCTTGTCCACGATATAG

>JL46_GRMZM2G011463
ATGGCGAAGGGCGGGCTGAGCAAGCTTAAGTGCATGATCAAGAGGTGGCACTCCTCGAGCCG
GATCTCGCGCACGCCGTCAGGGTGCTCGGCGCGCTCTCACGACGTCGGCGACGCTGGCGCCG
GGTTCGCTCTGGAGAACTCGTGGAGGAAGGGCGCCGCTGCCTCGTCCGTCTTTGCCTTCGGCG
GCGGTGGCACGGGGTCGGCATCGTTCCACGGCACCGACGGCCTGCCCCCGGGCCTCCACCC
GGTCTACGTCGGCAAGTCGCGCCGCCGCTACCTCATCGCCGCGGACCTCGTCGGCCACCCCC
TGTTCCAGAACCTCGTCGACCGCTCTGGCGGCGCCGGCGTAGGCGCCGGCGGACCGTCGTC
GGCTGCGAGGTCGTGCTGTTCGAGCACCTCCTCTGGATGCTCGAGAACGCCGACCCGCAGCC
GGAGTCGCTTGCCGAGCTCGTCGAGTATTACGCGTGCTGA

>JL47_GRMZM2G097275
ATGGGCTCCTTTGGGATGGACTGGAACCAGAAGGCCTCCGTGTTGTGGGACTGGGAGAACTTG
CCGCCCGTAGCCGTAGGCGCGAGCGGGAGCGAGAACCCCAGGATGGCTGCTGCGCCTCAGG
CTTTGCATTCTGCCTGTGGCACCATCTCTTCCAGCTCAGAGATGGGGTATGGCTCATCCAAGAG
CTCCGTATCCGCGTCGGCTGATGATTCTTCACCCAAGGCCAAGGGGAAAAGCATGGAGCTCAA
CTTCGCACCTGACAAAGTGCCTGACAAGAACACTGATTCAGGGAAGGTTGACGACGCCAGGAC
CTCTCCGTCGTCGCCGGTGATTGCCATCAGCAGTGCAGAGCCCGTGCTTAGCCTGAAGCTTGG
CAAGAGAACGTATTTCGAAGATGTCTGTGGAGGGCAGAGCGTCAAGAGTTTGCCGTCGGATAC
GAGTGCAGTGACTCCTCCTCCTGCTTCTCCGAAGAAGACGAAGGTGACTCCAAACAAGAAATCT
TACTGTCAGGTTGAAGGCTGCAAGGTTGATCTCTCTTCTGCTAAAGAGTACCATCGGAAGCACA
GAGTCTGTGAACCTCATTCTAAGGCTCCCAAGGTGGTTGTTGCTGGTCTGGAGCGGCGCTTTTG
TCAACAGTGTAGCCGGTTCCATGGATTAGCTGAGTTTGACCAGAAGAAGAAAAGCTGCCGCAGG
CGCCTCAATGATCACAATGCCCGCAGACGGAAACCACAACCTGAAGCACTTTCTTTTGGGTCAT
CAAGGCTGTCGGCAATGTTCTATGACACAAGACAACACACAAGCCTTCTGTTTGGTCAAGGTCC
TTATGGTCAAATGAGAAGTTGTGCAAGTTCTTCATGGGATAACCCTGGAGGTGGCTTCAAATTTG
CAGAGACAAAAGGTCCCTGGTTAAGAGCTGGTGGTGTTGATGGGTTGCATCAACCAAGTCAGCA
GGTGTGGAACAACTTTATGCCACATGGTGTCCATCAAGATTTTGATGGGTTCACAGCTTTCAAAG
GAACCAATGCAAAGGTCCTTCATCAAGGCGTTGAAGCTTCTGCGGTCGCCTCCAACCAGAACG
GCGCCCCACAACCGGACCTTCATCGTGCTCTCTCTCTTCTGTCAAACAGCTGGGCTGGTGGTG
GTACCGGTACCGGTACCGACCAGCACCCGACGGCCCAGCTGCGCCACGCTGCCCTGAGCAGC
AGCCTCGCCGCCAGCTCCGTCTCCGTGGCGCAGGCCTCGCCAGCAGGGCTGTGGCAGGACGG
CGGCGCGGCCCTGGGCCACCACGACTACGCCCACGCGTTCCAGGCTATCAACACGCCGGGCG
GCGGCGGCGCCGCCGCTCCCATTGCAGTGGGTCAGGAGCTGCTTCAGCTCCCGCGTCCGCCG
TCCCTGTACGACGACGGCTCCTCGTCCCGCTACGACCTGATGCGCTGA

>JL48_GRMZM2G148467
ATGGGGTGCCACGCCACGCCAGCGTGGGTAGCATACCGGCTGTACTTCCAGCAGCAGCATCAT
CAGCCGCCGCCGCCGCCGCCGCGCTTCCACTTTGTCTTGAGCCCTGCCCTCAACGACGACTCG
CGGGCCGGCAGCACAGCACGTATAGCTATGGAAGGAAACGGTGGCGCGGGCGGCGGTAGCG
GAAGCGCGGCACCGCCCTGGGATCTCGCCATGCACTGGGCACCCGCCGTAGTGTCGTCCTAC
CCGCCGCAGCCCTTGGAGCTGCAGCAGCAGGAGCTTACCTGCCTCAAGCTGGGGAAGCGGCC
CGCCTGCTGCTGGGCAGGGGCGCCGGGCAACCAAGCGGCGCAGGTCCACGGCAATGGCGGC
GCTGGTGGCGCAGCTGCTGAGGGTAAGAGGAAGGACAAGGCGCCTGCCGCGGCGGCCGTGA
CGAGGTGCCAGGTGGAGGGGTGCCACCTGTCGCTGGCGGACGCCAAGGAGTACCACCGGCG
GCACAAGGTGTGCGAGGCGCACTCCAAGGCGCCCCGGGTCGTCGTCCTCGGCGCCGAGCAGC
GCTTCTGCCAGCAGTGCAGCCGGTTCCACGCGATCTCGGAGTTCGACGACGCGAAGCGGAGC

TGCCGACGGCGTCTGGCCGGGCACAACGAGCGGCGGCGGAAGAGCAACGCCAGCGAGGCCA
TGGCAAGAGGCGTCGCGCACCCACACGGAGTGACGGCTTTCGGCCACGGCGGCTTCCTGCCC
TCGCGCGGCCTCGTCCCCGCAGGGTCGTCCCCGGCGGCGGCTGGTGCTCTCTCTCTTCTGTC
ATCGGCCAGAGGCAGCGTGGCGGGCGCCAGCGGGCCCTGGCTGGTCACGGCGGCGCGGGAG
GACATCCCGGCGCGCTCCAGCGCGGCGCTCGACGACCTTATCGCCGAGAACCGCGCCGCCGC
GCTCCTCGCGCGGCAGTACTTCGTCTCCGACCGCTCGCCGGCGCCCAGACGGGATTTCGTCG
CCTCTTCGACGGCGGGTTGCTCCCTCCAGCAGCAGCAGCACCCGCCCGACCACGACCACACA
GCCGGCTGGCACCCGCACCCCCACCCCTACAGTGGCGGTGGCGGTGGCAGCTGTGGTGGTCG
GTACCAGTACCACCACGTGGCGCCGCCGAGCGCGTGGCACGCGACGACGCTGGACCTTATGC
AGCCGACAGCAAGCACCACCGCCGCTCCCGCCACCGCCGGCGCAGCGTTGTGGCCAGCAGTG
CCGGAGAGGGCGGGCGCGAGGTCGTCTCGCTCTCGCAGCGAGACCAAGGATGGCGACGCGT
CGGGATGTAGCGGCTTGGACGCGTGGGCGCCCACTGGTGGAGCTCGTGTGCTGTGA

>JL49_GRMZM2G028643
ATGGCAAGCCGCATCGCATCGTTCCCCTCCTTCCTCGCCTTTGTCGTCGTCACCGTCGCCACGT
TATTCCCCTCCTCTTCCTCCCAGCAGCAGCCCGCCGCCGCCGTGCCACAGCCCAGAGGGTTCT
ATATAAGCTGCGGGTCGGACAAGGACGTGCAGGTAGGGAGCATCAAGTGGGCCAAGGATGAG
GGCTTCACCGCCGTCGGGAACGCCTCCGCCATCAACAAGCCCCACCTCCTCCCCCTGCTCGCC
GCGCTGCGCTACTTCCCGGACGCCACCGCGCGGAAGTACTGCTACCAGTTGCCCGTCGTCAAG
GGCACGCGCTACCTTGTCCGCACCACCTACTTCTACGGCGGCTTCGACGGGGGCAAGGAACC
GCCCGTGTTCGACCAGATCGTGGACGGCACCCTGTGGAGCGCCGTCAACACCACGGACAACTA
CCGGCGCGGCATGTCCACCTACTTCGAGATGGTGGCGCAGGGGCAGGGCAAGACCATGAGCG
TGTGCCTAGCGCGCCGCCCCGACACGGCGTCCAGCCCCTTCATCTCCGCGCTCGAGGTCATC
GACCTCGCCGACTCCATGTACAACACCACGGACTTCAGCAGGTTCACCATGACCACCGTGGTG
CGCAGCCGCTTCGGCAGCAAGGGCGATATCGTCAGCTATCCAGATGACCCGTACAACCGGTAC
TGGGCGCCGTTCGCGGACGCCAACCCCATGGTGGAGAGCCACTCCTCCATCTCGCCCGACGA
CTTCTGGAACCAGCCGCCGGCCAAGGCGCTCAAGGCCGGAATCACAACCAGCCGGGGCAAGA
AGCTCGCCGTGCAGTGGCCGACCACGGAGCTCCCCGCGGCGACCTACTACGTGGCGCTCTAC
TTCCAGGACCCGCGCACGGCGAGCCCCTTCAGCTGGCGCGTCTTCGACGTCGCGGTCAACGG
CAACGACTTCTTCCGGGGGCTCAACGCCTCCGCGGCCGGCGTCATGGTCTACTCCAGCATGAT
GCAGCTGTCCGGGAAGACGGAGATCTTGCTCACGCCCAACGAGACGTCCCCCGTCGGCCCGC
TCATCAACGCCGGAGAGATCTACCAGATCGTCCCGCTCGGCGGCAGGACCGCCACAAGAGAC
GTGGTTGCAATGGAAGACCTGGCAAGCAGCCTCAAGAACCTGCCTCCGGACTGGGCTGGAGAC
CCTTGTCTACCACAGCAACATTCCTGGACTGGGGTTGAATGCTCCCAGGGATCACCCGTGCGC
GTCTTGTCACTGGATCTAAAAAACCATGGTCTTTCAGGATCGCTACCCGACAGCATTGTACACTT
GACAGGGATGAAAACCATCTATCTCAGTGGTAACAATCTCTCAGGCCCCATACCTGACTTGAGC
AGCATGCACGCCCTGACTGCAGTGCGCCTTGATAGCAATCAGTTCAGTGGAACGATCAACCCAT
CAATGGAAAAACTAGCCAATCTCAAGGAGCTACACCTGAACAACAACAATCTCACGGGCAAAAT
ACCAGATGGTCTGAAAAACAAAGCTGGACTTGATTTGAGAACTGAGGGGAACAAATTTGAATGA

>JL50_GRMZM2G163724
ATGCCACGGCTTCACCTCACCTCCACCGCCATGGCCGGCACCAATGGCGCCCCTCGCCTTCTC
CGCCTCCCTACCACTGCCACTACCAGTACCAGCTCGACGCCACTGCTGCGACTACTAGCGCTA
CTACAGCTACTATGCCTCGCCTCGGCGCTTAACCAAGACGGCATACTGCTGCTGTCCTTCAAGC
AATCCCTCGCCTCCGACCCCCTCGGCTCCCTCTCCGGCTGGGGGTACGCCGACGTCACGCCCT
GCGCGTGGAACGGCGTCGTCTGCTCGCCGGACTCGCGAGTCGTCAGCGTCGTCCTGCCCAAC
GCGCAGCTCGTCGGCCCCGTCGCCAGGGAGCTCGCCCTCATCGAGAACCTCCGCCACCTCGA
CCTCTCCGGCAACGCGCTCACCGGGACCATCCCGCCCGATCTGCTCCGCGCGCCGGAGCTCC
GCGTGCTCTCGCTCGCTGGCAACGGCATTACCGGCGGCCTGCCGGAGCAGGTCGGCCAGCTG
CGCAGCCTCCGCGCGCTCAACCTAGCCGGCAACGCTCTCTCCGGGGCCGTCCCGCAGAACCT
CACCCTGCTGCCCAACCTCACCGCTGTCTCGCTCGCCAACAACTTCTTCTCCGGCACGCTCCCC
CGCGGGGGCTTCCCGGCGCTGCAGGTGCTCGACGTCAGCGCCAACCTGCTGAACGGCACCCT
GCCGTCGGACTTCGGCGGCGCCGCGCTTCGGTACGTCAACCTGTCCTCCAACGGCATCGCCG
GCGCCATACCGTCGGACATGGCCTCGCGTCTGCCGGCCAATGTCACCATCGACCTGTCCTACA
ACAACCTCACCGGCGCCATCCCGTCGGTGCCGCCGTTCTCGGCGCAGAGGCCCACGGCGTTC
GAGGGCAACGCCGAGCTCTGTGGAAGGCCCCTCGACAGCCTCTGCGGCTTCACGTCCTCCTCC
TCTGCTGCCGCGGAGCCCCAGCCCCCGAACGCTACCGCGAAGTCGCCGCCGGCCATCGCGGC
GATACCCAGGGACCCTACCGAGGCGCTTCCTGGCGATGGTTCCAGCAGTGCCGCAGGCGCGT

CGGCGTCCGGGGAGCAGCGCGGCAGGATGCGGCTTGCTACCATCGTCGCCATCGCCGCCGGT
GACGTGGCTGGCATCGCCGTCCTGTTCGTTGTGGTCATGTACGTTTACCAGGTGAGGAGGAAG
AGGCAGCGTCAGGAGGTGGCGGCGAAGCAGAGGACGGGCGTCGTGTTCAAGAAGCCGGATCC
CGACGAGTCTCCGGACGCTGTCAGCCGGAGCCTTTCCTGCTGCCTGCGCAAGAAGGCCGGCG
ACGACAGCGACTACTCGGAGGAAGTCACGGACACGTCGCCGGCCTCCTTCGCAGATCACAAGA
ACGGCAAGGCGGCCGGAGCTGAAGCGGCGAGCAATAAGAAGATGGGCGGGGACGGCGCGGT
GCTAGTGACGGTGGACGGCGGCGCGGAGCTGGAGCTGGAGACGCTGCTGAAGGCCTCGGCG
TACATCCTTGGCGCCGCCGGCAGCAGCATCGTGTACAAGGCCGTTCTGGCCGACGGCGCGGC
GCTGGCGGTGCGCCGGATCGGCAGCGAGTGTTCGGGGGTCCGGCGCTTCGGCGAGCTGGAC
GCGCACATGCGCGCCGTGGCGAAGCTGCGGCACGACAACATCCTCCGCCTGCGCGGCTTCTA
CTGGGGCCCTGACGAGATGCTCATCATCCACGGCTTCGCCATCAACGGCAATCTCGCCAACCA
CTCCGTTAAAAGTACGTTATCTATTCGGAATTCGGTCCATCATCATCACCCACCCCGGGCCGTT
GCCAAAATGACAATAAATGGCCGCATGCAGGGAAGCAGGGGTCGTCGCCGATCAACCTCGGGT
GGAGCGCGCGGCTCCGCATCGCGCGCGGCGTGGCGAGAGGCCTGGCGTACCTCCACGACAA
GAAGTGGGTGCACGGCAACGTGAAGCCAAGCAACATCCTGCTGGACGCGGACATGGAGCCGC
TGCTGGCGGACCTCGGCGTGGACCGGCTGGTCCGCAGCGCGGACGGCGGCGGGCTGACGAA
GCCGTCGTCGGCGGCGCTGGCGGGTCGGTTCGGCGGGAGCAAACGTTCGGCCAAGAGCCTC
CCCGACCTGTCGCCGCCGCCCAGCCACGTAGGCGGCACGGCCGCGCAACCGGCGAGCCCGG
CCGTCGACACGGCGGCGCACTACCGGGCGCCGGAGGCCGTGAGGAGCCCCAGGGCGAGCGG
CAAGTGGGACGTGTACTCGTTCGGCGTGCTCCTGCTGGAGCTGGTGGCCGGGCGCGCGCTGA
CGAGCCTGGAGCTGTGCCAGTGCGCTGCGGCGGAGGGGAAGGCGCAGGCGCAGGCGCTCGG
GGTGGTGGACCCCACGCTGCGCCGCGAGGTGGAAGGGCGGGAGGAGGAGGAGGAAGCGGTG
GCGAGCTGCCTCCGGCTGGGCGCCGCGTGCTGCGCCATGGTGCCGAGCAAGAGGCCGTCCAT
CAGGGACGCGCTGCAGGCCGTGGAGAGGATGATACCTGCTGCGGTTGCTTCGTCGTCAACATC
CACGGCAGCATCACACCGTCATTGA

>JL51_GRMZM2G178102
ATGAAAGAGAGAGAGAGAGTGACCAGAAAACATCTATTGCTGCTGCAGAGCAGCAGTACTGTAC
TGCTATTAAACAAGCCCAGAGCTCTGCTGCTACTGTCCTCTCCTCCTCCTCACTCCACCACTGC
CCACTCCCATCCCACGCGAGCACAACCACCACCTACTCCCTACTCCCTACACTACTCCCCCCGC
AAGCACGCCCAGCGCCATGGCCATGGCGGCAGCGCGCACGGGGGCCTGCTACTCCGCCTCCG
CTTCCTCTCCTCTCCTGCACTACTAGCGGGTCCGCTGGCGACGATGGCGGCGGTGGCGAGCA
GGGAGAGGCTCTCCCCGGGCGCCGCGCCGCAGGTGGACGCGGGCAAGTACGTGCGGTACAC
GCCGGAGCAGGTGGAGGCACTGGAGCGCGTCTACAGCGAGTGCCCCAAGCCCAGCTCGCTGC
GCCGGCAGCACCTCATCAGGGACTGCCCCATCCTCCGCAACATCGAGCCCAAGCAGATCAAGG
TCTGGTTCCAGAACCGCAGATGCCGGGAGAAGCAGCGCAAGGAGTCCTCTCGTCTACAAACCG
TGAACCGGAAGCTGAGTGCGATGAACAAGCTGTTGATGGAGGAGAATGACCGGCTGCAGAAGC
AGGTTTCCCGTCTCGTCTTTGACAACGGGTATATGAAGAATCGACTCCACAGTCCTTCTGTAGC
CACCACCGACACAAGCTGTGAGTCTGTGGTGACAAGTGGTCAGCACAACCAACAGCAAAACCC
AGCAGTTCTGCATCCTCCACAAAGGGATGCGAACAACCCAGCAGGTCTACTCGCTATTGCTGAG
GAGACATTGGCAGAGTTCATGTCCAAGGCGACTGGAACTGCTGTCAACTGGGTGCAAATGGTT
GGGATGAAGCCTGGTCCGGATTCCGTTGGAATCATCGCTGTTTCGCACAACTGCAGCGGCGTA
GCAGCACGAGCTTGCGGCCTTGTGAGCCTTGAGCCCACAAAGGTTGCTGAGATCCTTAAGGAT
CGTGCATCGTGGTATCGCGATTGTCGGCATGTTGATGTCCTCCATGTTATCCCTACGGGTAACG
GTGGAACGATTGAGCTGATCTATATGCAGACTTATGCACTGACAACTCTCGCGGAACCACGAGA
CTTTTGGACGCTCCGATACACTAGTGGTCTTGACGATGGCAGTCTTGTGATCTGCGAAAGGTCA
TTGACTCAGTCCACTGGAGGTCCTTCTGGACCTAACACTCCAAATTTTATCAGAGCTGAGGTGCT
TCCTAGTGGTTATCTGATTCGACCCTGTGATGGGGGAGGTTCCATGATTTACATTGTGGATCATG
TTAATCTGAATGCTTGTAGTGTGCCTGAGGTTCTTCGGCCACTCTATGAATCCCCTAAGATACTG
GCACAGAAGATGACTGCGGCGGCGTTGCGACACATTAGGCAAATTGCACATGAGTCAAGTGGT
GAAATGCCCTATGGTGCTGGGCGACAGCCGGCTGTTCTCAGAACTTTCAGTCAAAGGCTCAGC
AGAGGCTTCAATGATGCTGTGAGTGGCTTTCCAGATGATGGCTGGTCTTGTTTGTTGAACACCG
ATGGTGCTGAGGATATTACAGTTACAATCAACTCATCTCCAAATAAACTTGTTGGGTCTCATGTC
AGCGCTTCCCCATTATTTTCTGCTATGGGGGGTGGAATCATGTGCGCAAAAGCATCGATGCTGC
TGCAGAATGTGCCACCTGCTCTACTTGTGCGATTTTTGAGGGAGCATCGGTCTGAATGGGCTGA
TCCTGGCATCGATGCTTATTCTGTCGCCTCTTTGAGGGCCAACCCATACACTGTTCCAGGTTTAA
GGGCTGGTGGGTTTATGGGCAACCAGGTTATACTGCCCCTTGCACGCACCTTGGAGCATGAAG
AGTCCTTGGAGGTTATTAGGCTTGAAGGACATGGCTTTAGCCATGAGGAGGTGCTTATGTCCCG

GGATATGTTTCTTCTACAGCTGTGCAGTGGCGTCGACGAGGATGCACCTGGTGCGTGTGCACA
GCTTGTCTTTGCTCCTATTGATGAATCTTTTGCCGACGATGCACCGTTGCTGCCCTCAGGCTTCC
GTGTGATACCGCTTGATGCTAAGACGGATGTGCCAACGGCCACCACACGCACGCTTGACCTTG
CCTCTGCGCTCGAGGTTGGATCAGGTGGAAGCGGCATGCGCGCTTCATGCGATGGTTCCGGCA
CGTGCGCCACGAGATCGGTCCTGACCATCGCCTTCCAGTTCTCGTTCGAGAACCACCTTCGCG
AGAGCGTGGCGGCAATGGCCAAGCAGTACGTCAGGGGCGTGATGGCCTCCGTGCAGAGGGTG
GCCATGGCGATCGCTCCTTCTCGCCTCGGTTCGCGTATCGAACTGAAGCATCCGCCGGGCTCT
CCCGAGGCACTTGCACTAGCTACGTGGATTGGCAGGAGCTACAGGGCGCACACTGGAACGGA
GATCCGCTGGTCGGACACCGAAGGCGCGGACTCTCCACTGATGCTGTTCTGGAAGCACAGCGA
CGCGATACTCTGCTGCTCTCTGAAGCCGCCGGCTTTCACGCTCAAGTTCGCCAACAGCGCCGG
CTTCGACATCCTGGAGACGACGATGGTGAACATCCAGGACCTGCCGCTGGAGGCGGTCCTCGA
CGAGGAGGGCCAGAAGGCCCTGTCCGCGCAGCTCCCCGCGATCATGCAGCAGGGCCTGGCGT
ACCTCCCCGGTGGCGTGTGCAGGTCGAGCATGGGGCGGCAGGCGTCGTACGAGCAGGCAGTG
GCGTGGAAGGTGGTGGGTGACGACGGCGCCCCGCAGTGCCTGGCGCTCATGCTCGCCAACTG
GACCTTCATCTGA

>JL53_GRMZM2G163975
ATGATGGCAGCCTCGTCGTCGTCGTCCTTCAACCTCAGTGATCATCTCGTTCAAGATGACCTCC
CGTGGCCTGCTTCTTCTTCTTCTTCCTCCCAGCTGCCATTTGCTCCTTCTCATCCTCCTCACGGC
GCCATTATTGGAGGCAGCCAGCAATGGTGGCAGCAACCTCAAATGCTAAACTGTGCAGACCGG
CATCCCTCCGACGAGCTTGAGGTCCTCCTCTCAGCACAAGGCAGCCACCACCACCACCACCAT
CCTTCTTCTTCAGTGATCCATCCTCAGCTAAGCTCGCTGCTAATGATGCAAGACCTAGGGTTCCA
ATGGAGCAGCTGCGGCAGCTTCGTGGACACCTCCTCGTCGTCGCCCATGCCACCACCACTGGT
GAACGGCCAGCAGCACGACGGCCACAACAAGATCAAGGAGTGGGACCGCCCGCTCACTGCTA
ATAACTCATCAAGGTCATCGTGTGCGAGCACAGCTATGGCTTGCCGAGATGTCGTCCTCGACGG
CGGCGGCGGCGGCGGCCTCCCTGCCATGGCCGCCGTCGCCGCCGATCTTGACGACGGCACC
GTGCTCCCGGCGAGCGTCAACGTCCCCCGGACGACGACGACGCGCCAGAGGCCATTCCCGGC
GGCGCCGCCACCTCTCCCCGGCGACGCGTTCGAGATACTGGCTTCGTCGAGGCTATGCAAGAC
CCTGCTGCTTAGCCAAGCTTCTTCTTCGGTACTGCTCCATAATGGCACGACGATGCCTTTGTTGA
GATCAGAGCATGTGCCCTATGGCCCTCCTGCTCCTGCTCATCCCCAAGGACCATCCATCGGCAA
CTACAAGCAGTGCATGCAGATGGGCGCGTCTTGTGGAGGCCGCGCGCAGCACGAAGCGGCAT
GTCAAAGCATGGCGGCGGCAGCAGCACGCCCAGCACTGCCAGTGGTGCCGTCGTCGAAGAAG
CCTCGAGTCGAGCAGCGGTCCTCCACCATCGTCCCTTCTTTCAAGGTAAGGAAAGAGAAATTAG
GGGACCGGATCGCTGCTCTTCAGCAGCTCGTCTCACCGTTTGGCAAGACTGATACCGCATCTGT
GCTCATGGAGGCCATCGGTTACATCAAATTTCTTCAAGATCAAGTCGAGACTCTGAGCCGCCCC
TACCTGAAATCCTCCAGAAGTAAGAAGCCCAGGCCGACACCGCGGGGTTCTTCAAACGCGAGC
GCTGGGGAGCTGGAGGAAACGAGGCCTGACCTCCGCAGCAGAGGGCTCTGCCTGGTGCCTCT
CTCGTGCACGTCCTACGTGACCAACGAGAACGGAGTCTGGGTTCCACCGAATTTTAGGGCAAA
CTAA

>JL54_GRMZM2G077219
ATGAGCGCGGGTGCAGCCCGGTCCGTGCTCGTCGTCGTCTTCTTCGCCCTGCCTCTTCTCGTC
TCCATGACCCACGGCGGCCACCAGCAGCCGGCGCATGTCGGAGCGGACCCGCGCTCACCACC
GATCGATGGCGGCGAGCACGCAGAGCAGCGGCAGCAGCTTAGCAGGCTGGGCTCCAGGCCG
CCGTGCTGCGACCGCAAGTGCGGCGCGTGCGCGCCGTGCAAGGCCGTGCAGGTGCGCGCCG
GCGCCGGCGCCGCGGCGGAGCGCCGCCTCCGCCCGCGCTGCGCCAACTACGAGCCCGTCGG
GTGGAAGTGCAGGTGCGGCGCCGCCGTCTTCGACCCTTGA

>JL55_GRMZM2G082586
ATGGCACTGGAGGCCGTGGTGCTTTCCCAGCAAGGCAGTCGCTTTGGGTGTGGTGCCATGGCA
GCAGGAGGGCCTTGGAGTGGCCTGTTTTTTAGTGGAATGGAGGGCGTGGTCGAGATCGGTAGT
GGCGCTGGCGGCGGCCGGGACGTGGCCGGGTGCTCGCCGCCACAGCTCCTGCAGGAGCCGG
CTGCCCGCGCGCTGCCTGTGGCGTCGTCCGTGGGGAGCGCGAGTGGCGGCGCCGGTCAGGA
TGCCCCGGCGGCACTTGCTGTGACCGCGACAGCGGCGGCCGGAACCGGAAGAAGGAAGCGG
CGAAGGATGAGGCCACTGAAGAACGAGGAGGAAGTGGAGAGCCAGCGCATGGTCCACATTGC
CGTGGAGCGCAACCGGCGCAAGCAGATGAACGAGTACCTCGCCGCGCTCCGCTCCCTCATGC
CGCCCGCCTACACGCAACGGGGCGATCAGGCGTCCATCGTCGGCGGCGCGATCAACTTCGTC
AAGGAGCTGGAGCAGCTGCTCCAGTCGCTGGAGGCGCAGCAGAGGCGTTCCTCCCGGCGGCA

GGGCCCCGCGTGCGCCGTCGATCCGGACGACGCAGGGCCGTTCGCGGACTTCTTCACCTTCC
CGCAGTACTCAATGTGCGCTGCCGCGGCGGCCCCATCAGAGAACCCCGGCGCCGACGCCGAC
GCCGAGCAGGAGGCGTCCGGGTCGAAGCCGTCGGGCGTGGCCGACGTGGAGGCGACCATGG
TGGAGAGCCACGCCAACCTGCGGGTGCTGTCCCGGCGCCGGCCGAGGCAGCTGCTGCGGCT
GGTGCTGGGGCTCCAGGGCCACCGCCTCACCGTGCTCCACCTCAACATGAGCAGCGCCGGCC
ACATGGTGCTCTACTCGTTCAGCCTCAAGGTGGAGGATGATTGCCCGCTCACCTCAGTGGATGA
GATTGCAGGTGCGGCTCATCAGATCGTCGAGAAGATCAACGAAGAACAAGGCGGCGGCAGCTT
AGAGTAA

>JL56_GRMZM2G171365
ATGGTGCGGGGCAAGACGCAGATGAAGCGGATAGAGAACCCGACCAGCCGCCAGGTCACCTT
CTCCAAGCGCCGCAACGGCCTGCTCAAGAAGGCGTTCGAGCTCTCCGTCCTCTGCGACGCCGA
GGTCGCCCTCGTCGTCTTCTCCCCGCGCGGCAAGCTCTACGAATTCGCCAGCGGAAGTGCGCA
GAAAACGATTGAACGTTATAGAACATACACAAAGGATAATGTCAGCAACAAGACAGTGCAGCAG
GATATTGAGCGAGTAAAAGCTGATGCGGATGGCCTGTCAAAGAGACTCGAAGCACTTGAAGCTT
ACAAAAGGAAACTTTTGGGTGAGAGGTTGGAAGACTGCTCCATTGAAGAGCTGCACAGTTTGGA
AGTCAAGCTTGAGAAGAGCCTGCATTGCATCAGGGGAAGAAAGACTGAGCTGCTGGAGGAGCA
AGTCCGTAAGCTGAAGCAGAAGGAGATGAGTCTGCGCAAGAGCAACGAAGATTTGCGTGAAAA
GTGCAAGAAGCAGCCGCCTGTGCCGATGGCTTCGGCGCCGCCTCGTGCGCCGGCAGTCGACA
ACGTGGAGGACGGTCACCGGGAGCCGAAGGACGACGGGATGGACGTGGAGACGGAGCTGTA
CATAGGATTGCCCGGCAGAGACTACCGCTCAAGCAAAGACAAGGCTGCAGTGGCGGTCAGGTC
AGGCTAG

>JL57_GRMZM2G098813
ATGTTACTTGCTCTTGTTCGATCATACCGCAAACTCGCAGCGCTGTCCGACGAGCGCGACGCCG
CGGCCAGCGGCGGCGGCATGGCAGAAGGCGAGGCCGGCAGGAGGATGGTGACGACGACCGC
CGGCAAGAAGGGCAAGAAAGGGGTCGTTGGCACGAGGAAGGGCAAGAAGGCGAGGAGGAAG
AAGGAGCTGAGGCCGCTGAACGTGCTGGACGACGAGAACGACGGGGACGAGTACGGCGGCG
GGTCGGAGTCGACCGAGTCGTCCGCGGGAGGCTCCGGGGAGAGGCAGCGGGAGCACCCGTT
CGTGGTCACCGAGCCCGGCGAGGTGGCGAGGGCCAAGAAGAACGGGCTCGACTACCTCTTCC
ACCTGTACGAGCAGTGCCGCGTCTTCCTGCTCCAGGTGCAGTCCATCGCTAAGCTGGGCGGCC
ACAAATCCCCTACCAAGGTGACCAACCAGGTGTTCCGGTACGCGAACAAGTGCGGGGCGAGCT
ACATCAACAAGCCCAAGATGCGGCACTACGTGCACTGCTACGCGCTGCACTGCCTGGACGAGG
AGGCCTCCAACGCGCTGCGCCGGGCGTACAAGTCCCGCGGCGAGAACGTGGGCGCCTGGAG
GCAGGCCTGCTACGCGCCGCTCGTCGAGATCGCCGCGCGCCACGGCTTCGACATTGACGCCG
TCTTCGCCGCGCACCCGCGCCTCGCCGTCTGGTACGTGCCCACCAGGCTGCGCCAGCTCTGC
CACCAGGCGCGGGGGAGCCACGCCCACGCTGCCGCCGGACTCCCGCCGCCCCCGATGTTCTA
G

>JL58_GRMZM2G039074
ATGGCTACTACTACTGCCGCAGCCCCCGACCTCTCGCTGCATATCAGCCCTCCCTCGCCGCCC
GATGATGCTAGGAGCAGTGACGTGCTCTTCTCCAAGCAGACGTTGTGCCTCGGACTCGTTCAGA
CGACGACGGCGGCGGCGCATGATCAGGATGGCCAATGCGACGACGTCCAGCAGCAGCAGCGG
CAGCGACTGCACCAGCAGCCTAGCCAGATCCAGAGGTTCAAGAAAACCTCGTCATCGTCGGCG
GCGTTGTTATCTGCCGGAGGAGGCACGACGAGATCTGGGAACGGCAACAGCGGGGGCGGGAA
GAGGAGCTCCAGAGCGCCGAGGATGAGGTGGACCACGGCTCTTCATGCACACTTCGTCCATGC
CGTAGAGCTCCTTGGCGGCCATGAGAGAGCAACGCCAAAGTCAGTGCTGGAGCTGATGAACGT
TAAGGATCTCACATTGGCTCATGTTAAGAGCCACTTGCAGATGTATCGAACAGTGAAGGGAACG
ACGGCAGACAGATCATGTGCTGCAGGCCATGTCCAGATGAGAGACATGGCTTTCTGCACAGG
GGTTGTGAACTGAATGGCTTCGAAGCGTTTAGCAACGGTACTTCTAATGCTACCGCCAATAATA
GAATGCAGGCAGCTGCCGGGTCGCCGGCAGGGAAGCATGCTGAGGTAGCCTGGTTCTTGCGG
CCACCCTTGGCACACCAGAGTAGTGGTGGTGGTGGTGGTGGTGGGGCGGTGCCGCTTCCGCT
GCCGCTGCCCTGCCCCTACCTCATGTCTGCCCACCACAACCGCTACTTGCTCTCACAGAATCAG
GGATGGAGGACCAGAGGCGCGCATCAGCAAGACGCTGCCGCCCCCCCGCCGCTCGTCCTAGG
CCAGCAGGACAGTGCGCGGCGTCTGCATCCAGGCTTCCCCGACACGGCCATCAGCAGCAGCA
GACCGTCATCCTGGAGCGCAGGCGTGGCAAGCCGCTGGTCGTCGTCGACGGCGGCGGCGCCT
TCGCCGTCAAGTGGCAGGAGTAGCATCAGCAGCGGCACGGAACAGCCAGCAGCATGCAGCTG
GACGATGATGACGACGAACAAGCAGCAGCAGCAGCCGCCGCCGTCGACGTCATCATCATCAAG

GGTCGTCGCGGTACCGAACCTGGAGATGAGCCTGGGGAGGCAGGGGTGGCAACACGACCTGC
AGGATCATCATCCGCAGCAGCAGCAGCAGCAGCGCAGCGGGGAGTCCACGGCGCCAAAAGAG
CTGACCCCCGGCCTCCTCAAGTGCTTGTGA

>JL59_GRMZM2G139324
ATGATGATGATGATGATGGACAGGCAGCTCGTCAGGCAATGCGACATGGAGGTCATGAGGATG
GCCATGCTCAAGCACGAAGAGACCTTCCGGCAGCAGGTTCACGAGCTGCACCGCCTGTACCGC
ATACAGAGGGAGCTGATGAGCGACCTAATAACGACCCGATACGACGACGAGCCGCTGCTGCTG
ACGACCGCCGGCCGGCGCAGCAAGCAGCCGCGCCGGGCGCTGGACCTGCAGCTGCCAGCCG
ACGAGTACATCGTCAGCGCCGACGAGGAGTCGGACGCCGCCGGCGCGGAGCTGGAGCTGAC
GCTGGCCGTCGGCGGGCGCTGCGCAGCCGGCGGCGGCCGGCGCAAGAGCAACAGGCGCCG
GAGGCAATTAGCAGAGCGGCAGCAGCGCGACAACAACGACGCCGCCGCCGCCGGCGGCGGC
TCCTCGCCGTTCGGGTCCGACTGCTCCGGCGCGAGCGCCCTGTCGTCGCCGCCGCCTTCGTC
GGCCGAGTACTACTCCGACGACGGGCCGGCGGCGGCGGCGTGCCAGAGGGCCGTTGCGTTC
GACCTTGGAGGAGAAGGCGACGGCGTGATGAGGCAGCAGCACGCGCCGTGGCTGGCGCAGT
GCCAGCAGTACCTCAGCCTGAGGATGACATGA

>JL60_GRMZM2G061314
ATGTCATACCCCGGCTCCTCTGCTTCTCGGTTCCCGCCGTTCCATGTCCGCCACAAGGTGCCC
GCCACAAGGGCAGACCGGCGGCAGAGGTTGTACCGATCGCTCTGGCCCACGTCGCCGGCCGG
ACCATCAACCACCGCGCGGAGCATCAAGCAGCTGCCATTCCAAAGCCCGTTGAAGCATCGAAG
GATTTCCAGCCGTGAGCGTGTGCGGTGGCGGCGCGGCCGGGTCGCCGGGATGGGCAAGATG
AACTGCTTCTCCGGCCTGCTCAGCGGCAAGAGCAAGGCATTCAAGGGTGGCAACAAGGGCGCT
TACGCCAAGAAGGCGGCCAGTGGTGGCAACGATTGCCCCAAGGTGAAGCCGGTAGAGTTCGTA
GACATGGCGGGCACTGCTGTCGACGACTTCGTCGGCAGACGCGGCGGAGGCAGCGTTCATGC
GTCCGCATGCAGCAGTAGCTTTGTCGTTCACGCAGCCACCGAGCTGCCACGCCATGCCACTGC
CGAGAACGGTGAAGACGAGGCCGCGGTTAACAAGGACTCATCCTCCGCCGGCGCTGGCTCGC
CCGGTTACAGCATTGGCCGCATGTCGCCAACGGCATCGCCGAAGCTGACGCGCTCCTGCTCCA
ACATGGAGACGGCGAGGTCTGTCCCCCCGCCAGGGTCCGACTCCGACCTGCCGGCGAGGTCA
CGCTCCCACAACGACCTGCTGAAGAACCTGCCCCCCGGAAGGCCGACGACCGCCCGCAGCGG
CTTGAGCCCGACGGCGTCCTTCAGGTCCTCGTGCTCCGCCGACCGCGTCCTGCTGAAGAAACG
GTCGTCGCGGCAGGTGCTCCCGTCCCGGAGCCGGAAGCTGTGGTGGCAGACGTTCCTCTGGA
GCCACCGCAACCTGCACCGGCCGGGCGCGAGCGCGACCACTCCCACGCTGCCGTCTGCGTGG
AAAGACGGCCGTCCCGTGCACCACGGGTACACGTCCGACACGCTGGGCACTGCCACCGCGGA
CGCCAAGAACAAGGGCGTCGTCGCGGTGGAGGAGGACCCCGTCCCGAACCAGTGGGTCGCGT
TCTCCGCCGAGGCGTCTCCACTGGACCGGGTCAGCGCGTGGGTGAACAGCCTCGGGGACGCC
TCGTTCCACGCCGTCGACGAGGAGGACGACGCCACGCTGCACGGCGCGCGACCGCGGCCCC
GACCGAGACGCTCTGAGATCGTGGAGCTGTGGACGGCCGGCGGCAAGAGGCAGCCACAGGC
GAAGCGGCGCGCCGCGGACGAGGCCGCCCCCGCCCAGGCGAGCGGCGTCGTGCACACGCTC
AACGCCTTCTCCTCCGTCGCGCACATCGCCGGCATGGGGCTCCGGACCGTCCCGATGATCGCC
GCCTTCTCGACCCTCCGCGCCGTCAACCTGTCTGGAAACATGATTGTGCAAATCACCGCTGGAT
CGTTGCCCAAAGGCCTGCACTCACTGGACCTGTCGCGGAACAGCATTGCAGTCATCGAGGGGC
TGCGGGAGCTGACGAGGCTGCGGGTGCTCAACCTCAGCTACAACCGGATCTCAAGGATCGGC
CATGGCCTGTCGAGCTGCACGGCCATCAGGGAGCTGTACCTGGCCGGGAACAAGATCAGCGA
CGTGGAGGGGCTCCACCGCCTGCTGAAGCTGGCCGTCCTGGACGTGAGCTTCAACAAGATCAG
CACGGCCAAGTCCCTGGGCCAGCTCGTGGCCAACTACGGCTCGCTGCGGGCGATCAGCCTGC
TGGGCAACCCGGTGCAGGCGAACACCGGCGAGGACACGCTCCGCAAGGCGGTGTCGGGCCT
GCTCCCGCGGATCGACTACCTGAACAAGCAGGCCGTGAAGCCGCAGCGCGCGCGCGAGGTGG
CCAAGGACAGCGTCGCGCAGGCGGCGCTCGGGAACAACGCCGGGGGCAGCTCGCGGAGGCG
GCTGGCGCGCCGCCTCAGCCAGAGCCCCGGCTCGTCGTCGGGCAAGGGCCGGGGCCGGGGC
GGCAGGGAGGGGAACGGCAGCCGCAGGGGCAGCAGGAGCAGGTTCGTGGCCAGGCCCCAGA
GCTCGAGCTACCAAGGAGATGAGGCAAAACATGACAACGGTGTTGGGCTTGAAGAGGGAGAAG
AAACAAGTCCGTGCGTTTAA

>JL61_GRMZM2G151955
ATGCACTGCCGCCCCTGCCCGTCCCAGCTCTCGCTCGTCTTTCTCCTCCTCCTCTCGCTCGCAC
CCATCATTTCGGCACTCCCCTTCCCGGAGCCAGCTCTTCCGATGGCGTGCAGCGGCCTCCGCC
TCTCGCTCCCCGCCGCCGTCCTCGCCCTCCTCCTCCCCTCCGCGCTCGCTTCCGAGCCCC

TCAACCCCGAAGTGCAGGCGTTGATCGCCATCAGGCAGGGGCTGGTCGACCCGCACGGCGTG
CTCAGGAGCTGGGACCAGGACTCGGTCGACCCCTGCAGCTGGGCCATGATCACCTGCTCCCC
GCAGAACCTCGTCATCGGCCTGGGAGTGCCGAGCCAGGGCTTGTCGGGGACTCTGTCCGGGA
GGATCGCCAACCTCACCCACCTCGAGCAAGTGCTGCTGCAGAACAACAACATCACGGGGCGGC
TGCCGCCGGAGCTGGGCGCGCTGCCGAGGCTGCAGACGCTGGACCTCTCCAACAACCGCTTC
TCCGGCCGCGTGCCCAACACGCTGGGCCGCATCACCACCCTCCGATACCTGAGGCTGAACAAC
AACAGCTTGTCCGGGCCGTTCCCCGCGTCGCTGGCCAAGATCCCGCAGCTCTCCTTCCTGGAC
CTGTCCTTCAACAACCTCACTGGCCCCGTCCCGCTCTTCCCGACACGGACCTTCAACGTCGTGG
GCAACCCGATGATATGTGGGAGCAACGCCGGCGCAGGGGAGTGCGCCGCCGCGCTGCCGCC
GGTCACCGTACCCTTCCCGCTGGAATCCACTCCGGGCGGCAGCAGGACAGGGACAGGGGCGG
CGGCAGCGGGCAGGTCAAAGGCTGCTGGCGCGCGGCTGCCGATCGGCGTAGGGACAAGCCT
GGGCGCCTCCTCCCTTGTGCTCTTCGCCGTGTCCTGCTTCCTCTGGCGGCGAAAGCGCCGGCA
CACGGGGGGGCGGCCCTCCTCCGTTCTCGGCATCATCCACGAGCGGGGCGGCTGCGACCTGG
AGGACGGCGGCGGCGGTGGCGTGGTGGCGGCGGCGGCGCGGCTGGGGAACGTGCGCCAGT
TCGGGCTGCGGGAGCTGCAGGCGGCCACGGACGGGTTCAGCGCCAAGAACATCCTGGGCAAG
GGCGGGTTCGGGAACGTGTACCGCGGCCGCCTCGCCGACGGCACCACGGTGGCCGTGAAGC
GGCTCAAGGACCCCAGCGCGTCGGGCGAGGCCCAGTTCCGCACGGAGGTGGAGATGATCAGC
CTCGCCGTGCACCGCCACCTGCTGCGCCTGGTCGGCTTCTGCGCCGCCTCGGGCGAGCGCCT
CCTCGTCTACCCCTACATGCCCAACGGCAGCGTCGCGTCGCGGCTGCGAGGGAAGCCGGCGC
TGGACTGGGCGACGCGGAAGCGGATCGCGGTGGGCGCCGCGCGCGGGCTGCTGTACCTGCA
CGAGCAGTGCGACCCCAAGATCATCCACCGCGACGTCAAGGCCGCCAACGTGCTGCTGGACG
AGCACCACGAGGCCGTCGTGGGCGACCTGGGCCTGGCCAAGCTGCTGGACCACGGCGACTCG
CACGTCACCACGGCGGTGCGCGGCACCGTGGGCCACATCGCGCCCGAGTACCTCTCCACGGG
GCAGTCGTCGGAGAAGACGGACGTCTTCGGCTTCGGGATCCTGCTGCTGGAGCTCGTCACGG
GCCAGCGCGCGCTCCAGCTCGGCAAGGCCTCCGGCGCCCTGCACAGCCAGAAGGGCGTCATG
CTGGACTGGGTGAGGAAGGTGCATCAAGAGAAGATGCTTGACCTGCTGGTGGACCAGGACCTG
GGCCCACACTACGACAGGATCGAGGTGGCCGAGATGGTGCAGGTGGCGCTCCTCTGCACGCA
GTTCCAGCCGTCGCACCGGCCCAAGATGTCCGAGGTGGTCCGGATGCTGGAGGGAGACGGCC
TGGCCGAGAAATGGGAGGCCACGAACCGGCCGGGCGTGGCCGCGGGGGCGCCGTGCCACGA
CGCGCTCGGCTACGACCACCGCAACGACTCCAACGGCTCCGTCTTCTTCAACGACTTCCACGA
CAACGACAGCAGCCTCAGCAGCGACGAAGCGCGGTCGATCGACATGGTGGAGGAGATGGAGC
TGTCCGGACCAAGGTAG

>JL62_GRMZM2G159953
ATGGCCGCCGCTCACCTCCGGTTATTGCCGCTCTTGCTGCTAACGCTGACAGCGGCGGCCAAG
AACGTCACCCTCGACTCTGCCATACTATCCTTCGCGGACCTCACGCTGCTGGGGGACTCCTTCC
TCCGCAACGGCTCCGTCTGGCTCACCCGGGATACCGACGTGCCCTCCTCCAGCGCCGGCACC
GTGCTCTGCACCCAGCCCGTCGCGTTCCGGGGCCCCGACGCCACGGCCTCCAATGCCACCGC
CGCCGTCGCATCGTTCGCGGCCAGGTTCTCCTTCGTCATCGCCAACCCCAACGCGGGCGCCG
CGGGGGGCGACGGCATCGCGTTCTTCATCTCCCCCGACCGCACCACGCTCGGCGCCACCGGC
GGCTACCTCGGCCTCTTCAACTCCTCCAACTCCGCCGCAGCCAAGAACGGCTCCGCGTCCGCC
ACCATTGTCGCCGTCGAGTTCGACACCATGGCCAACCCGGAGTTCGCCGACCCCAGCGACAAC
CACGTCGGCCTGGACCTCGGCTCGCCGGTCTCCGTCACCGCGGCCGACCTCGCCGCATCTGG
GGTCGACCTCAAGAGCGGCAACGTCACGACGGCGTGGATCGACTACCGCAGCGCGGACCGCC
GCCTGGAGGTGTTCCTGAGCAGCTACGCCGTGGCCGCCAAGCCGAAGCGGCCGGTCCTCTCC
GTCGCTGTCGACCTCTCGCCGTACATCAAGGAGGCCATGTACGTCGGCTTCTCGGCGTCCACA
GAGGGGAGCACGCAGCAGCACACCATCAAAGGGTGGACCTTCCAGACGTTCGGCTTCCCGCC
CACGGCCAACTCAACCTTTGAATCCAATGCCACCAGCAACTCTTCAGAGCCGGCCGCGCCAGC
GCCACCGCCAGCCTCCAACGCCCCCAACAGCCACAAGAGGAGGGTCGGGATCGCGCTTGGAA
TCCTCGGCCCCGTTGCGCTTGCCCTCGCCTTCATCTTCTTCGCCTGGGTCTCCATCAAGAAGCT
CATAGAACTCACCTCCCGGACCGACGCCGCTTTCTCGACCGAGCTGGTCAAGGGCCCGAGGAA
GTTCAGCTACAGGGAGCTGAGCGCGGCCACCAGAGGGTTCCACATGAGCAGGGTCGTCGGCA
AGGGCGCGTTCGGGACTGTGTACAAGGCCGCCATGCCGGGAGCTGCCGCCACCACCTACGCC
GTTAAGAGGTCGACGCAGGCGCACCAGAGCCGGAGCGAGTTCGTCGCCGAGCTATCCGTCAT
CGCGTGCCTCCGGCACAAGAACCTTGTCCAGCTTGAGGGGTGGTGCGAGGAGAAAGGCGAGC
TGCTGCTGGTGTACGAGTACATGCCCAATGGAAGCCTGGACAAGGCCCTCTACGGCGAGCCTT
GCACGTTGTCGTGGCCGCAGAGATACACGGTCGCCGCCGGCATCGCGTCGGTGCTGTCGTAC
CTCCACCAGGAGTGCGAGCAGCGCGTCATCCACCGGGACATCAAGACCAGCAACATCCTGCTG

GACGTCAACCTGAGCCCGCGCCTGGGCGACTTCGGGCTGGCCAGGCTCATGGACCACAACAA
GAGCCCCGTGTCCACGCTCACCGCGGGACCATGGGCTACCTCGCGCCGGAGTACCTTCAGT
CCGGCAAGGCCACCGAGCAGACCGACGTGTTCAGCTACGGCGTGGTGCTCCTCGAGGTGTGC
TGCGGGAGGCGGCCCATCGACAAGGACGAGCGCGGCGGGGGCGGCAGGGGCGGCGGGGGC
AAGAACGTGAACCTGGTGGACTGGGTGTGGCGCCTCCACGGCGAGGACAGGCTCATCGAGGC
CGCGGACGCACGGTTGGCCGGCGAGTTTGACAAGGCCGAGATGCTGCTGCTGCTGCTCGTGG
GGCTGAGCTGCGCCAATCCCAACTGTGAGGAGCGTCCCGCGATGCGGCGGGTGGTGCAGATC
CTCAACCGAGAGGCGGAGCCTGCGCCCGTGCCGCCCAAGAAACCACTGCTGGTGTTCAGCTC
CAGCGCGTCCGCGAAGCTGCAGGAGATCGCCTTCTCCTGTGGCGATGACGTCCGAGGCGGCT
ACTCCGTTGCCAAGCCTGCATCACCAAAGTCCGAGGGAGCCGACATTGAACGCTGA

>JL63_GRMZM2G039934
ATGGCCGCCACCACGGGCACCTCGTCCTCGCCGCCGCAGCTCCCGGTCCACGGCGGCCTCCT
GCTCCTGCTCCCTCTCCTGACCATTACCTCCGCCGCGTCGTCGGCGCCGCTGCCCCTCCTCGC
GCTGCTCTCCCTGAAGTCGTCCCTGCACGACCCGGCCGGCGCACTGCGCGCTTGGACGTACG
CCGCTGCGGCGTCCGCGGGCGCCACGCGGTCGCTGGCGCCGCCGTGGTGCGCGTGGCCGG
GCGTGTCCTGCGACCCGGCCACGGGGGACGTCGCCGGGCTCGACCTCTCCCGCCGCAACCTC
TCCGGCACCGTCTCCGCCACCGCGGCCAGGCTGCTGGCCCGCACGCTGACGTCGCTCAACCT
CAGCGCGAACGCGTTCGCGGGGGAGTTCCCGCCGTCGGTGTTCCTGCTCCGGCGGCTCCAGT
CGCTCGACGTCAGCCACAACTTCTTCAACGGCACGTTCCCCGACGGCGTCGCCGGGCTCGGC
GGCTCGCTCGCCGCGCTCGACGCCTACAGCAACTGCTTCGTCGGCTCACTGCCGCGCGGCCT
CGGCGAGCTCCGCCGGCTGCAGAGTCTCAACCTCGGCGGCAGCTTCTTCAACGGGACCATCC
CAGCCGAGATCGGCCAGCTCCGTTCGCTGCGGTTCCTGCACCTCGCCGGGAACGCGTTAACC
GGTCGGCTCCCTTCGGAGCTCGGCGGCCTCGCGTCGCTCGAGCAGCTCGAGATCGGATACAA
TGCATACGACGGCCGCATTCCCACGGAGCTCGGCAACCTGACGCAGCTCCAGTACCTCGACAT
CGCGGTCGCCAACATGTCCGGCCCGCTGCCACCGGAGCTCGGCAAGCTCGCGCGGCTCGAGA
AGCTCTTCCTGTTCAAGAACCGGCTCGCCGGTGCCATACCGCCGCAGTGGTCCCGCCTCCGCG
CGCTGCAGGCCCTCGACCTGTCTGACAACCTGCTCGCCGGCACCATCCCGGCAGGACTCGGG
GATCTTGGCAATCTCACAATGCTGAACCTCATGAGCAACTTCCTTTCCGGTACAATCCCGAAGG
CGATCGGCGCGCTGCCGAGCCTCGAGGTGCTGCAGCTCTGGAACAACTCGCTCACCGGACGG
CTGCCGGAGTCGCTCGGCGCGAGCGGGCGGCTCGTCCGGGTGGACGTGTCGACCAACTCCCT
GTCCGGCCCGATTCCTTCCGGGATGTGCATCGGCAACCGTCTCGCCCGCCTCATCCTCTTCGA
CAACCAGTTCGACTGGACCATCCCAGCGAGCCTGGCCAACTGCTCGTCGCTCTGCCGAGTCCG
GCTGGAATCTAACCGCCTGTCTGGCGAGATCCCGGTCGGGTTCGGCGCGATCCGTAATCTGAC
ATACCTGGACCTGAGCTCCAACTCACTCACCGGCGGCATTCCTGCTGATCTCGTGGCTTCTCCG
AGCCTCGAGTACATCAACATCTCCGGCAACCCCGTCGGCGGCGCGCTCCCGAACGTGTCGTGG
CAGGCGCCGAACCTGCAAGTGTTCGCAGCGAGCAAGTGCGCCCTGGGCGGCGAGGTCCCGGC
GTTCCGCGCCGCGGGGTGTTCGAACCTGTACCGGCTGGAGCTGGCAGGGAACCATCTCACTG
GTGCCATCCCTAGTGACATCAGCACCTGCAAGCGGCTGGTGAGCTTGAGGCTGCAGCACAATC
AGCTCAGCGGCGAGATCCCAGCAGAGCTCGCCGCCTTGCCGTCGATCACTGAGATCGACCTGT
CCTGGAACGAGCTCAGCGGCGTCGTCCCGCCGGGCTTCGCTAACTGCACCACGTTGGAGACCT
TCGACGTGTCCTTCAACCATTTGGTGACCGCTGGCTCGCCGTCAGCGTCGTCACCAGGCGCCC
GTGAGGGGACCGTTCGGCGCACCGCGGCAATGTGGGTTTCTGCCGTGGCAGTGTCCTTGGCG
GGGATGGTGGCGCTCGTCGTCACCGCTCGCTGGCTGCAGTGGCGCGAGGACGGCACTGGCG
CGCGGGGGGTTGGTAGCAGGGGCGGCGCAGGTGCACGCCCCAACGTGGTCGTCGGACCGTG
GCGGATGACCGCGTTCCAGAGGCTCGACTTCACCGCCGACGACGTGGCGCGGTGCGTTGAGG
GGAGCGACGGCATCATTGGCGCCGGGTCATCGGGGACGGTGTACCGCGCGAAGATGCCGAAC
GGGGAGGTCATCGCGGTGAAGAAGCTGTGGCAACCGTCGGCGCAGAAGGAGGGAGGAGCTCA
GGCGCCGGAGGAGCCACCAAAGCGGAAGGACGAGGCAGACGCGGACGACGGCAACAGGAGC
ATGCTCGCCGAGGTGGAGGTGCTGGGTCACCTCCGGCACCGCAACATCGTCCGGCTGCTGGG
GTGGTGCACCGACGGCGAGGCGACGCTGCTGCTCTACGAGTACATGCCGAACGGCAGCCTGG
ACGAGCTCCTGCACGGCGCCGTGTGCAGGGGCAAGCAGGCGGGGCTGGACTGGGACGCGCG
CCACCGGATCGCCGTGGGCGTCGCGCAGGGCATGAGCTACCTTCACCACGACTGCGTGCCGG
CCGTGGCGCACCGCGACCTCAAGCCTAGCAACATCCTGCTCGACGCAGACATGGAGGCGCGC
GTGGCGGACTTCGGCGTCGCCAAGGCGCTCCAGGGCGCCGCGCCCATGTCCGTCGTCGCCG
GCTCCTACGGCTACATCGCACCAGAGTACACGTACACTCTCCAAGTAGACGAGAAAAGCGACGT
GTACAGCTTCGGCGTGGTGCTGCTGGAGATCCTGATCGGCCGGAGGTCCGTGGAGGCGGAGT
ACGGCGAGGGAAGCAACATCGTGGACTGGACGAGGCGTAAGGTCGCCGCCGGCAACGTGATG

GACGCGGCGGAGTGGGCGGACCAGCAGACCCGCGAGGCGGTGCGGGACGAGATGGCGCTGG
CGCTGCGGGTGGCTCTGCTCTGCACCAGCCGGTGCCCGCAGGAGCGGCCGTCGATGAGGGAC
GTCGTGTCCATGCTGCAGGAGGTCAGGCGCGGCCGGAAGATACTAGCTCCTGGGATGGCCAA
GAAGCAGCCAAAGATTAATTAA

>JL64_GRMZM2G046316
ATGCCACTCCAGGTCCACCCCCTCCTCCTGCTCCTCTTGGTCAGCTCGCTTCCCTCCTGCGCCA
TCTCCTCCGGTTCCTCCTCTTGCGCCGGCCGCGACGACGCGGCCATCGTCGCCGCCGCGTTC
CGGCACGTGCGCAACTTCCGGGCCCCGAGGACGAAAGCATGCCAGCCGGTCAGAGCGCTCCG
CCTCCCGTCGCGGAACCTCACCGGCGCGGTGTCGTGGGCGGCGCTGGCAAACCTGTCCGCGC
TCGCCGCGGTTGACCTCTCGGGCAACGCGCTCCAGGGCGCCATCCCGGGCGGGTTCTGGCGC
GCGCCATCGCTCCGCGCGGTTGACGTCTCCCGCAACCAGCTCGGTGGCGCGCTCCGGGTGGA
GCCCAACCCGCAGCTCCAGTCGCTCAACGTATCCGGCAACCGCTTCACCGGCGTCGACGGGG
TGGAAGGGCTCTCGGGGCTCGTCGCGCTTGACGTGTCAACGAACAGGATCCGGGCGGTGCCG
CGGGGTCTGCGGCGTCTGACACGGTTGAAGCGGCTCGACCTGTCCTCGAACGGGATGCGGGG
GTGGTTCCCCGGCGACTTGCCGCCGCTCGGTGGGGTGCGTTCGTTGAACGTCTCGTACAACAA
GTTCTCCGGCGTGGTGGACACGGGCGCCGTCACGAAGTTCGGCCACTCGGCGTTCGTTCACG
CCGGCAATGCGTCGCTGGTGTTCTCGGGCCACTCGACAGAGCCACGGCGGCCACGGCCATCA
CCTCCTCATGGAAAAAGCAAAAAGAACGGTGGTTCGGCCGGGACGTCGACGGAGAGCAAGGC
GACGAGGAGTAGCAAGAAGAGGAAGCATCTGAGCATCGTGGCGGTGGCGGTGATCTGCGGGG
TGGTGTCCCTGGCCATGCTGCTCTGCCTGGTCGGGTGCGTGGCGTGCGGCGTGCTGAAGTCC
AGGCAGAAGGGAGGAAAAGACGACGACGAGAAGAAGCCACAATGGGGCGAGAAGGGCGAGGA
GGAGAAGGAGGAGGACGTGGTGGTCGCGGCGGCCAGGGGTGCATCGGCAGCGCCCGTGGTG
CTGTTCGAGCGGCCGCTGATGCAGCTCACGCTGGCCAACCTGGCCGCGGCCACGTCGGGCTT
CGGCCGCGAGTCGCAGCTCGCGGAGCGCGGCGGCCGCAGCGGCGCAGCGTACAGGGCCGTG
CTCCCCGGCGACCAGCACGTCGTCGTGCGCGTCGTGGAGGGCGCCATGGCCGGGCTCGGCG
AGGACGACAACCCGGCGGCCGCTGCCACCGCATTCCGGGAGCTCGCGCGCCTCCGCCACCCC
AACATTCTTCCGCTCCTTGGCTACTGCATTGCAGGCAAAGAGAAGCTGCTACTGTACGAGTACA
TGGAGAAGGGCGATCTGCACCGGTGGCTGCACGAGCTGCCAGCGGGTCGGCCGGACATGGAC
GACACGGGCAGCGGCGACATTTGGGAGGCGGCGGAGGTCAAGCGGTCCATCTCCGACTGGCC
GACCCGGCACCGCATCGCGCTGGGGGTCGCCCGGGGCCTGGCGTTCCTGCACCAGGGATGG
GCCGGCGGGTCGGGGCGCGCGGCGGTCGTGCACGGCCACCTGGTGCCGACCAACGTTCTGC
TGTGCGACGACCTGGAGCCCCGGATCTCAGACTTCGGGCACAACAACGACCCGCCGTCGGCG
GCGACGCCCGAGGGCGACGTGTACGGGTTCGGCGTGCTGGTGCTGGAGCTGATGACGGGGC
AGGCCGGGTGGGACGAGGCGTCCGTGAGCTGGGCGCGGGGCATCATCCGCGACGGCAAAGG
GCTGGACATCGTGGACCCTAGGGTGCGGGACGAGGCCGCCGGGGCGGAGGCGGAAGCGGAG
CGAGAGATGGTGGAGTGCCTGCGGGTGGGGTACCTCTGCACGGCGCACGCGCCGGACAAGC
GGCCGACAATGCAGCAGGTAGTGGGGGTGCTCAAGGACATCCGGCCGGCGCCTCCCACACCC
GGTCCGGGCAGCTGA

>JL65_GRMZM2G034155
ATGCGTCTGCCCTTGCCATTGCCATCGCCAAGCCCCATGCGCCGCCGCCACCTCCCCCTCCCC
CTTCCCCTTCTTCCCCTTGTCTTACTCCACCTCCTCTTCGCGGCGTCCTCTGGCTCGCCTTCGTC
AGAGGTCGCCTTCTTGACGGCCTGGCTCAACACCACGGCCGCGCGCCCGCCGGACTGGTCGC
CGGCCGCATTGTCCCCGTGCAACTGGTCGCACGTCTCCTGCGCCGGCGGGACAGGCGAGACG
GGCGCCGTCACGTCCGTGTCCTTCCAGTCCGTGCACCTCGCGGTCCCGCTCCCGGCGGGCCT
CTGCGCCGCGCTGCCGGGGCTCGTGTCGTTTGTCGTCTCGGATGCCAACCTCACAGGCGGCG
TCCCCGATGACCTGTGGCGGTGCCGCCGCCTCACCGTGCTAGATATCAGCGGCAACGCGCTCA
CCGGCTCCATCCCGTCATCGCTCGGGAACGCCACGGCGCTCGAGAACTTGGCGCTCAACTCCA
ACCAGCTCTCCGGGCCCATCCCGCCGGAGCTTGCGGCGCTTGCGCCCACGCTCAGGAACCTG
CTCCTCTTCGACAACCGCCTGTCCGGGGAGCTCCCGCCGTCTCTCGGCGACCTGCTCTTGCTC
GAGTCGTTGCGCGCCGGCGGCAACCATGACCTGGCGGGCTTGATACCCGAGTCCTTCTCCAG
GCTGTCCAGCCTCGTCGTGCTCGGCCTCGCCGACACCAAGATCTCCGGCCCGCTCCCGGCGT
CGCTGGGCCAGCTCCAGAGCCTCCAGACGCTGTCCATTTACACCACGGCGCTCTCCGGCGCCA
TCCCGCCGGAGCTCGGCAACTGCTCCAACCTCACCAGCATCTACCTCTACGAGAACTCGCTGT
CCGGCCCTCTGCCACCGTCGCTCGGCGCGCTGCCGCGGCTGCAGAAGCTGCTGCTGTGGCAG
AACGCGCTCACGGGCCCCATCCCGGAGTCCTTCGGCAACCTCACTTCGCTCGTCTCGCTCGAC
CTCTCCATCAACTCCATCTCCGGCACCATCCCGGCGTCGCTCGGGCGGCTGCCGGCGCTGCA

GGACCTCATGCTCAGCGACAACAACATCACCGGCACCATCCCGCCGTTGCTCGCCAACGCCAC
GTCGCTGGTGCAGCTGCAGGTCGACACCAACGAGATCTCCGGCCTCATCCCGCCCGAGCTTGG
TCGCCTCTCTGGCTTGCAGGTGCTCTTCGCCTGGCAGAACCAGCTGGAGGGCGCCATCCCAGC
GACGCTCGCGTCCCTGGCGAACCTCCAGGCGCTCGACCTCTCGCACAACCACCTCACCGGCAT
CATACCGCCGGGCCTCTTCCTGCTCCGCAACCTCACGAAGCTGCTGCTCCTGTCCAATGATCTC
TCCGGGCCGCTGCCGCTGGAGATCGGCAAGGCTGCCAGCCTCGTGCGCTTGCGCCTCGGTGG
CAACCGCATTGCGGGGTCGATCCCCGCGTCCGTGTCTGGCATGAAGAGCATCAACTTCCTCGA
CCTCGGAAGCAATCGGCTAGCCGGTCCGGTGCCGGCCGAGCTGGGCAACTGCTCGCAGCTCC
AGATGCTTGACCTGAGTAACAACTCGCTCACGGGGCCGCTGCCGGTGTCGCTCGCCGCGGTG
CACGGCCTGCAGGAGCTCGACGTCTCGCACAACAGGCTTAACGGCGCTGTACCAGACGCGCTC
GGCAGGCTGGAGACGCTGAGCAGGCTCGTGCTCAGCGGCAACTCGCTGTCGGGACCGATACC
GCCGGCGCTCGGTCAGTGCCGCAACCTGGAGCTCCTCGACCTGAGTGACAACGTGCTCACCG
GAAACATCCCCGACGAGCTCTGCGGCATCGACGGGCTAGACATCGCGCTGAACTTGAGCCGGA
ACGCCCTCACCGGGCCGATCCCGGCGAAGATCTCGGAGCTGAGCAAGCTCTCAGTGCTCGAC
CTCTCGTACAACGCGCTCAACGGAAACCTTGCGCCGCTCGCCGGGCTCGACAACCTCGTCACG
CTCAACGTGTCGAACAACAACTTCTCCGGGTACCTCCCGGACACGAAGCTCTTCCGGCAGCTCT
CGACGTCCTGCCTCGCAGGCAACTCTGGGCTCTGCACCAAAGGCGGGGACGTATGCTTCGTGA
GCATCGACGCCAACGGTAACCCGGTGACGAGCACAGCCGAAGAGGCGCAGCGCGTGCACCGC
CTCAAGATCGCCATCGCGCTGCTGGTGACTGCGACGGTGGCGATGGTCCTCGGCATGATGGG
CATACTGCGAGCGCGGCGGATGGGCTTTGGGGGGAAGAGCGGCGGCCGCAGCAGCGACTCC
GAGAGCGGCGGCGAGCTGTCGTGGCCGTGGCAGTTCACGCCGTTCCAGAAGCTGAGCTTCTC
CGTGGACCAGGTGGTGAGGAGCCTGGTGGACGCCAACATCATCGGCAAGGGCTGCTCCGGCG
TGGTGTACCGCGTGAGCATCGACACCGGCGAGGTGATCGCCGTGAAGAAGCTGTGGCCTAGC
ACCCAGACCGCGGCGACGTCCAAGGACGACGGCACGAGCGGGCGCGTCCGGGACTCGTTCTC
GGCGGAGGTGCGCACGCTGGGCTCCATCCGGCACAAGAACATCGTGCGGTTCCTCGGCTGTT
GCTGGAACAAGAGCACCCGGCTGCTCATGTACGACTACATGGCCAACGGCAGCCTCGGCGCG
GTGCTCCACGAGCGGCGCGGCGCGGGCGCGCAGCTGGAGTGGGACGTGCGCTACCGCATCG
TGCTCGGCGCGGCGCAGGGGATCGCGTACCTGCACCACGACTGCGTGCCTCCGATCGTGCAC
CGCGACATCAAGGCCAACAACATCCTCATAGGCCTCGACTTCGAGGCATACATTGCCGACTTCG
GCCTGGCCAAGCTCGTCGAGGACGGCGACTTTGGACGGTCGTCCAACACCGTTGCCGGCTCCT
ATGGCTACATTGCCCCTGAGTACGGGTATATGATGAAGATCACAGAGAAGAGCGACGTGTATAG
CTATGGCGTGGTGGTGCTGGAAGTGCTGACGGGGAAGCAGCCGATCGACCCGACGATCCCAG
ACGGCCTGCACGTGGTGGACTGGGTGCGGCGGTGTAGGGACCGCGCCGGCGTGCTCGACCC
GGCGCTCCGGCGACGGTCGAGTTCGGAGGTGGAGGAGATGCTGCAGGTGATGGGAGTGGCG
CTGCTGTGCGTGAGCGCGGCGCCGGACGACCGGCCGACGATGAAGGACGTGGCGGCCATGC
TCAAGGAGATCCGGCTCGAGCGTGAGGACGTGGCCAACGTGGACGTCCTCCTCAAGGGAGGC
TCGTCGCCTCCTCATCATGTTCATGCCAACGCCGCGGCGATAGCTGACAAGGCCACGTCGTCA
ACGTCCAGCACGCCGCCGTGCCCGCAGGGTCCGAGCAACAGCTGCAGCAGTAGCAGCTTCTC
TGCCATCTACTCCTTGTCCAACAAGGCCAAGTCACCCTTTGGCTGA

>JL66_GRMZM2G114276
ATGCCGCCGTCGCGGTCGTTGTTCCTGCTGCTCATGCTGGCGCTGGCGCCGCTCGCGTCGCT
GACCGCGCCGGCGCCGGCGCCGGTGGCGAGGACAGCCGGGGTGCAGGCGGAGATCGACGC
GCTGCTCGCGTTCCGCCGCGGCCTGCGCGACCCCTACGGCGCCATGTCCGGGTGGGACGCG
GCGTCCCCCTCCGCGCCCTGCTCCTGGCGCGGCGTCGCGTGCGCGCAGGGCGGCCGCGTCG
TCGAGCTGCAGCTCCCGCGGCTCCGTCTCTCGGGCCCCATCTCGCCAGCGCTCGGCTCGCTG
CCGTACCTCGAGCGGCTCAGCCTCCGGTCCAACGACCTCTCCGGCGCCATCCCGCCGTCGCT
GGCCCGCGTCACCTCCCTGCGCGCCGTCTTCCTCCAGTCCAACTCGCTCTCCGGCCCCATTCC
CCAGTCCTTCCTCGCCAACCTCACCAGCCTCGACACCTTCGACGTGTCCGGCAACCTCCTGTCC
GGCCCCGTCCCCGTCTCGCTTCCTCCCAGCTTGAAGTACCTCGACCTATCCTCCAACGCCTTCT
CCGGAACCATCCCGTCGAACATTAGCGCCTCCACCGCCAGCCTCCAGTTCTTGAACCTGTCCTT
CAACCGACTCCGGGGCACCGTCCCGGCGTCGCTGGGAAACTTGCAGGACCTGCACTACCTCTG
GCTGGACGGGAACCTTCTCGAGGGGACCATCCCGGCCGCGCTGGCAAACTGCTCGGCGCTGC
TCCACCTGAGCCTTCAGGGGAACTCGCTCCGCGGCATCTTGCCGTCTGCGGTCGCCGCGATAC
CCACGCTGCAGATTCTCTCCGTGTCGCGGAACCAGCTCACCGGCGCCATCCCGGCGGCGGCG
TTTGGTCGTCAGGGGAACTCGTCCCTGCGCATTGTGCAGCTCGGCGGCAACGAGTTCTCTCAG
GTGGACGTGCCGGGAGGTCTCGCCGCGGATCTTCAGGTGGTGGACCTGGGAGGCAACAAGCT
AGCTGGTCCATTCCCGGCTTGGCTTGCTGGGGCGGGGGGACTGACGCTGCTCGACCTCTCGG

GCAATGCGTTCACCGGAGAGTTGCCGCCAGCGCTCGGGCAGCTCACTGCGCTGCTGGAGCTG
CGCCTCGGCGGTAATGCTTTCGCTGGCGCCGTGCCTGCGGAGATTGGCAGATGCGGCGCACT
CCAGGTACTTGATCTCGAGGACAATCACTTCACCGGCGAGGTGCCGTCAGCTCTTGGCGGTCT
CCCGAGGCTCAGGGAGGTCTATCTTGGTGGGAACACTTTCTCCGGCGAGATTCCGGCGAGCTT
GGGGAATCTGTCGTGGCTAGAGGCATTGTCCATACCGAGGAACAGACTCACTGGTGGCCTTTC
TGGTGAGCTCTTCCAGCTGGGGAACCTGACGTTCCTGGACCTATCAGAGAACAACCTCGCCGG
TGAGATACCTCTGGCCATCGGTAACCTGTTGGCTCTCCAGAGCTTAAATTTGAGCGGCAATGCC
TTTTCTGGTCACATTCCTACGACCATTAGCAACCTCCAGAACCTGCGAGTTCTTGATCTCTCTGG
TCAGAAGAACCTCTCTGGCAATGTCCCGGCGGAACTTTTTGGCCTACCGCAGCTGCAGTATGTG
TCATTCGCAGACAACTCCTTCTCTGGTGATGTTCCTGAAGGATTCAGCAGTCTTTGGAGTCTGC
GCGACCTCAATCTCTCCGGCAATTCATTCACTGGGTCAATACCGGCGACATATGGGTACTTGCC
GTCACTTCAAGTGCTCTCGGCCTCACACAACCACATTTCTGGGGAGCTGCCACCGGAGCTTGC
CAATTGTTCCAACCTTACTGTGCTTGAGCTCAGTGGCAACCAGCTGACCGGCTCCATCCCGAGT
GACCTCTCGCGCCTTGGTGAATTGGAAGAGCTGGACCTCAGTTACAATCAGTTTTCAGGCAAGA
TACCACCAGAGATATCCAATTGCTCATCACTCACCCTTCTCAAGCTTGATGACAATCGTATTGGC
GGAGACATACCAGCCTCTATTGCCAACCTCTCAAAGCTGCAGACGCTTGACCTGTCGTCCAACA
ATCTTACCGGCAGCATCCCTGCATCGTTGGCTCAGATTCCTGGCCTTGTGTCATTCAATGTGTCA
CACAACGAGCTTTCTGGTGAGATACCGGCAATGCTCGGCTCCCGCTTTGGTTCTGCTTCAGCGT
ATGCTTCAAACCCAGATTTGTGTGGGCCGCCATTGGAGAGTGAGTGCGGAGAGCAGCGGCGGC
GGCAGAGACGGCAGAAGGTGCAGCGCCTGGCTCTGCTAATTGGTGTGGTGGCTGCTGCCGTG
CTGCTCCTTGCGCTGTTGTGCTGTTGCTGCGTGTTTAGCTTGCTGCGATGGAGGCGTCGGTTCA
TTGAGAGCCGTGATGGTGTGAAGAAGAGGAGACGCAGCCCGGGACGCGGCAGTGGGTCGAGT
GGCACAAGCACGGAGAACGGTGTCAGCCAGCCGAAGCTGATTATGTTCAACTCAAGGATCACTT
ATGCCGACACTGTTGAGGCAACCCGCCAGTTTGACGAGGAGAACGTGCTCAGCCGAGGCCGC
CATGGCCTCGTGTTCAAGGCTTGTTACAGTGACGGCACCGTGCTGGCGATTCTGCGGCTCCCG
TCCACGTCTGCGGATGGTGCCGTGGTGATTGATGAAGGGTCGTTCAGGAAAGAGGCCGAGTCT
CTCGGGAAGGTGAAGCACAGGAACCTGACTGTGCTCCGTGGCTACTACGCCGGTCCTCCGCCG
GATGTCCGGCTGCTGGTCTACGACTACATGCCCAACGGCAACCTCGCCACGTTGCTGCAGGAA
GCGTCGCACCAGGACGGGCACATCCTCAACTGGCCAATGAGGCACCTCATCGCGCTGGGCGT
CTCGCGCGGCCTTGCGTTCCTGCATCAGTCCGGCGTCGTGCACGGCGACGTGAAGCCGCAGA
ACATCCTCTTCGACGCCGACTTCGAGCCGCACCTGTCGGACTTCGGCCTGGAGCCGATGGTGG
TGACCGCGGGTGCCGCCGCGGCAGCCGCCGCCGCGTCGACATCAGCTGCGACACCCGTGGG
CTCGCTCGGGTACGTCGCCCCCGACGCGGCCGCGGCCGGGCAGGCCACGAGGGAAGGCGAC
GTGTACAGCTTCGGCATCGTGCTGCTGGAGCTCCTGACGGGCCGGCGCCCCGGCATCTTCGC
CGGGGAGGAGGAGGACATCGTGAAGTGGGTGAAGCGGCAGCTGCAGCGCGGCGCCGTGGCG
GAGCTGCTGGAGCCGGGCCTGCTGGAGCTGGACCCGGAGTCCTCCGAGTGGGAGGAGTTCCT
GCTGGGCATCAAGGTCGGGCTGCTCTGCACCGCGTCGGACCCGCTGGACCGCCCGGCCATGG
GCGACGTGGTGTTCATGCTGGAGGGCTGCCGCGTCGGCCCGGACATCCCGTCCTCCGCCGAC
CCCACCTCCCAGCCGTCGCCGGCCTGA

>JL67_GRMZM2G059117
ATGGCCACTGCTACTGCCACCTCCCCTGCTCTCCTCCTACTCCTCCTCTTCCTGCTCATTGGCG
GGGCGCCACCAGCGACCAAGGCTGACATGCCGATGCCGGTGAACGAGGAGGTGCTGGGGCTG
GTGGTGTTCAAGTCGGCGCTGTCCGACCCGACCAGCGCGCTGGCCACGTGGACGGGGTCGGA
CGCGACGACGCCCTGCGCCTGGGCGCGCGTGGAGTGCGACCCGGCCACCTCCCGCGTGCTC
CGCCTCGCGCTCGACGGGCTCGCGCTGTCCGGCCGCATGCCCCGGGACCTCGACCGCCTGCC
GGCGCTCCAGTACCTCTCCCTCGCCCGCAACAACATCTCCGGCCCGCTCCCGCCGGGCCTCTC
CCTCCTCGCCTCCCTCCGCTCCCTCGACCTCTCCTACAACGCCTTCTCCGGCCCGCTCCCCGA
CGACATCGCGCGCCTCGCCTCCCTCCGCTCCCTCGACCTCACCGGCAACGCCTTCTCCGGCCC
GCTCCCGCCCGCCTTCCCCGAGACGATCCGCTTCCTCGTGCTGTCCGGCAACCAGTTCTCCGG
CCCCGTGCCGGAGGGGCTGGCGTCTGGGAGCCCGCTCCTGCTCCACCTCAACGTGTCCGGCA
ACCAGCTGTCCGGCTCGCCGGACTTCGCGGGCGCGCTCTGGCCGCTCCAGCGCCTGCGCACG
CTCGACCTCTCCCGCAACCAGTTCTCCGGCCCCGTCACCGGCGGCATTGCCAGGCTCCACAAC
CTCAAGACCCTCATCCTCAGCGGCAACCGGTTCTTCGGAGCCGTCCCGGCGGACATCGGCCTG
TGCCCGCATCTCAGCGCCATCGACCTCAGCTCCAACGCGTTCGACGGCCACCTCCCTGACTCC
ATCGCCCAGCTCGCCTCGCTGGTCTACCTGTCCGCGTCCGGGAACCGGCTCTCCGGCGACGT
CCCCGCCTGGCTCGGCAAGCTGGCAGCGGTGCAGCACGTGGATTTATCCGACAACGCGCTCA
CCGGAGGCTTGCCGGACTCGCTCGGCGACCTCAAGGCGCTCAGGTACCTGAGCCTATCCAGG

AACCAGCTCTCGGGCGCCGTCCCGGCCTCCATGTCCGGGTGCACCAAGCTCGCCGAGCTGCA
CCTGAGGGGCAACAACCTCAGCGGCAGCATCCCGGACGCTCTGCTCGACGTCGGGCTCGAGA
CGCTCGACGTGTCGTCGAACGCGCTCTCGGGCGTCCTCCCCTCGGGCTCCACCAGGCTGGCC
GAGACGCTGCAGTGGCTCGACCTCTCCGGCAACCAGCTCACTGGCGGCATCCCCACCGAGAT
GTCGCTCTTCTTCAAGCTCCGGTACCTCAACCTGTCCCGCAACGACCTCCGCGCGCCGCTGCC
GCCGGAGCTCGGCCTGCTCCGCAACCTGACGGTGCTCGACCTGCGTAGCACTGGGCTGTACG
GCGCCGTGCCAGCCGACTTCTGCGAGTCCGGTAGCCTCGCCGTGCTCCAGCTCGACGGCAAC
TCCCTCTCCGGCCCCATCCCCGACAGCATCGGAAACTGTTCTTCCCTGTACCTCCTGAGCTTGG
GGCACAACGGCTTGACGGGGCCGATCCCGGCGGGCATCTCGGAGCTGAAGAAGCTGGAGATC
CTGCGGCTGGAGTACAACAAGCTGAGCGGCGAGATCCCGCAGCAGCTGGGCGCGCTGGAGAA
CCTGCTGGCCGTGAACATCTCGCACAACCGGCTGGTGGGGCGGCTGCCGGCGTCGGGCGTGT
TCCAGAGCCTGGACGCGAGCGCGCTGGAGGGCAACCTGGGCATCTGCAGCCCGCTGGTGACG
GAGCCGTGCAGGATGAACGTGGCCAAGCCGCTGGTGCTGGACCCCAACGAGTATACGCAGGG
CGGCGGCGGCGGCGACAACAACCTGGAGACGGGCGGCGGCGGTGGTGTAGAGGCGCCGAGG
AAGCGGCGGTTCCTGATGAGCGTGTCCGCCATGGTGGCCATCTTCGCGGCGGTCGCGATCGT
GCTCGGCGTCATCGTGATCACGCTGCTCAGCGTGTCGGCCCGGCGGAGGGTCGAGGCTGCGG
GTGTTGGAGGCCCTGGCCACGACCGGAAGGAGGTGGACGAGAGCATCGTCACCACCAGCTCG
ACGACGACGACCAAGTCATCGTCGTCGCCGCCGCCCGGGGGCAAGGTAAAGGAAAAGCTCGC
CACCGGCAAGATGGTGACGTTCGGGCCCGGGAGCAGCCTCCGGTCGGAGGACCTCGTGGCG
GGCGCCGACGCGCTGCTGAGCAAGGCAACAGAGATCGGGCGCGGCGCGTTGGGCACGGTGT
ACCGCGCGGCCGTGGGCGACGGGCGGGTGGTGGCGGTGAAGAAGCTGGCGGCGGCTCACCT
GGTGCGGTCGCGCGAGGAGTTCGAGCGGGAGGTGCGCGTGCTGGGAAAGGCGCGGCACCCA
AACCTGCTGGCGCTGAGGGGCTACTACTGGACGCCGCAGCTGCAGCTGCTGATCACGGACTAC
GCGGCGCACGGCAGCCTGGAGGCGCGGCTGCACGGCGGCGGAGAGGCGGCGCCGATGACG
TGGGAGGAGCGGTTCCGCGTGGTGTCCGGGACGGCCCGGGCGCTGGCGCACCTGCACCAGG
CGTTCCGGCCGGCGCTGGTCCACTACAACGTGAAGCCGAGCAACATCTTGCTGGCGGACGCG
GAGTGCAACCCGGCGGTGGGCGACTTCGGGCTGGCGCGGCTGCTGCATGGCAGTGGCAGTG
GCAGGCAGGTGGCGATGGCGGGCAGCCGGTTCCGGCAGGGCGGCGGCGGCGGGATGGGGT
ACGTGGCCCCGGAGCTGGCGTGCCAGAGCCTGCGGGCGAACGACAAGTGCGACGTGTACGG
GGTGGGCGTGCTGATCCTGGAGCTGGTGACGGGGCGGCGCGCGGTGGAGTACGGGGACGAC
GACGTGGTGGTGCTGACTGACCAGGTGCGCGCGCTGCTGGAGCACGGCAACGCGCTGGAGTG
CGTGGACCCGGGCATGGGCGGACGCGGGCACGTCCCGGAGGAGGAGGTGGTGCCGGTGCTG
AAGCTGGGCATGGTGTGCGCGTCGCAGATCCCGTCCAACCGGCCGTCCATGGCGGAGGTGGT
CCAGATCCTGCAGGTCATCAAGGCGCCCGTGGGCGGCGGTGGCAGAATGCAAGCCTCCTTCT
GA

>JL68_GRMZM2G089819
ATGCCGCCGCCGCCACGGAGGCTTCCACCGCGCCTCGCCCACCGCCGCTGTGGCATCGCCGC
CGCGTCCACGCTCCTGTTCCTCCTCCTGCTGCTCTCCGCTCCTCCTACCATCGCCCAGCACCAC
CGCCACCACGCCACGGGCGACGGCGTGGTGATCAGCCAGGCCGACTACCAGGGCCTGCAAGC
CATCAAGCACGACCTCTCGGACCCCTACGGCTTCCTGCGCTCCTGGAACGACAGCGGGCTCGC
CGCCTGCTCGGGCGCGTGGGCGGGCGTCAAGTGCGTCCTAGGCAGCGTCGTGGCCATCACGC
TCCCCTGGCGCGGCCTCGGCGGCATGCTCTCCGCGCGCGGCCTCGGCCAGCTCGTCCGCCTC
CGCCGCCTCAGCCTCCACGACAACGCCATCGCGGGCCCCGTCCCGTCCTCGCTCGGCTTCCT
GCCGGACCTCCGCGGCGTCTACCTCTTCAACAACCGCTTCTCCGGGACCCTCCCCGCCTCCAT
CGGAGGGTGCGTCGCCCTGCAGGCCTTCGACGCCAGCAACAACCGCCTCACGGGCGCGGTCC
CCGCCGCCGTCGCCAACTCCACCAGGCTCATGCGCCTCAACCTCAGCCGGAACGCGCTCTCC
GACGCCGTACCCGTCGAGGTCGTCGCCTCCGCCTCGCTCATGTTCCTCGACCTCTCCTACAAC
AACCTCTCGGGCCCCATCCCCGACGCCTTCGCCGGCTCCTACAGCTCGCCTTCGAAGCTGCGC
CTCAACAGGGACGCCATCACCGGGAGCTACCAGCTCGTCTTCCTCAGCCTCGCGCACAACTCC
CTCGACGGGCCCATCCCGGAGTCCCTCACCAAGCTCACCAAGCTGCAGCAGCTCCACCTCTCC
GCCAACAGCCTCAACGGCACCATCCCGGCGCAGCTCGCCGCGCTACCGGACCTCAAGGCGCT
GGACCTCTCCGGGAACGCGCTGGCCGGCGAGATCCCGCCCGGGCTCGACAACCTCACCGCCA
CGCTCCAGTCCTTCAACGTGTCCTACAACAACCTCTCCGGCGCGGCGCCGCCGTCGCTGGCGC
GCAAGTTCGGGGAGCCCGCGTTCACCGGGAACGTCCTCCTCTGCGGCTACTCTGCTTCCACGC
CCTGCCCGGCGTCCCCGTCCCCCGCGCCGGCGTCGCCCGCGCAGGAGCCGCCGCGCGGAGG
CCGCAAGTTCAGCAGGAAGGCGCTCGTGCTCATCGTCGCCGGGGTCGTCGTCGGCGTCCTCG
TCCTGCTGCTCCTCTGCTGCCTCCTGCTCTGTTTCCTGAGCAGGAACAAGAGGTCTCCCAGCGG

TACTGCTGGAACACGGAGCGGGAAGCAGGCGGCCAAGGAGACCGGTGGTGCTGGCGCCGCC
GCCGCGGCGGCCGGGCGTGGCGAGAAGCCCGGGTCCGGCGCGGCGGAAGTGGAGTCCGGT
GGCGATGTGGGCGGCAAGCTGGTGCACTTTGATGGGCCGCTGGCGTTCACGGCCGACGACCT
GCTTTGCGCCACTGCCGAGATCATGGGGAAGAGCACCTACGGCACGGTGTACAAGGCCACGCT
GGAGGACGGCAGCCTGGTGGCCGTCAAGCGGCTGCGGGAGAAGATCACCAAGGGGCACAAG
GAGTTCGAGGCCGAGGCGGCGGTGCTGGGACGGATTCGCCACCCCAACCTGCTGGCGCTCAG
GGCATACTACCTGGGACCCAAAGGGGAGAAGCTACTCGTCTTCGATTACATGCCCAACGGCAG
CCTCCACTCGTTCTTGCACGCTCGCGCTCCCAACACGCCGGTGGACTGGGCGACGCGGATGAC
GATCGCCAAGGGCACGGCGCGTGGCTTGGCCTACCTCCACGACGACATGAGCATCGTGCACG
GCAACCTGACGGCCAGCAACGTGCTCCTGGACGAGCAGCACAGCCCCAGGATCTCCGACTTCG
GGCTGTCGCGGCTGATGACGACGGCGGCAAACTCGAACGTGCTGGCGGCGGCGGGCGCGCT
GGGGTACCGCGCGCCGGAGCTGTCGAAGCTGAAGAAGGCGAGCGGCAAGACGGACGTGTACA
GCCTAGGCGTCATCATCCTGGAGCTGCTGACGGGCAAGTCCCCCGCGGACAGCACCAACGGC
ATGGACCTGCCGCAGTGGGTGGCGTCCATCGTGAAGGAGGAGTGGACCAGCGAGGTGTTCGA
CCTGGAGCTGGTGCGCGACGCCGCCGCGGGGACGGCCGCGGACGAGCAGCTCATGGACACG
CTGAAGCTCGCGCTGCACTGCGTCGACCCGGCGCCGGCGGTCAGGCCGGAGGCGCACGAGG
TGCTGCGGCAGCTCGAGCAGATCAGGCCTGGTGGCAGCGAGGGCGGCGCTGGGCCAAGCGA
AGAAGGTGGTGGTGGCGCCGCCGCCGCCCATGTGCCGGCGGCTTCTGCTTCTGCGGGAGACG
ACGACGAGTAG

>JL69_GRMZM2G344857
ATGGAGAGGAGCTGTTTGACCCGCGTGTCGCACAGGGCTAACTCCAAAGCTCAGGTCAACGAG
CTGCTGCTGGTCTGGAGGACAGCAACTGATGCCTTTGCCGTGCCATGGAAAGAATTGAAAAGGT
ATCAACCATCTGCTGGCCTGGAAAAAGCTTGGAGCAGGAAGAGAGAAATGGTGGCATGGCAAT
ATGGCATGCATTCCCATGCGCAGTGCACAACACAATGCCTGGACTGGATTCAATTCAACGGCCG
AGGCGCTGCCTCCGCTTCCCTCCAAATGTTGCGCTCCCGAGCTCTCCTCGGCAGCCTCCCACC
CCCGAGTCGCCCCGACAACTCCTCCCAGCCTCCAACCACCGAGAGCGTAGAACTCGTAATCCA
AGGCCAGCGCGCGCACGCCATTGCCTCGCCCCGTCCCGCTCCGGCGCCCCCCACCGCCAGC
ATGCACAGGCTCGCCCCGCCCCGGTCGTGCTGAGACGACGTACCTATACTATACACATACACA
CGGACACACCAAACGACGCCAACGTGGCGACGTCGGCGAGGAAGATGAGCCAGCTCCCCGCC
CCGGCCAGCCGGCTGTGGGAAGCGAGCATCCGCAAGCTCCAGACCATCCGCCGCGCGACGAC
AGCCACAGGGCCCGCGGCCGACGGCGCCGATGGCGGGGCCGCCCTCACGCCCAGTCTCCTC
TCCATCAATTCCTCCTCCGCGTCCAGCACTATCTACCAGTACCACCACGAGGACGGCGAGGACA
GCGACACGAGCACCGATGGCGGCAACGATTCGGAAGCCGGCGAGGACGAGGACGCCGGCGA
GCCCACGCACGCGGAGCAGCTGCTGCCCAGCGGGGACTTCTACCAGGGCGACCTGCGCGGG
GACCAGCCGCACGGCGCGGGCAAGTTCCTCTGGACCGACGGCAGCATGTACGAGGGCTCCTG
GCGCCGCGGCCGCGCGTCCGGCCGCGGCAAGTTCTCATGGACCTCGGGGGCCACCTACGAG
GGCGACTTCGCCGGCGGGTACATGCACGGCCAGGGCACCTACATCGGCGAGTTCGGGGACAC
CTTCGCGGGGCTCTGGGCCAACAACCTCCGCCACGGCCGGGGCACGCAGGCGTACGCCAACG
GCGACGTGTACGACGGCCACTGGCGCGACGGCCTGCAGGACGGCCACGGGCGCTACATCTGG
CGCCACGGCCACGAGTACATCGGCACCTGGAGGGCCGGCGACATGCACGGCTGCGGGACCGT
GATCTGGGCGGACGGCGACCGCTACGACGGCGCCTGGGAGGACACCAGGCCCAAGGGCCAG
GGCACGTTCCGCTGGGCCGACGGCGGGATGTACATCGGCACCTGGTGCGAGGAGGCCGGCG
CCGTGCACGCCGACGGCGTCTACTACCCGCCGTCCGGTGGCCCCGCGGTGCCCGTGCCCCGC
GAGCCCTGCGACCCCATCACGGCGCTGCTCCAGGAGCTAGAGGTGTGCGAGGGCAAGACGGC
GTCGCTCATGCCGTCGCAGAAGGTCCTCACGTGGCCCGGCGTGGAGGCCGTGCAGAAGAAGC
CGGTGTGGCGCCCGCCCAAGGTTAGCCTTGACCAAGGCAGGAGGTCCAGCGTGAGCAGGAGG
AGCAGCGCGTCGCTGGACCTGGACATGCTGCAGCCGGCCGCCGCCGAGGGTGGCGAGACCG
AGGAGGTGCGCGCGGACAGGTCGTGCCTGCGGACGTCGTCGTGCATGCGCACGCCGCCGAG
GCCGGGCAAGAAGCAGGGGGAGACCATCTCCAAGGGGCACAGGAACTACGAGCTCATGCTCA
ACCTGCAGCTTGGCATCAGGCACGCTGTTGGTAGGCAGTCAGCGCCAAATACGCTGGATCTCA
AGTCATCGGCATTCGATCCAAAAGAGAAGATATGGACAAGGTTTCCTCCCGAAGGATCAAAGCA
TACTCCTCCTCACCAGTCCTGCGATTTCCGGTGGAAAGACTACTGCCCCCTGGTTTTCAGGACT
CTGCGCAAGCTCTTCGACGTCGATCCAGCAGACTACATGATCTCCATTTGCGGGGACGAGGCG
CTCCGGGAACTGTCGTCTCCTGGTAAGAGCGGGAGCTTCTTCTACCTCACGAACGATGACAAGT
ACATGATCAAAACGATGAAGAAGGCAGAGGTTAAAGTGCTCCTTAGGATGCTTCCAGCCTACTA
CAAACACGTCCGCTTTTTCGAAAACACTCTGATCACTAAATTCTTCGGCCTGCACTGCGTCAACG
TCAAGCTTACAGGAGCTATCCAGAAAAAGGTCCGGTTTGTTATAATGGGGAACCTTTTCTGCTCC

AGCTACGCGATCCACAGGCGCTTCGACCTGAAAGGGTCCTCGCATGGTCGCATGACTGACAAA
CCCATCGATCAGATCAGCGAGCACACAACGTTGAAGGATCTCGATCTTAATTTCATCTTCCGGCT
GCCAGGGACCTGGTTTGAGGAATTCTGCAGGCAAGTGGACAAAGACTGCGAATTACTGGAGGC
GGAGAGGATCATGGATTATAGTCTTTTGGTCGGTATTCACTTCAAGGACAGATGCAAAGACAGC
AGCAATGGTGACAATGGGACGTCACAGAATGCTGAAGATTCTGAAGAAAACAGGTAA

>JL70_GRMZM2G087243
ATGAGCTTTGGGAGCTGTCCGGCCGTGGCCGGGGGCGCGACGGCAGAGGAGCTGCTTGAACG
GGCGCGGGGGCTGGTGCCACCCGCCCTGGCCGCTGCGCGCGCGGCCACTGGCTTCGGTGGG
CGGTGGAAGGCTATCGCAGCGAGGCTGGGGAGGCTGCCGCAGTGCCTGTCGGACCTGTCTAG
CCACCCCTGCTTCTCCAAGAACTCGCTCTGTCGGGAGCTGTTGCAGTCGGTGGCTGCAACGCT
AGCGGAGGCCGCCGAGCTCGGTGCGCGCTGCCACGAGCCTCCGAAGGCGGGGAAGCTGCAG
ATGCAGAGCGACCTCGACGCGCTGGCCGGGAAGCTGGATCTCAACCTCAGGGATTGCTCTCTT
CTTGTCAAGACTGGTGTGTTGTCTGACGCGACGGTTCCCGCGGCCCCGGCTGAGACAGCGTCG
GCTGCTGGCGCACAGGTGGACGTGCGGGAGCTGCTTGCCAGGCTGCAGATTGGGCACGCAGA
GGCGAAGCACCGGGCTGTGGATGGCCTGCTCGATGCTCTGCGCGAGGACGAGAAGAGCGTGC
TGTCGGCGCTCGGCCGTGGCAACGTGGCCGCACTCGTGCAGCTGCTCACGGCGACGACACCC
AAGGTTAGGGAGAAGGCCGCCACTGTTTTGTGCTTGCTCGCCGAGTCTGGCAGCTGCGAGGGC
TTACTGGTGTCAGAAGGCGCGCTGCCACCGCTCATCCGGCTGGCTGAGTCCGGTAGCCTTGTT
GGGCGGGAGAAGGCCGTCATCACGCTGCAGCGGCTGTCCATGTCGGCCGACATTGCCCGTGC
GATCGTCGGCCACAGCGGCTTCCGCGCTCTCATCGACATGTGCCAGACCGGGGACTCGATCAC
ACAGTCCGCCGCGTCGGGAGCGCTCAAGAACATCTCTGCGGTGCCAGAAGTCCGGCAAGCGC
TGGCCGAGGAAGGCGTGGTGCGAGTCATGATCAACATCCTCGACTCCGGCGTCGTTCTCGGCT
CCAAGGAGTACGCCGCGGAGTGCCTGCAGAACCTCACGTCAAGCAACGACAACCTGCGGCGC
GCCGTCGTGTCCGAGGGCGGCCTCCGTAGCCTGCTCGCCTACCTCGACGGGCCACTGCCGCA
GGAGCCACCCGTGGCCGCGCTCCGGAACCTGGTCACCGTTGTCCCACCTGACAGCCTGGTGT
CACTTTGCGTGCTCCCGCGCCTCGCACACGTGCTCCGCGACGGTTCCGTGGGTGCCCAGCAG
GCTGCCGCGGCGACCATCTGCAAGGTCTCCGGCAGCATGGACATGAAGCGGCTGGTGGGCGA
GCACGGGTGCGTCCTGCTCCTGGTGCGCCTGCTGGAGGCCAAGTCGAACGGCGCTCGCGAGG
CCGCGGCGCAGGCGGTGGCGAGCCTGATGGGGTGCCCCGCGAACGCGAGGGACGTCAAGAA
GGACGAGAAGAGCGTGCCCAACCTGGTGCAGCTGCTGGACCCGAGCCCCCAGAACACGGCCA
AGAAGTACGCCATCTCCTGCCTGCTGGCGCTTTCGGCGAGCAAGCGGTGCAAGAAGCTGATGG
TCTCTCACGGCGCCATCGGGTACCTGAAGAAGCTCTCGGAGAAGGACGTGGCCGGCGCGAGG
AAGCTGCTCGAGAAGCTGGACCGCGGTAGGCTTCGCAGCCTGTTCAGCAGGAAGTAG

>JL71_GRMZM2G133716
ATGGCGGCGGCGGACGGGGACGCCTTGATCGCGGCGTTTGCGGTGTCCAAGGGCGGCATCGT
GCTCAAGCACATTTTCCTCAACGCACCACCGCCGGAGGCGATGTGCGGGTCCGGCGGAAGAG
AAGTGGAGAGTGATGAGGAGGACCCGCCGGTGATGGTGGGTCGGCACCCCGACTGCCACGTG
CTCGTCGACCACCCCAGCGTCAGCCGCTTCCACCTCGAGCTACGGTGCCGACGTCGACAGAGT
CTCATCACCGTCACCGACCTCCACTCCGTGCACGGGACATGGGTTTCGGGGCGACGGATCCCA
CCCAACACGCCAGTGGATCTCGCTACCGGTGACACGCTGCGGCTCGGGGCCTCTAAGAGGGA
GTATAAGCTGCTCTGGCTGTCGCTTCGTGAGGCATTTGAGATGGACGATCTGATGTATATGCCA
TCGCTGCCTGAGGAGGACAAAGAGGAGCCATATGTTAAGGAACCAAGCAGCAAGTTATTGCCT
GGGCACAGGGATTCAGTGAACATGGAGACCCATCAGGATACAAGTGAACAAATTGTGTCTGAAG
ACATTGCATTTCCAGCCAAAGTGGCCCCCTCAGCACCTCCATTGTCTGAGTTCCTTCAACCTTTT
TTTGTGGAAGAGCATTCACTTTCCCAATTTCATGAGAAAAGAAACGGAGTGACAGAGGAAAAGTT
GGTCGACAAGAATCAAATTTCAGAATCTTTTGGTTCGTTGATCATACAGGAAATGCCAGGCACAC
TGACAAATGCTGGAAAATCAATCCAGTCAGGCGAGCAGGAGGATGCCTCAAACAAAGTGTCCAA
GAGGTCCAAGTTGAAGTCAGTCAAATCACTTCGTGTTGACACAGGCAGGAGCAGCGAGAGAAG
CAGCACTCTGAGCCATAGCTTTCAGAAGGGAGACCAAAATGATATTGTTGTATGTTCTCAGAGCT
GTGGGACAGAATGTGCGGTATGTATAGCTTTATTTGGTATTTCTGAAATTGAAAAGGCTGAAGAA
AAGGAAGAGTTGATTGCTGAAGATAACGTTGACATGAATCCCCCGGCCAGCATGATCATGGAGG
GAAATATGAACGAGAGAAAACCTGATAATTATATCCCACAAGATCCAATTGGTGCAAAGCTGCAG
AAGAAACTAGGCTTATTAGATTCTGCTCTCCCTTTGCATTTCAAAGATGATGTGTTCGCCGACAA
GGAAATTCCACAATGGAATGTTGCTAGTGTTCATACAGAATCTGAGCTTCTTTCAGAATATCTTAT
AATACCAGAAGTGAAGCACGATGACTTGAACCATCTTAACTTGGAGGAAGGTCTCTCAAAAAGC
GAGAATATAAACCCAAATAAGATTACTGAGGGCCCTGGGAACTGTCAGCTCGAAGGGACCATTC

GTGGGAATCTATTTGACAATTTGGATACTGATGGAATTGAAGAAGGTGAAGAGATCTGCCCACT
GGACAAGGATGAAATCACCCCCAATGGATCAGGTAACATAATAATGGAGAGGAGCAATATAGTT
TTAAAGCCTACCATTTCCCAACAGTTGATGGATTCTATTTCCCCTCTCAATTTAGATCATGGCGAC
TTTTCTGAAAATGAGAACTCCATGCTGAACACTGGAGATCAGATGAAATTAAATGAACCTGTCTC
TGAGAATCTTAACCCATTAATTCCAACTGATGAAAAATATCTGAAGAGCCAAACGGAGGAGTGTA
TGCCCATTTCATATCTGGAATTCAAAGATGATATCCTTCTGGATAGGGAAAACTCAGTGCTGGCT
CCGAGAAAGTACGAGGCTATGTCCCCTGTGAGGCAGGAAAATCTGTTCTCAGACAAAGAGAAC
GTGACTCCTGCCTCTAAGGTGAAGACCGTTGTTAGGGGAGTTCTTGGTACAAGGATGGATAATT
CAGTGTCAGCAGCAAATGCTTCAAATAAGAATAAAGTTCTTGGGTCAAGGGTCGATAATTCAGTG
TCAACAGAGAATAGCTCAAATAAGAAGCAATGTGAATTATCATCGAAGTCCAAAAAAGTCCACAC
AGTAGATTTTGATGTTTTCTATTCGGATAAGGAGAACTTGACTCCTATATCTTCAGGAGGCATGA
AAGCAAGGAAGTGTTTTCCCAAGGACCTCTCAGTCGACTTAGACCAAGATCAGGAAGCATTCTG
CTCAGACAAGGAGAACTTGACACCACTATCTTCTGCAGCTCGGAAAACAAGGGACATGTCTGGA
AACCTTACACGAGTTGAAAGTGCGGTCACAAAGAAAAGGGTTGTTGGTAGGCTCCCCTTCCAGA
CTCTTGTGTCAAATTCTCCTTTGAGACCTGCTAGCTCGCATGATTGTACCTGTGCTGTTGCTAGA
CCAGCTGGCGTTGCTGCTGGTGACTTGGCGATCAAGTTAGAAGATAAATTGAACGATCTCTCAT
GTAATGGCCACGAATCAGGTAGTGCTGGAGAAGGCATGAAAACCTGGACCATGGTGGCTAATA
CAGACAGTCTTCTTGATGACGAGTCTAGGAAGGCTATTATGCTGCTAAAAGGCTTAAAAGGAACT
CGCCTGTTCATACCAAGGATTGTGATCAGGGAGTTGGATTCCATGAAGCAGCGGGAGGGCCTG
TTCAGACGGTCAACGAAGGCGACCTCCATCCTGCAATGGATCGAGGAGTGCATGGCAAGGGAG
AGCTGGTGGATCCACGTCCAGAGCTCGGCCGACATGTTCCCGGTAGCACCAACCCCGCCCGC
GACCCCTTCAGCGCAGCGCATCGACGAGGAGATCGAGATCAGCTCCGGCTCCTTCAACCCGAT
GATGGCGTTGTTCGGCCCTCGAAGCTCCGCCGCGCTCGCGGATATGATATCTCCCAGACCCGA
GGACCGCGTCCTCGACTGCGCCCTCCTCGTCAGTAGGGTCCGGAGCAACGAGAAGGTCGTGG
TGCTGTCAAACAGCGTCACGCTCAAGATCAAAGCCATGGCAGAGGGCCTGCCATGCGAGGGAG
CCAAGGAGTTCCGCGAGTCCCTCGTGGACCCGTCCTCCAGGCGGTTCATGTGGGCGGCCAGC
GCCCCACGGGGGTCGGCCTGGTCCTGCCTGGACGCGTCCGCGCTGGCGGAGAACTACTACAA
CAGCCGCCACCACGCTATGAAGCGGAGGGTTCTGGTGGCGGCGAGGCCCTCCGAGTCCGAGG
CCGCCAAGGGCCTGAAGCTGATCCTGCGTCACAATTCCCTGTACGCGCAGGCCACGGACGCC
GTCAACAAGACGCCGCTGGTATCGCTGGCCGCGGTGTGA

>JL72_GRMZM2G469304
ATGTTCCAGTCCTCGGTACTCCAGTCCAGACATGAGGATCTGTATGGTGCAGCACCATCAGGAT
TCAGGACAGTAGTGCGGCTAGTGCCTGTGGCGACCTTGCATTTGCCACTGCGAGTACCGGTCC
AAGTTCTTGAAAAGCGATTGAATGATCAATTCGTCATGCGCCGTGCTCTTGAGAAAGCTCTAGGT
TACAAGCCTTGTGCTATCCATGCATCAAGTGAGAACTGCATTCCAAAGCCGACTGAGGAGCTAA
TAAAGGAGATTGCAGTTCTCGAGCTAGAGGTCATATGCTTGGAGCAACATCTCCTAACACTATAC
AGAAAGGCATTTGAGCAACAATTTTCCCCTGCAAGTTCTGCTTGTGACATGGAGAGCAACAAGC
AGCCAGCAAGGTCCTTTTCAGGCATACTCTCTGAAGCTCCAGAGCTGAGCTTCTCGACCCCAAG
GAAGCATCATCAGCTAAACCAGTCCAGTCGAATGGTCCTGGCACGCAAGTCCACGCCTACAGCT
TCTACAAGCGAGACTAGCAATGAGAAGATCAATATCGGACGCAGCCATTCCTCGCTCCTGCACC
GCTCCGTTAGGGTATCTCCGTCGGCAAACAATCTCGCTAGAGCTCTGAAACCATGCCATACTTC
GCCTCTATCTTTTGTTGAGGAAGGGAAATGCATGGATCCTGGCGTGGTGAGTTTGGCAGACATC
TTGGGGACCAGGGTCGCGGACCATGTTCCCCAAACGCCGAACAAGATATCCGAGGACATGATC
AAATGCATTACGGCCGTATACATGAGGCTGAGAGACGTCCCCGCCGTGCAGCACGCCTTCTTC
CCCTCACCGTGCTCATCCTTTTCTTCGGCGAGCGGGCTGTCTTCCAAATACACCGCGGACATAT
GGAGCCCCAGGTGCAGGAAAGAGAGCTTCGTTGAGGAGTGGCAGGACAACGGGTTAGGCAAC
GGTGAATCTAGGGAGCTGGCCCTGCCATATGATTCTGTGGTAGAGGTGTCTGCTCTTTGCAAAG
GTGATCAGAGGTCCGCTGACGTGAAGGATATGCTGCGCAAATACATGTCACTTGTGCAGCTTCT
CGAAAGCGTTGATCTCAGCGGGATGAAACATGAAGAGAAGCTCGCTTTCTGGATCAACGTGCAT
AATGCGATGATGATGCATGCCCATATAGAGTACGGGATCCCACAGAGTAACAGCAAAAGGATAC
TGCTTACCAAGGTGTCGTACATCATTAGTGGCCAAAGAGTGAACGCGGAGCTGATAGAGTACCA
GATCCTGTGCTGCCGAGCGCACTCTTCTGGACAGTGGCTTCGGCTGCTGCTATACCCGAAATG
GAAGTCCAGGGACAAGGACGAGCTGCAAGGCTTCGCCGTGGACCGCCCCGAGCCGCTGGTGC
ACTTCGCACTGTCCTCCGGTAGCTACTCGGATCCAGTGGTGCGGCTGTACAGCCCCAAGAGCG
TGTTCCAGCAGCTGGAGGCGGCGAAGGAGGAGTACGTCCGCGCCAACGTGGGCGTCCGCGG
GCGGGGGCAGCACAAGATCATCCTCCCCAAGGCCCTGGAGCTGTACGCGAGGGACGCCGGGC
TGGGCGCGCAGGAGGTGGTGGCCGCGGTCGAGTGCCACCTGCCCGAGGGCCTCCGGGACGC

CGTGCGCCGGAGCCAGCAGCAGGCCGGCCGGGCGCGCGCCGTGGAGTGGAAGCCCCACAAC
CTGGCGTTCCGGTACCTGCTGGCCAAGGAGCTGGTCGGCGGGTCCCCCGCGTGCAGCCGGCC
GCTGGAGCCGGTCAGTGCGCCGCTGCGGGCTGGTCCGTGA

>JL73_GRMZM2G002280
ATGGCGTCCAGAGATGCGGGCGCCAGCCCCAGATCCGCGCCGCATCGCCCTGGAGCGGTGGC
GCCCAAGGACGGCGCCGACCTGCTCAGCCCGCGGTTCCGTTCAGCCGCCGCGCTGGCCGGGT
GGGACGAGGAGTCCGTCCTTCTCGCCGCGATGGTCGTCGAGGACACTCCCGTCCGCGAGTCC
CGTCGCAAGAGGCGGGCCTCCACCTCCTCCGCCGGCGGCAGCGCCGGATCCAGCACCAGGAA
GATGAGGCCGCGTAGGCAGTCTCCGGCCAAGATCCCGCCGGTGGTTCTCGCGCTCGACGATG
ACGACGACGACGACGCGGCAGATGGCAAATCGATAGTGAATGCTGCCACCAAGGAGGAAGAGA
AAGTTGTCGTTGTGGGCGACAAGGAGGCGTCCGGATCTGGCGACAAGGCGGCGTCCGGATCT
GGCGACAAGGCGGTGGCCACGGGAAACCTGCCGTGCATGGATCGCCTCCGGGAGGAGCTCTC
TTGCGCTATTTGCCTGGAGATCTGCTTTGAGCCGACCACCACGTCTTGTGGGCACAGCTTCTGC
ATGAAATGCTTAATACATGCTGCATCCAAGTGTGGAAAACGGTGTCCAAAATGCAGGCAATTCAT
CAGCAACTCAAGATCGTGCACCATCAACACAGTGCTCTGGAACACCATTCAGCTGTTGTTTCCC
GGTGAAGTTGAGGCAAGAAAGAATTCGACCTCACCATGCGATAAGGACGCGAGCCGCAGCCCA
CTCAGAGCTAATAGGCTATCACAAGGTGGCAATGGTATGAGAACTAGAACTAGGAGCAGGAGCA
GCAGCACCGAAGCTAGAACAAGAAGCAGCTACAGGAGCTTCATGGAACCCGGCAGTACAACAA
GCACCAACACTGGTGCGAACTTCATCAGCGCACAAGTCAACACAAGAAGCAGGAGGGGCTTCG
TCCAGGCCTCGCAGCTGCTGTTGCCTAGGAGGAGCGCAGTAAGGTTGGACCAATCCGAAGATG
CTGCGTTGGCTTACAGGTTGCAACAGGAGGAGTTCATGAATGCCTTTGAGGAGCCTGAGCAGG
AGAGGCAGCAGCCACGGAACGCCGTGTCCACAGCACGAGACACTCTGAGAGCCATCGCCTCC
CGAGCCGTCCGCCTCCGTGCTCGAGGCTGGCATGTTTAA

>JL74_GRMZM2G462623
ATGGCCACCGCCCACCGGCTGAATACCTCCACTGCTCCACACGTCCCTGTGCCGCCGTATCTT
GGTGCGCTCTTGCTCCTAGGTAACATACATCTCTGTCCCTCCCTAGGAGACCGAGACAGCACCG
TAATGGCGTCCCCCTGCGGCGATGCTGCGGCGGCAGCCGAGCTCACCAGCCTGCGCATCAGC
GAAGGAGCTGGGATCCCTCCGCTTCCCGGACGGCGAAGCGTCGGCGGCAATGCAGCTGAGGG
AGAAGAGGAGGGTGATAAGAACAAGAAGGCGAGGAAGGGGAAGGCTGGTACGCAGCGGATCG
CCGGGTGGGGGCTCCGCGAGTTCAGCAAGATAGTTTCTAAAAAGGTTGAGACCAAAGGACGGA
CTACTTACAGTGAGGTTGCAGATGAAATTTGTGGAGAGCTGAAGTTGACTCTTATTGGTCAAGAG
TTTGATGAGAAGAATATCAGGAGGAGAGTGTATGATGCTTTTAATGTGCTAATTGCACTACGTGT
TATCACAAAAGATGAAAAAGAGATAAAATGGATGGGCTTGTCTAATTTCCAATATGAAAAGATAAA
GTTGGAGGAAACTCGCAAAGAACTCATGATCAGGATTAAGAACAAGAAAAAACTTCTCCAGGAA
ATTGAAAGACAGTTTGATGACCTCCAGAATATCAAGTTTCGCAACCAGTTTCTACAGAGGCCAGC
AGAGAGTGTGAATGGTATCTGCCTTCCATTCTTATTGGTCAAGGCATCCAGAAAAGCAAGGGTG
GAGATTGAGATCTCAGAAAACTCAAAGTTTGCTGGTTTGGACTTCAACTGGTACTACCAGAACTC
AAAACTGATGCTCCCTCCAAATTAA

>JL75_GRMZM5G850129
ATGATGCTGAGCGGGCACGGCGGCGGGAGGCGCCTGTTCACGGCGTCGCAGTGGCAGGAGC
TCGAGCACCAGGCGCTCATCTTCAAGTACATGGCCTCGGGCGCGCCCGTGCCGCACGACCTC
GTCCTACCGCTCCGCCTCGCCACCGGCGTCGACACCGCGCCCTCCCTCGCCTTCCCGCCGCA
GCCTTCGCCGTCGCTGGCGTACTGGGGCTGCTACGGCGCGGGGGCGCCGTTCGGCCGCAAG
GCGGCGGAGGACACGGAGCCGGGGCGGTGCCGGCGGACGGACGGCAAGAAGTGGCGGTGC
TCCAGGGAGGCCCACGGCGACTCCAAGTACTGCGAGAAGCACATTCACCGCGGGAAGAGCCG
TTCAAGAAAGCCTGTGGAAGTGACCTCCTCCCCCGCCGCCGGCCCCGCTGCGGCGTACCGAC
CGTCCGCGATCTCCACCATCTCGCCGCCCCGCGCGGCCGACGCGCCGCCGCCGAGCCTCGCC
TACCCGCAGCAGCATCTCCTCCACGGCGCCTCCTCCGCAGCAGGAGCAGCAGCCCGCGTCCC
CGCTGGCGCTCTCCAGCTCCACCTCGACGCGAGCCTGCACGCGGCGGCGGCGGCGGCGTCG
CCATCGCCGCCGCCGTCCTACCACAGGTACGCCCACTACACACCGCCAGCGTCGTCGCTCTTC
CCGGGCGGCGGCTACGGCTACGACTACGACTACGGGCAGTCCAAGGAGCTCAGGCGACGGCA
CTTCCACGCGCTCGGGGCCGACCTGAGCCTCGACAAGCCGCTGCCCGAGCCCGACACCGGCT
CCGACGAGAAGCAGCCCCTGCGGCGTTTCTTCGACGAGTGGCCGCGGGAGAGCGGCGACATG
GCCGCGGACGACGCGACGCAGCTTTCCATCTCCATCCCCGCGGCTTCGCCCTCCGACCTCGCT
GCTACCTCCGCCTCCGCCGCCGCCGCGCGATTCCACAACGGGGAGTGA

>JL76_GRMZM2G061734
ATGGATTGGGATCTCAAGATGCCGGTCTCCTGGGACCTGCCCGACCTGGAGCACGACGCCATG
CCGCCGCCGCCGCCCGTCTCCGCGGCCTCCACCGCGGCGGCGTCGGGCATTGCCGCCGCAG
CTGCCGCGCCTTCCTCCGCGACGGCGCCGAGCCGGGCGGAGTGCTCCGTCGACCTCAAGCTC
GGCGGGCTAGGCGAGTTCGGGGCGGCGGACGGCACGGCCACGAAGGAGCCGGCGGCTGCAA
CCGCAGCTCCGTCCGCCAGCCCGATGAAGCGCCCGCGCTTGGGGCCCGGCGGCGCCGGCGG
GGCGCAGTGCCCGTCGTGCGCGGTGGACGGCTGCAAGGCCGACCTGAGCAAGTGCCGCGACT
ACCACCGCCGCCACAAGGTCTGCGAGGCGCACTCCAAGACGCCCGTCGTCGTCGTCGCCGGC
CGCGAGATGCGCTTCTGCCAGCAGTGCAGCAGGTTTCACTTGCTCCTAGAGTTTGATGAGGCC
AAGCGCAGTTGTAGAAAGAGGCTTGACGGGCACAACCGGCGCCGCAGGAAGCCGCAGCCAGA
TACCATGAACTCTGGGAGCTTTATGACAAGTCAACAAGGCTTGTTTTCGAGTGCAGGGACAAGG
TTCTCGTCGTTCCCGGCTCCAAGGCCGGAGCCAAGCTGGTCTGGAGTCATCAAATCCGAGGAC
AGCTCATACTACACACACCACCCGGTGCTCAGCAACAGGCCGCACGTCGCCGGCACCTCCACG
TCGCCGGCCTACTCCAAGGAAGGCCGGCGCTTCCCGTTCCTCCAGGACGGCGACCAAGTCAG
CTTCAGCGCATCAGGCGCCGGGACGCTCGAGGTCTCCACGGTGTGCCAGCCCCTCCTCAAGA
CGACCGCCGCCGTGGCCCCGCCGCCGCCCGAGAGCAGCAAGATGCTCGCCCCCGTGCTGGA
CTCGGATTGTGCTCTCTCTCTTCTGTCATCCCCCGCCAACTCTTCCAGCGTCGACGTGAGCCGG
ATGGTCCAGCCCGCGGAGCGCATCCCCATGGCGCAGCCCCTCGTCCCCGGCCTCCAGCACCA
CCAGTTCGGCGGCTCCCCGGTGCCGGACTGGTTCGCCGGCTCCGGCGCCGTCCCTGCCGCG
GGCACCGGCGGGTTCGCCTGCCCCCACAGCGTGGAGAGCGAGCAGTTCAACACAGTCCTAGT
GCCGAGCTCCGACGGCGGCCACGAGATGAACTACCACGGCATATTCCACGTCGGCGGCGAGG
GCTCCTCCGACGGGACGTCCCCTTCGCTCCCGTTCTCGTGGCAGTAG

>JL77_GRMZM2G318592
ATGGAGTTCGGCTTCCGCGCAGGAAACAGCACGCAGCGCCGCCCTTCCCCCGATCGACGCGT
CACAGCACAGCAAGCCGGACCCTTCCACGGCGACCACCCGCCGCCCTCGCCGTTGCAGTGGG
AGGCTGCGGCCCGGCGCGAGCGGATCAAACGCGAGGTGGAGCGCCGGCTCATCGAGGAGGA
GGTCCGCCTGGAGCTCGCCCTTGAGCGCGCCCGCCTCCACGGTGGTTTTGGCCCCGCCCCGT
TCCTCGGGACCGACGGCTCCGTCGTGCCGGTGCCGCCTCCACCTCCACCTCCAGGCCGGTTC
TCCAGGGCCGACGGCCCATCGATGACGCCGGCAATGTCGTGGGACAGCCCTCGGCGGCTCGC
GAGATTTGAGCAACCGATGCTCCTCAGTGAGACGGGAACGTGGTCCCTGCTGCCGAAGCCGAG
GCATACGCTCCGGCTGCGCGAGATCGCGCCCAGCGAGAGCTCCGAGGTTCTTTCATCAGCAAC
AAAGGCTTCTGGCGTGAAGAGGAAAGCAGCAGCTGCGATTTCCGCAACAACGACTGAACAACA
ACCAACGGGAGTACAAGACCCGAGTTCCGGGCAATGGAGCTGCGCAATCTGCCAGGTGAGCG
CGACCAGCGAGGCCAACCTGAACGAGCACCTCCAAGGGAAAAAGCACAGGGCAAAGCTGGCC
CGATGCGGAGCGACCAAGGCGACGACGGATCCTCCTCCTAACAGAAGCGGCGATGGCGCAGT
AGCAGTAGCAGCAGGCCCGAGCGACGCGCCGAAGAGGATCCAGATCCTGGTCGACGGGGAGG
CGCACCAGGTCGTCCAGAGGAGCAGCTGCGTGTGGTGCGAGCGCTGCAGGGTCGGCTGCACC
AACGCCGCCGCCATGGTCGACCATCTGCGGGGCAAGAGGCACAGCCTGTCGAACAAGGTCTG
GACGGCGATAAAGGCCGTGAGGAGGAGCAGCTAA

>JL78_GRMZM2G095899
ATGGAGGACTCGAGCCAGTTCATGCAGTGGGCCCTGAGCACGCTGCAGCACGATGAGCTCCCA
CCGGCGACGCCGCCGGCGGCCATCGCCTACGACGACAATGACTGCAACACCTTCTCATCAGTC
CCGGCGCTGGGGTACTCCGCTGCATCAGTCAACAGCATGGTCCCGGCGGAACCACCAGCACA
GGAAGGCCACCGGGCCACCGCCGCCACCAACAGCTGGAGCTCGGCCGACACCGCGTCCGTCA
CGGCCGCGCAGCGTGACGCCTGGTCGCCGTCTTCGCAGCAGAACTCGGTCAATTGTGCCACG
CCCCGCAGCAGCGGCAGCAGCCAGCCCGTGAGCTGGGACTTCCATTCGGCGTCGGCGTCGGC
GTCGGCGCAGCTAATAATCAAAGAAGCCCAGGTCAACTCTGCCACGGCCGCAAGGGCAGAGAG
CGCCGCTGGCGGCATGCCGGTGCCACAGCCACAGATGGTTCAAAACGGGTCGCCGCCGACGA
GGAGGGCCTCTGCCAAAATGTCGTCGGCTTCTTCGTCTGCGCCGCCGTGCTCCCAGGATCACA
TCGTCGCGGAGCGGAAACGCCGGGAGAAGATCAACCAGCGTTTCATTGAGCTCTCGGCAGTCA
TCCCCTGCCTCAAGAAGATGGACAAGGCGACTATCCTATCCGACGCGACGAGGTACGTGAAGG
AGCTCCAAGAAAAGCTCAAGGCACTGCAACAAGGCGGAAGCTGCAACGCCCGGGGCGGCACG
GAGTCGGCGCCGGTGCTTGTCAAGAAGCCGCGCATAGCGGCGCCGGGGGATGACGACAAGGA
CCGCGGCGGCGCGCCTTCCCCTTCCTGCGCGCCGCCTGGGGCTGCGGCGACCACCGGGAAC
GCGCTGCCGGAGATCGAGGCGAGGATCTCGGACGGCAACGTCGTGATGCTGAGGATCCACTG

CGAGGACGGCAAGGGGGTGCTCGTCAGGCTGCTGGCCGAGGTCGAGGGACTCCGCCTCAGCA
TCACGCATACCAATGTCATGCCCTTCTCGGCCTGCATCCTCATCATAAACATAATGGCAAAGGTG
GCGGAGGGCTTCAACGCCACGGCGGATGGCATTGTAGGGAGGCTTAATGCAGTGCTAGCAGCT
GGACCAACGTGTTAG

>JL79_GRMZM2G015666
ATGGCACTGGAGGCCGTGGTGCTATCCCAGTCCCAGTTCCAGCAAGGCAGTCGCTTTGGGTGC
GGTGCCATGGCAGCAGGAGGGGCTTGGAGTGATCTGTTGTTTAGTGGAACGGAGGGGTTGTTC
GAGATGGGTGGCGCCGCCGGCCGCGGCTGGAACGCGGCCGCGAGCTCGCCGCCGCAGCTCC
TGCAGGAGCTGGGTGACAACGGTGCTGCCGGCTCACTACCTGTGGCGTCTGCGGGGAGTGCG
AGTGGCGGCGCCGGTCAGGATGCCCCGGCAATGGCTGCGGCAGCGGCGTCCGGAAGGAGGA
AGCGGCGGAGGATGAGGCCAGTGAAGAACGAGGAGGAAGTGGAGAGCCAGCGGATGATCCAC
ATTGCGGTGGAGCGCAACCGCCGCAAGCAGATGAACGAGCACCTCGCCGCGCTCCGCTCACT
CATGCCGCCGGCCCACACGCAACGGGGCGATCAGGCATCCATCGTCGGCGGCGCGATCAACT
TCGTCAAGGAGCTGGAGCAGCTGCTGCAGTCGCTGGAGGCGCGGAGGCGTTCCCCGCAGTGC
GCTGCCTACGCCGTCGATCCCGACGACGCGGGGCCGTTCGCGGACTTCCTCACCTTCCCGCA
GTACTCGATGTGCGCCGTCATTGCGGCCCCAGAGAACACTGGCCACCACCGTGAGGGCGGCG
CCGTCGCCGAGCAGGAGGCGTCCGGGTCGAAGCCGTCGGCCGTGGCCGACGTCGAGGCGAC
AATGGTGGAGAGCCACGCCAACCTGCGGGTGCTGTCCCGGCGCCGGCCGAGGCAGCTGCTGC
GCCTGGTGCTGGGGCTCCAGGGCCACCGCCTCACCGTGCTCCACCTCAACATGAGCAGCGGC
GCCCACATGGTGCTCTACTCGTTTAGCCTCAAGGTGGAGGATGATTGCCAGCTTACCTCCGTGG
GTGAGATTGCGGCTGCGGCTCATCACATCGTCGAGAAGATTAATGAAGAGCAAAACAAGGCTG
CAGCTTAG

>JL80_GRMZM2G126018
ATGGCGACCGGCGGCGGCGGTGGGAGCGACGACTTCCACGGGCTCACGTTCGGCCAGAAGAT
CTACTTCGAGCAGGACGCGGGCGCCGCCGGGGGGAGCGCGACGGGCGGCAGGAAGGGGAA
GGGCGCCGCGGCGTCCGCGAGCGCCGCCCAGCCGCCGCGGTGCCGGGTGGACGGGTGCGG
CGTGGATCTGAGCGCCGTCAAGCAGTACTACTGCCGGCACAAGGTGTGCTACATGCACTCCAA
GGAGCCGCGCGTCTTCGTCGCCGGCATCGAGCAGCGCTTCTGCCAGCAGTGCAGCAGGTTCC
ACCAGTTACCTGAATTTGACCAAGGGAAACGAAGCTGCCGCCGCCGCCTCATTGGTCACAACG
AGCGCCGGAGGAAGCCACCACCTGGACCACTCACCTCACGATACGGCCCGCTTGCTGCATCAC
TAGTTCAAGAGCCTGGTAGGTTCAGGAGCTTCCTGCTCGACTTCTCGTACCCGAGGGTTCCAAG
CAGCGTGAGAGATGATGCGTGGCAATGGCAAGGGATGCAGCATGGCGGCGACAGGATGCTGC
TGGGTACCGCCAGCGCCATCCAGTGGCATGGGAACCAAGAACCTCACCCACCACATCGCAGCG
CAGCTGCGGGCTATGGCAACCATGCGTACAACAACTGCCATGGTGGCGGCGGCTTGGTAGCAG
CAGCAGCGGGGGCGTCAATGCTCTCCTCTGCCTTCGAGCCCCCGCCTGGCGGTGGCGGATGT
GTCGCAGGAGCTACCGCGGCCGACTCCAGCTGTGCTCTCTCTCTTCTGTCAACTCAGCCATGG
GATACGCCCACCCAAAGTGGCAGCAGCCACCACAACCGGTCCCCAGCGATGTCCGCGGCTGC
AGGCGCCTTCGAAGTGGCGCCGTCGTCCGTCGTGGCGAGCAGCTACACGGCGTCGAGCTCCT
GGTCTGGCTCACGGGACCCTGCCGACGGCGCCAGGAAAAACGCGCAGCGTCACGATGATGGC
CTGCACCTGGTCCATCCACCAGGCCCCGTCCACCACGGCCACTTCTCCGGCGAGCTCGAGCTC
GCCCTGCAGGGAAGCGGCCCGCCGCATGTCGACCATGGCGGCGGCACCTGCTTCAGCCATTC
TGACACCAACGCGATGAATTGGTCCCTGTAG

>JL81_GRMZM2G312419
ATGGCCGCCTTCTTCGGTGCTGCACCGCCGCCGGCTCTCGCTTCGTTCCGCCACGAGGAGGA
GCAGCATCAGGACGCCGCCGAGGAGGTGTACCACGTGGACGATGTCGACGAGGGCAGCGGTG
ACGGCCGTGGCCACGGCGGCGCCGGCCAGGGGAAGCTGTGCGCCAGGGGCCACTGGCGCCC
CGCCGAGGACGCCAAGCTCAAGGAGCTCGTCGCTCAGTTTGGGCCCCAGAACTGGAACCTCAT
CGCCGAGAGGCTGGACGGCAGATCAGGGAAGAGCTGCCGCCTGCGGTGGTTCAACCAGCTGG
ACCCGCGGATCAACCGGCGCGCCTTCTCGGAGGAGGAGGAGGAGCGGCTCCTGGCGGCGCA
CCGCGCGTACGGCAACAAGTGGGCTCTCATCGCGCGCCTCTTCCCGGGCCGCACCGACAACG
CCGTCAAGAACCACTGGCACGTCCTCATGGCGCGCAGGCAGCGGGAGCACTCGGGCGCGCTC
CGCCGCCGCAAGCCCTCCTCGACCTCGCCCTCGCCGGCGCCGGCGCCGGCGCCGCACCTGG
CGCCCGTCGTCGTCCTCCACCACCACCACCACTACGCCGGCGGCTCCCCTCCCATGCCGCCGT
TGCACGCCGCCGACGCCCACACGCAGTACACGGCGGCCGCGGCGGACACCCGCGCGCACAG
CGGCGGCGAGTCCGACGAGACCGCCTCCACGTGCACCACCGACCTTTCCCTCGCCTCCGCCG

CCCCCTGCTTCTACCAGAGTGGCTACGACGTGGCCCCGCGCGCCGCGGCGTTCGCACCGAGC
GCGCGCTCCGCCTTCTCCGCGCCGCCGGCGACGGCGCGCTCCGACGACAAGGTCTCGCTGCC
CTTCTTCGACTTCCTCGGCGTCGGCGCGGCGTGA

>JL82_GRMZM2G478876
ATGGACGCCGCCGGGCCGCCATCGGCAGCCAGTGGCTCCGGCTCCAACTCCAAGACCTCCCT
CCTCGGCATGCCGGTTCCCATCGCGATGGCCGCCGCCGCGGCACTCGTCGTCCTGCTCGTCC
TCTCCGCGGTTGTCGCCGCTGTTCTACTCGCGCGCAGGCGGGGCGCAAGGCCGCCCTCGCTG
TCGCGCGTCGAGCACGCGCCGTCCTCCGCCTCCGCCTCCGCCTCCGGCTCCTCCCGCGCCGC
GTCGTCCGCGCGCAGGGAGAAGGTCGGCGCGGACCAGGTCGCCGGGGCGGGCGCCACCTCC
TCATCTGGCGTTGCCAGCTCCTCCGCGGCCGCGAGCTCGCTCGAGTCGCCGGTGAAGAGGAA
GGCGGAAGCGGTCAGGGCCGCGGCGGGCGTGGACATGGAGATGGAGATGGGGTGGGGGAG
GTGGTACGACCTCACGGAGCTGGACGTCGCCACGGGCGGGTTCTGCCCAGAGAACGTGGTCG
GGGAGGGAGGCTACGGCACCGTGTACCGCGGCATCCTTGCCGGCGGCGAGGTCGTCGCGGT
CAAGGACTTGTTTGATCACAAGGGTCAGGCCGAGAAGGAATTCAAGGTGGAGGTTGAAGCCAT
CGGCAAAGTGAGGCACAAGCACCTTGTCGGTCTCATCGGGTACTGCGCAGAAGGCCCTAAACG
GATGCTCCTTTACGAGTTCGTCGAAAACGGGAACCTGGAGCAGTGGCTGCACGGCGACGTCGG
TCCGGTCAGCCCTTTGACCTGGGAAATACGGATGAAGATCGCCGTCGGAACGGCCAAAGGCAT
AGCGTACCTGCACGAAGGCCTGGAACCGAAGGTGGTGCACCGCGACATCAAGTCGAGCAACAT
CCTGCTGGACAAGAAGTGGAGCCCCAGGGTGTCGGACTTCGGCATGGCCAAGGTGCTGGGGT
CCGGGTCCAGCTACGTGACGACCCGCGTGATGGGCACGTTCGGGTACGTGGCCCCCGAGTAC
GCGTCCACGGGCATGCTGAACGAGAGCAGCGACATCTACAGCTTCGGCGTGCTGCTCATGGAG
CTCATCTCCGGGCGCAGCCCCGTGGACTACAACCGCCCCGCCGGCGAGGTCAACCTGGTGGA
GTGGTTCAGGGGCATGGTGGGCAGCCGGCGCGTGGAGGACCTGGTGGACCCGCGCATCGCC
GCGCCGCCGCCGCCGCGGGTGCTCAACCGGGTGCTGCTCGTCTGCCTCCGCTGCATCGACGC
CGACGCGCACAAGCGGCCCAAGATGGGGCAGATCGTGCACATGCTCGAGGGCGACGAGTTCC
CCTTCCGCACGGAGCACCGCTCGCCGAGGGCGTCTCACCGGACGTCGATGGCCACGCGGCCG
TCGCTTCTGTCGGAGAACGTAGGTGCCACTGACGACTCAGACAAGTCCATGTGGAGATGA

Information S2. Individual datasheets for T0 rice lines summarized in Dataset S2. Each page shows the gene expression profile in maize foliar and husk leaf primordia as reported in [1] (green circles refer to relative expression level in samples of the shoot apical meristem plus plastochron 1/2 leaf primordia, orange circles to levels in P3/4 leaf primordia and blue circles to levels in P5 leaf primordia); construct composition and ID; number and ID of independent transgenic rice lines. Cropped agarose gel images show PCR products of representative genomic PCR reactions (detecting presence of transgene). Linear regression plots illustrate number of veins versus leaf width in each line (x axis = leaf width in cm; y axis = vein number). Representative leaf samples are shown with views of the adaxial leaf surface taken using a hand-held microscope.

GRMZM2G123900 **ZnF DoF** **Gene ID: JL44**

Foliar expression

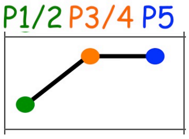

Husk expression

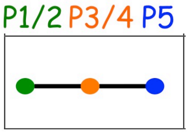

Construct: $ZmUBI_{pro}$:cDNA (ID# IRS1085)

Nineteen independent T0 lines:
IR64-IRS1085-001 to IR64-IRS1085-019

Genomic PCR

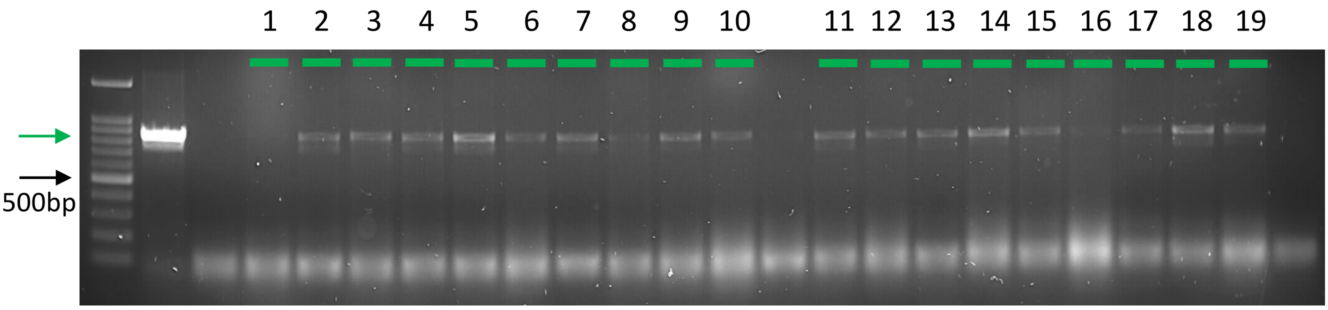

Number veins versus leaf width

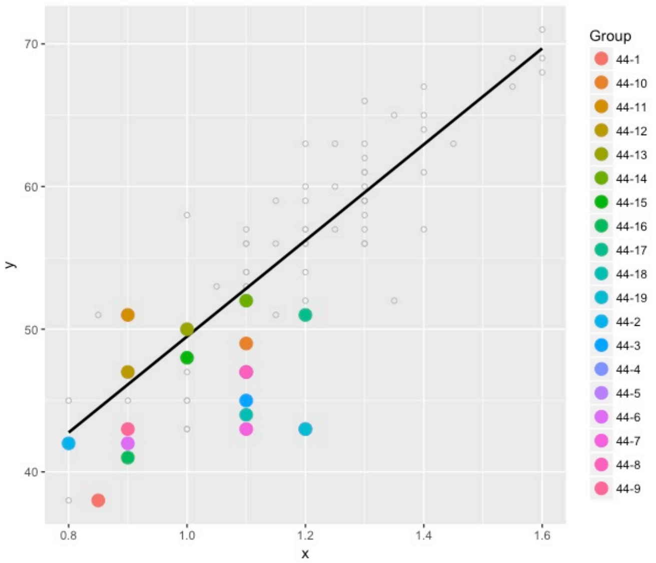

Representative surface image of segregating WT leaf

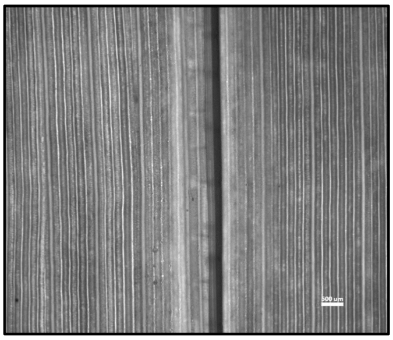

Representative surface image of line 15 leaf

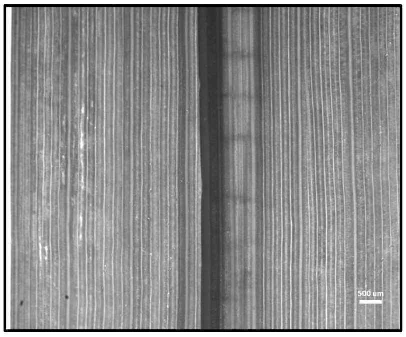

GRMZM2G374986 **Myb** **Gene ID: JL45**

Foliar expression

P1/2 P3/4 P5

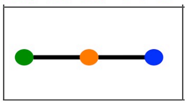

Husk expression

P1/2 P3/4 P5

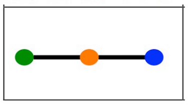

Construct: ZmUBI$_{pro}$:cDNA (ID# IRS1086)

Thirty five independent T0 lines:
IR64-IRS1086-001 to IR64-IRS1086-035

Genomic PCR

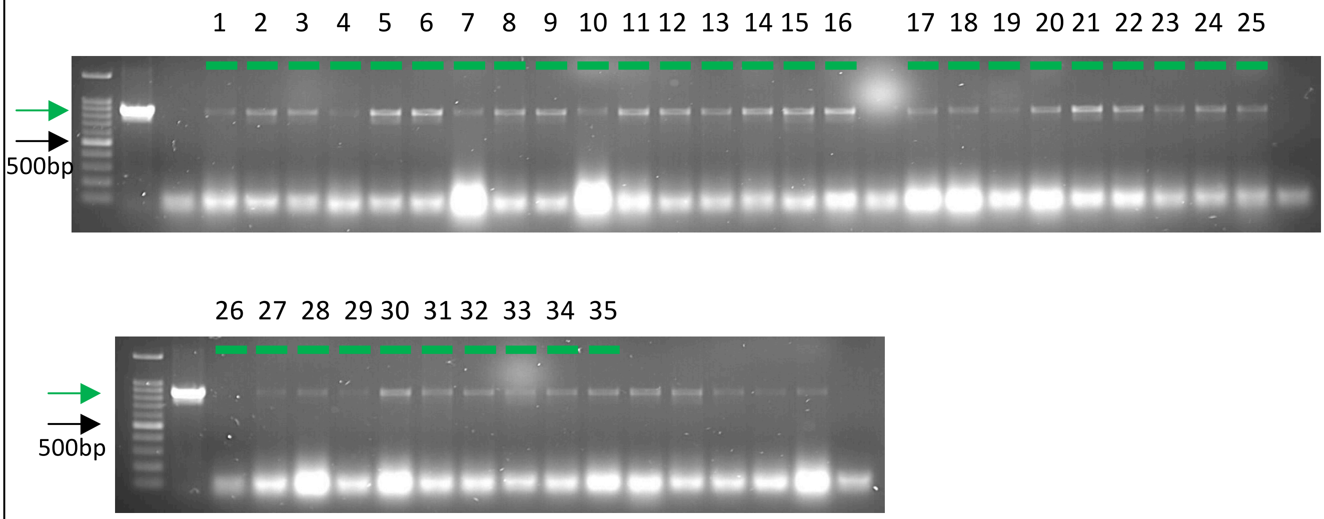

Number veins versus leaf width

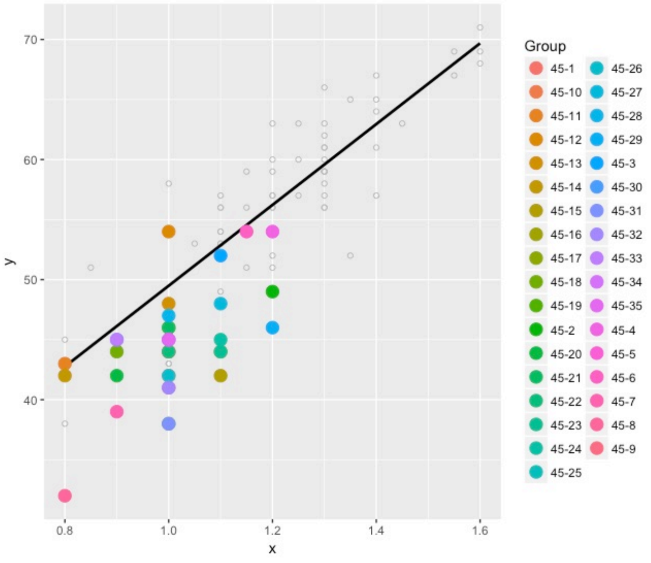

Representative surface image of segregating WT leaf

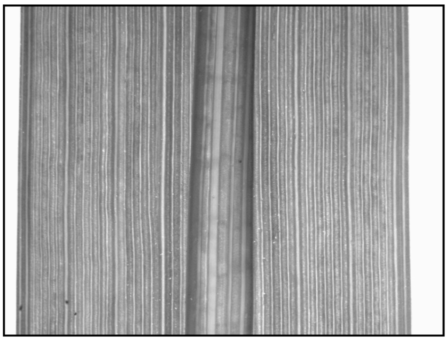

Representative surface image of line 35 leaf

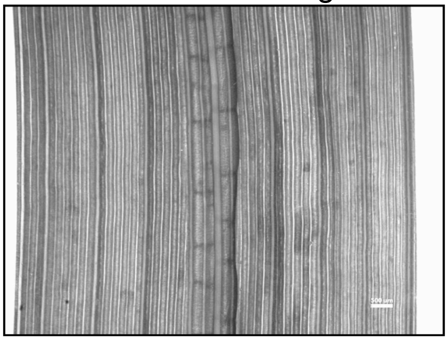

GRMZM2G163975 **AP2 domain** **Gene ID: JL53**

Foliar expression

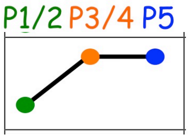

Husk expression

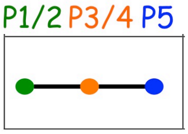

Construct: $ZmUBI_{pro}$:cDNA (ID# IRS952)

Twenty independent T0 lines:
IR64-IRS952-001,002,005,007,008,013,014,017-020,023-025,028-031,034,035

Genomic PCR

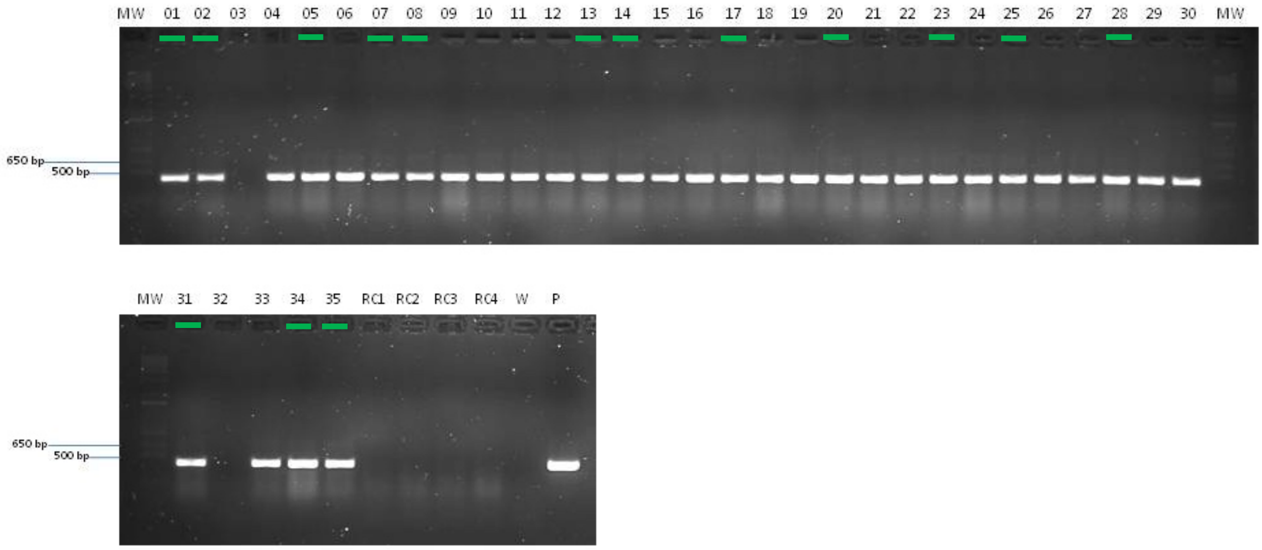

Number veins versus leaf width

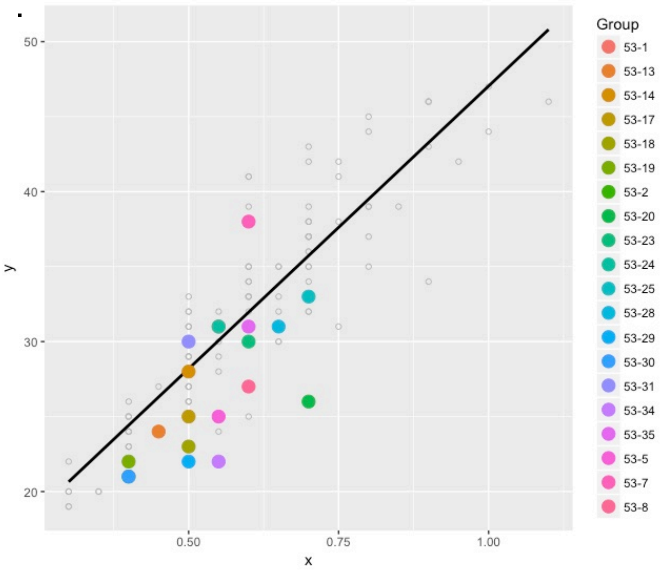

Representative surface image of segregating WT leaf

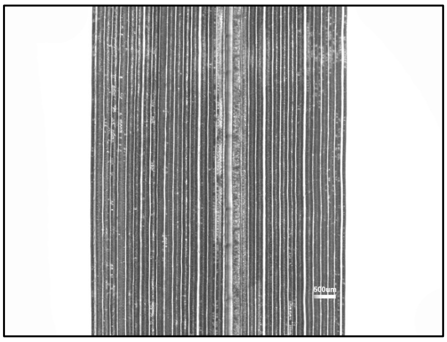

Representative surface image of line 14 leaf

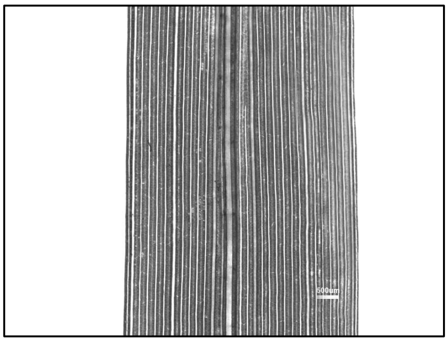

GRMZM2G077219 **unknown** **Gene ID: JL54**

Foliar expression

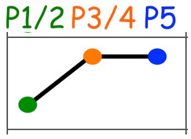

Husk expression

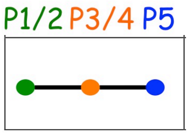

Construct: ZmUBI$_{pro}$:cDNA (ID# 1010)

Seventeen independent T0 lines:
IR64-IRS1010-002-004,007-009,015,016,018,019, 021,022, 028-030, 034,035

Genomic PCR

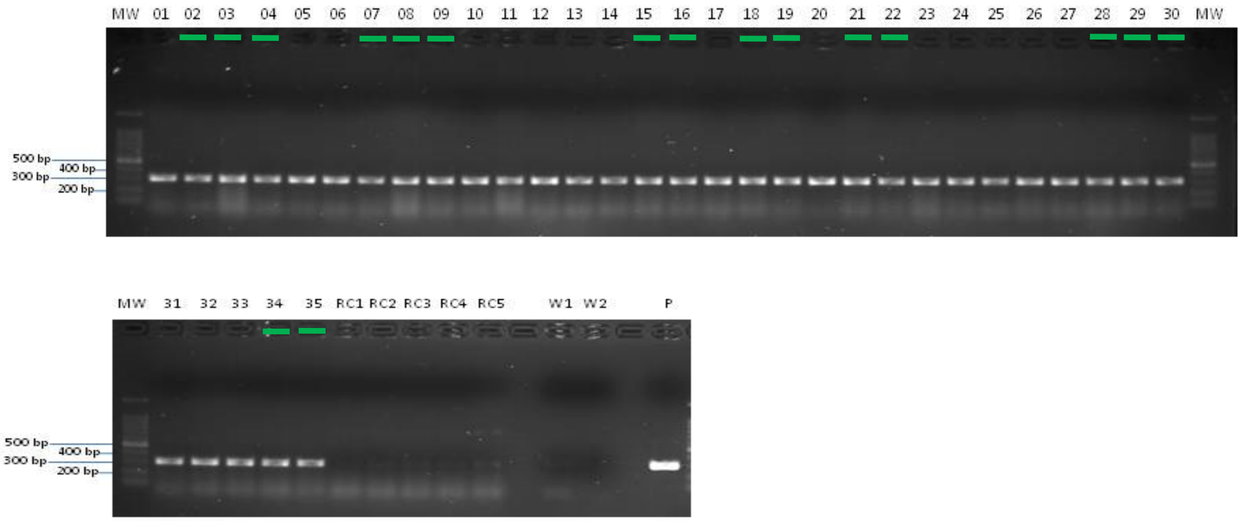

Number veins versus leaf width

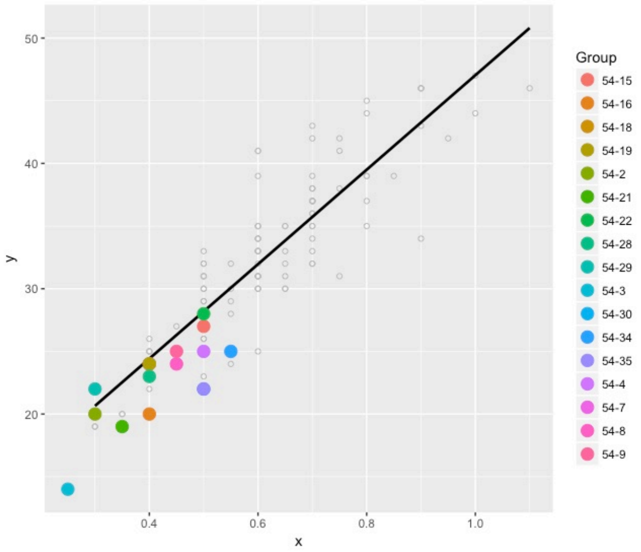

Representative surface image of segregating WT leaf

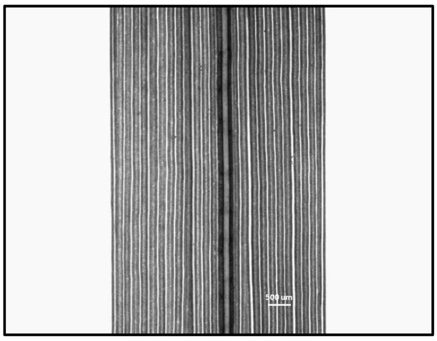

Representative surface image of line 19 leaf

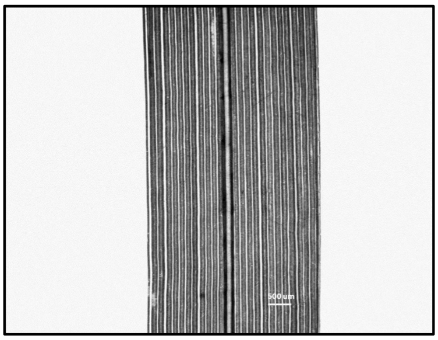

GRMZM2G082586 **bHLH** **Gene ID: JL55**

Foliar expression

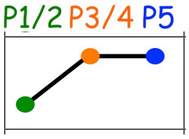

Husk expression

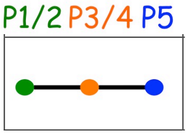

Construct: ZmUBI$_{pro}$:cDNA (ID# 878)

Ten independent T0 lines:
IR64-IRS878-001–009, 011

Genomic PCR

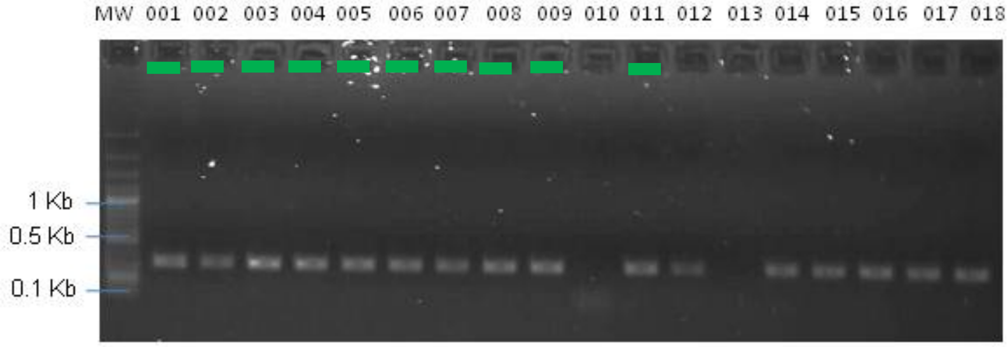

Number veins versus leaf width

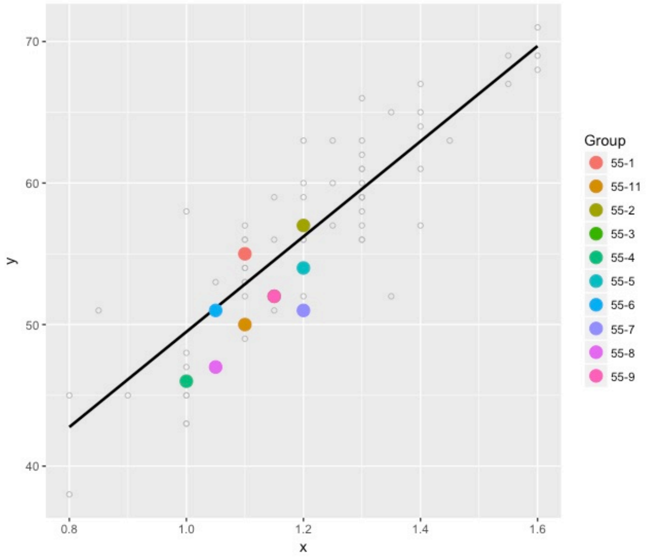

Representative surface image of segregating WT leaf

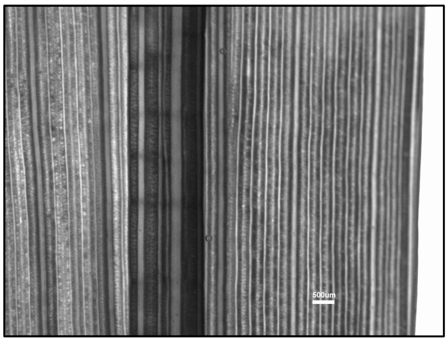

Representative surface image of line 1 leaf

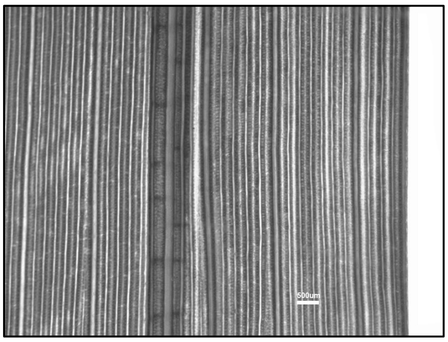

GRMZM2G171365 **MADS box** **Gene ID: JL56**

Foliar expression

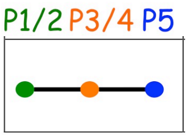

Husk expression

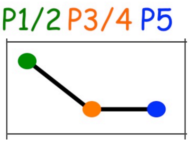

Construct: ZmUBI$_{pro}$:cDNA (ID# 921)

Thirty four independent T0 lines:
IR64-IRS921-001-004,006-035

Genomic PCR

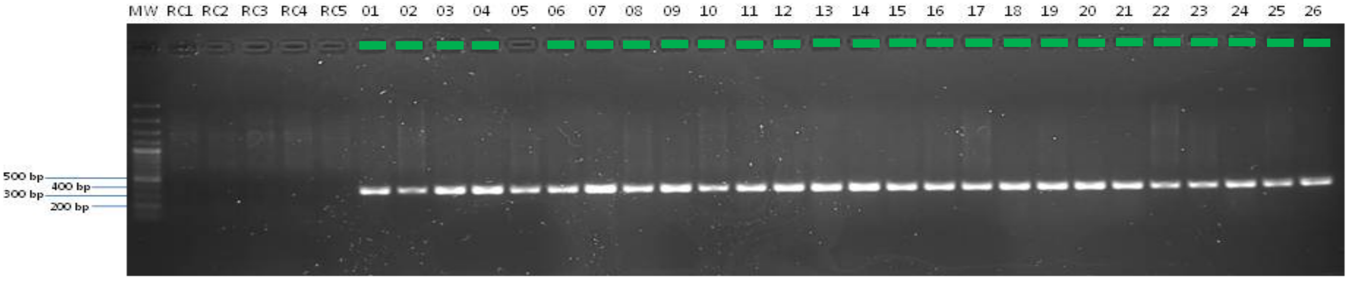

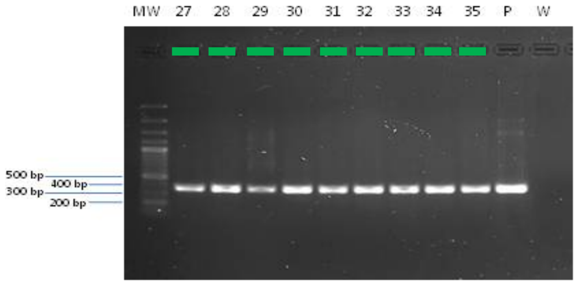

Number veins versus leaf width

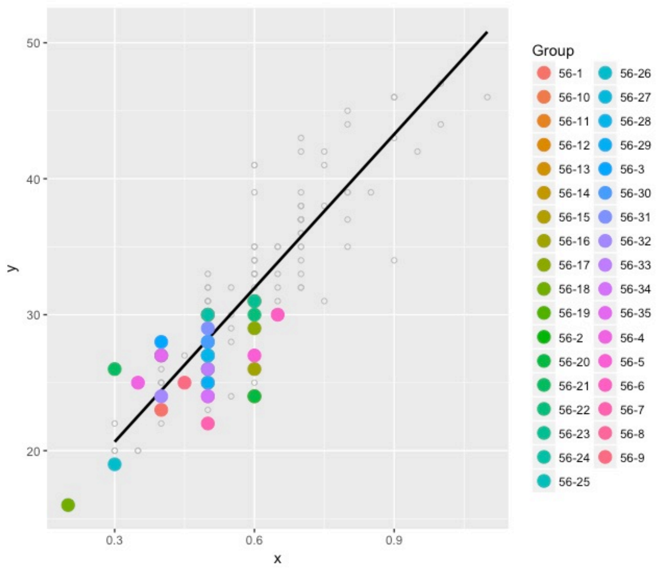

Representative surface image of segregating WT leaf

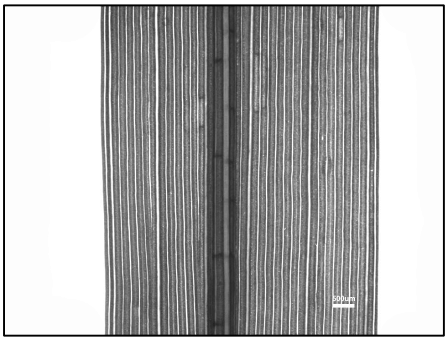

Representative surface image of line 30 leaf

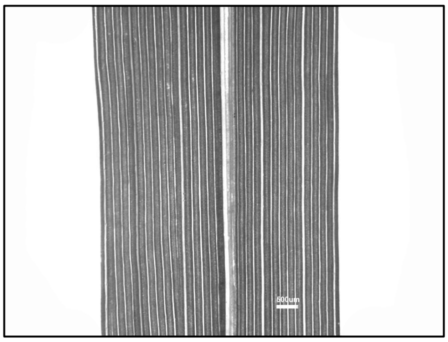

GRMZM2G098813 **LFY-like** **Gene ID: JL57**

Foliar expression

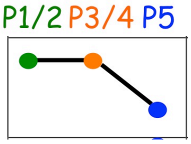

Husk expression

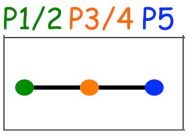

Construct: ZmUBI$_{pro}$:cDNA (ID# 922)

Thirty four independent T0 lines:
IR64-IRS922-001-033, 035

Genomic PCR

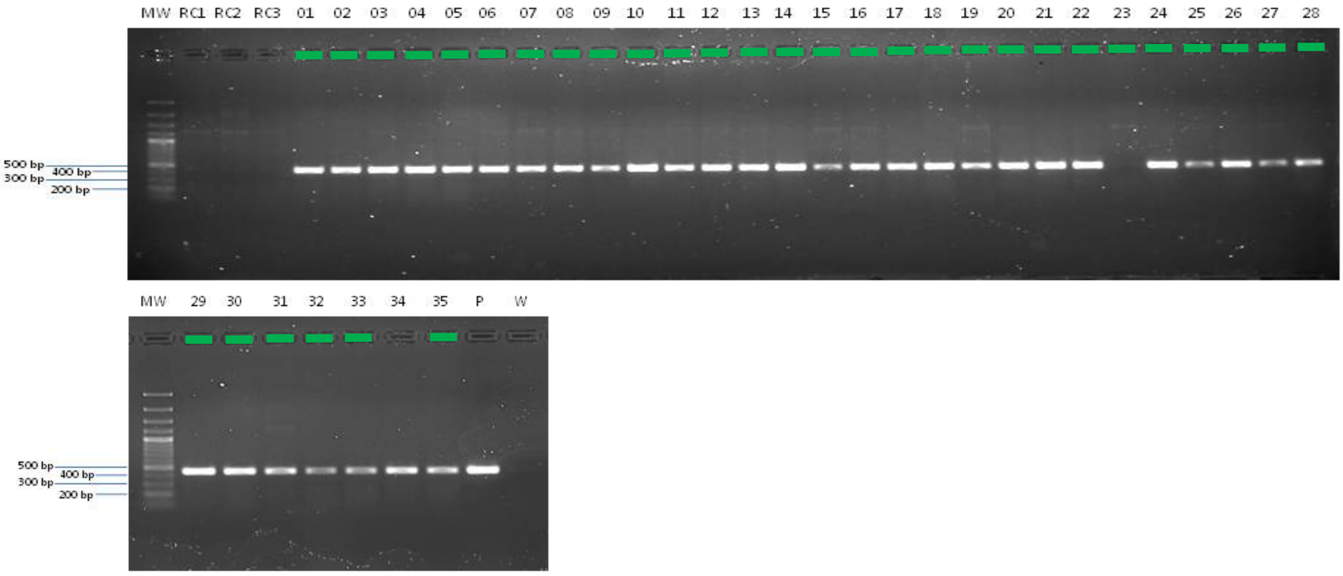

Number veins versus leaf width

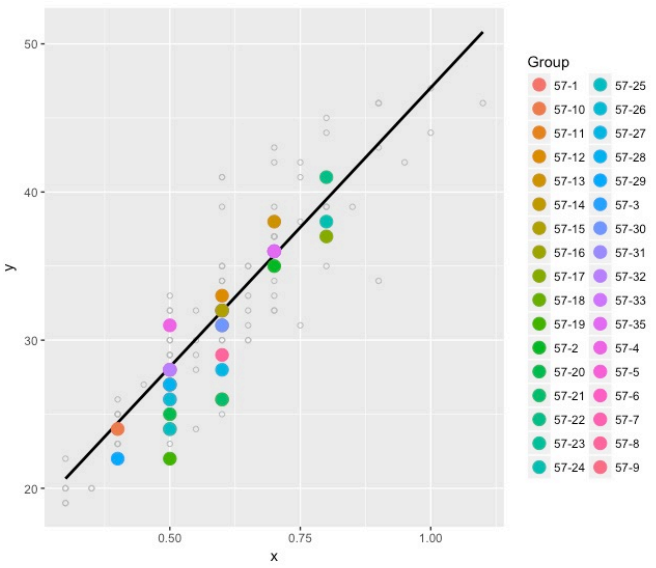

Representative surface image of segregating WT leaf

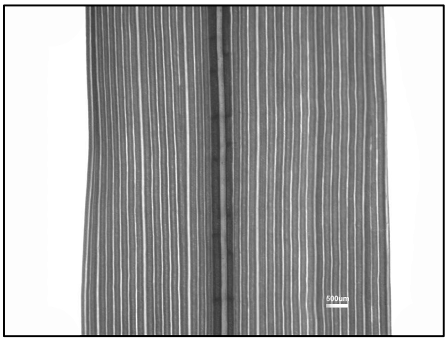

Representative surface image of line 12 leaf

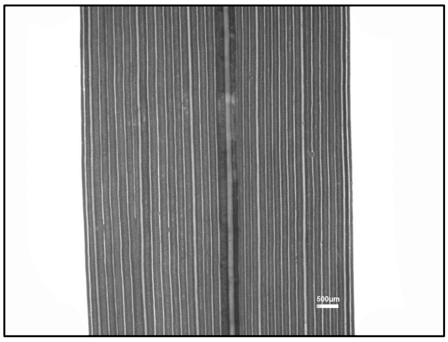

GRMZM2G139324 **unknown** **Gene ID: JL59**

Foliar expression

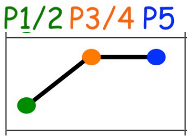

Husk expression

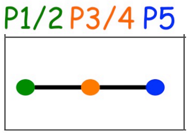

Construct: ZmUBI$_{pro}$:cDNA (ID# 1004)

Thirty five independent T0 lines:
IR64-IRS1004-001–035

Genomic PCR

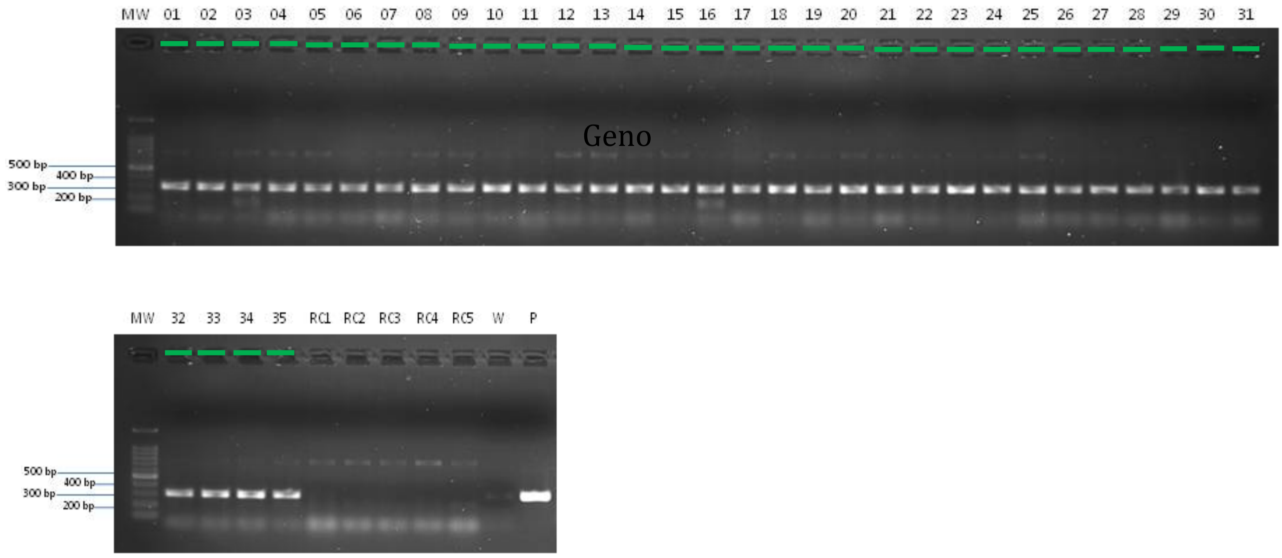

Number veins versus leaf width

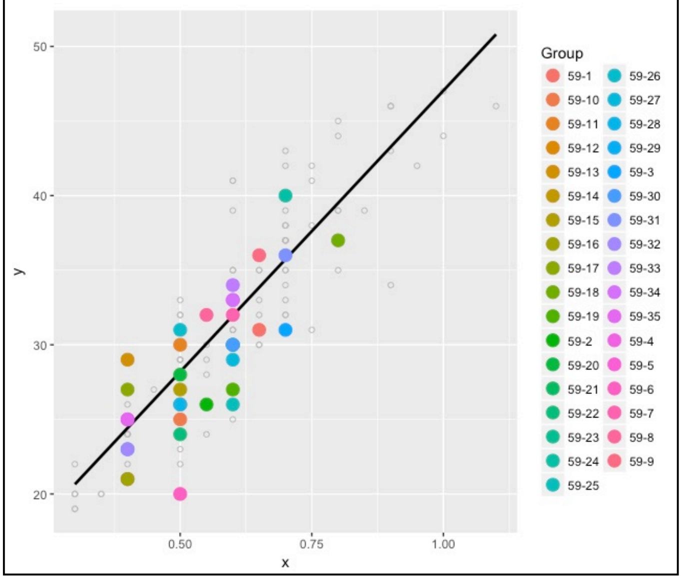

Representative surface image of segregating WT leaf

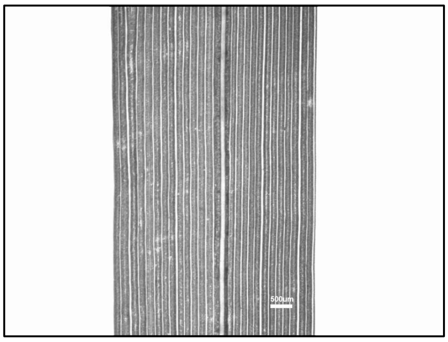

Representative surface image of line 5 leaf

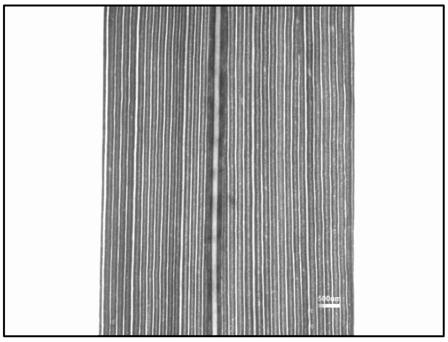

GRMZM2G061314 **LRR binding protein** **Gene ID: JL60**

Foliar expression

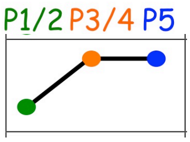

Husk expression

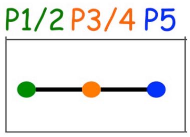

Construct: ZmUBI$_{pro}$:cDNA (ID# 945)

Twenty eight independent T0 lines:
IR64-IRS945-001–028

Genomic PCR

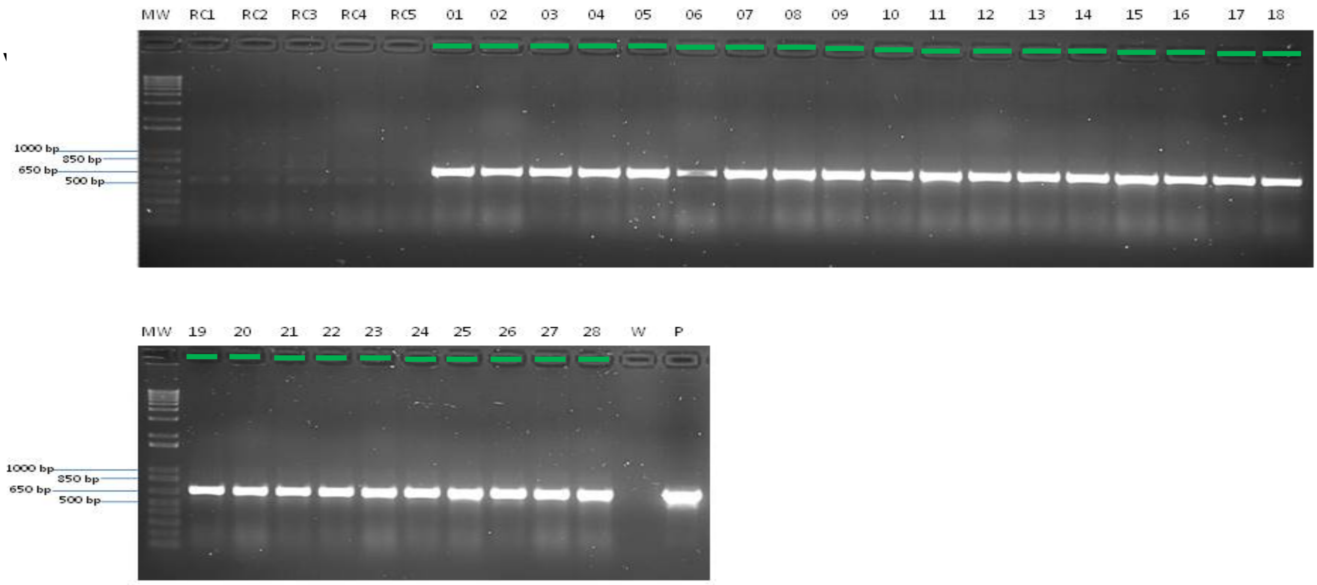

Number veins versus leaf width

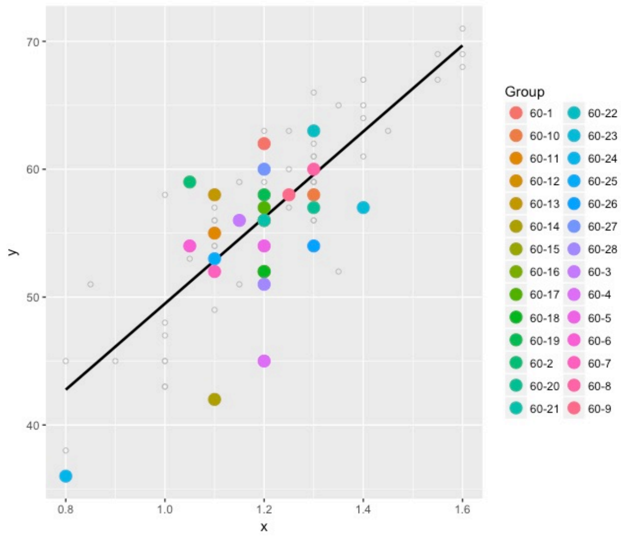

Representative surface image of segregating WT leaf

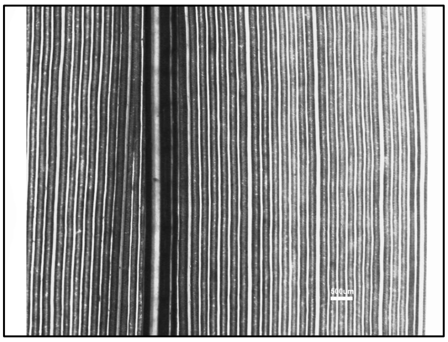

Representative surface image of line 16 leaf

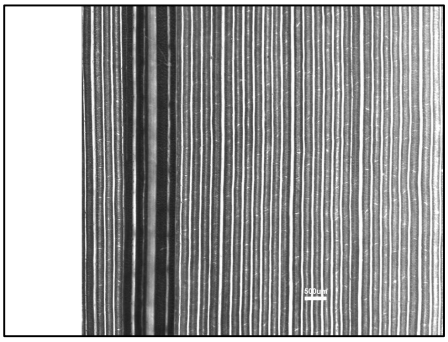

GRMZM2G151955 **LRR RLK** **Gene ID: JL61**

Foliar expression

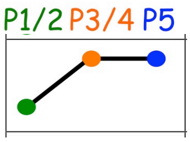

Husk expression

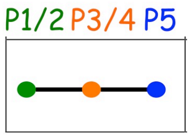

Construct: ZmUBI$_{pro}$:cDNA (ID# 1078)

Thirty four independent T0 lines:
IR64-IRS1078-001–009, 011-035

Genomic PCR

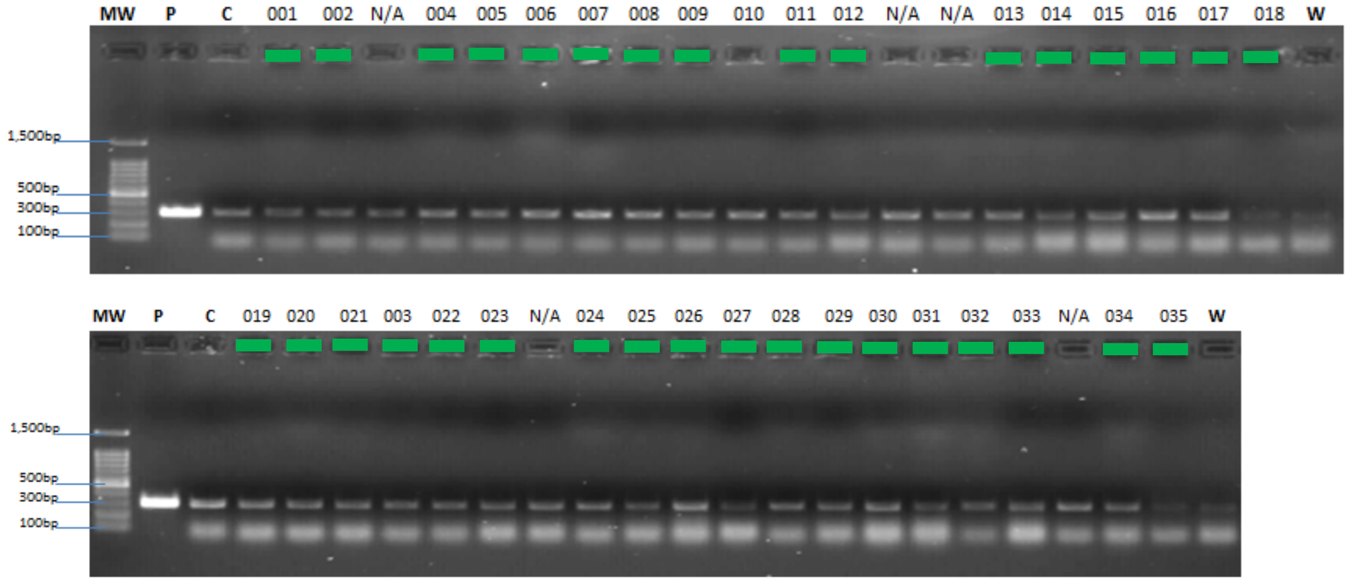

Number veins versus leaf width

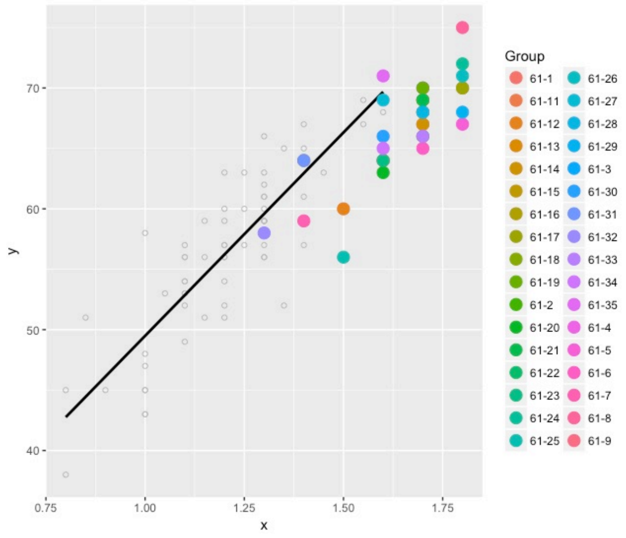

Representative surface image of segregating WT leaf

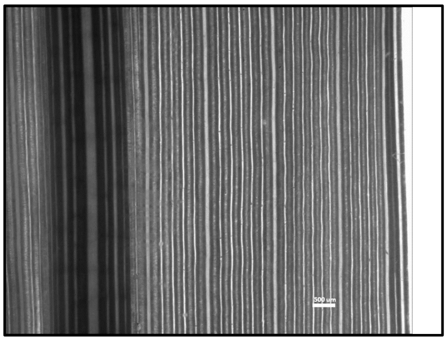

Representative surface image of line 3 leaf

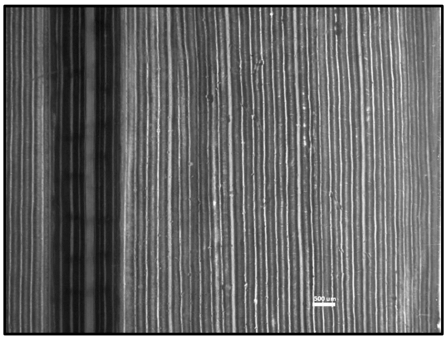

GRMZM2G159953 **Lectin family RLK** **Gene ID: JL62**

Foliar expression

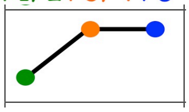

Husk expression

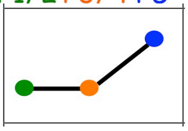

Construct: ZmUBI$_{pro}$:cDNA (ID# 913)

Ten independent T0 lines:
IR64-IRS913-009–016A, 017B, 018

Genomic PCR

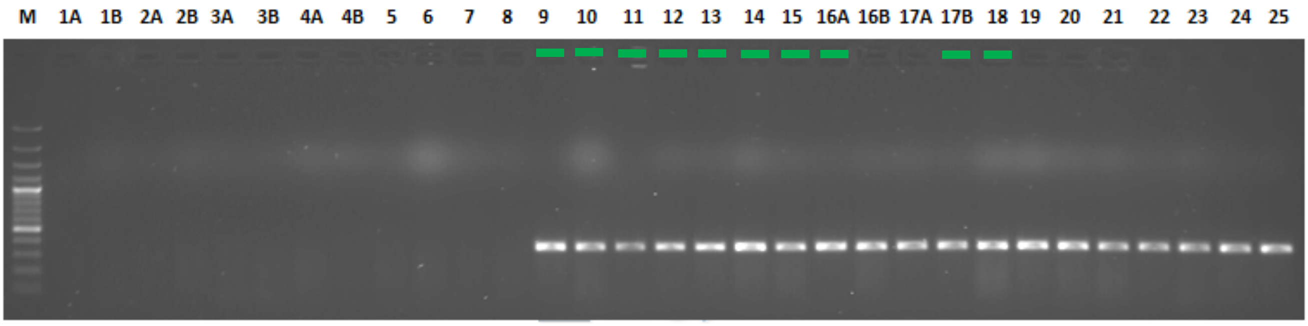

Number veins versus leaf width

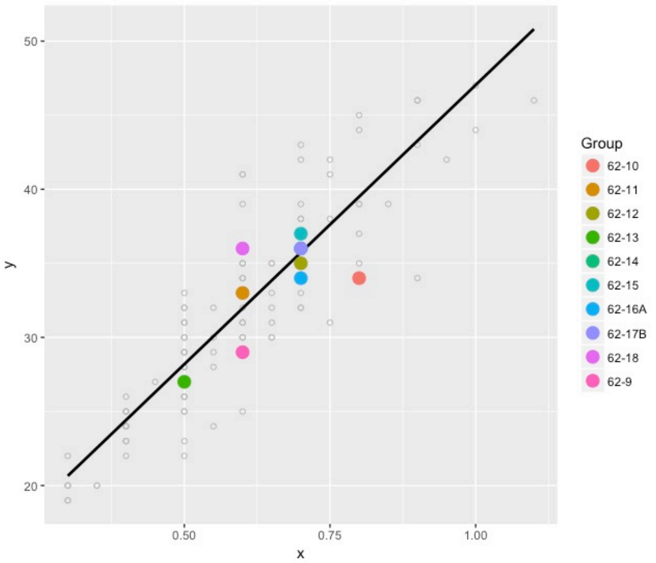

Representative surface image of segregating WT leaf

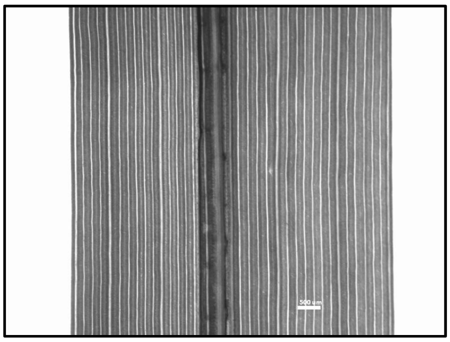

Representative surface image of line 12 leaf

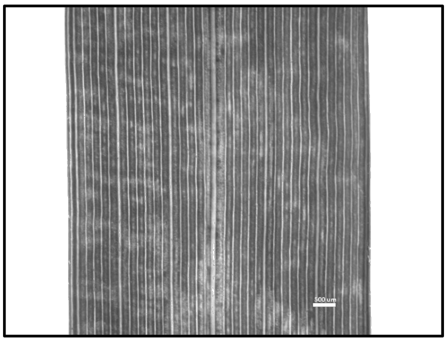

GRMZM2G039934 **TDR/PXY LRR RLK** **Gene ID: JL63**

Foliar expression

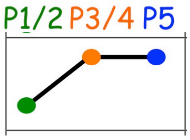

Husk expression

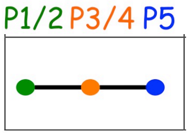

Construct: ZmUBI$_{pro}$:cDNA (ID# 1011)

Thirty five independent T0 lines:
IR64-IRS1011-001–035

Genomic PCR

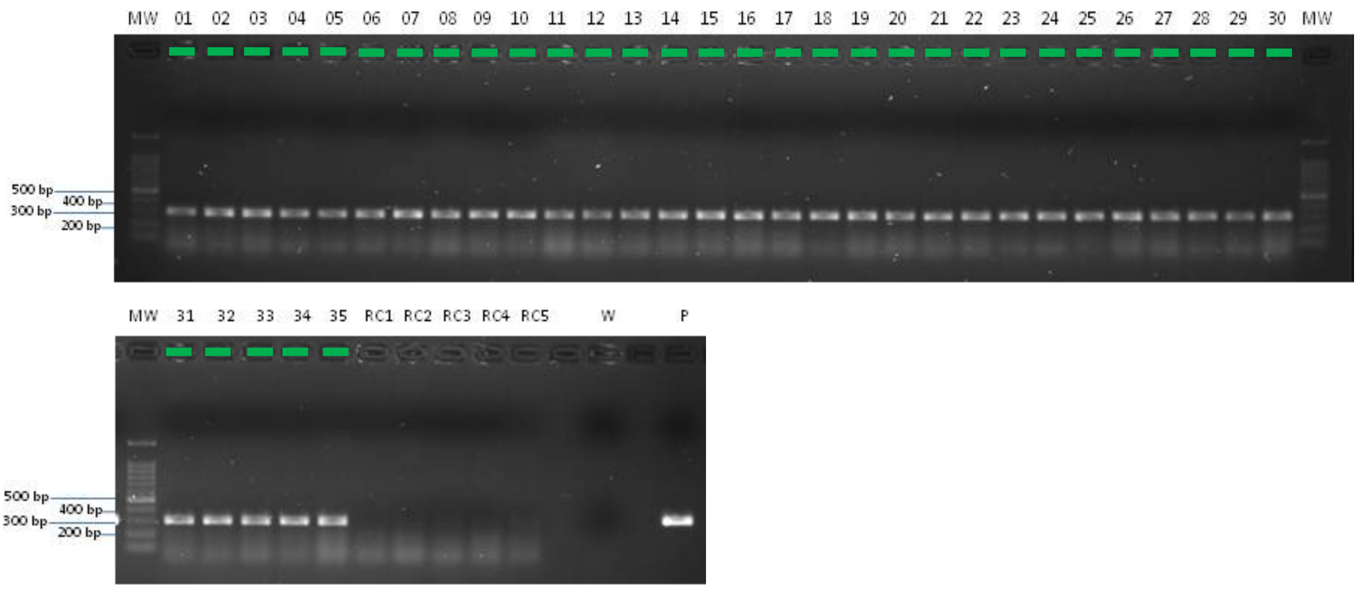

Number veins versus leaf width

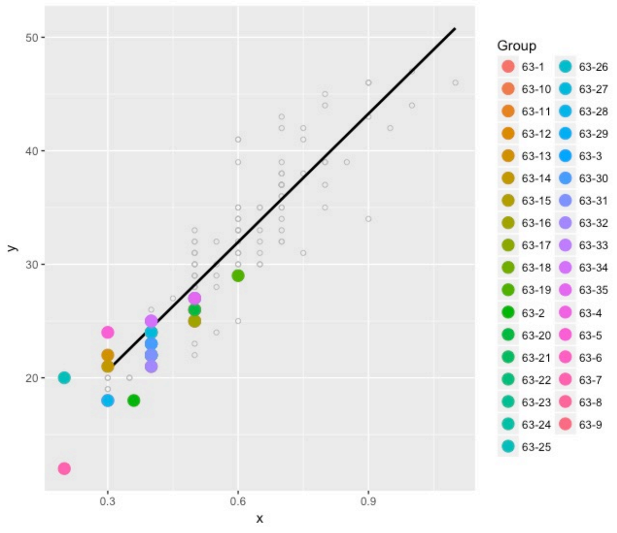

Representative surface image of segregating WT leaf

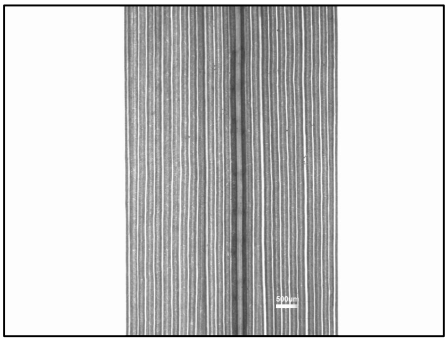

Representative surface image of line 23 leaf

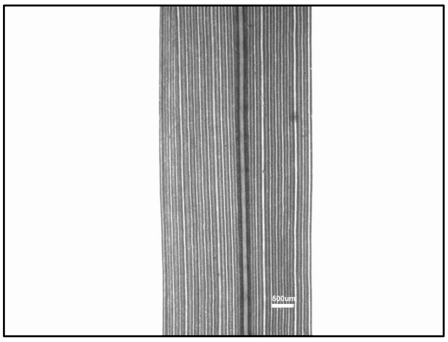

GRMZM2G046316 **LRR-RLK** **Gene ID: JL64**

Foliar expression

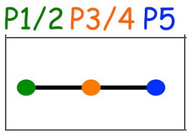

Husk expression

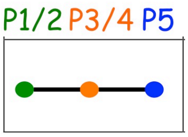

Construct: ZmUBI$_{pro}$:cDNA (ID#1106)

Thirty five independent T0 lines:
IR64-IRS1106-001–035

Genomic PCR

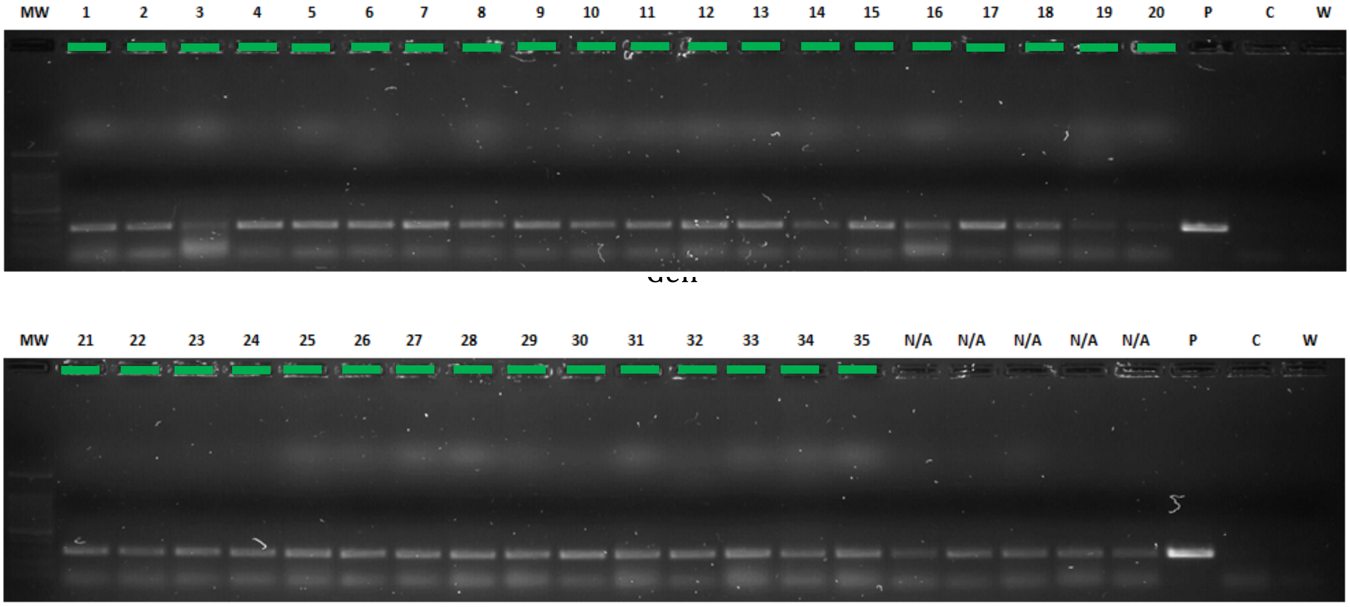

Number veins versus leaf width

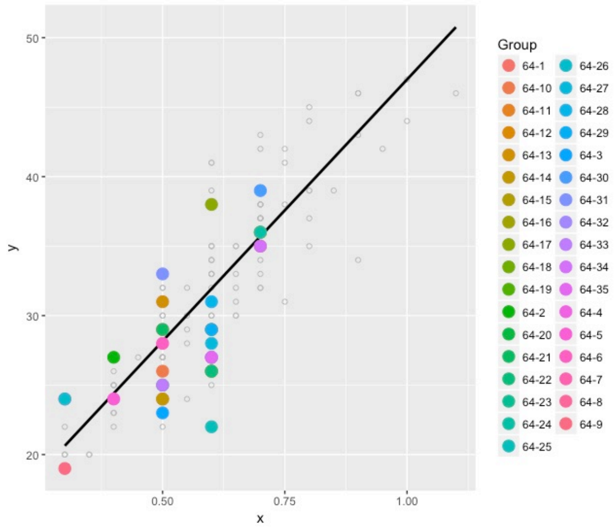

Representative surface image of segregating WT leaf

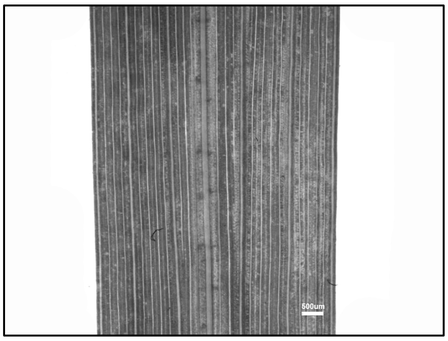

Representative surface image of line 23 leaf

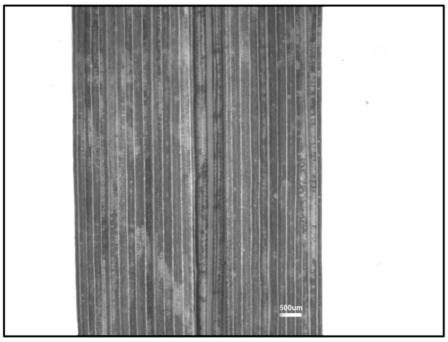

GRMZM2G034155 **LRR-RLK** **Gene ID: JL65**

Foliar expression

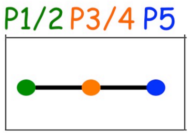

Husk expression

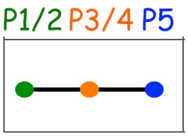

Construct: ZmUBI$_{pro}$:cDNA (ID# 1005)

Thirty five independent T0 lines:
IR64-IRS1005-001–035

Genomic PCR

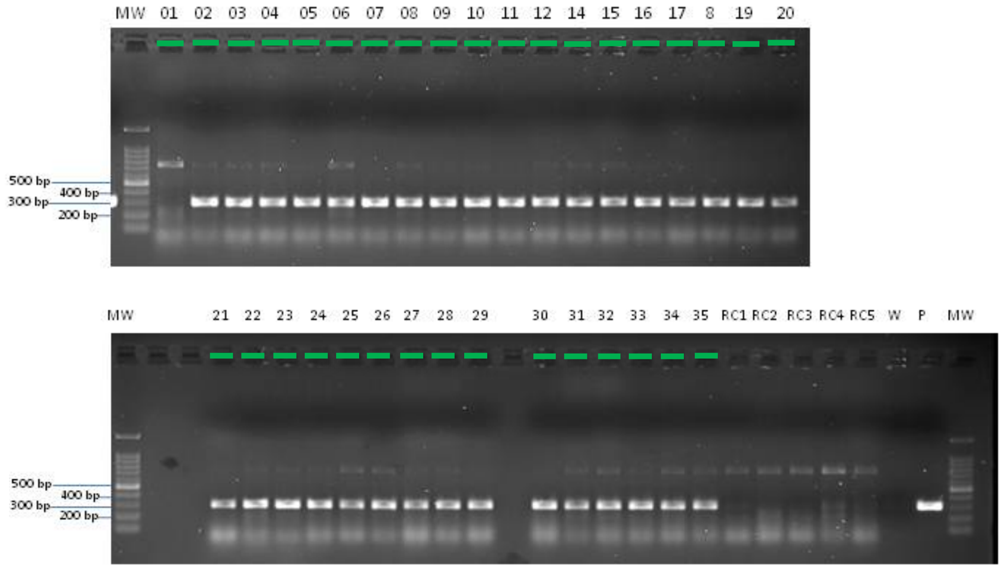

Number veins versus leaf width

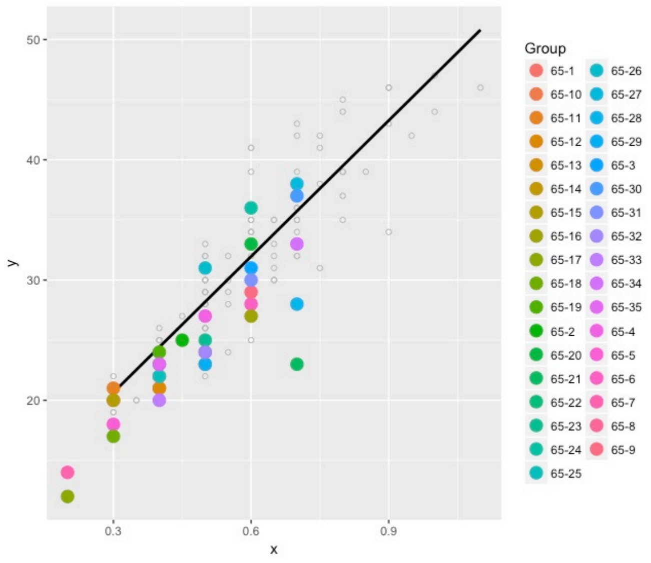

Representative surface image of segregating WT leaf

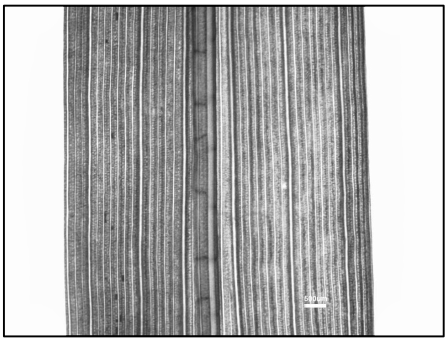

Representative surface image of line 4 leaf

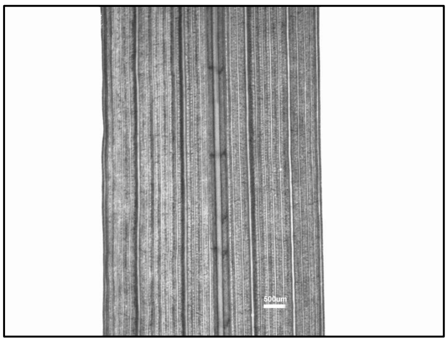

GRMZM2G114276 **LRR-RLK** **Gene ID: JL66**

Foliar expression

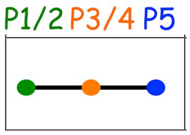

Husk expression

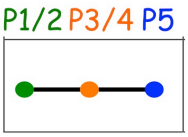

Construct: ZmUBI$_{pro}$:cDNA (ID# 959)

Thirty five independent T0 lines:
IR64-IRS959-001–035

Genomic PCR

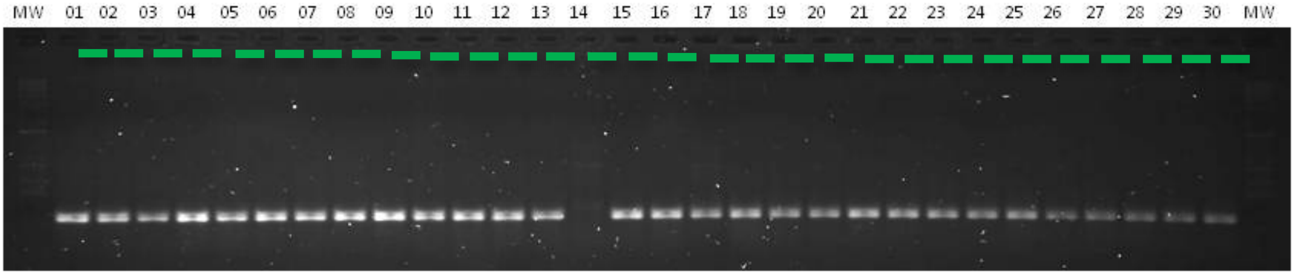

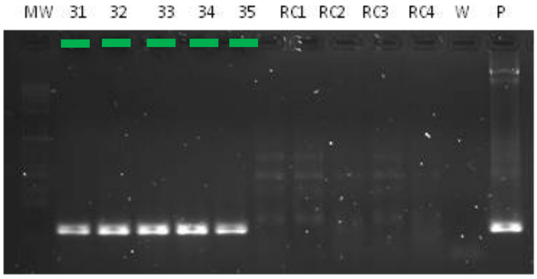

Number veins versus leaf width

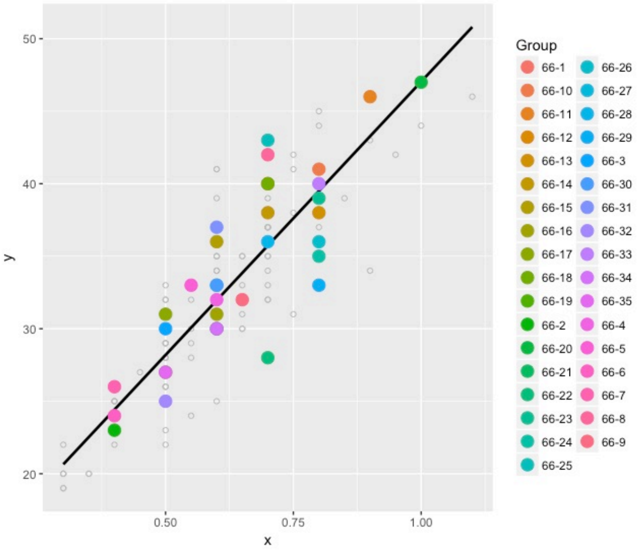

Representative surface image of segregating WT leaf

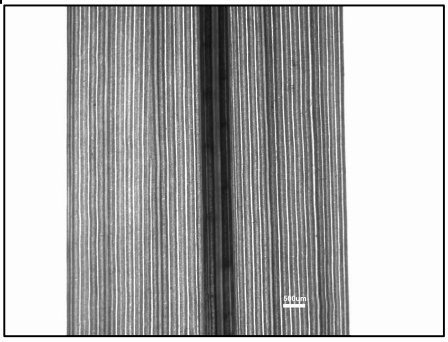

Representative surface image of line 9 leaf

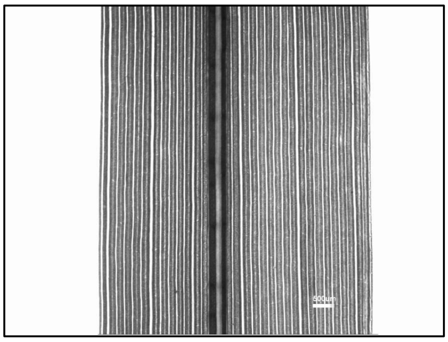

GRMZM2G059117 **LRR-RLK** **Gene ID: JL67**

Foliar expression

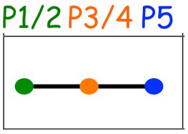

Husk expression

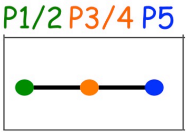

Construct: ZmUBI$_{pro}$:cDNA (ID# 1006)

Thirty four independent T0 lines:
IR64-IRS1006-001–031, 033-035

Genomic PCR

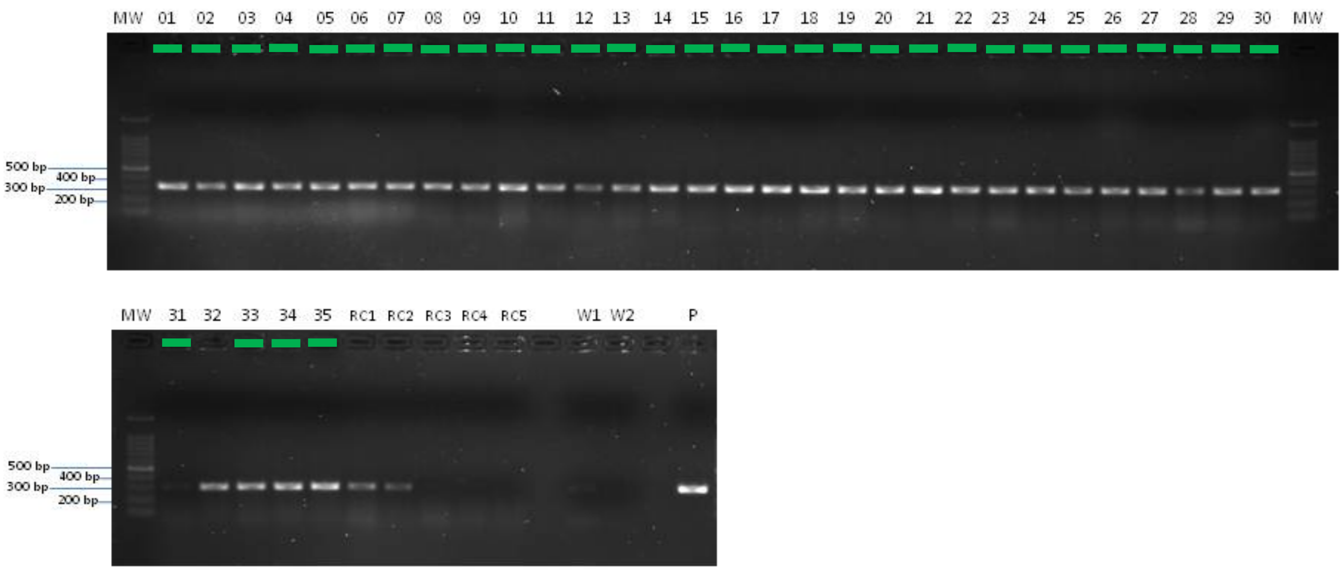

Number veins versus leaf width

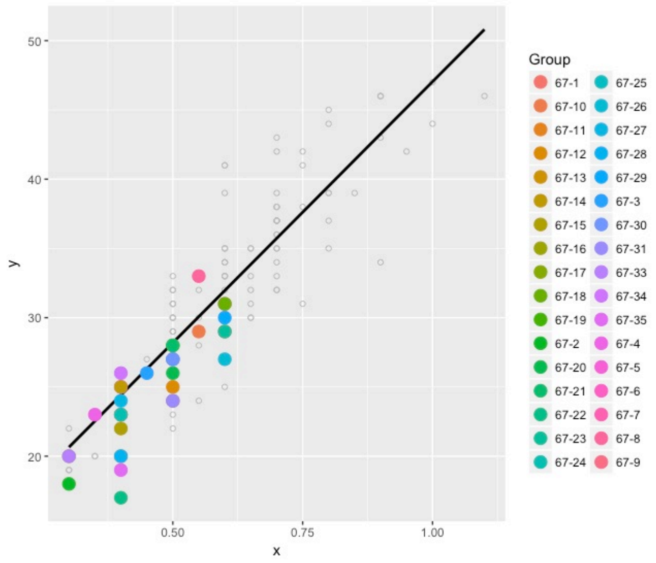

Representative surface image of segregating WT leaf

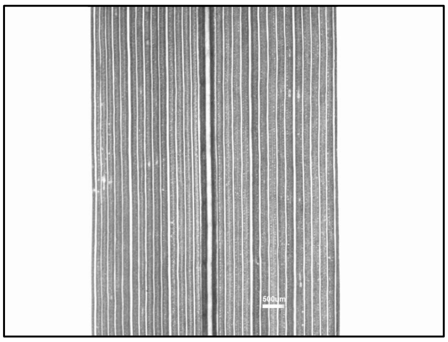

Representative surface image of line 3 leaf

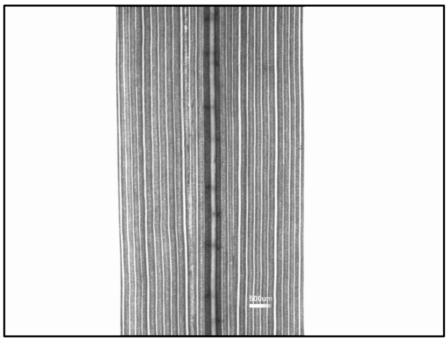

GRMZM2G089819 **Brassinosteroid LRR-RLK** **Gene ID: JL68**

Foliar expression

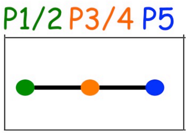

Husk expression

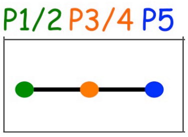

Construct: ZmUBI$_{pro}$:cDNA (ID# 953)

Thirty five independent T0 lines:
IR64-IRS953-001-035

Genomic PCR

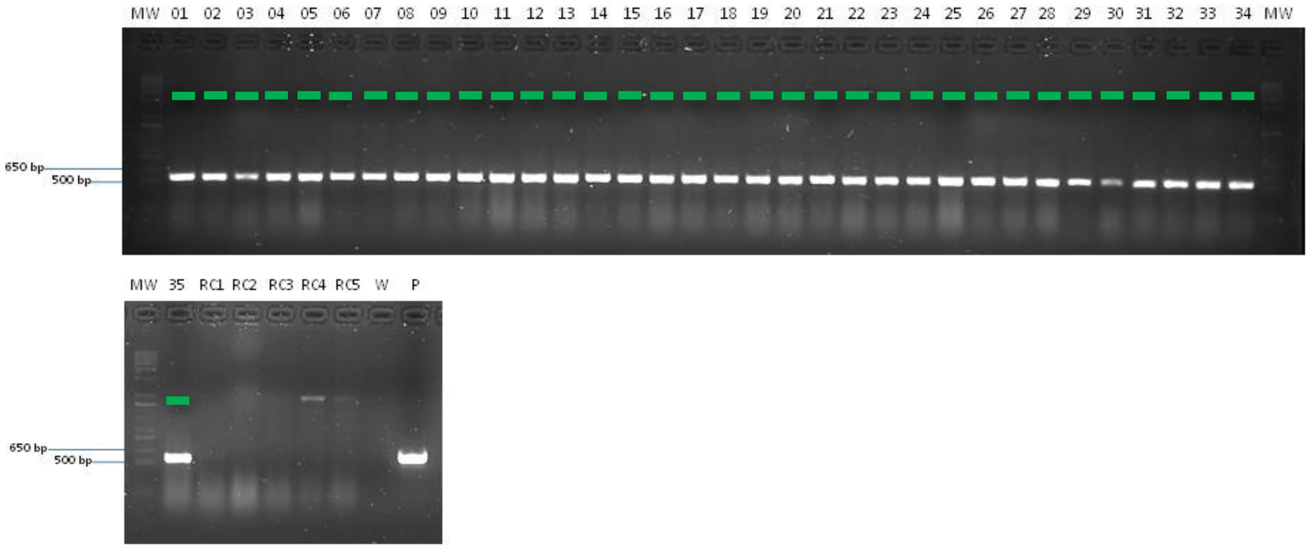

Number veins versus leaf width

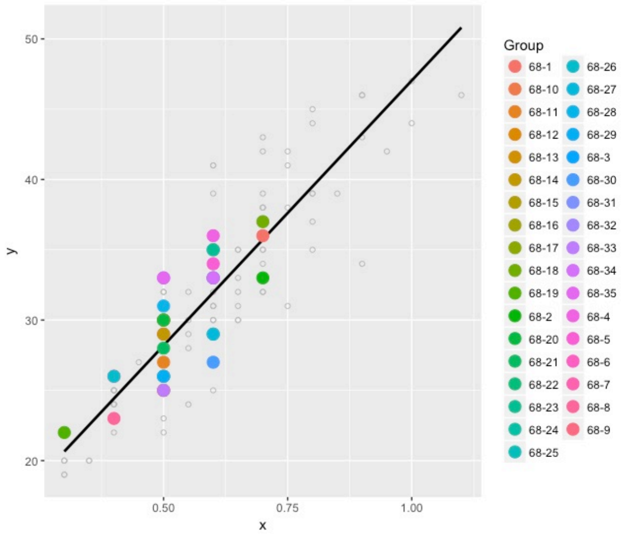

Representative surface image of segregating WT leaf

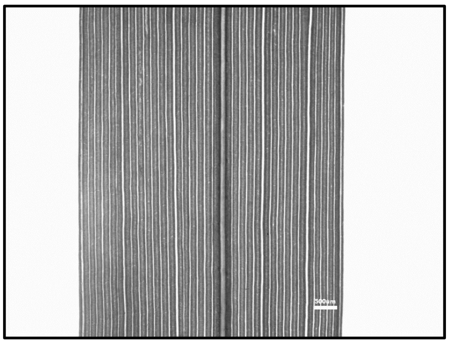

Representative surface image of line 14 leaf

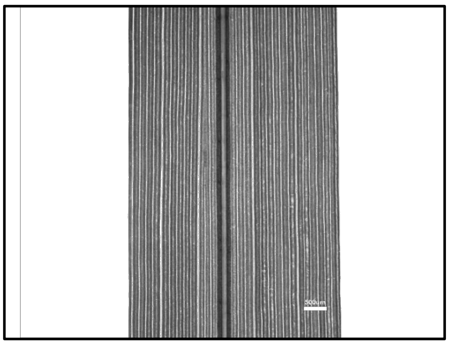

GRMZM2G344857 Phosphatidylinositol-4-phosphate 5-kinase Gene ID: JL69

Foliar expression

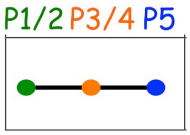

Husk expression

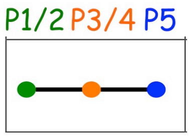

Construct: ZmUBI$_{pro}$:cDNA (ID# 1007)

Thirty five independent T0 lines:
IR64-IRS1007-001-035

Genomic PCR

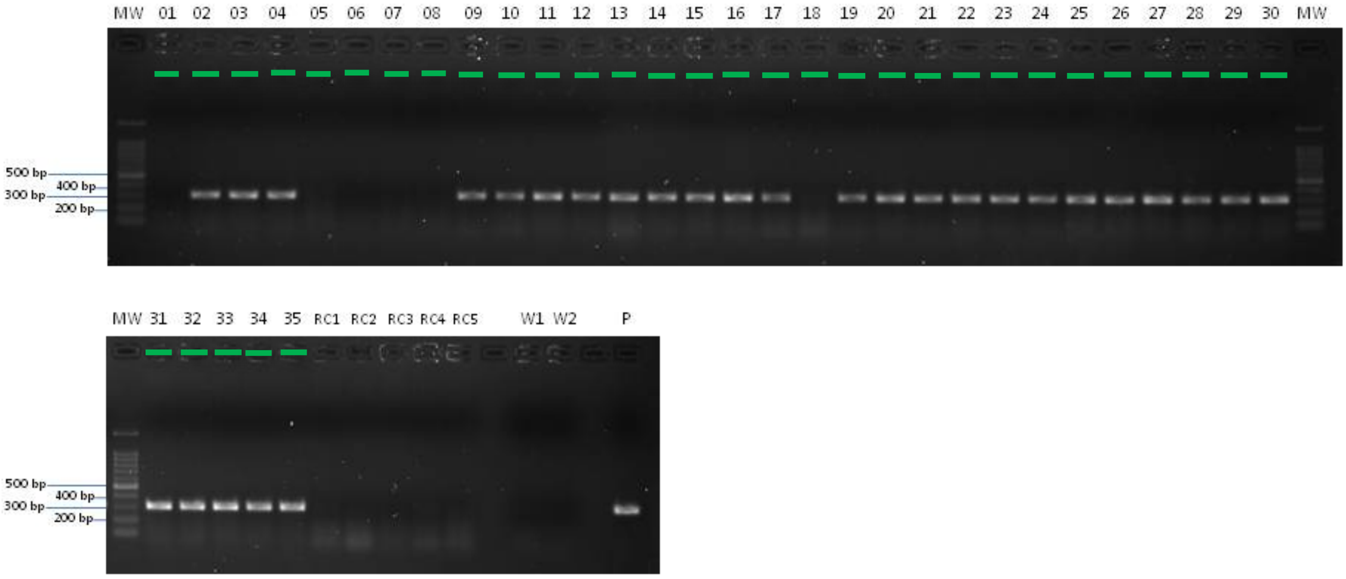

Number veins versus leaf width

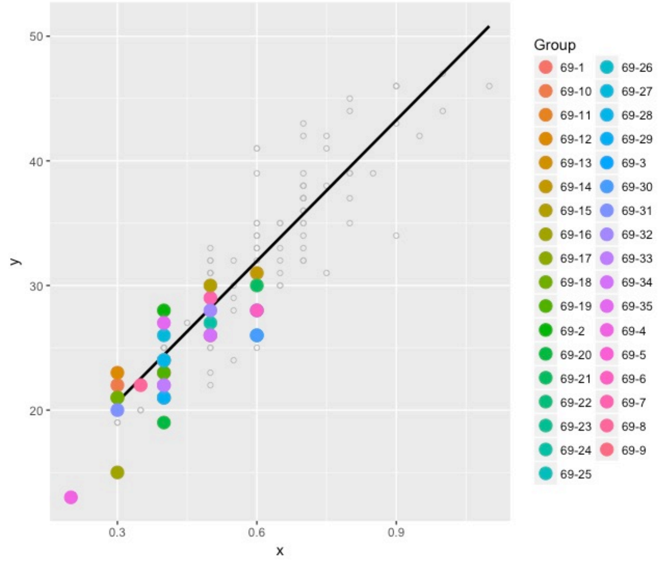

Representative surface image of segregating WT leaf

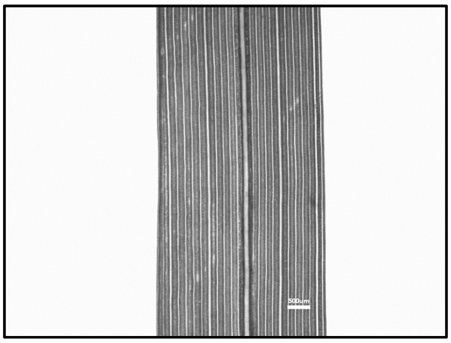

Representative surface image of line 27 leaf

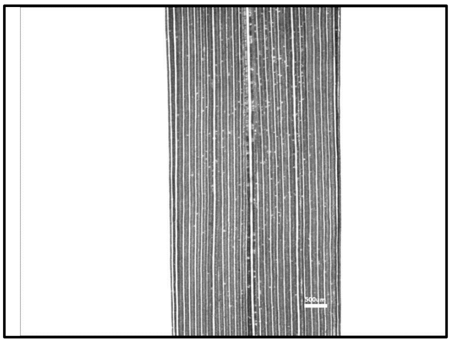

GRMZM2G087243 **Armadillo like** **Gene ID: JL70**

Foliar expression

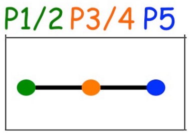

Husk expression

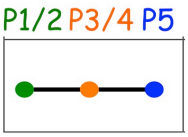

Construct: ZmUBI$_{pro}$:cDNA (ID# 929)

Thirty four independent T0 lines:
IR64-IRS929-001-026, 028-035

Genomic PCR

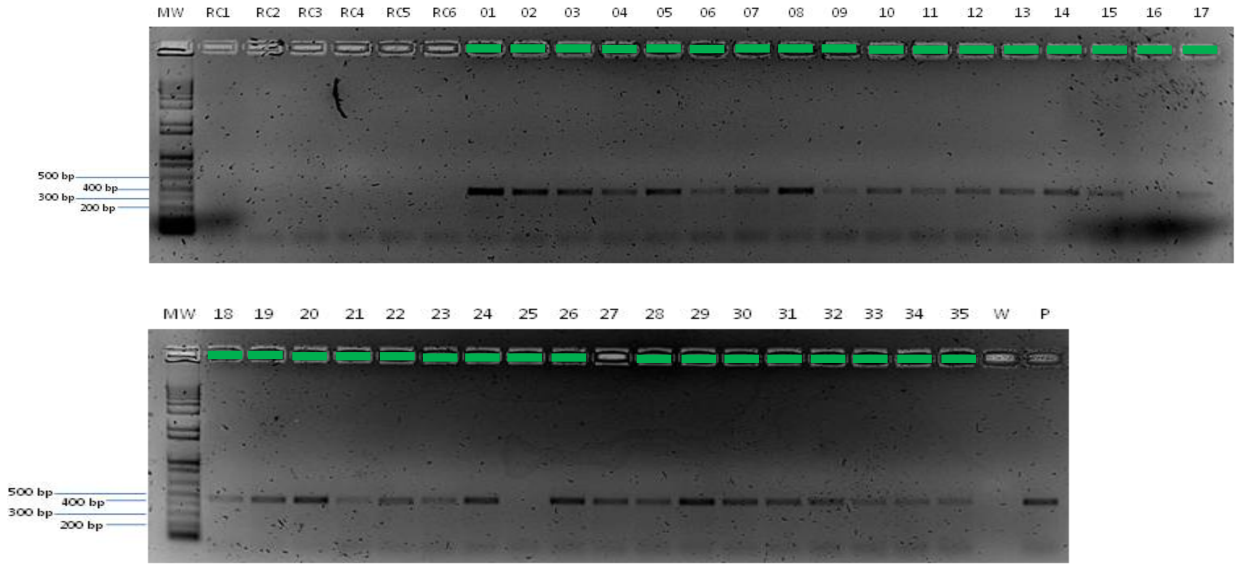

Number veins versus leaf width

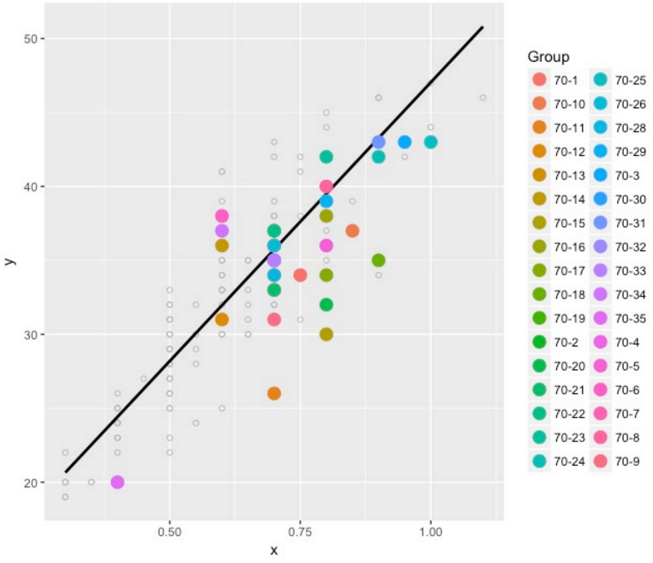

Representative surface image of segregating WT leaf

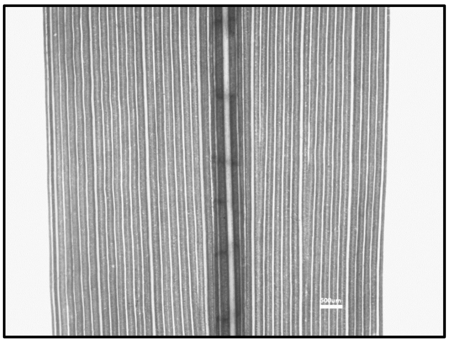

Representative surface image of line 2 leaf

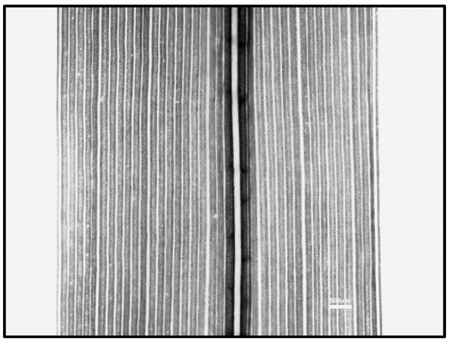

GRMZM2G133716 **Forkhead domain** **Gene ID: JL71**

Foliar expression

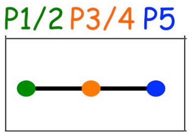

Husk expression

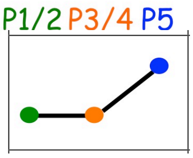

Construct: ZmUBI$_{pro}$:cDNA (ID# 986)

Twenty nine independent T0 lines:
IR64-IRS986-001-009,011,012,014,016,017,020-027,029,030,031-035

Genomic PCR

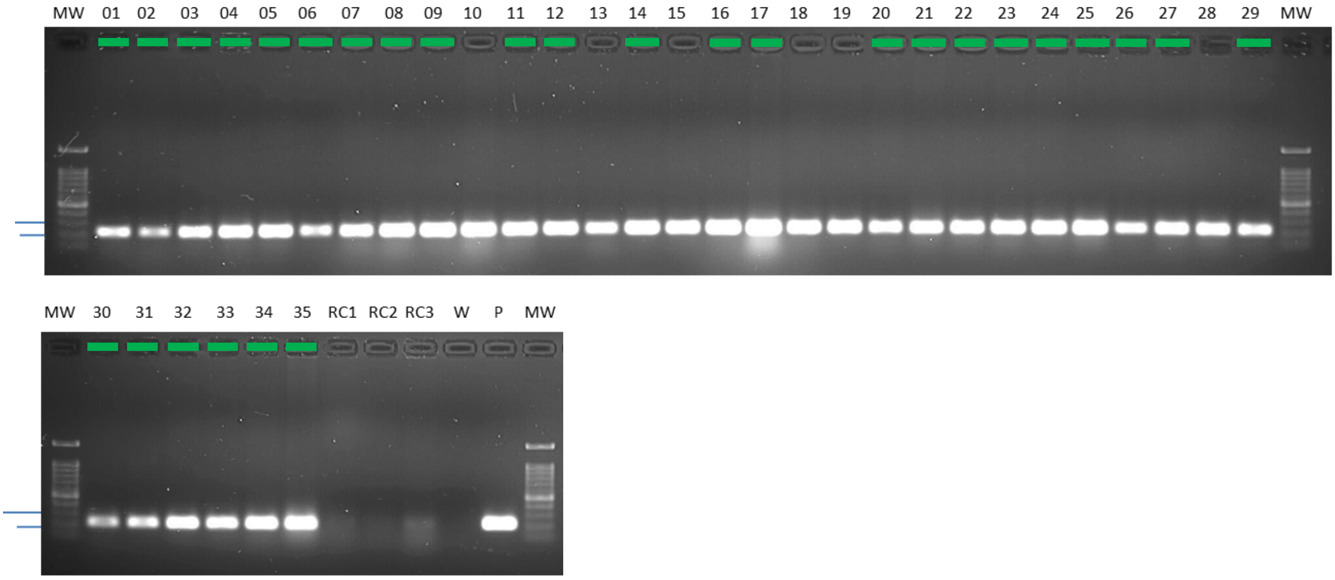

Number veins versus leaf width

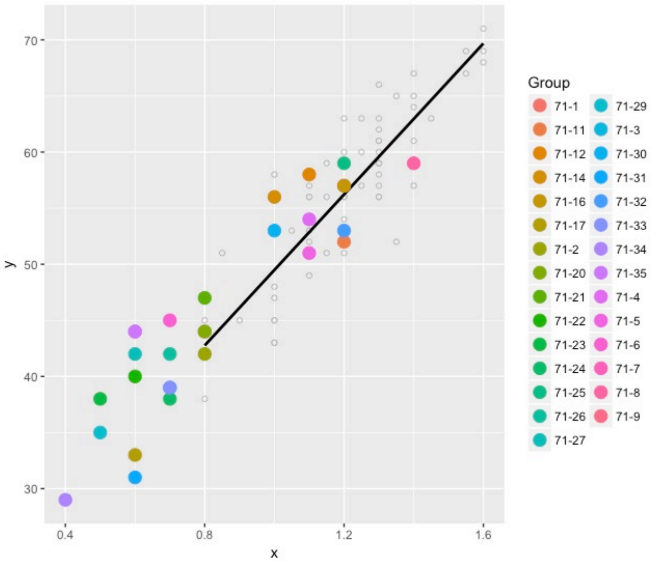

Representative surface image of segregating WT leaf

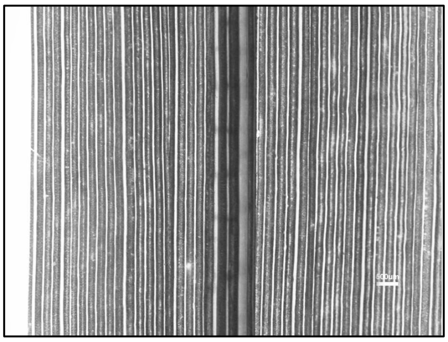

Representative surface image of line 21 leaf

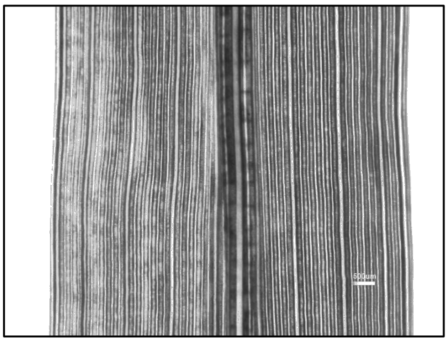

GRMZM2G469304 **Ternary complex factor** **Gene ID: JL72**

Foliar expression

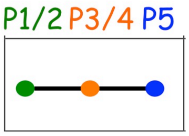

Husk expression

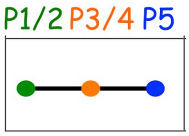

Construct: ZmUBI$_{pro}$:cDNA (ID# 1008)

Thirty five independent T0 lines:
IR64-IRS1008-001-035

Genomic PCR

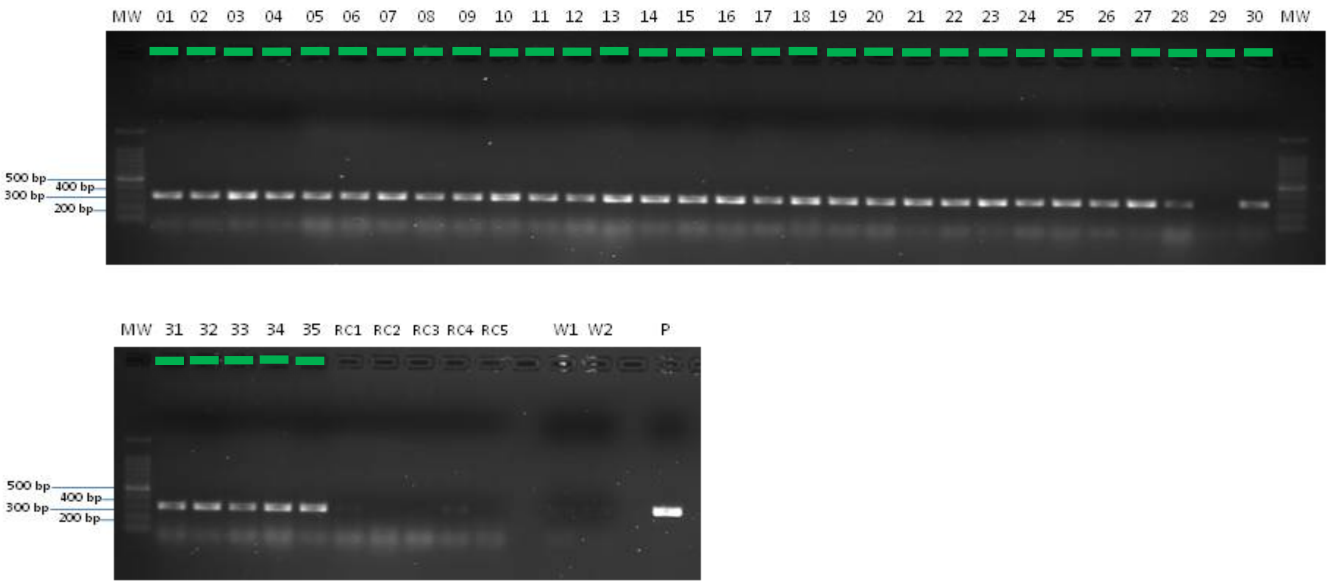

Number veins versus leaf width

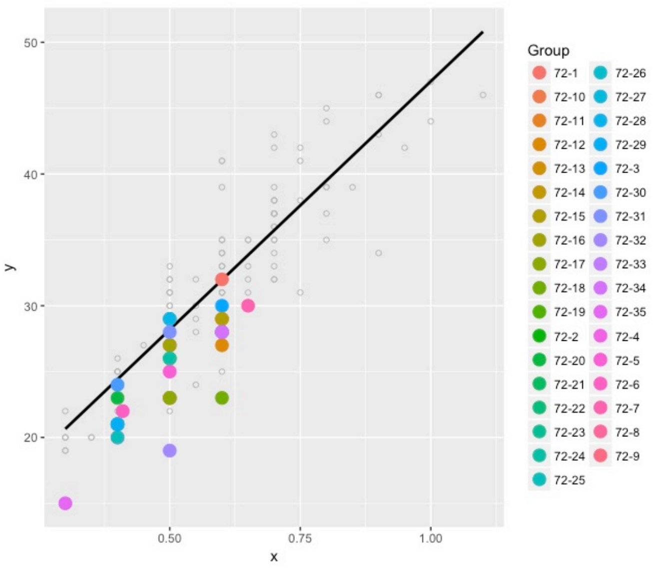

Representative surface image of segregating WT leaf

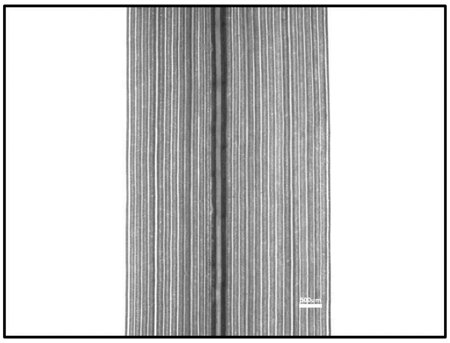

Representative surface image of line 5 leaf

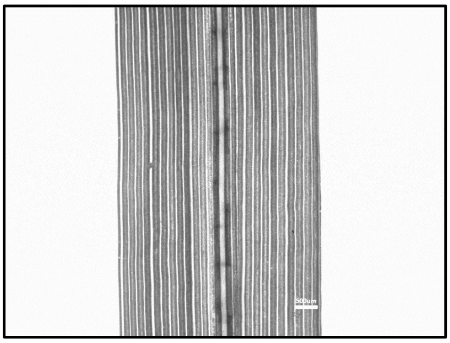

GRMZM5G850129 **Growth regulatory factor** **Gene ID: JL75**

Foliar expression

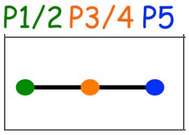

Husk expression

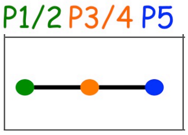

Construct: ZmUBI$_{pro}$:cDNA (ID# 948)

Five independent T0 lines:
IR64-IRS948-009,022,024,026,028

Genomic PCR

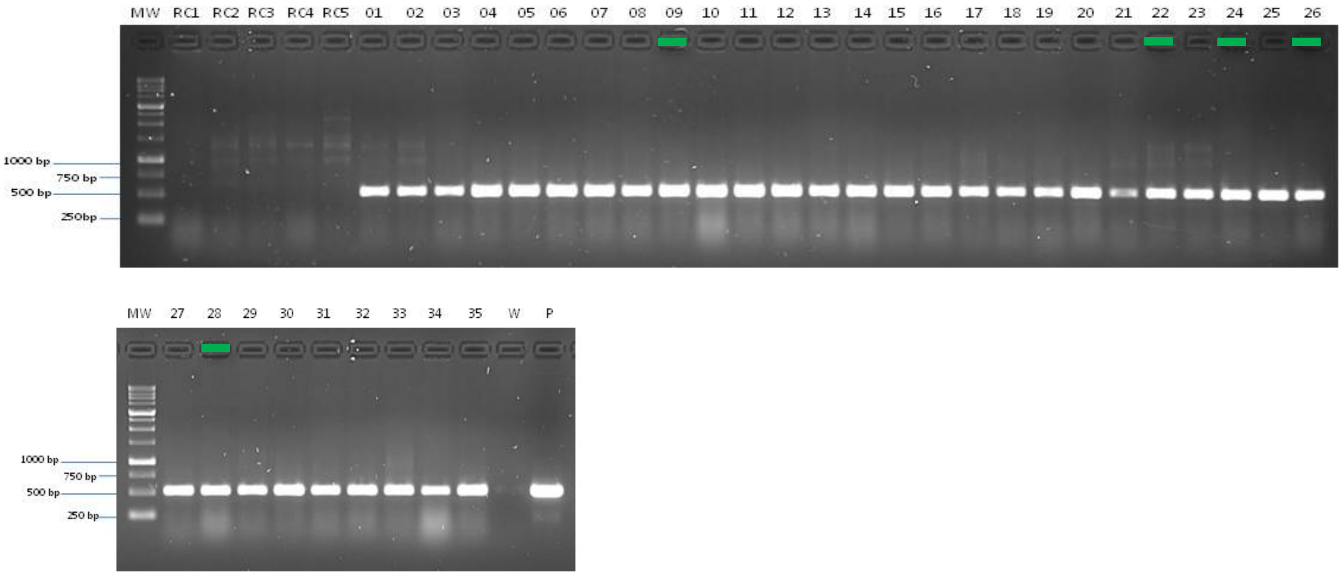

Number veins versus leaf width

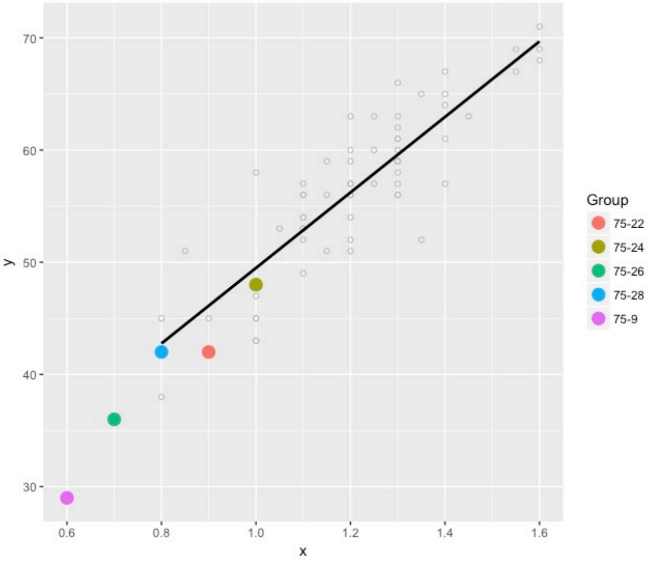

Representative surface image of segregating WT leaf

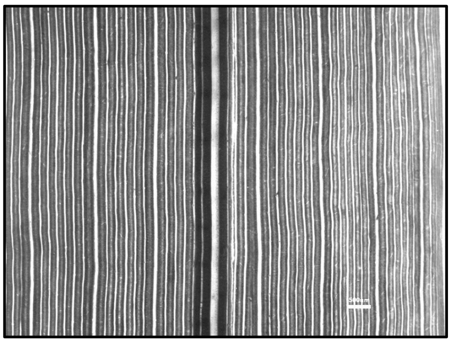

Representative surface image of line 22 leaf

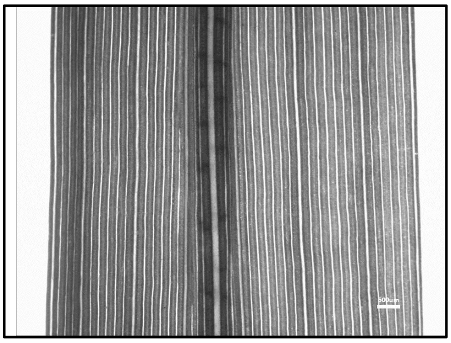

GRMZM2G061734 **SBP domain TF** **Gene ID: JL76**

Foliar expression

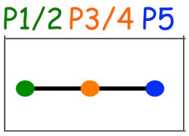

Husk expression

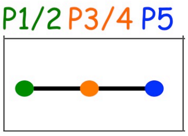

Construct: ZmUBI$_{pro}$:cDNA (ID# 960)

Thirteen independent T0 lines:
IR64-IRS960-002,006,007,011,012,017,018,020,022,025,031,033,034

Genomic PCR

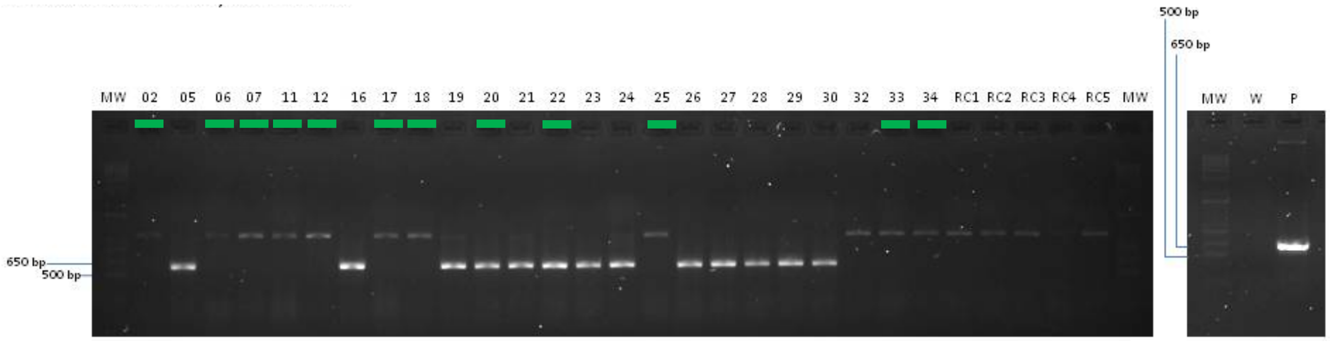

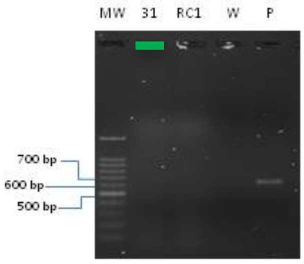

Number veins versus leaf width

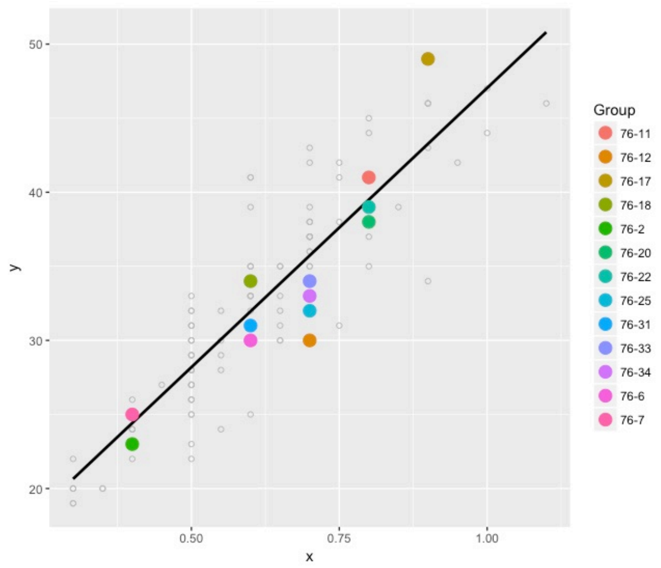

Representative surface image of segregating WT leaf

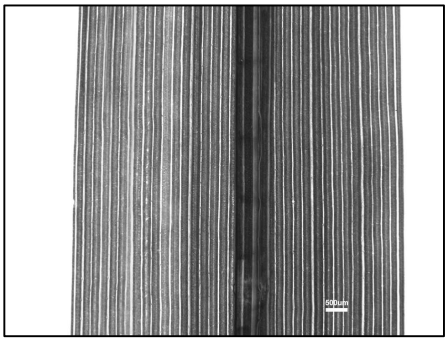

Representative surface image of line 34 leaf

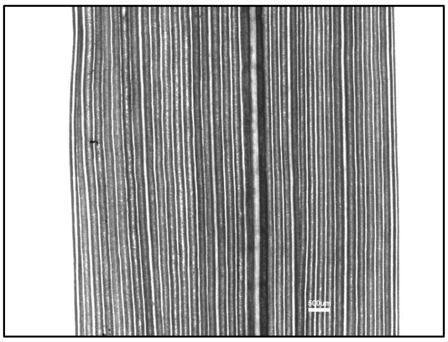

GRMZM2G318592 **ZnF C2H2** **Gene ID: JL77**

Foliar expression

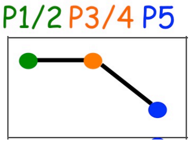

Husk expression

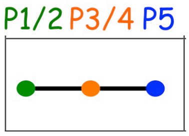

Construct: ZmUBI$_{pro}$:cDNA (ID# 1009)

Thirty five independent T0 lines:
IR64-IRS1009-001-035

Genomic PCR

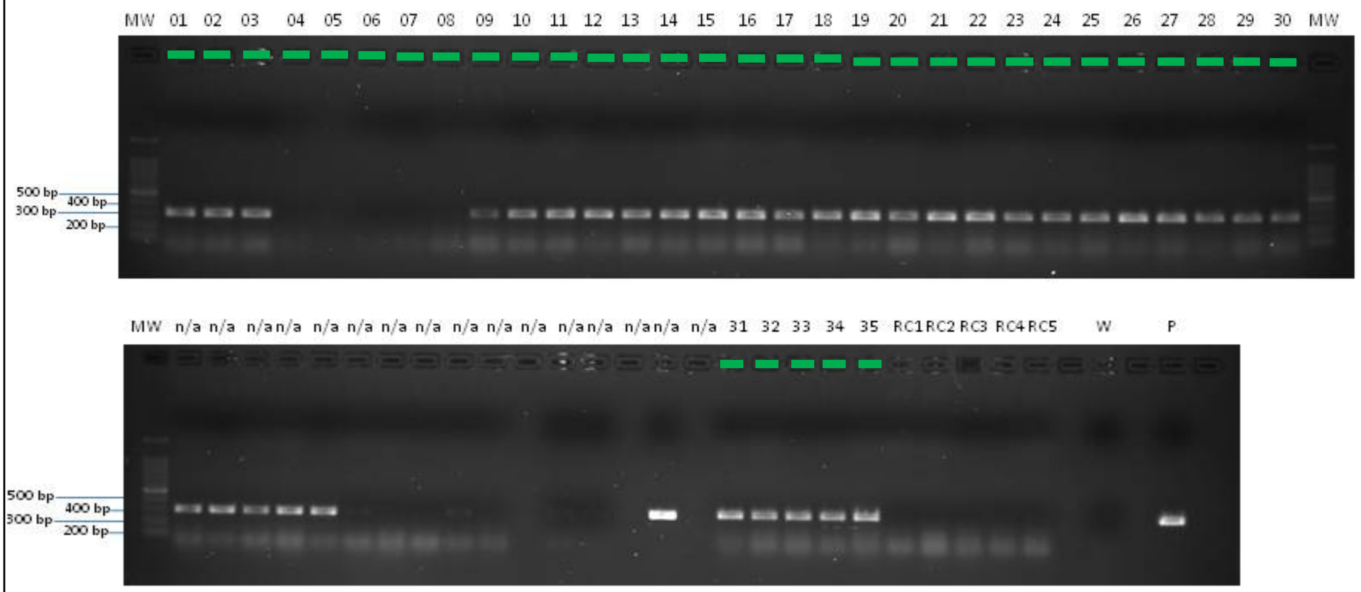

Number veins versus leaf width

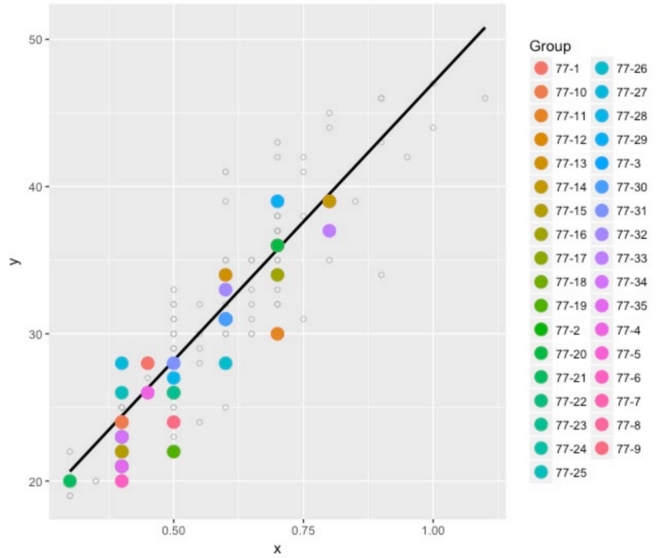

Representative surface image of segregating WT leaf

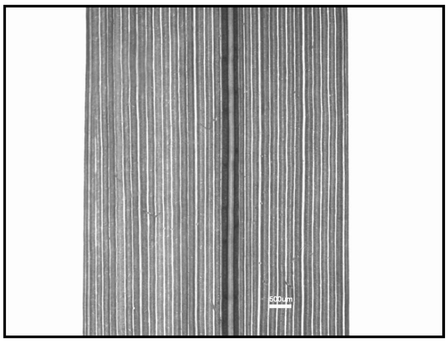

Representative surface image of line 3 leaf

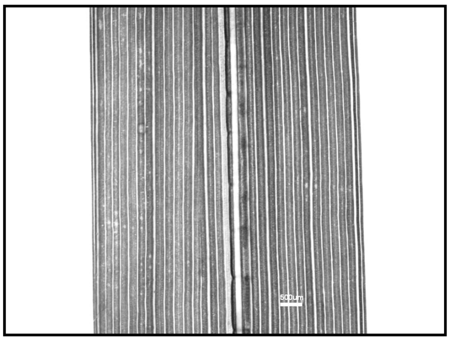

GRMZM2G095899 **bHLH** **Gene ID: JL78**

Foliar expression

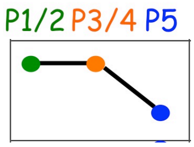

Husk expression

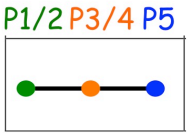

Construct: ZmUBI$_{pro}$:cDNA (ID# 942)

Ten independent T0 lines:
IR64-IRS942-001,003,004,007,009,010,012,013,018,019

Genomic PCR

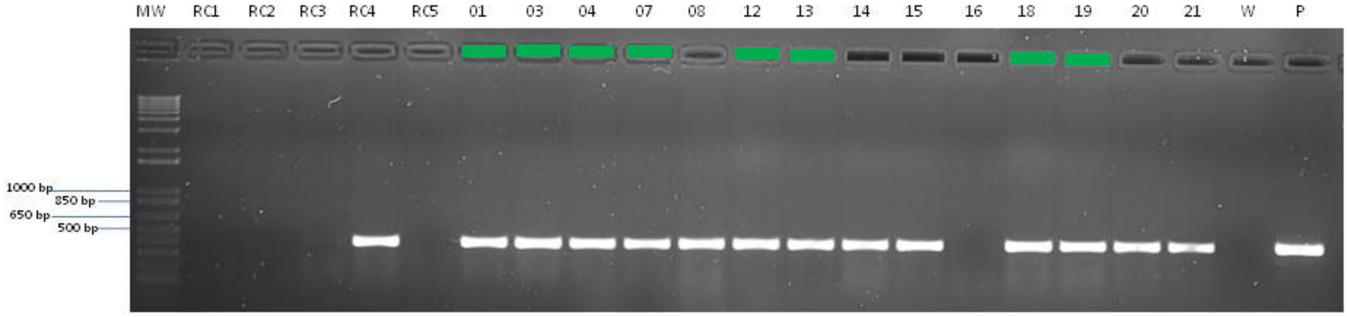

Number veins versus leaf width

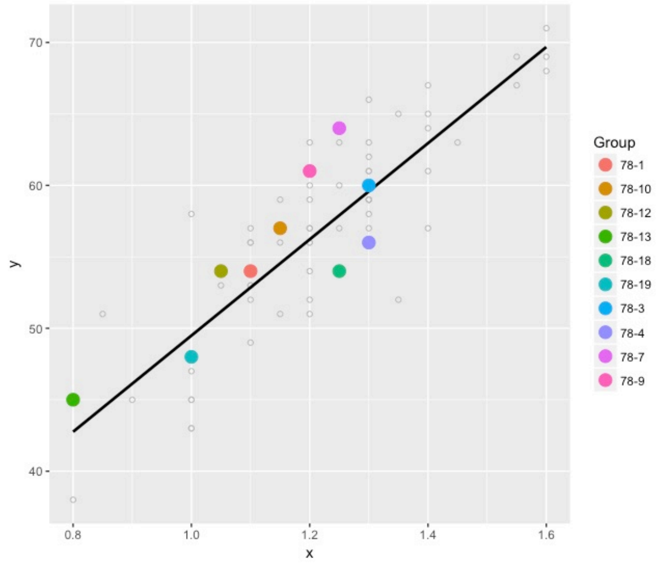

Representative surface image of segregating WT leaf

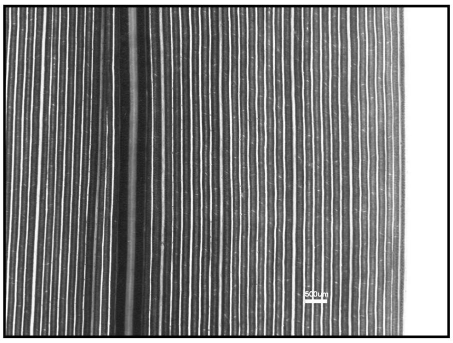

Representative surface image of line 10 leaf

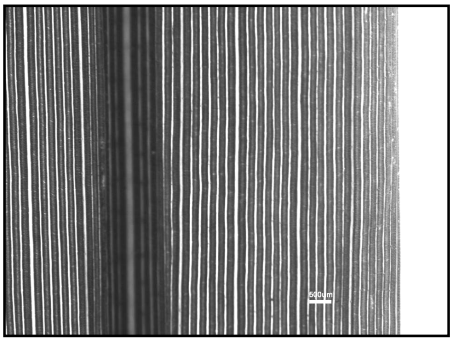

GRMZM2G015666 **bHLH** **Gene ID: JL79**

Foliar expression

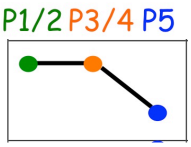

Husk expression

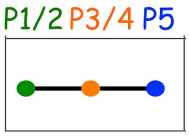

Construct: ZmUBI$_{pro}$:cDNA (ID# 946)

Thirty five independent T0 lines:
IR64-IRS946-001-035

Genomic PCR

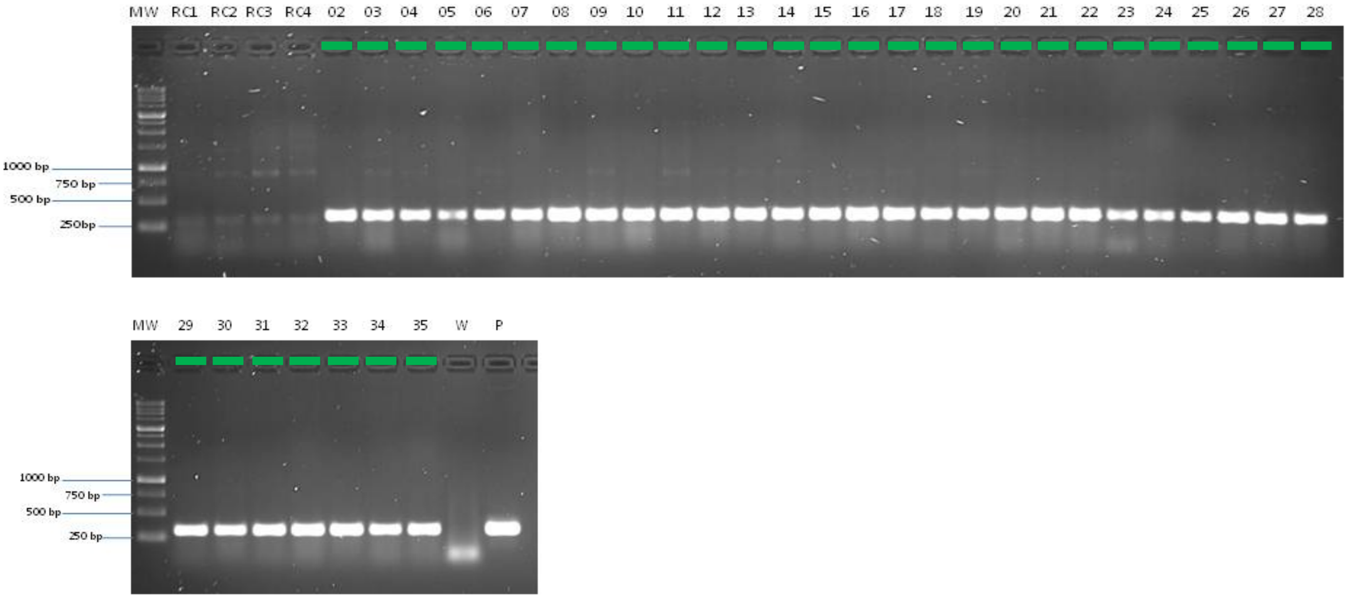

Number veins versus leaf width

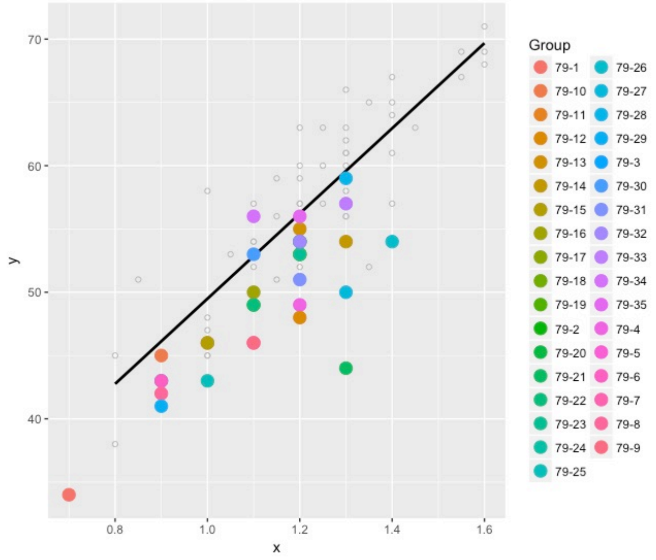

Representative surface image of segregating WT leaf

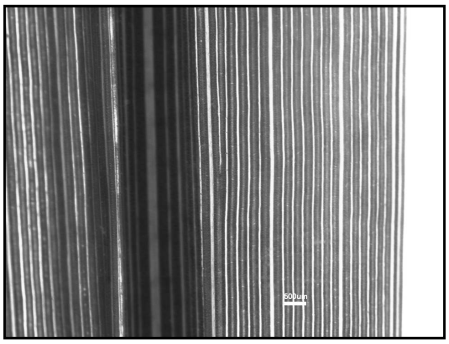

Representative surface image of line 2 leaf

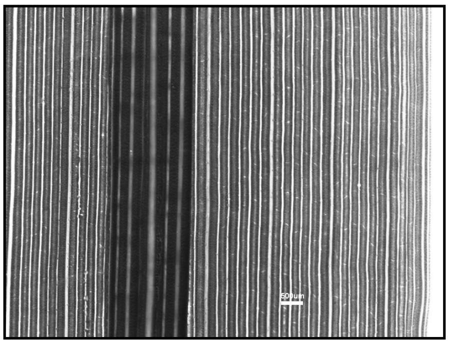

GRMZM2G126018 **SBP domain TF** **Gene ID: JL80**

Foliar expression

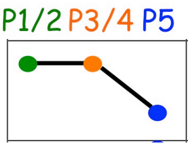

Husk expression

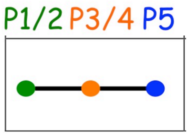

Construct: $ZmUBI_{pro}$:cDNA (ID# 954)

Twenty seven independent T0 lines:
IR64-IRS954-001-004,007,010,012,014-018,020-025,027,028,031,032,034-039

Genomic PCR

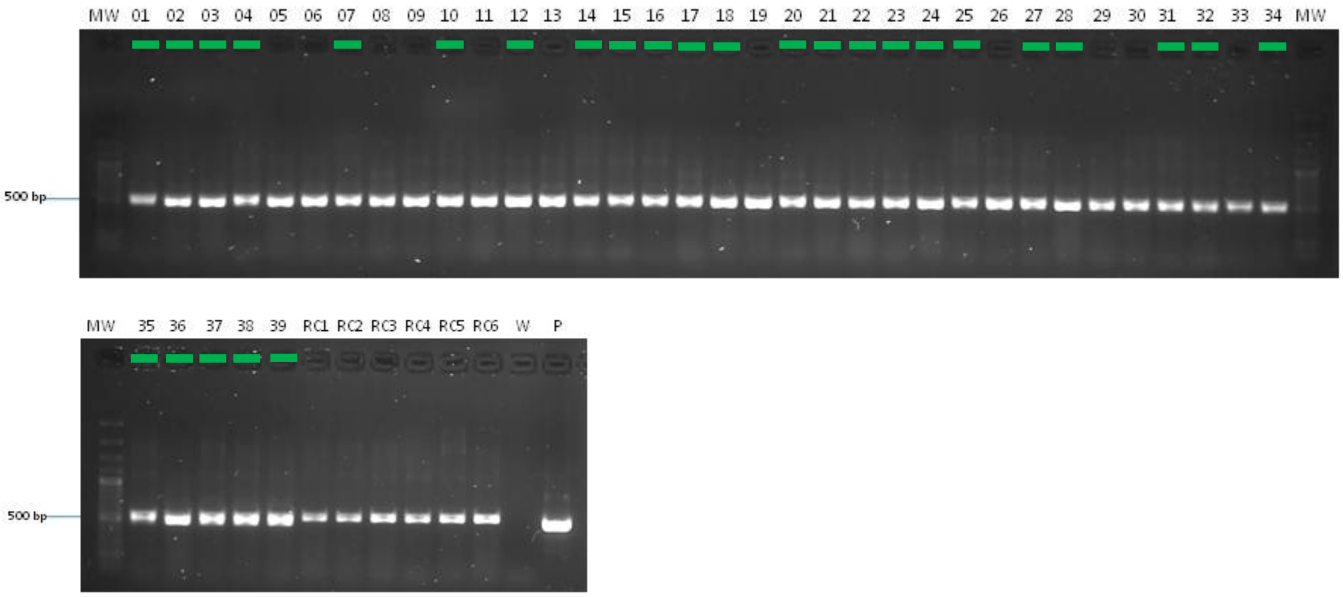

Number veins versus leaf width

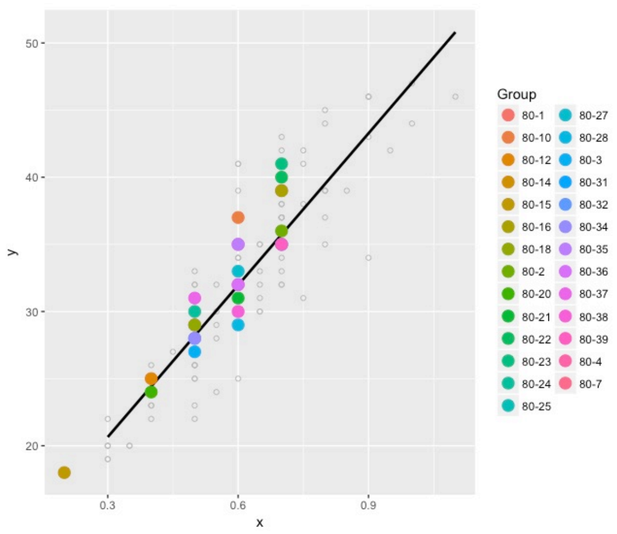

Representative surface image of segregating WT leaf

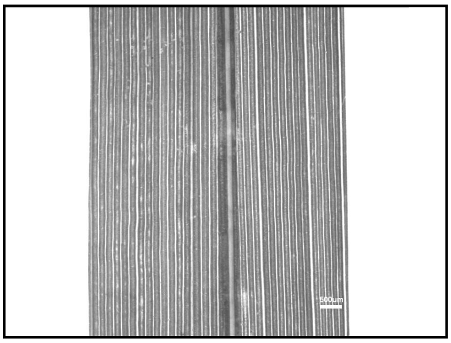

Representative surface image of line 21 leaf

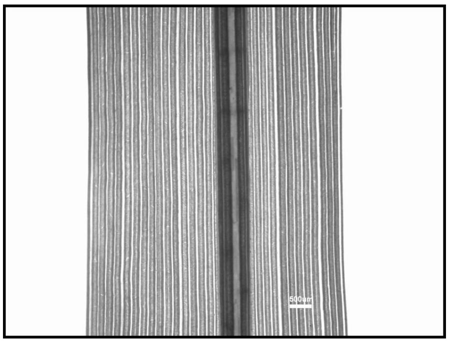

GRMZM2G312419 **R2R3 Myb** **Gene ID: JL81**

Foliar expression

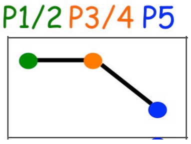

Husk expression

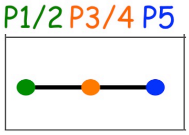

Construct: ZmUBI$_{pro}$:cDNA (ID# 961)

Seventeen independent T0 lines:
IR64-IRS961-001-017

Genomic PCR

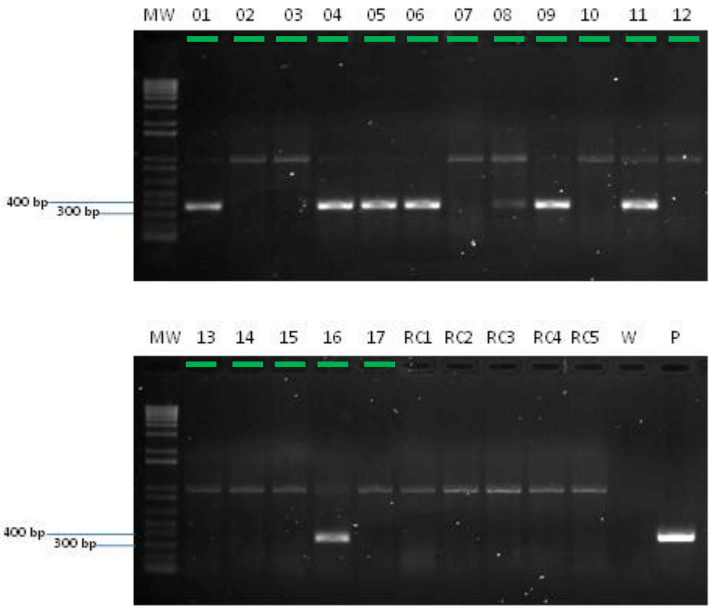

Number veins versus leaf width

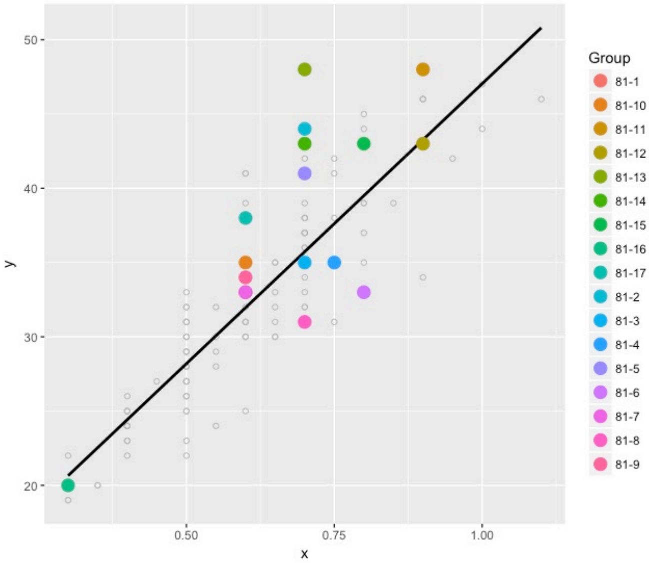

Representative surface image of segregating WT leaf

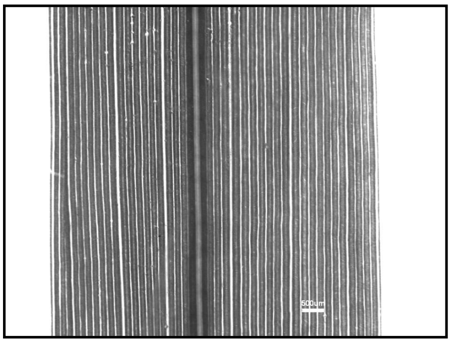

Representative surface image of line 5 leaf

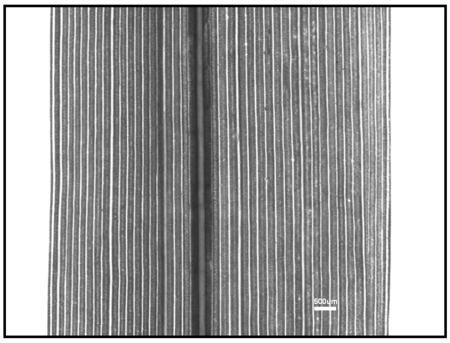

GRMZM2G478876 **Serine threonine kinase** **Gene ID: JL82**

Foliar expression

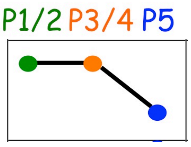

Husk expression

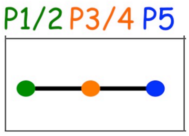

Construct: ZmUBI$_{pro}$:cDNA (ID# 940)

Thirty one independent T0 lines:
IR64-IRS940-003-016,018,019,021-035

Genomic PCR

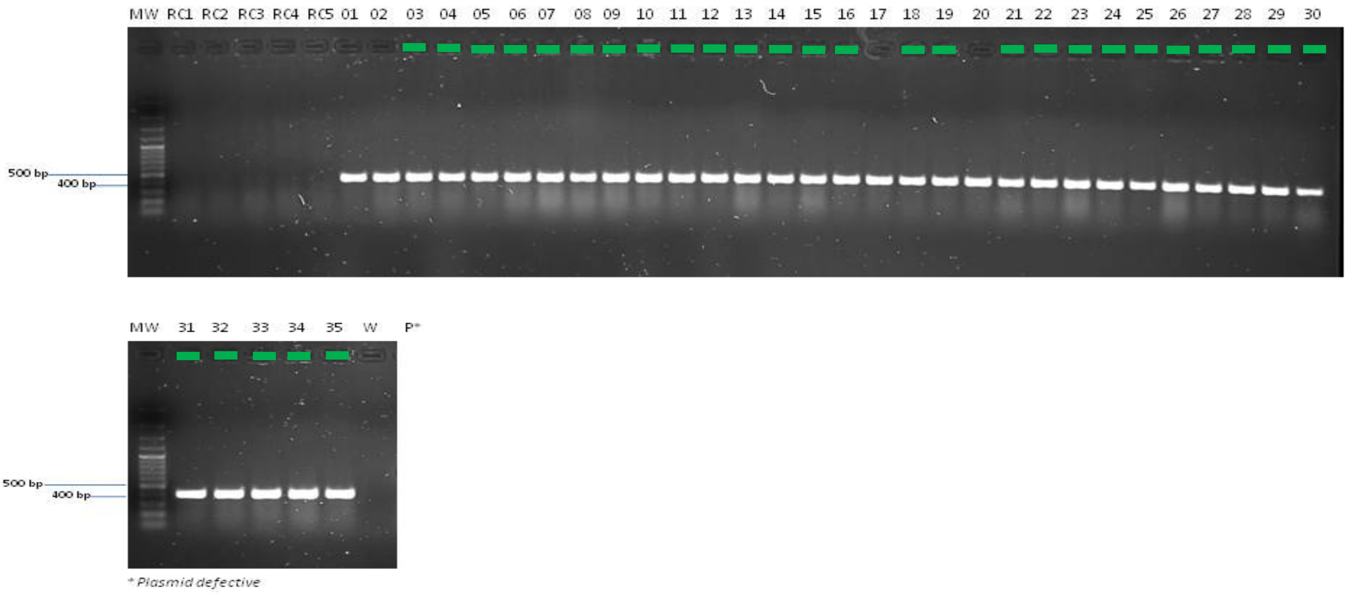

Number veins versus leaf width

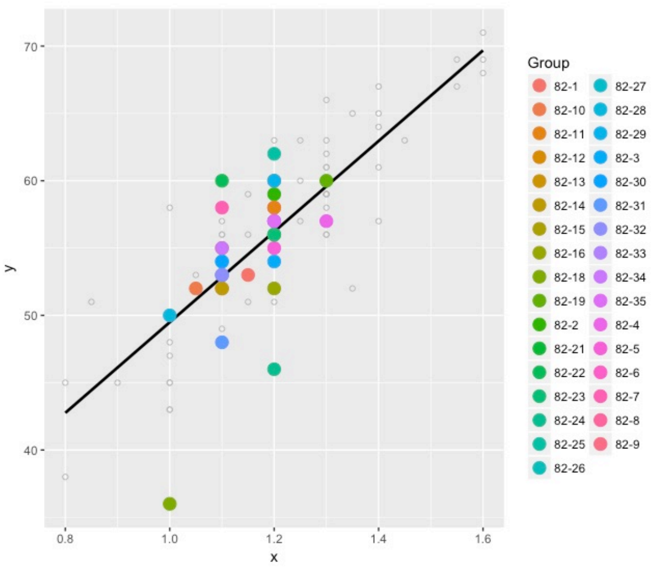

Representative surface image of segregating WT leaf

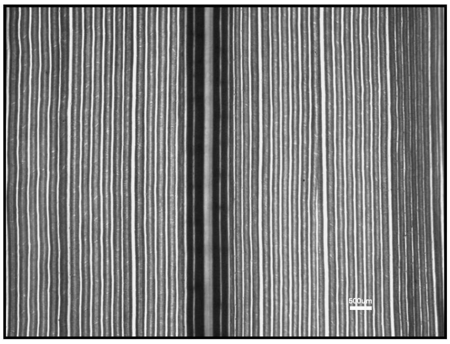

Representative surface image of line 21 leaf

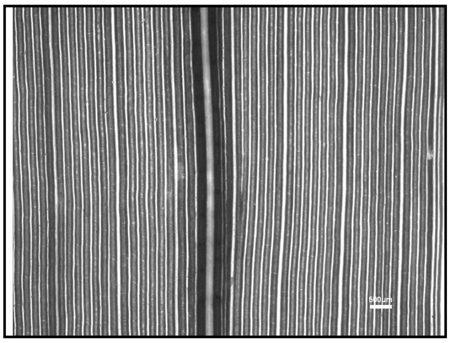

Information S3. Individual datasheets for T1 rice lines summarized in Dataset S3. Each page shows the gene expression profile in maize foliar and husk leaf primordia as reported in [19] (green circles refer to relative expression level in samples of the shoot apical meristem plus plastochron 1/2 leaf primordia, orange circles to levels in P3/4 leaf primordia and blue circles to levels in P5 leaf primordia); construct composition and ID; number and ID of independent transgenic rice lines. Where available, cropped agarose gel images show PCR products of representative genomic PCR reactions (detecting presence of transgene) and RT-PCR reactions (detecting transgene transcripts). qRT-PCR data are shown for one construct. Polynomial (Kitaake lines) or linear (IR64 lines) regression plots illustrate number of veins versus leaf width in each line. Representative leaf samples (either hand cut transverse leaf sections viewed by fluorescence microscopy or views of the adaxial leaf surface taken using a hand-held microscope) are shown in each case. Fluorescence images are visualized through autofluorescence of chlorophyll (red) and lignin (blue).

GRMZM2G028046 **MYB-interacting protein (ZmMIL1)** **Gene ID: JL2**

Foliar expression

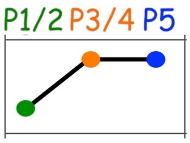

Husk expression

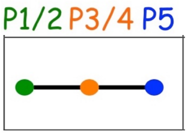

Construct: ZmUBI$_{pro}$:cDNA (ID# 76CC)

Two independent T1 lines:
ZmMIL1-1 (76CCS1 – 8 individuals)
ZmMIL1-2 (76CCS2 – 7 individuals)

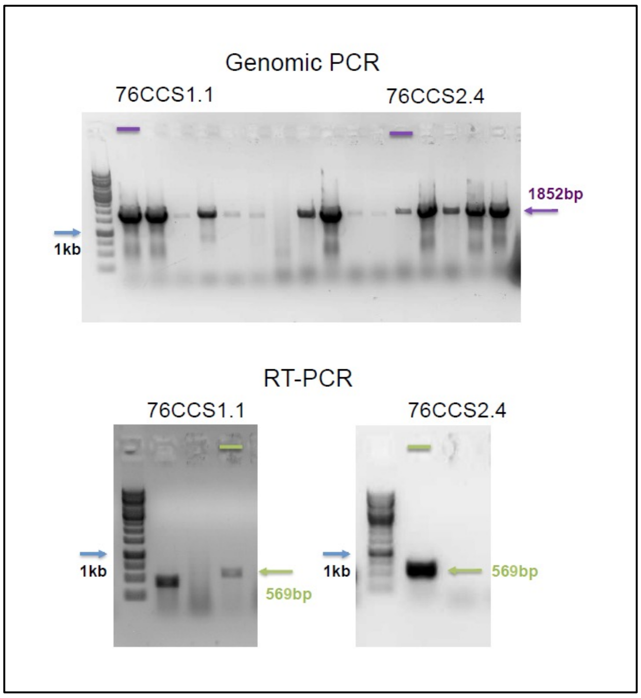

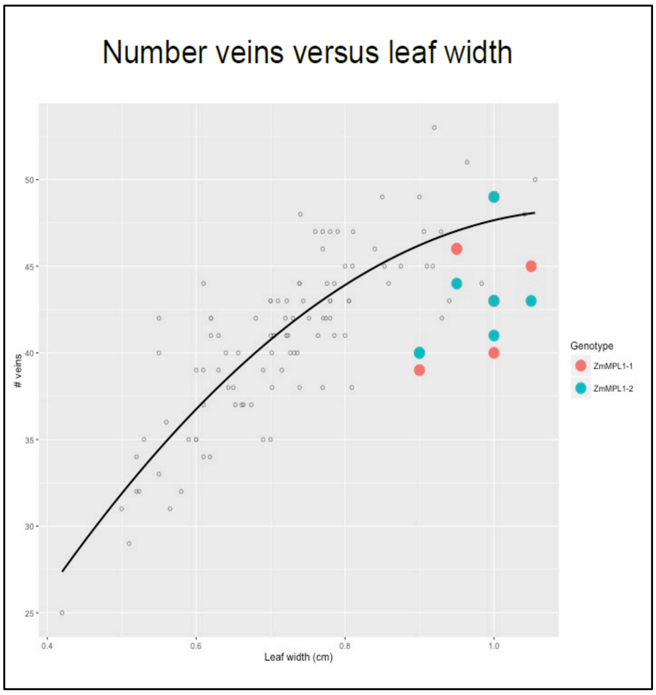

Representative leaf cross sections:

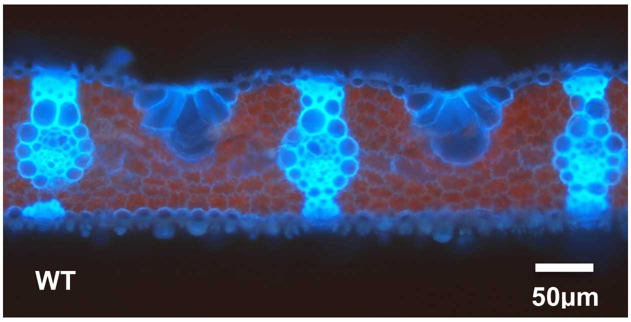

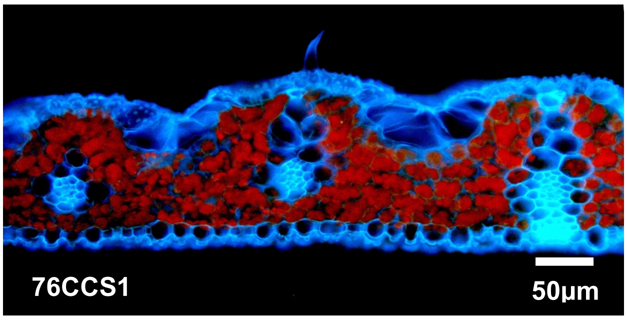

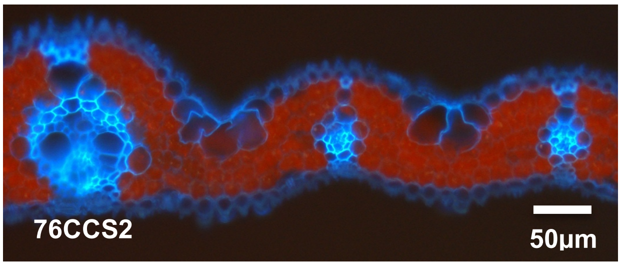

GRMZM2G136494 **MYB-interacting protein (ZmMIL2)** **Gene ID: JL3**

Foliar expression

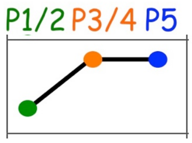

Husk expression

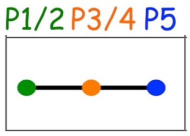

Construct: ZmUBI$_{pro}$:cDNA (ID# 84CC)

Three independent T1 lines:
ZmMIL2-1 (84CCSR1 – 7 individuals)
ZmMIL2-2 (84CCS201 – 6 individuals)
ZmMIL2-3 (84CCS204 – 2 individuals)

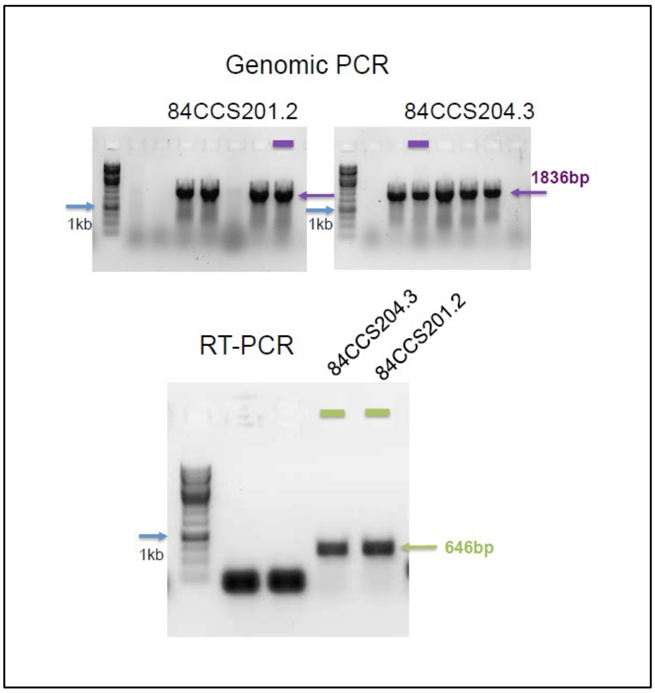

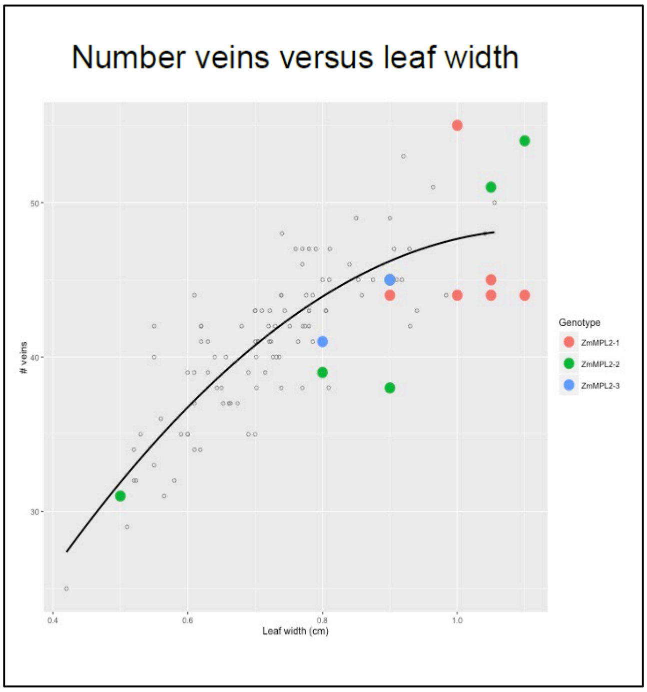

Representative leaf cross sections:

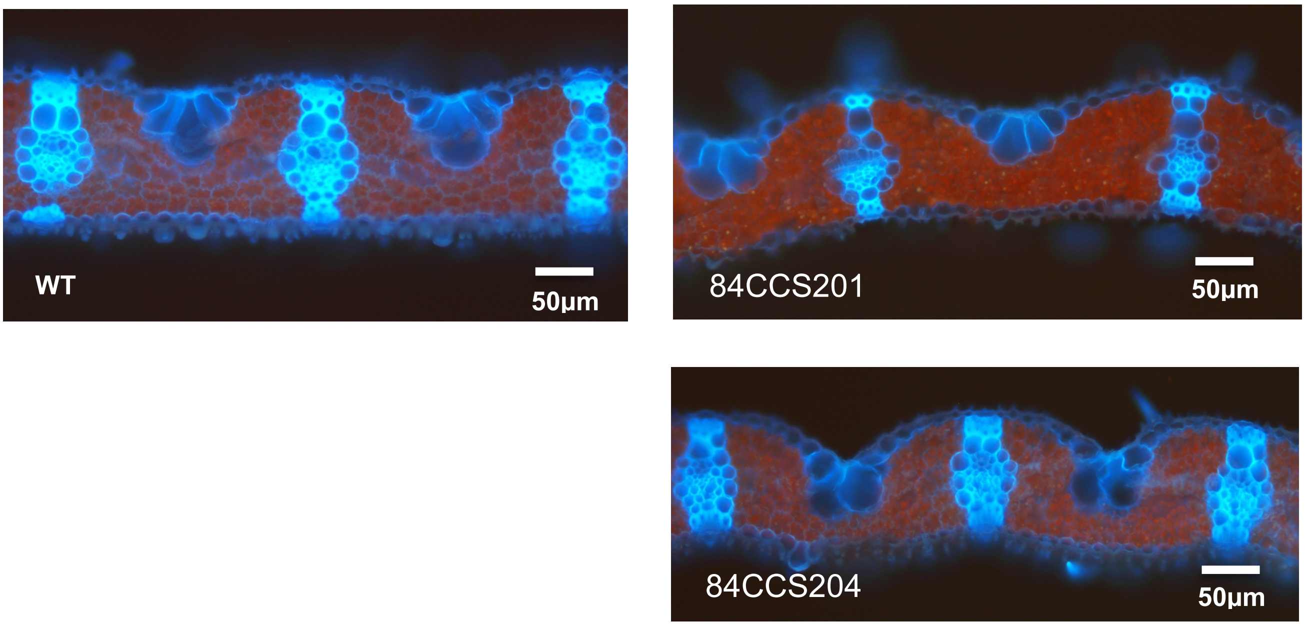

GRMZM2G045883 **bHLH (ZmSPL1)** **Gene ID: JL4**

Foliar expression

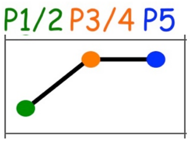

Husk expression

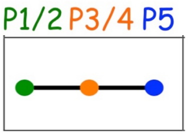

Construct: ZmUBI$_{pro}$:cDNA (ID# 79CC)

Two independent T1 lines:
ZmSPL1-1 (79CCSR16 – 1 individual)
ZmSPL1-2 (79CCSR21 – 3 individuals)

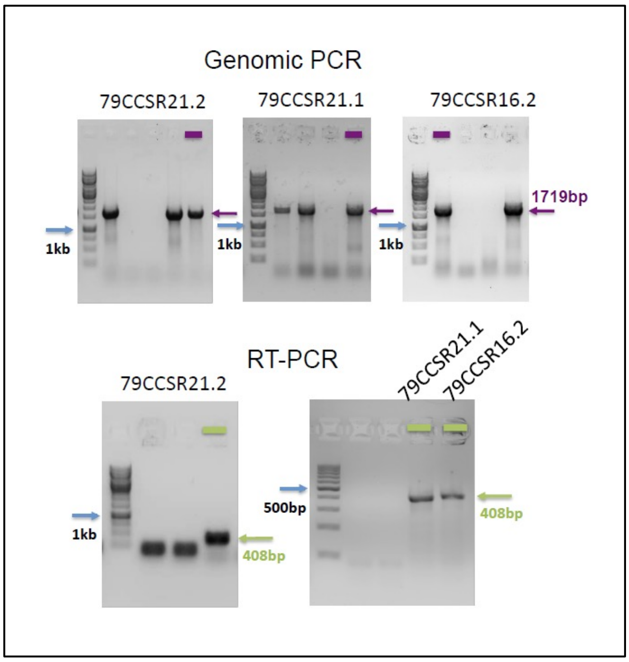

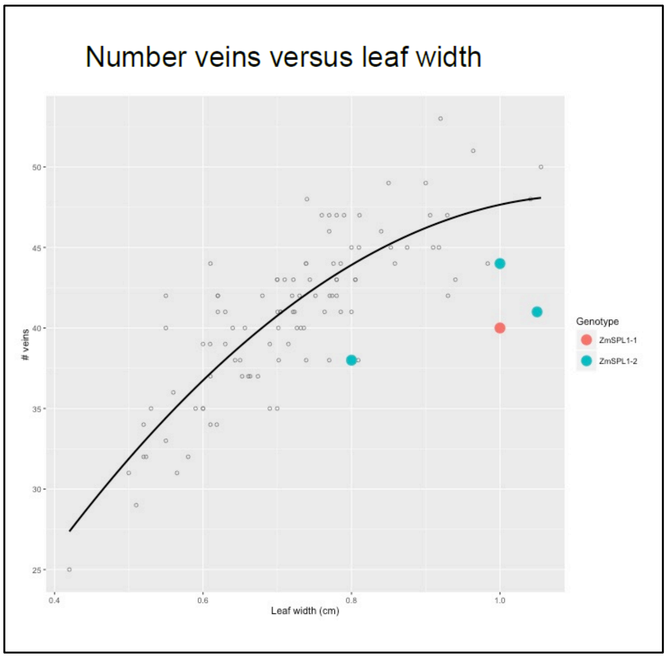

Representative leaf cross sections:

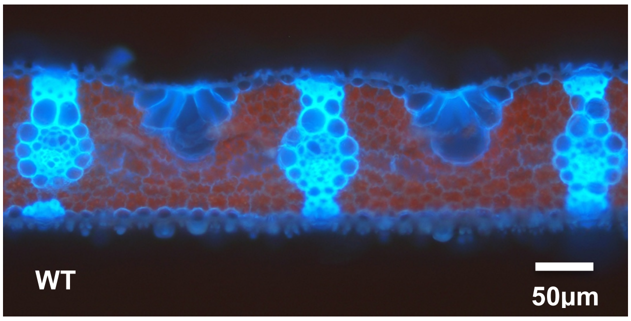

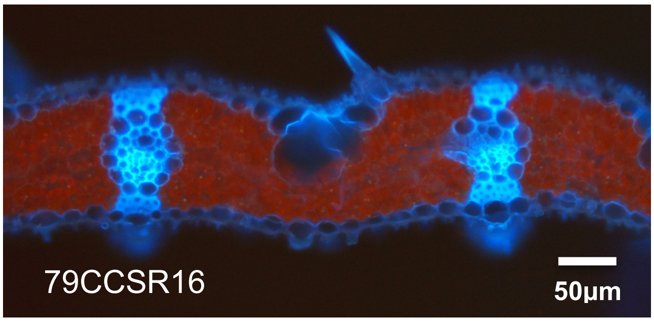

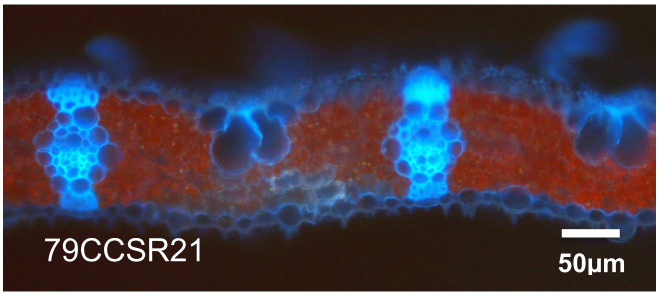

GRMZM2G131516 **GRAS (ZmSCR1)** **Gene ID: JL8**

Foliar expression

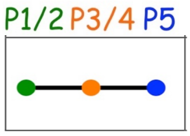

Husk expression

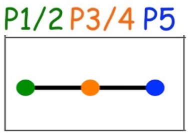

Construct: ZmUBI$_{pro}$:cDNA
(rice codon optimised) (ID# MS154)

Three independent T1 lines:
ZmSCR1-1 (1B – 3 individuals)
ZmSCR1-2 (5A – 3 individuals)
ZmSCR1-3 (7C – 3 individuals)

Genomic PCR

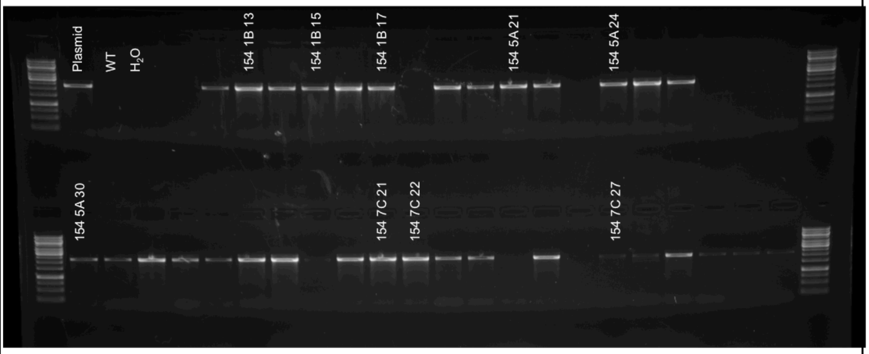

RT PCR

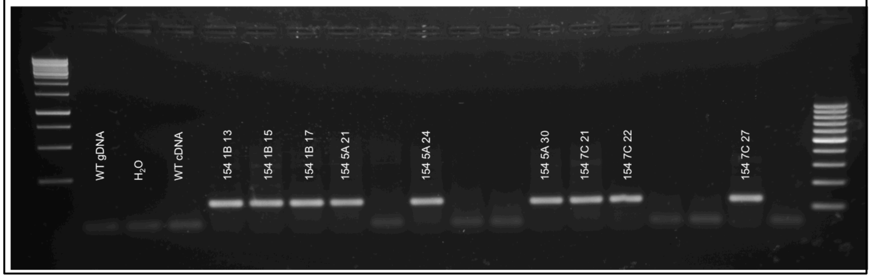

Number veins versus leaf width

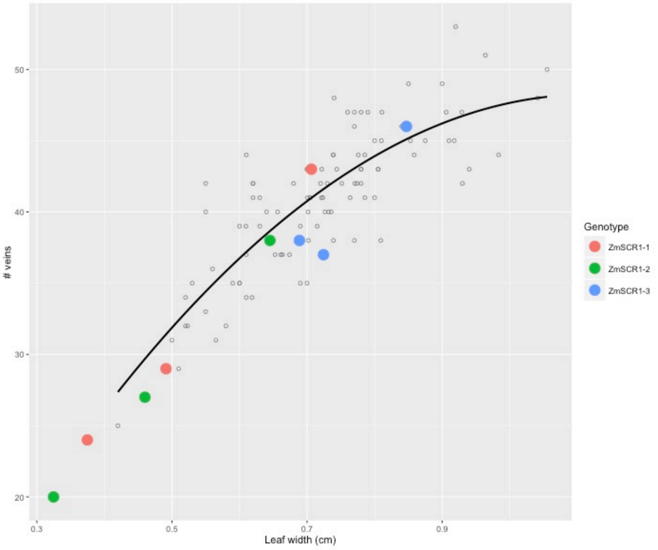

4 leftmost points (2 green, 2 orange) are not full leaf widths- veins counted across a subset of the total width as some of section not reliable.

Representative leaf cross sections:

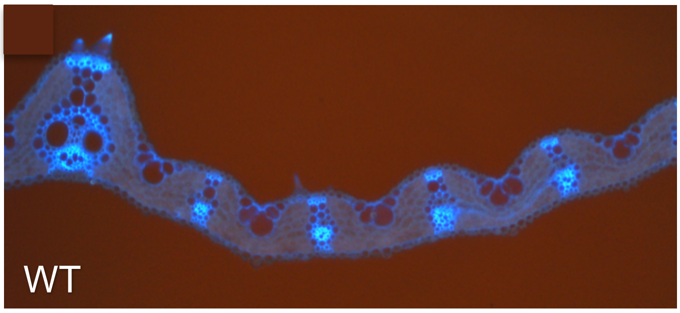

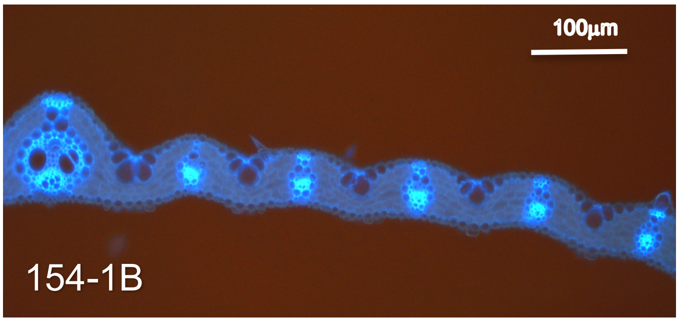

AC215201.3_FG008 **bHLH** **Gene ID: JL13**

Foliar expression

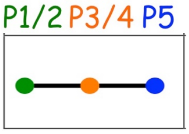

Husk expression

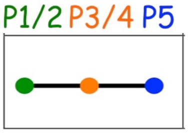

Construct: ZmUBI$_{pro}$:cDNA (ID# R1B6)

Two independent T1 lines:
13-1 (R1B618.1 – 10 individuals)
13-2 (R1B623.1 – 10 individuals)

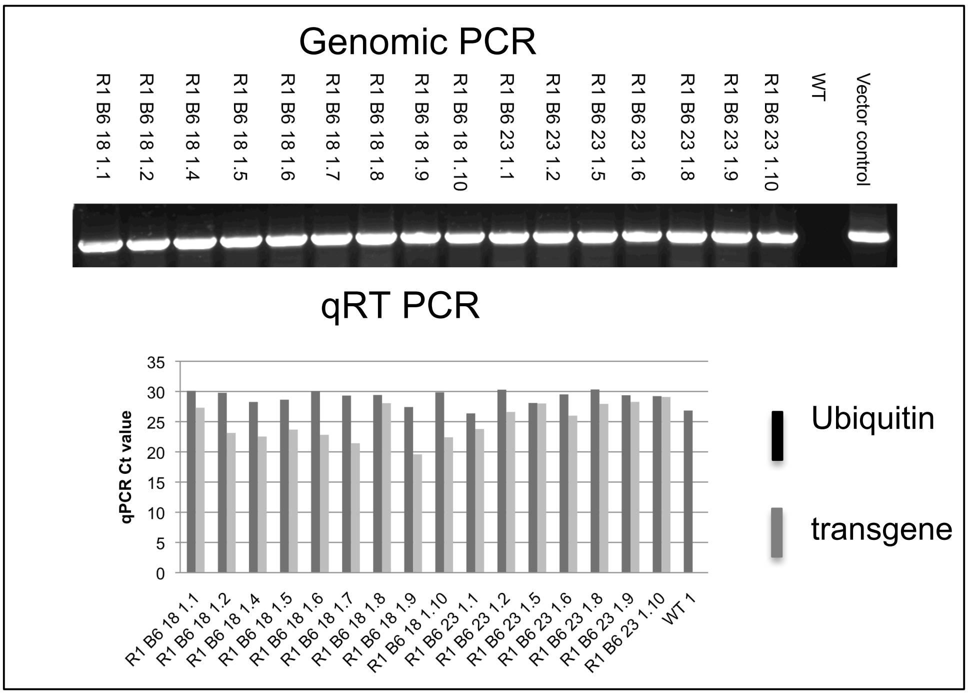

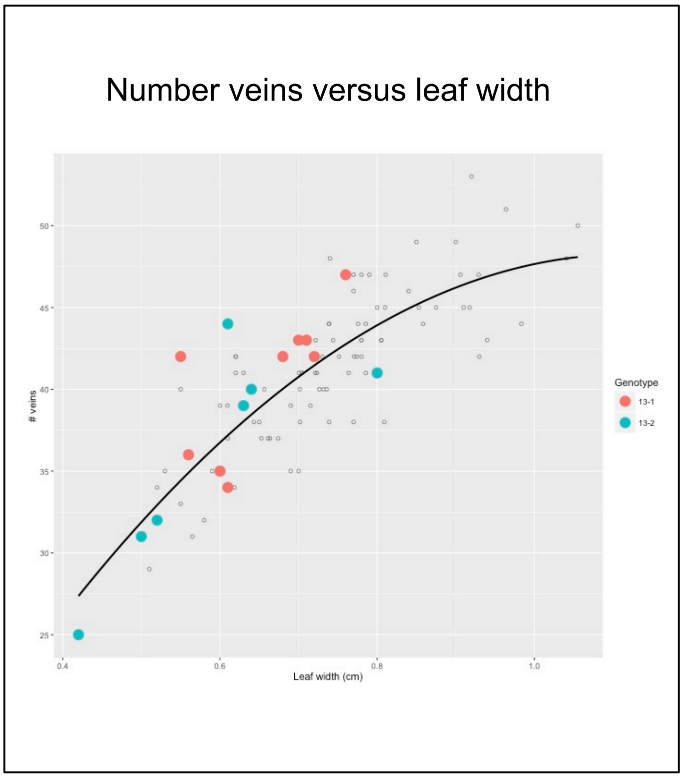

Representative leaf cross sections:

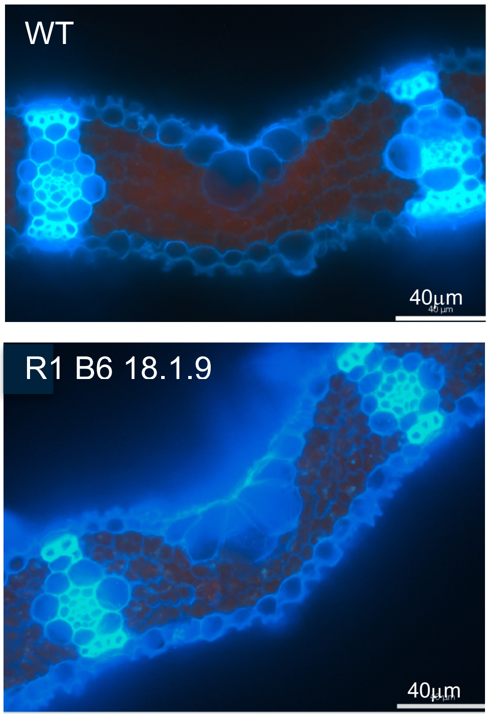

GRMZM2G016477 **LRR kinase** **Gene ID: JL15**

Foliar expression

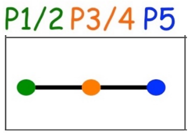

Husk expression

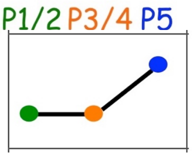

Construct: $ZmUBI_{pro}$:cDNA (ID# MS80)

Three independent T2 lines:
15-1 (MS80-10 – 1 individual)
15-2 (MS80-12 – 2 individuals)
15-3 (MS80-13 – 2 individuals)

Genomic PCR

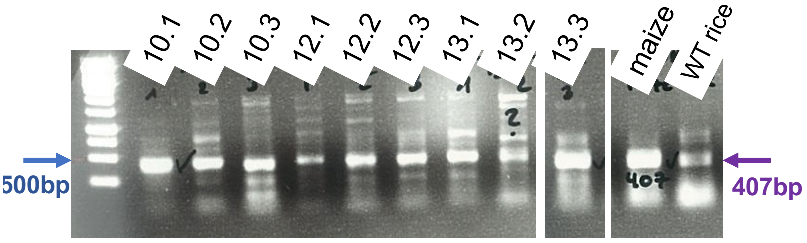

RT PCR

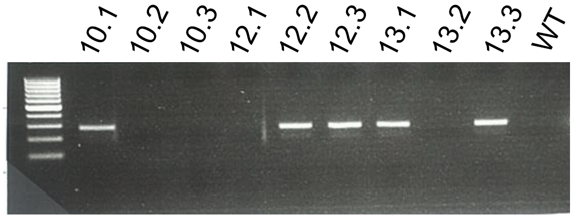

Number veins versus leaf width

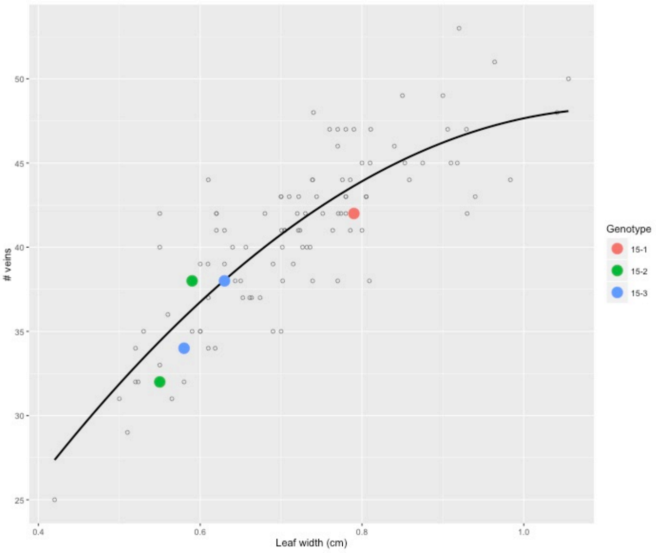

Representative leaf cross sections:

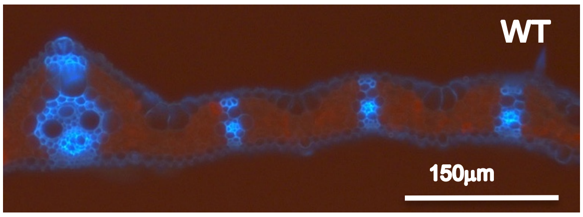

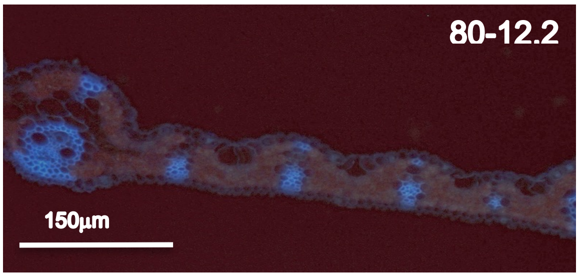

GRMZM2G119359 **Growth Regulating Factor** **Gene ID: JL26**

Foliar expression

P1/2 P3/4 P5

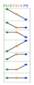

Husk expression

P1/2 P3/4 P5

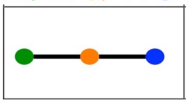

Construct: ZmUBI$_{pro}$:cDNA (ID# 70CC)

Two independent T2 lines:
26-1 (70CCS3 – 5 individuals)
26-2 (70CCS101 – 7 individuals)

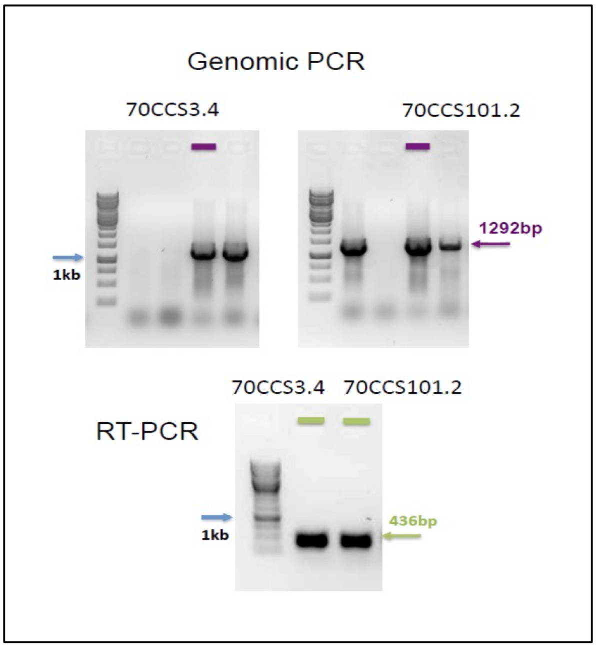

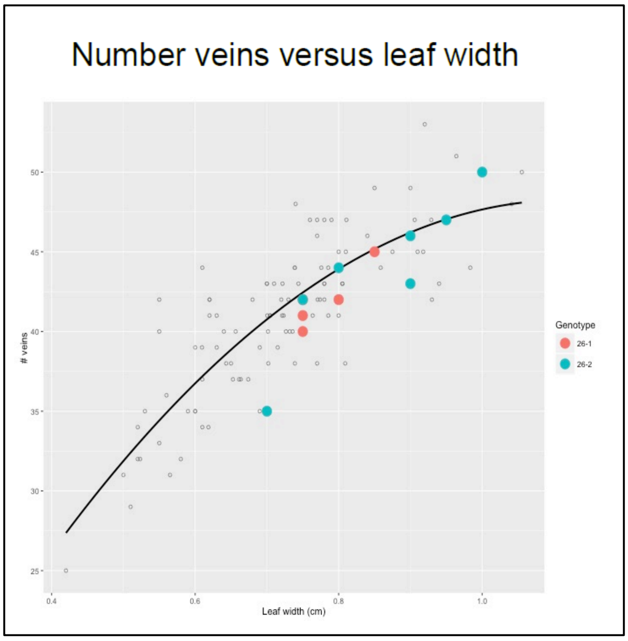

Representative leaf cross sections:

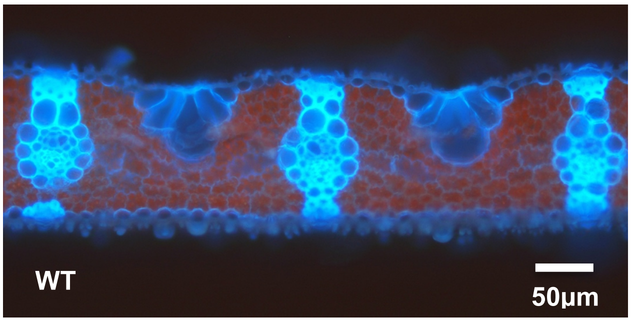

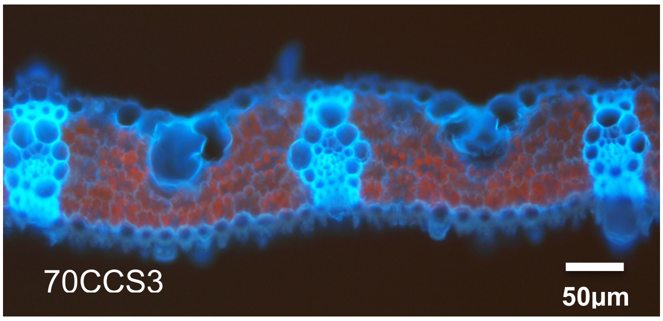

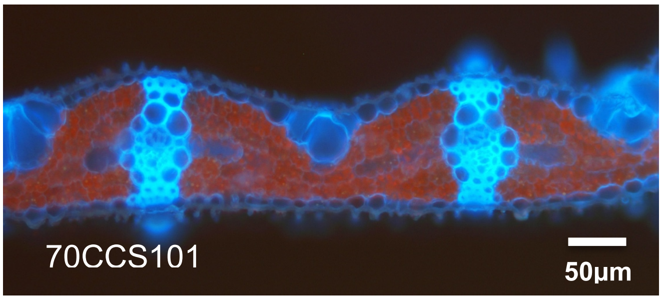

GRMZM5G893117 **Growth Regulating Factor** **Gene ID: JL27**

Foliar expression

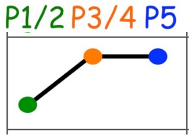

Husk expression

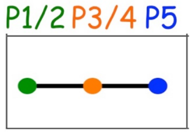

Construct: ZmUBI$_{pro}$:cDNA (ID# 71CC)
ZmUBI$_{pro}$:gDNA (ID# 71GC)

Four independent T1 lines:
27-1 (71CCS11 – 1 individual)
27-2 (71CCS16 – 1 individual)
27-3 (71GCS1 – 3 individuals)
27-4 (71GCS3 – 7 individuals)

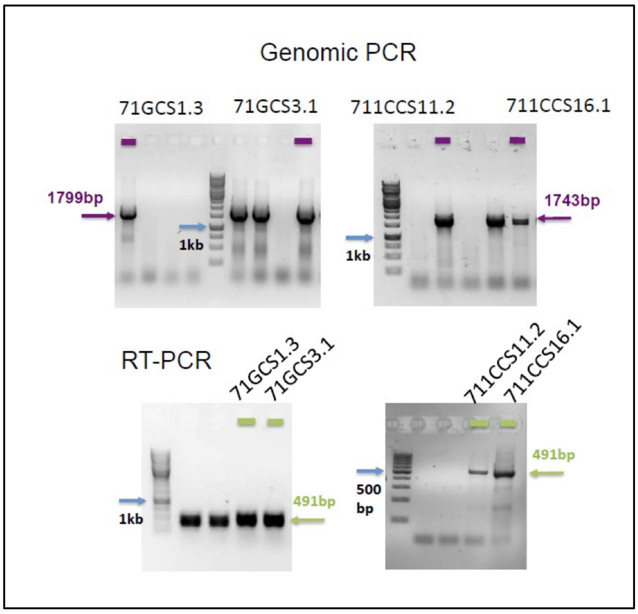

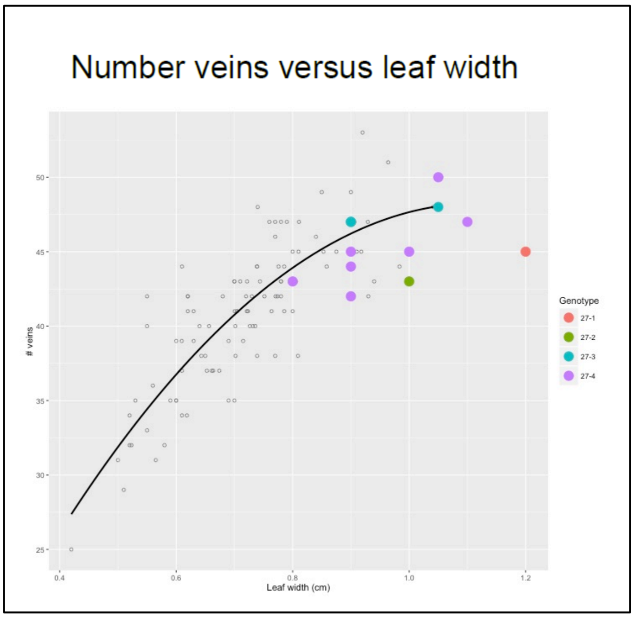

Representative leaf cross sections:

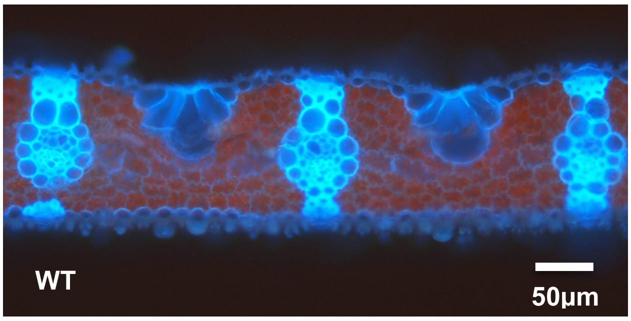

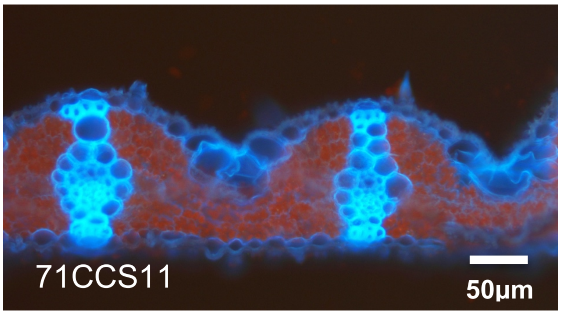

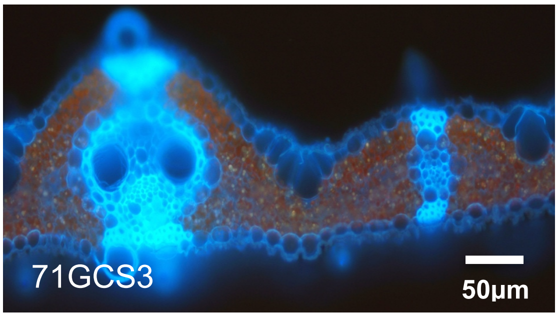

GRMZM2G178182 **bHLH** **Gene ID: JL29**

Foliar expression Husk expression

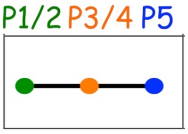

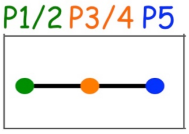

Construct: ZmUBI$_{pro}$:cDNA (ID# 73CC)

Two independent T1 lines:
29-1 (73CCS1 – 6 individuals)
29-2 (73CCSR19 – 3 individuals)

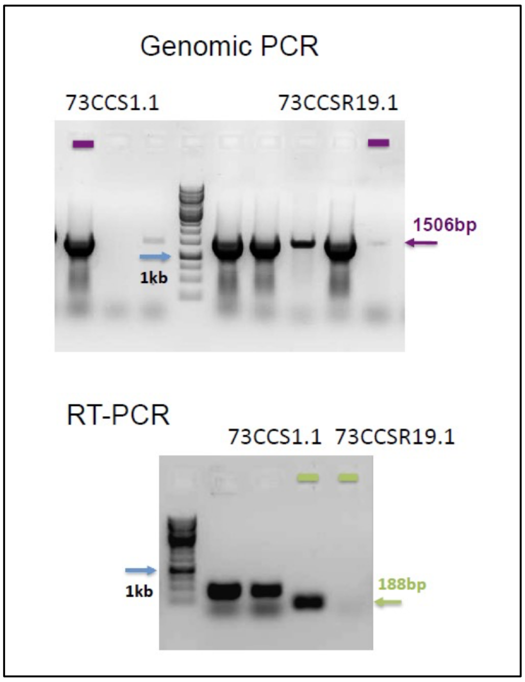

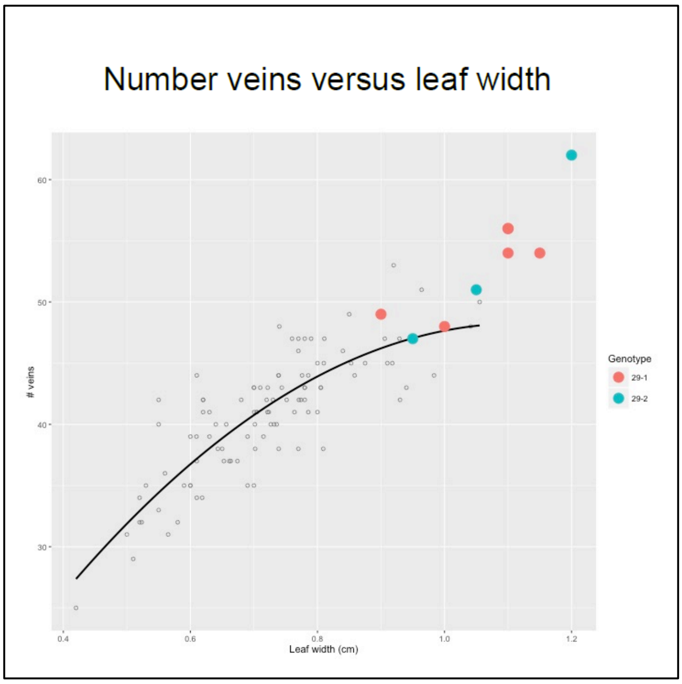

Representative leaf cross sections:

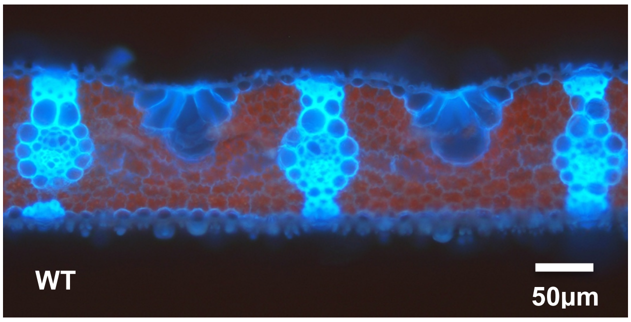

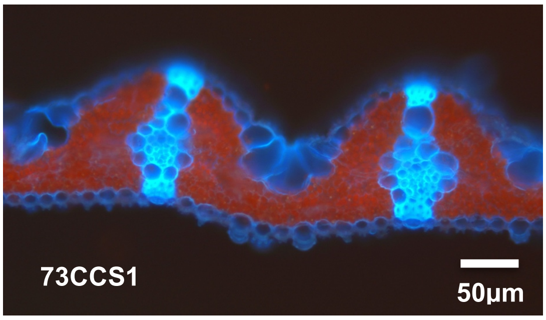

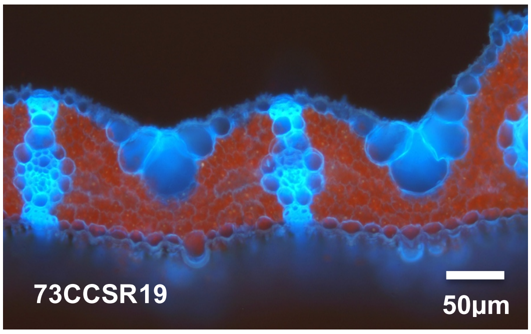

GRMZM2G140669 **GATA ZnF** **Gene ID: JL30**

Foliar expression

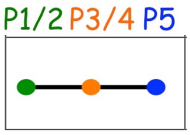

Husk expression

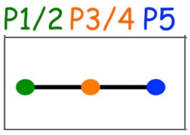

Construct: $ZmUBI_{pro}$:cDNA (ID# 74CC)

Two independent T1 lines:
30-1 (74CCS1 – 1 individual)
30-2 (74CCS10 – 1 individual)

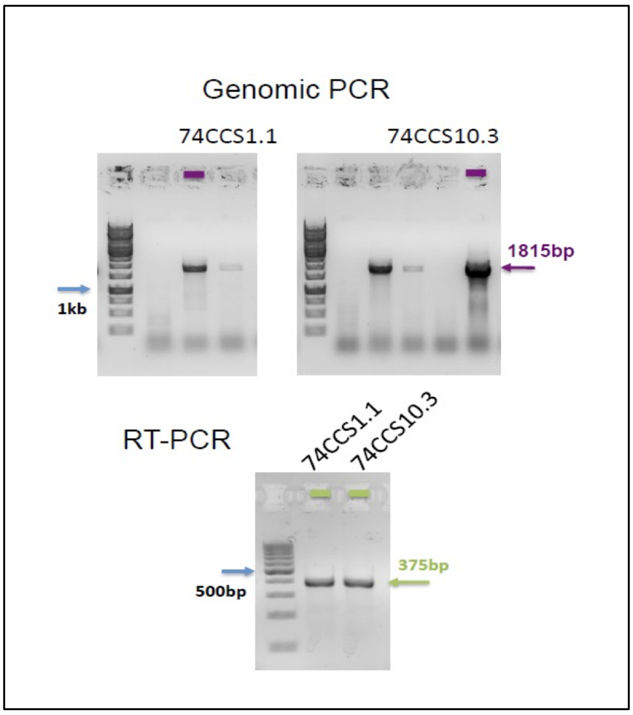

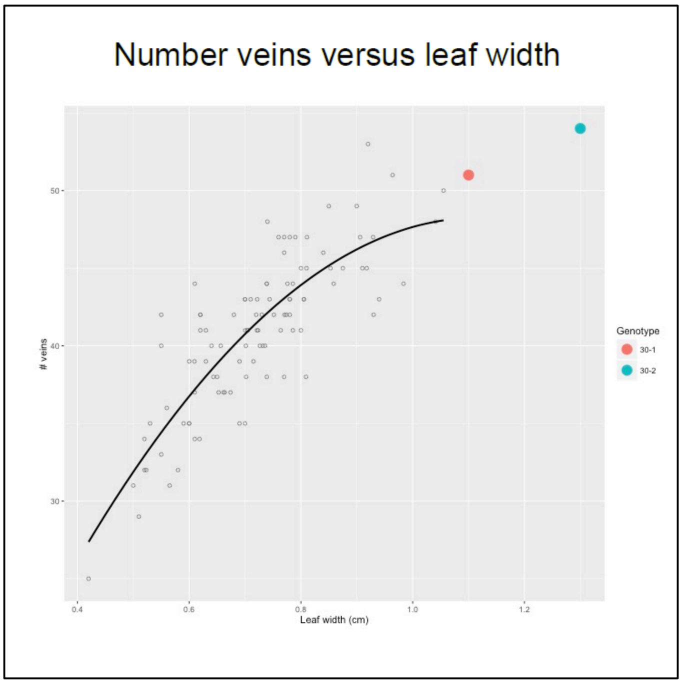

Representative leaf cross sections:

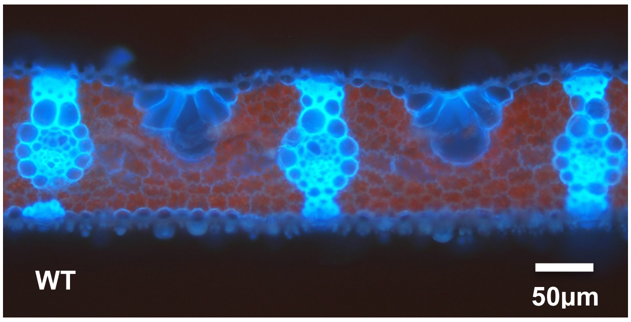

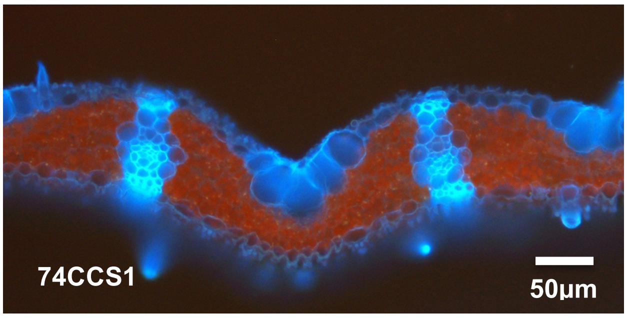

GRMZM2G472945 **TUBBY-like** **Gene ID: JL31**

Foliar expression

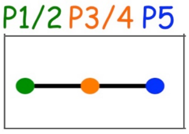

Husk expression

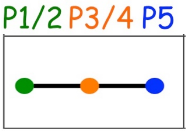

Construct: ZmUBI$_{pro}$:cDNA (ID# 75CC)

Two independent T1 lines:
31-1 (75CCS6 – 3 individuals)
31-1 (75CCSR15 – 11 individuals)

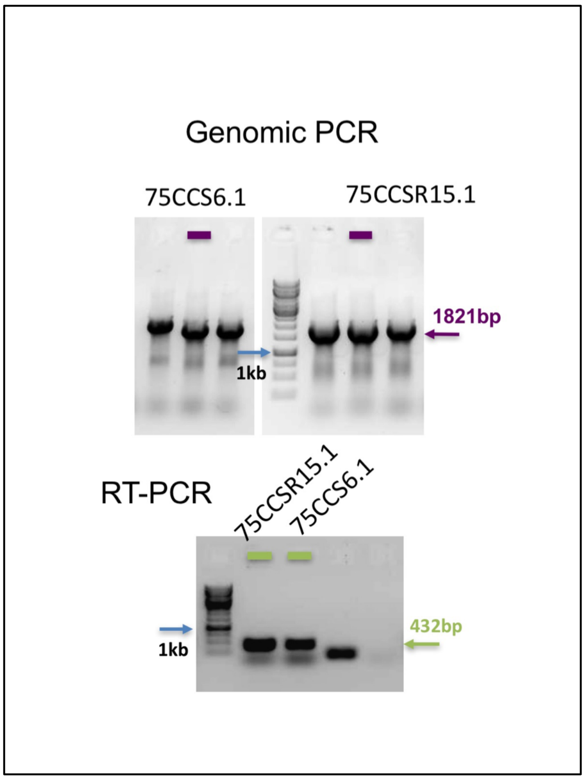

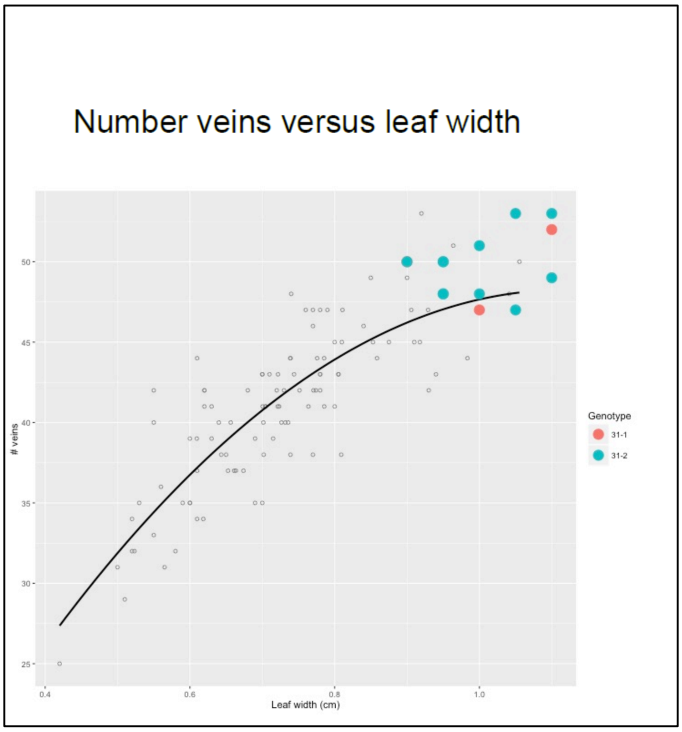

Representative leaf cross sections:

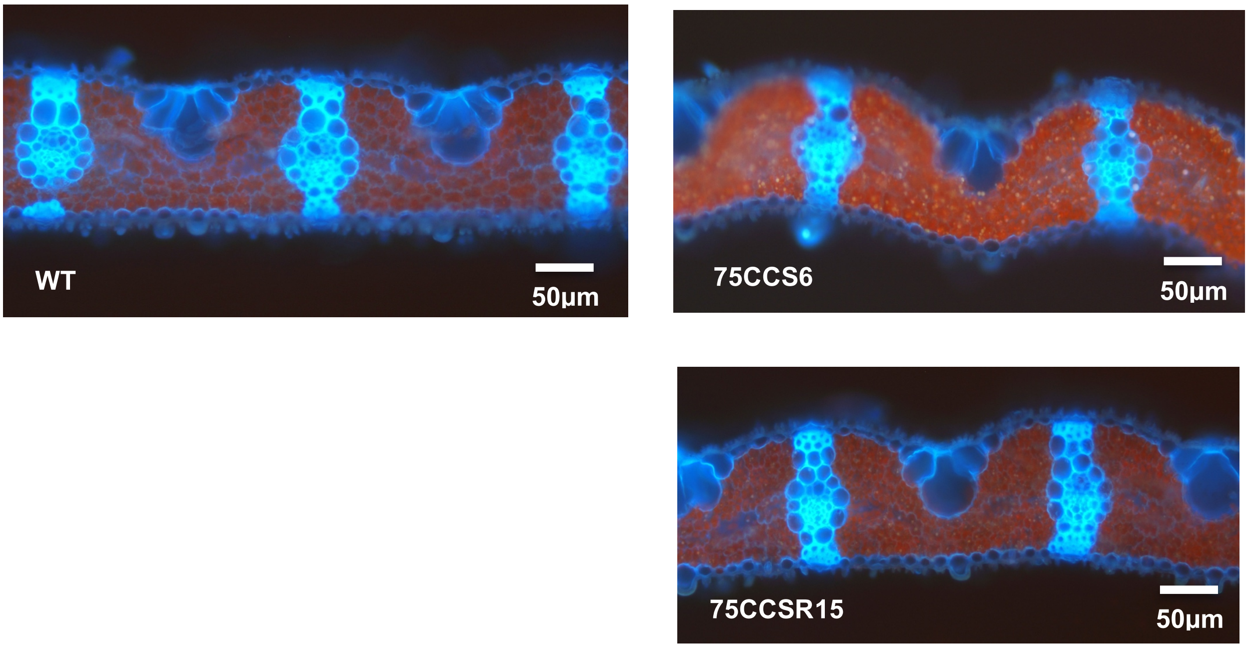

GRMZM2G146688 **AP2** **Gene ID: JL32**

Foliar expression

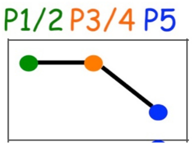

Husk expression

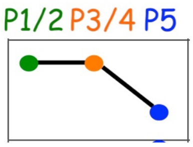

Construct: ZmUBI$_{pro}$:cDNA (ID# 77CC)
ZmUBI$_{pro}$:gDNA (ID# 77GC)

Four independent T1 lines: This gene has been transformed with both CDS (77CC) and genomic sequence (77GC), but no gene expression was detected in either attempt.
32-1 (77CCSR10 – 5 individuals)
32-2 (77CCSR8 – 6 individuals)
32-3 (77GCS1 – 1 individual)
32-4 (77GCS18 – 1 individual)

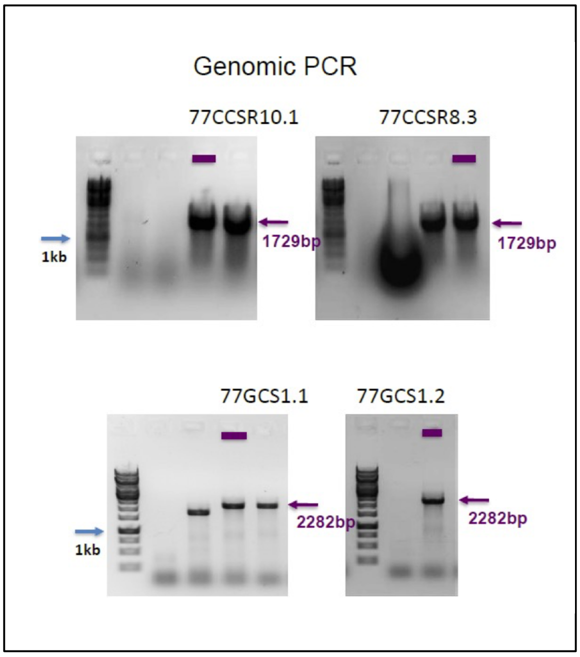

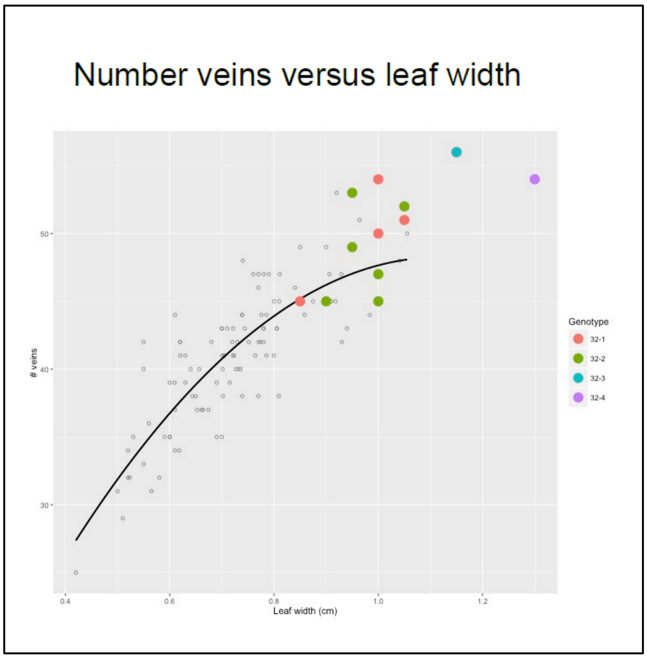

Representative leaf cross sections:

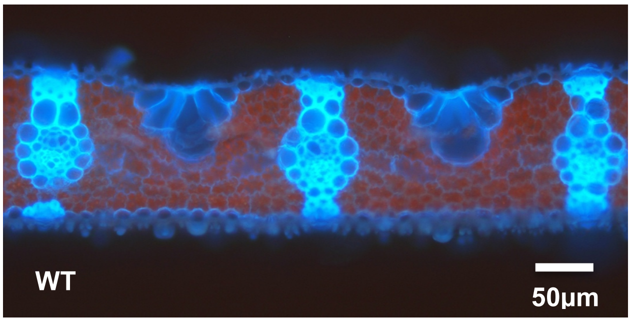

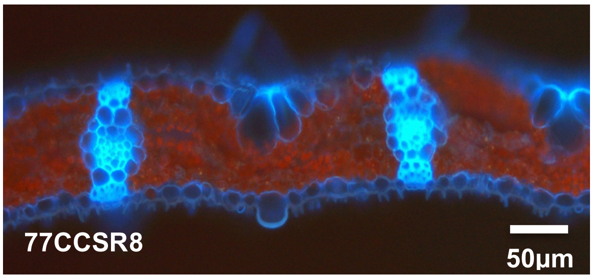

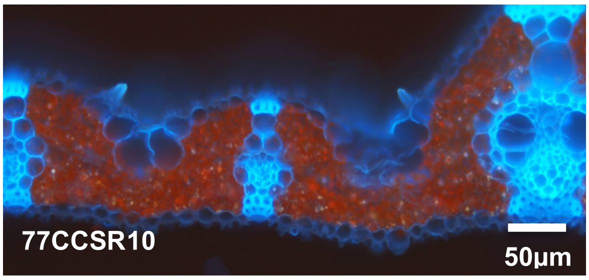

GRMZM2G023051 **Unknown** **Gene ID: JL35**

Foliar expression

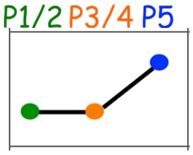

Husk expression

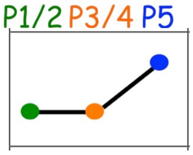

Construct: ZmUBI$_{pro}$:cDNA (ID# 81CC)

Two independent T1 lines:
35-1 (81CCS7 – 8 individuals)
35-2 (81CCS2 – 9 individuals)

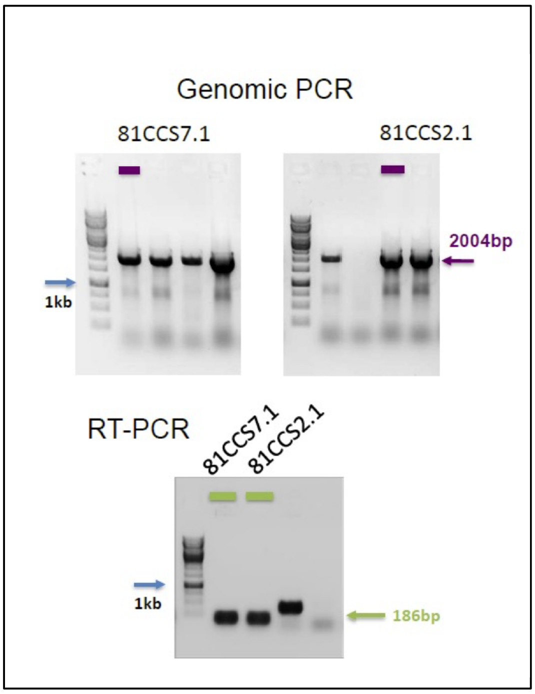

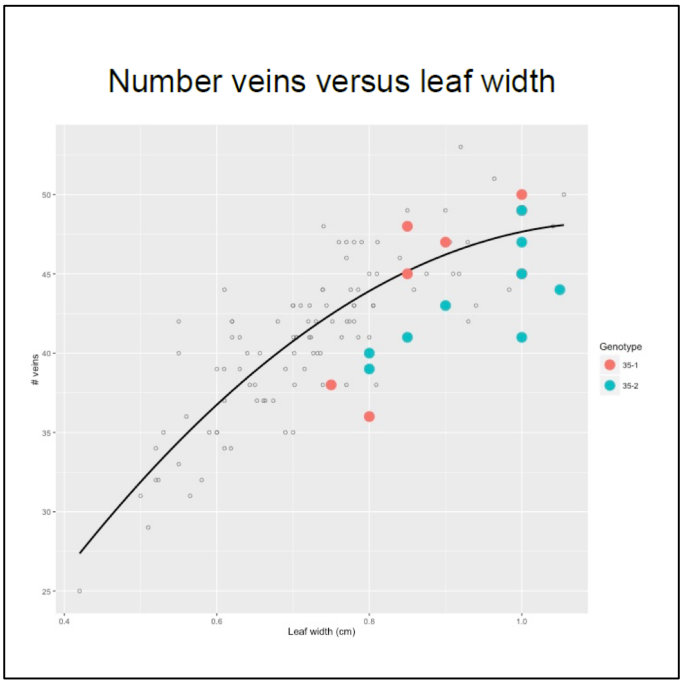

Representative leaf cross sections:

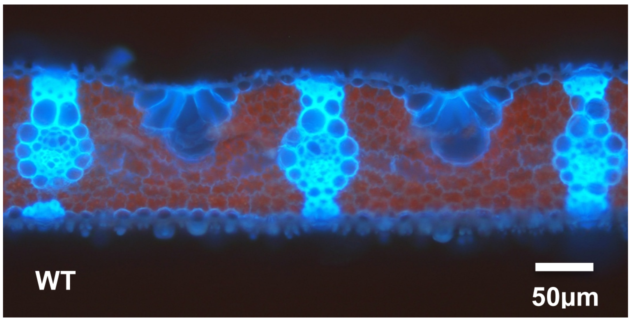

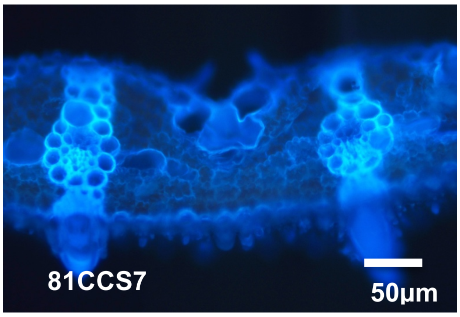

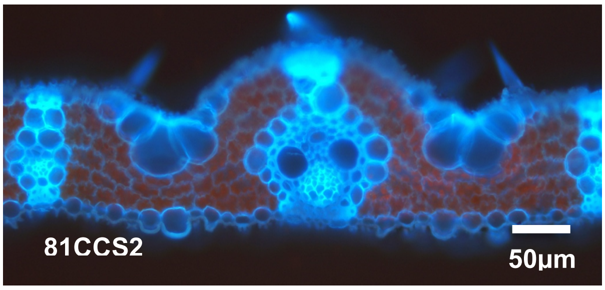

GRMZM2G109480 **Unknown** **Gene ID: JL36**

Foliar expression

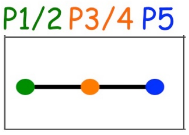

Husk expression

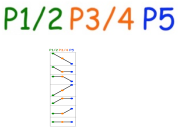

Construct: ZmUBI$_{pro}$:cDNA (ID# 82CC)

Two independent T1 lines:
36-1 (82CCS1 – 3 individuals)
36-2 (82CCS2 – 4 individuals)

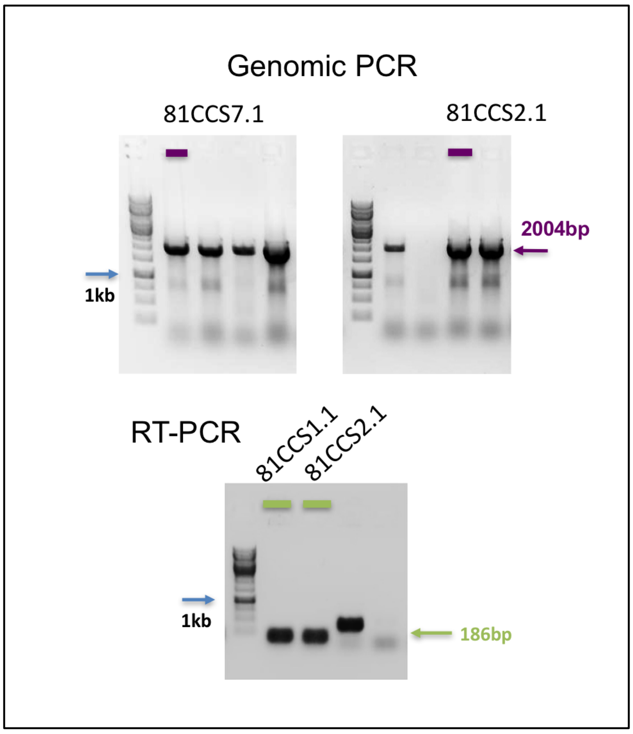

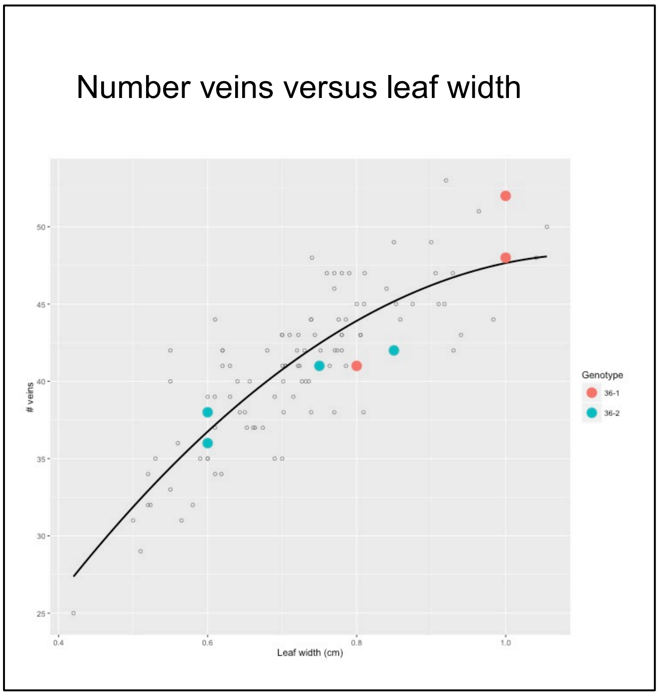

Representative leaf cross sections:

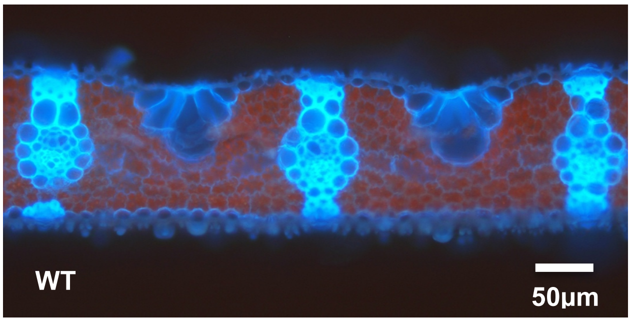

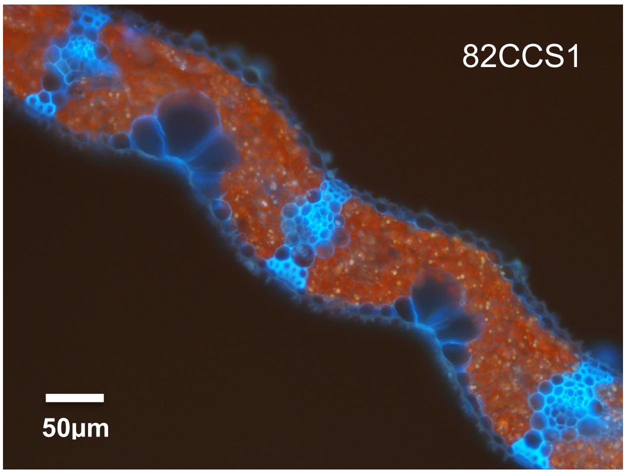

GRMZM2G097275 **SBP** **Gene ID: JL47**

Foliar expression

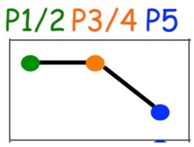

Husk expression

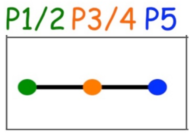

Construct: ZmUBI$_{pro}$:cDNA (ID# 89CC)

Two independent T1 lines:
47-1 (89CCS105 – 4 individuals)
47-2 (89CCS107 – 2 individuals)

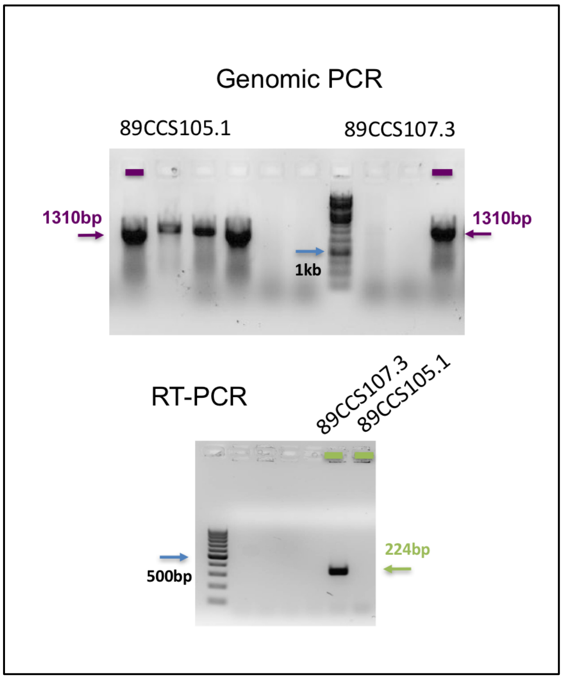

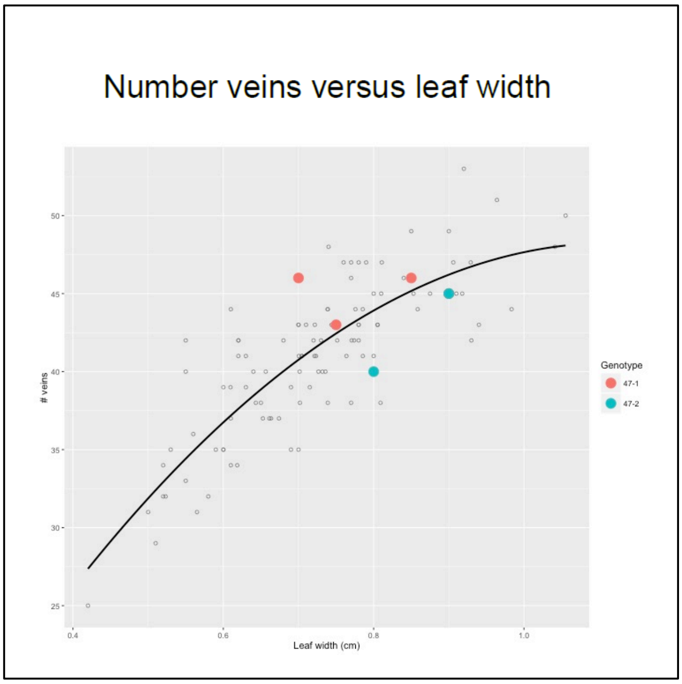

Representative leaf cross sections:

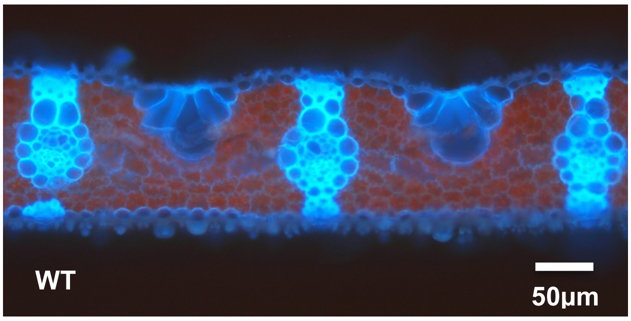

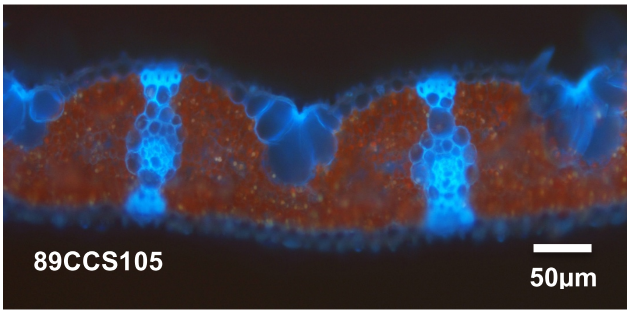

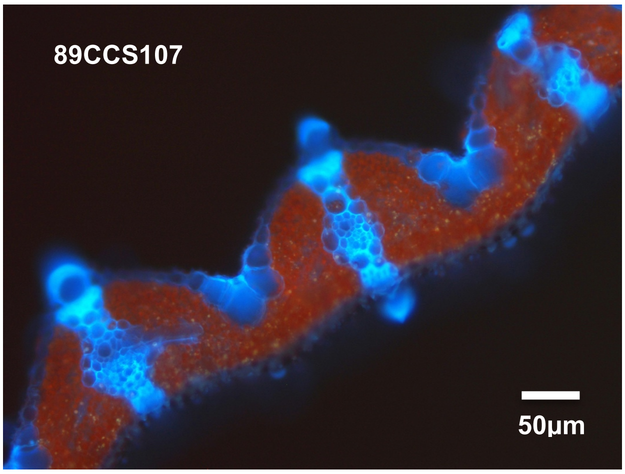

GRMZM2G148467 **SBP** **Gene ID: JL48**

Foliar expression

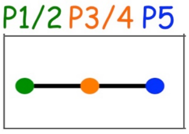

Husk expression

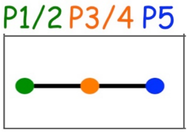

Construct: ZmUBI$_{pro}$:cDNA (ID# 88CC)

Two independent T1 lines:
48-1 (88CCS1 – 2 individuals)
48-2 (82CCS3 – 7 individuals)

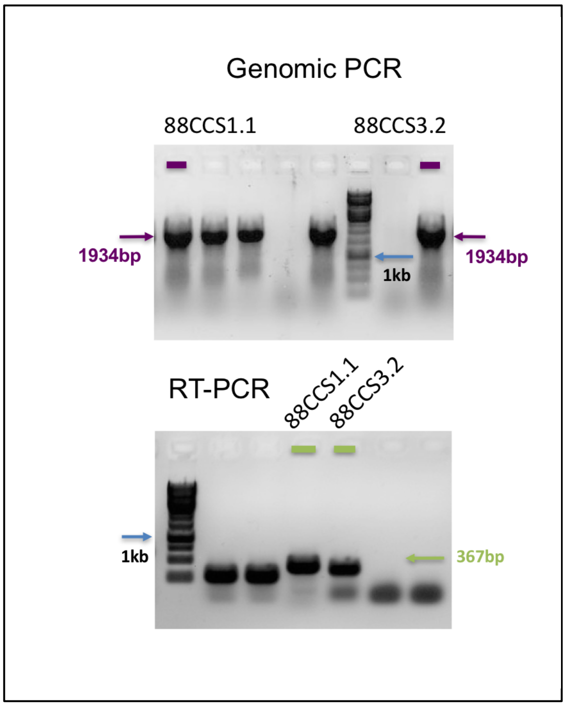

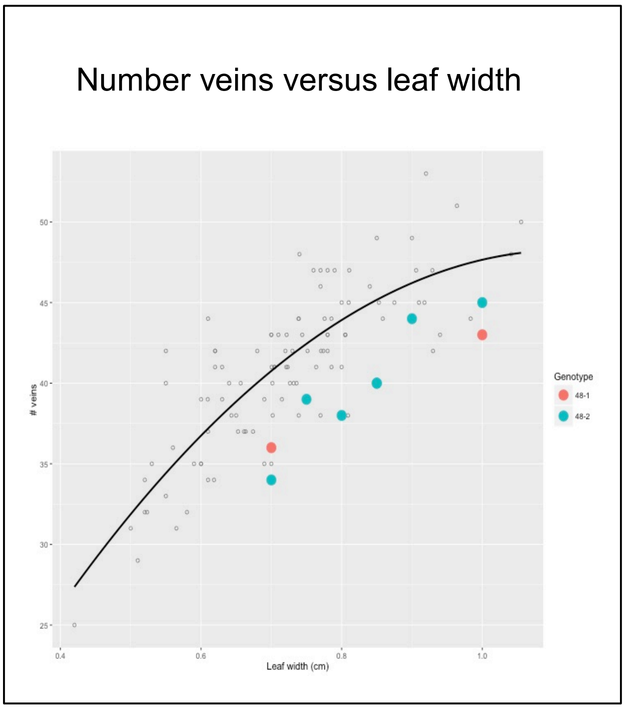

Representative leaf cross sections:

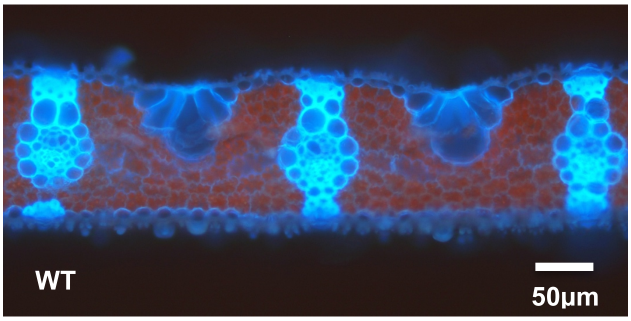

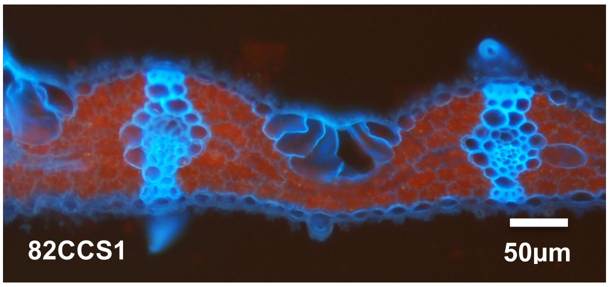

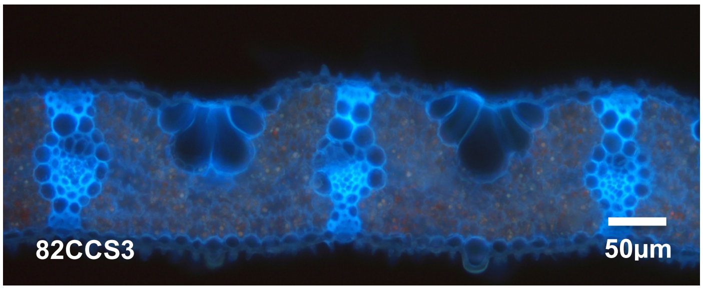

GRMZM2G028643 **LRR kinase** **Gene ID: JL49**

Foliar expression

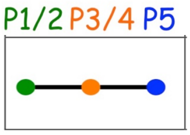

Husk expression

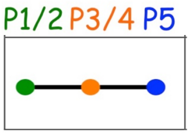

Construct: ZmUBI$_{pro}$:cDNA (ID# 86CC)

Two independent T1 lines:
49-1 (86CCS1 – 6 individuals)
49-2 (86CCSR3 – 5 individuals)

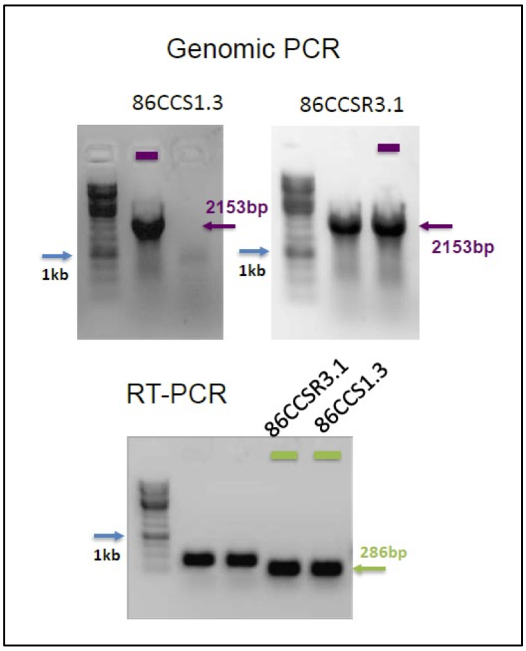

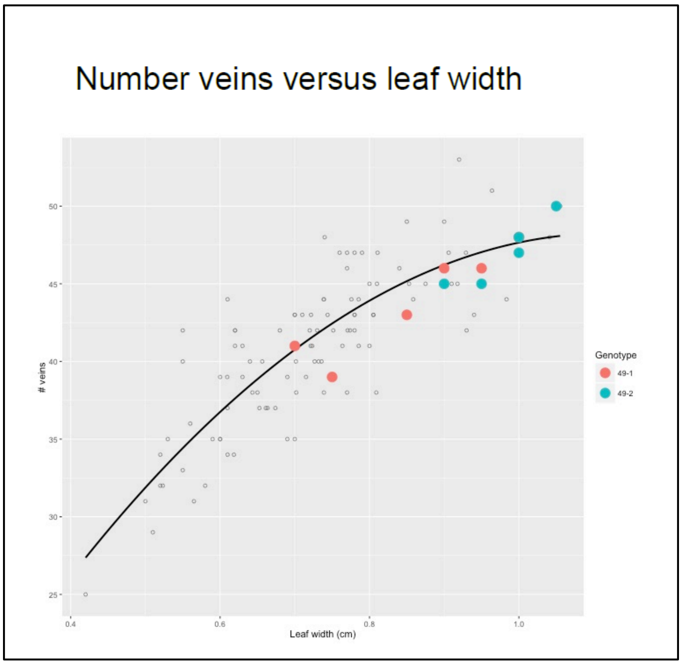

Representative leaf cross sections:

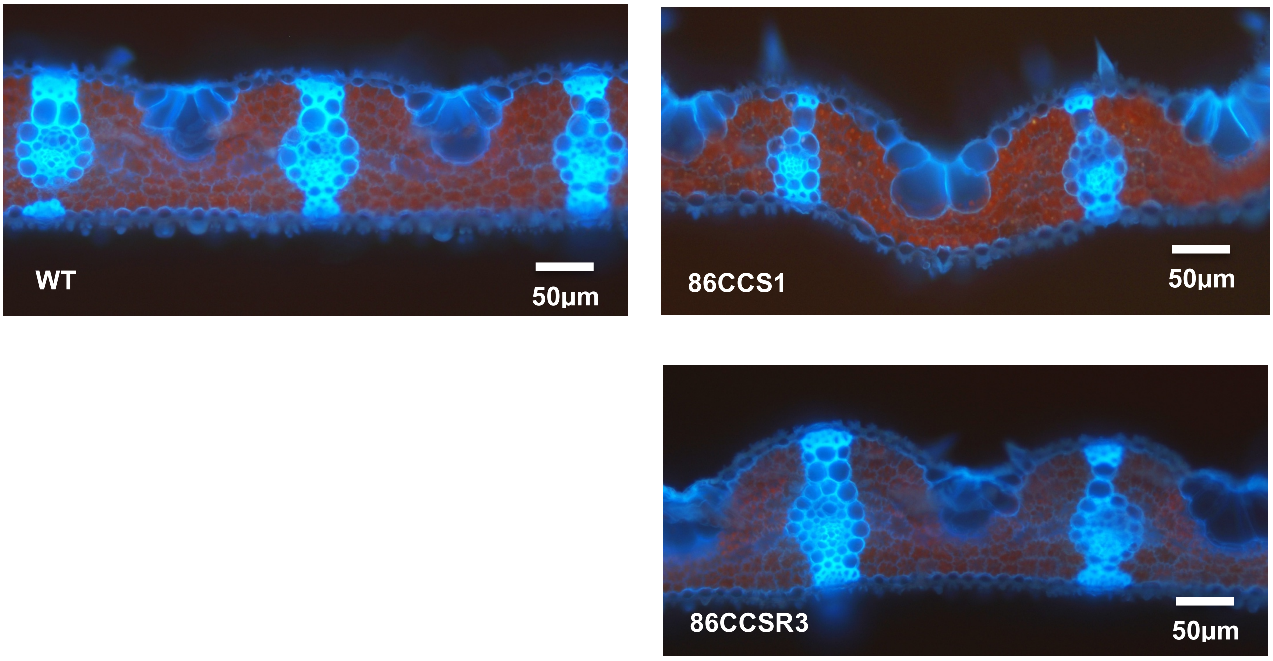

GRMZM2G178102 **Class III-HD ZIP** **Gene ID: JL51**

Foliar expression

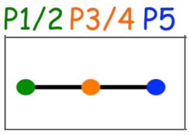

Husk expression

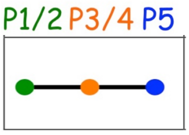

Construct: ZmUBI$_{pro}$:cDNA (ID# 87CC)

Two independent T1 lines:
51-1 (87CCS1 – 3 individuals)
51-2 (87CCS2 – 2 individuals)

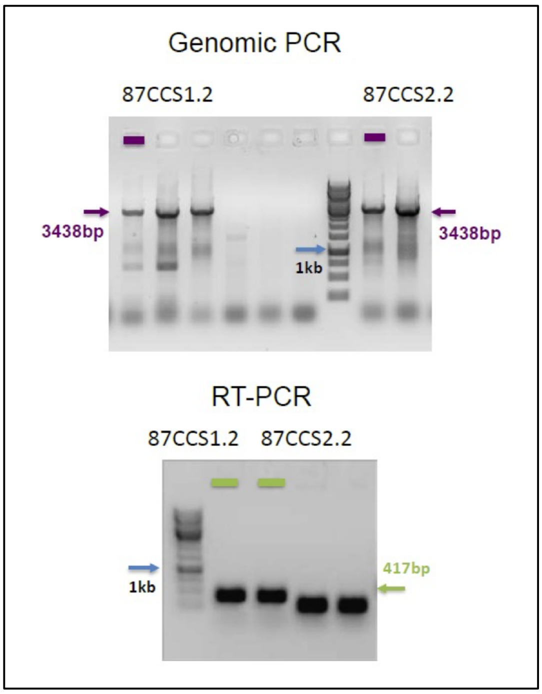

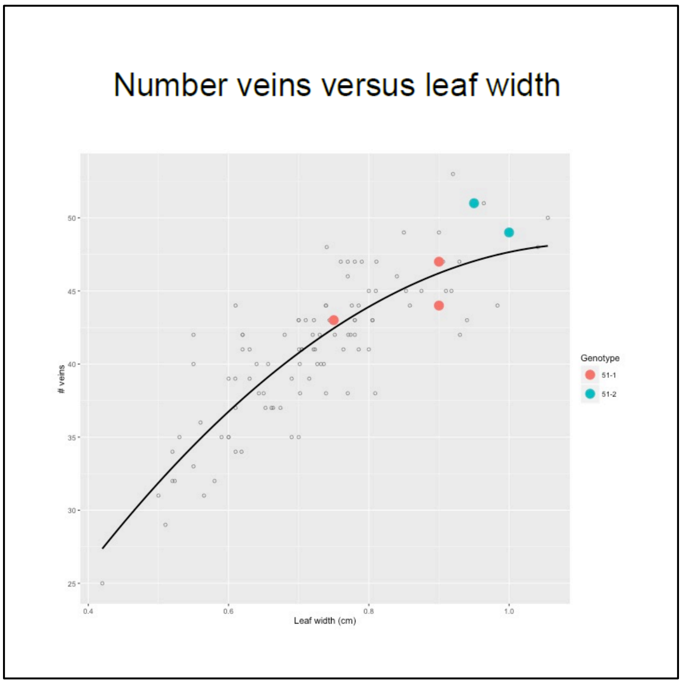

Representative leaf cross sections:

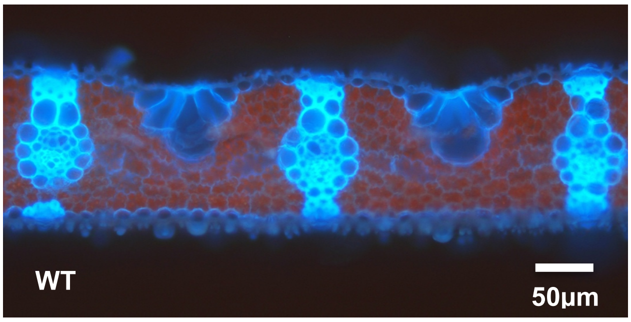

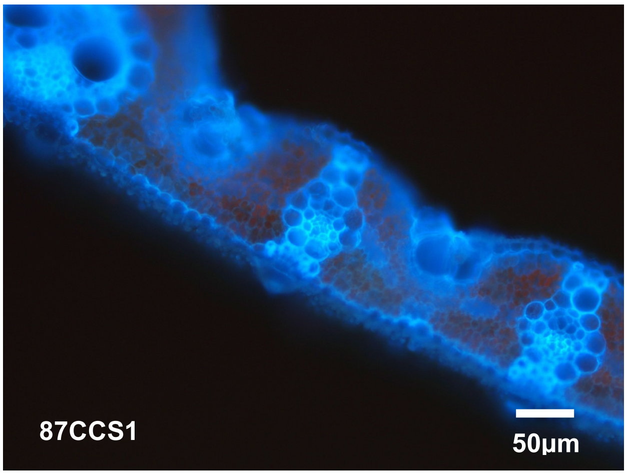

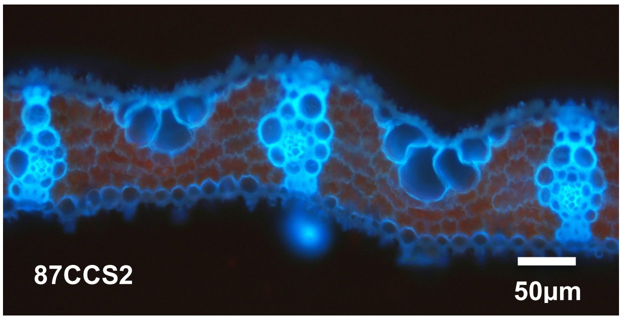

GRMZM2G061314 **LRR binding protein** **Gene ID: JL60**

Foliar expression

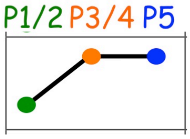

Husk expression

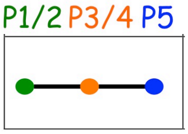

Construct: ZmUBI$_{pro}$:cDNA (ID# 945)

Three independent T1 lines:
60-1 (IRS945-19 – 1 individual)
60-2 (IRS945-20 – 1 individual)
60-3 (IRS945-11 – 1 individual)

Southern blot

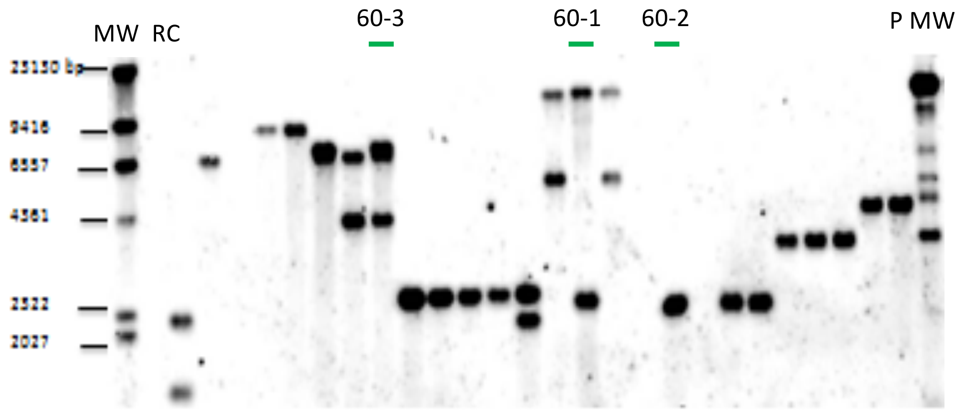

Number veins versus leaf width

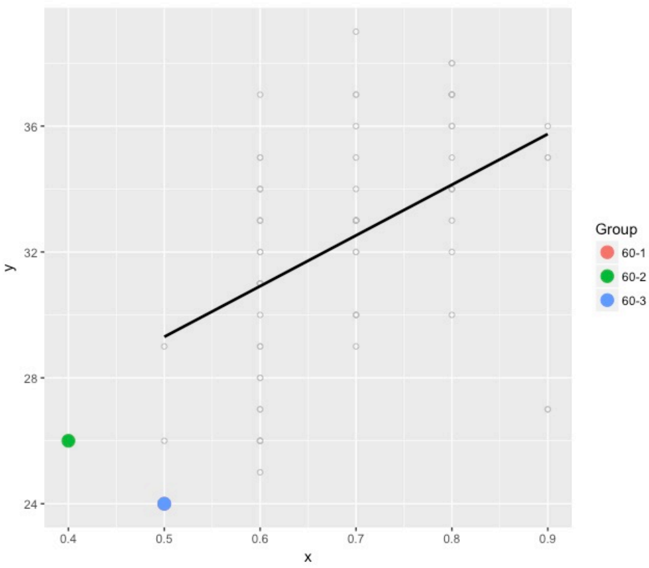

Representative cross section of segregating WT leaf

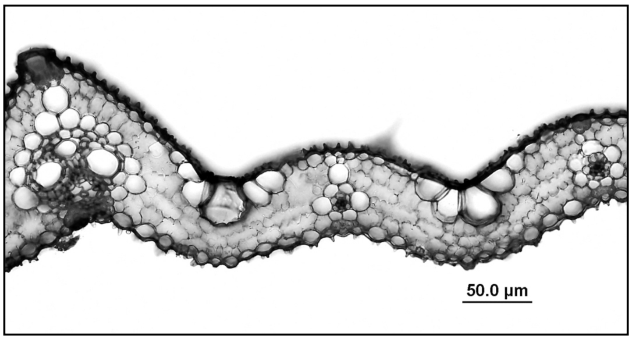

Representative cross section of line 60-3 leaf

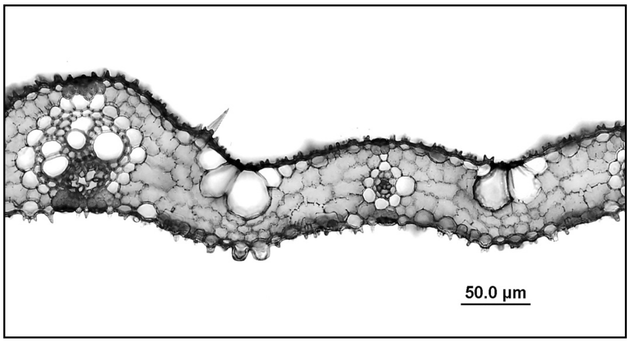

GRMZM2G089819 **Brassinosteroid LRR RLK** **Gene ID: JL68**

Foliar expression

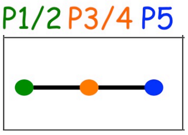

Husk expression

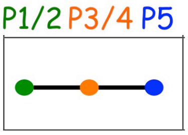

Construct: ZmUBI$_{pro}$:cDNA (ID# 953)

Six independent T1 lines:

68-1 (IRS953-1 – 1 individual)
68-2 (IRS953-2 – 1 individual)
68-3 (IRS953-3 – 3 individuals)
68-4 (IRS953-6 – 2 individuals)
68-5 (IRS953-7 – 1 individual)
68-6 (IRS953-10 – 1 individual)

Genomic PCR

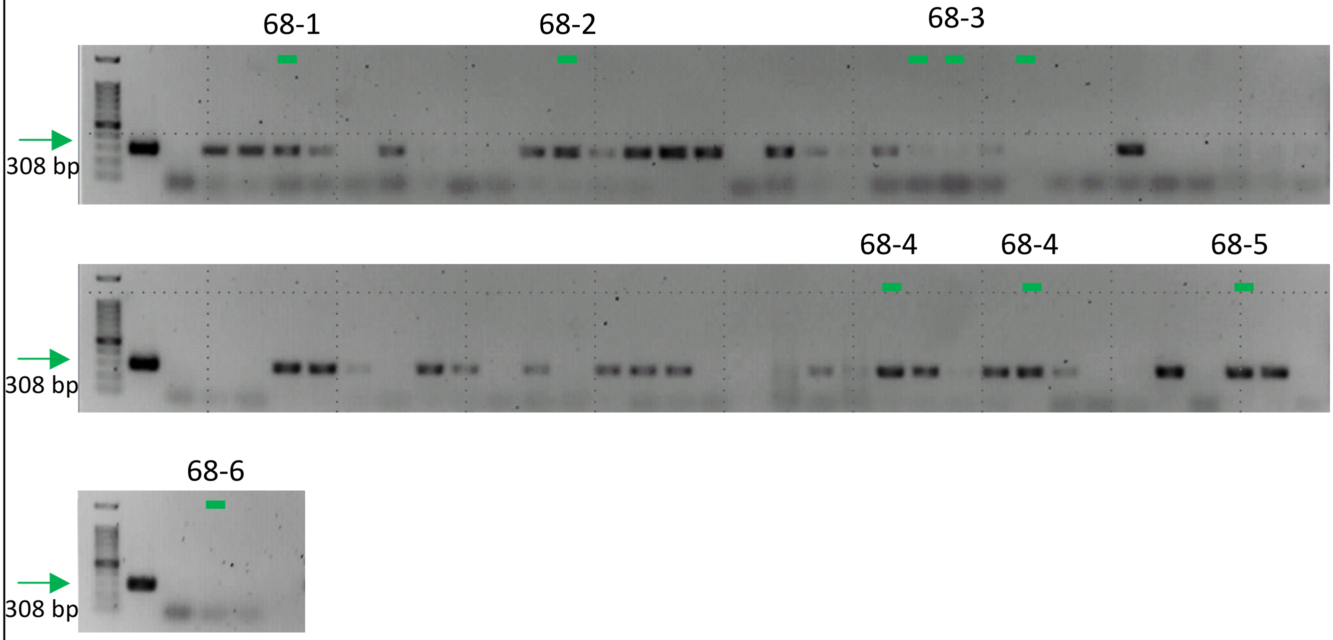

Number veins versus leaf width

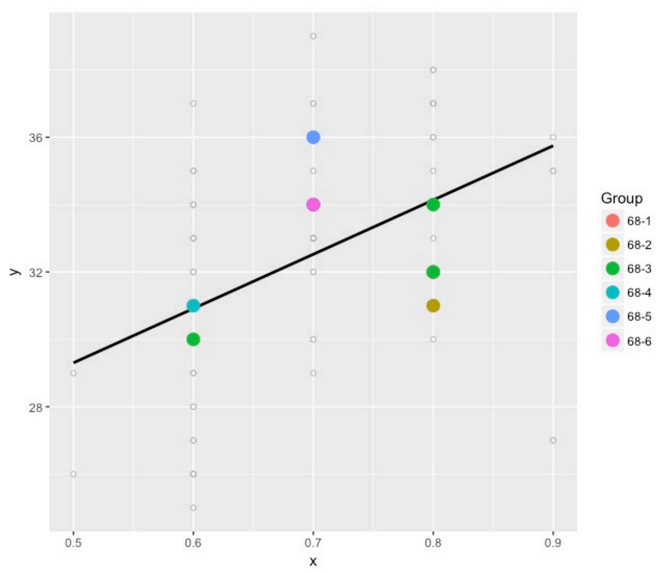

Representative cross section of segregating WT leaf

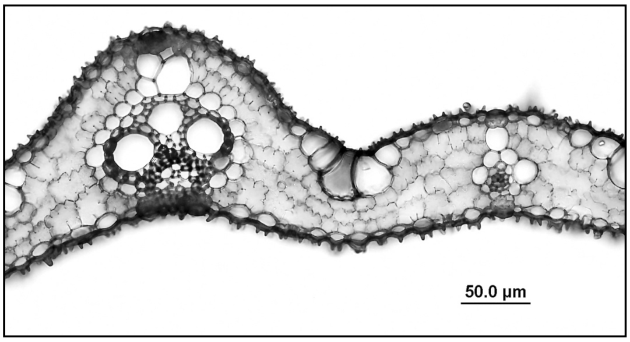

Representative cross section of line 68-6 leaf

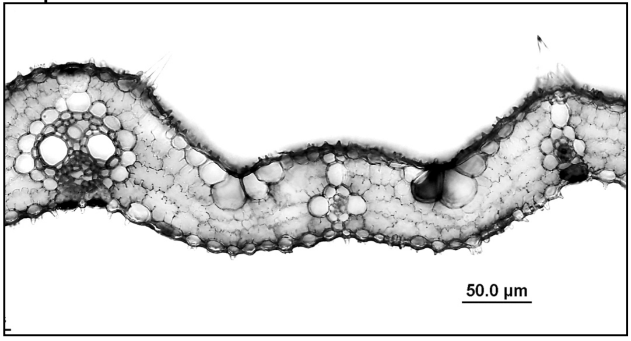

GRMZM5G850129 **Growth Regulatory Factor** **Gene ID: JL75**

Foliar expression

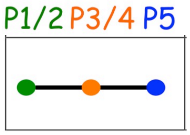

Husk expression

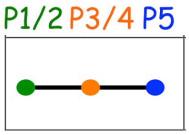

Construct: ZmUBI$_{pro}$:cDNA (ID# 948)

Two independent T1 lines:
75-1 (IRS948-19 – 6 individuals)
75-2 (IRS948-23 – 2 individuals)

Genomic PCR

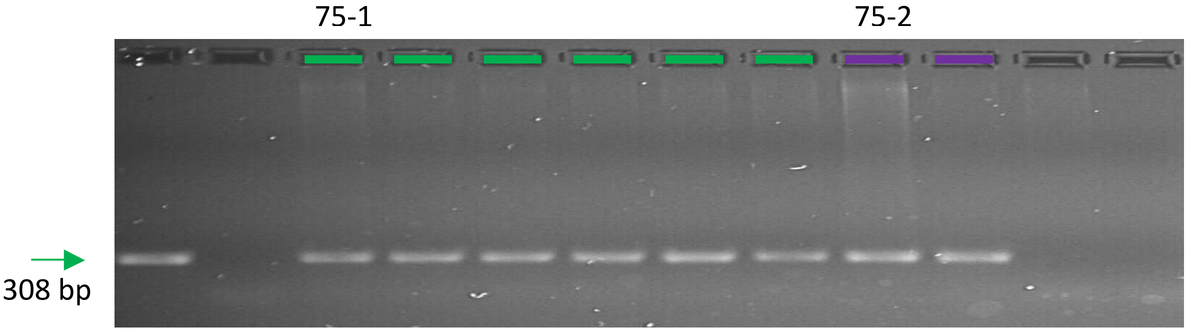

Number veins versus leaf width

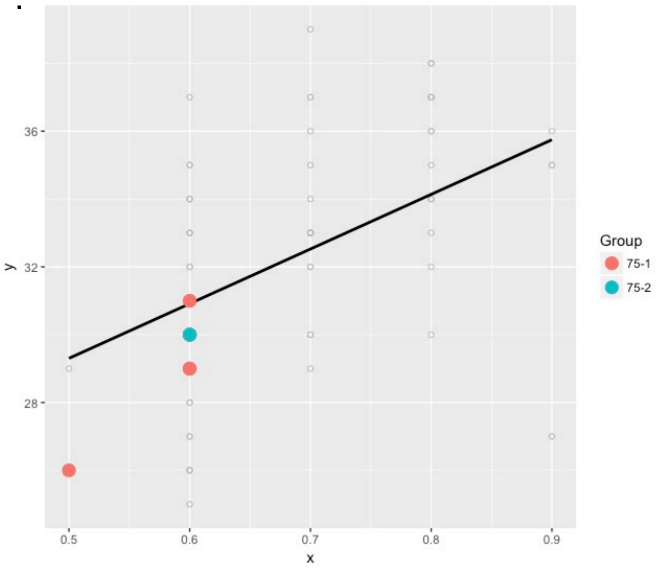

Representative cross section of segregating WT leaf

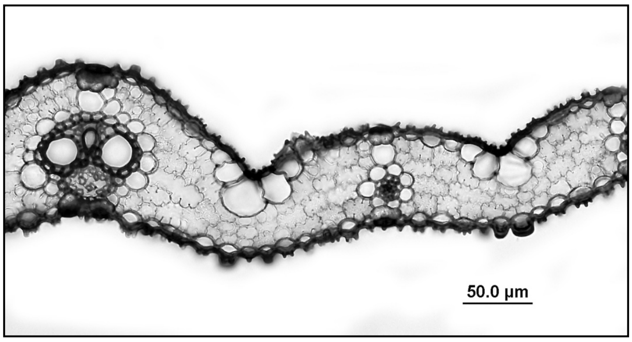

Representative cross section of line 75-2 leaf

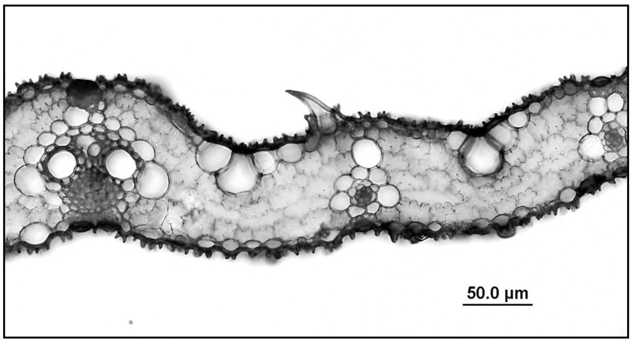

GRMZM2G061734 **SBP domain** **Gene ID: JL76**

Foliar expression

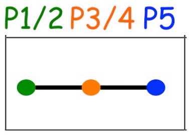

Husk expression

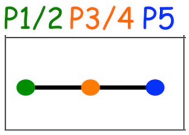

Construct: ZmUBI$_{pro}$:cDNA (ID# 960)

Two independent T1 lines:
76-1 (IRS960-20 – 2 individuals)
76-2 (IRS960-22 – 2 individuals)

Genomic PCR

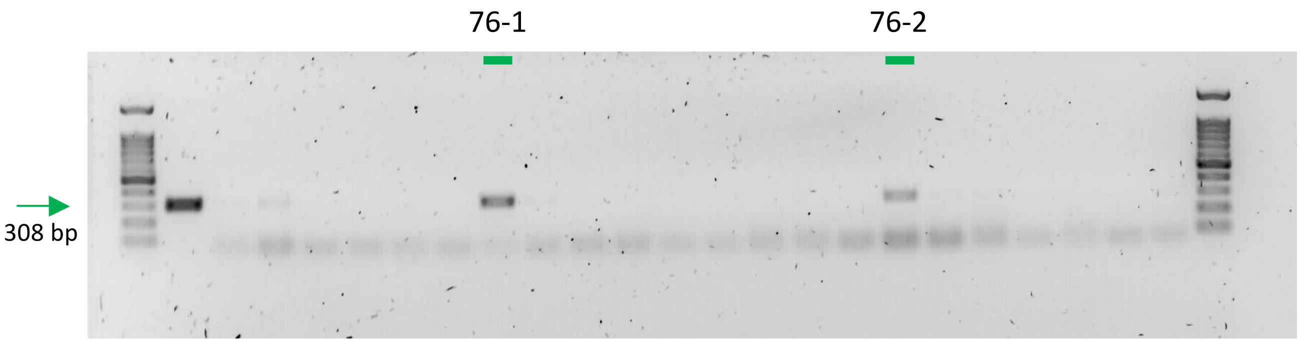

Number veins versus leaf width

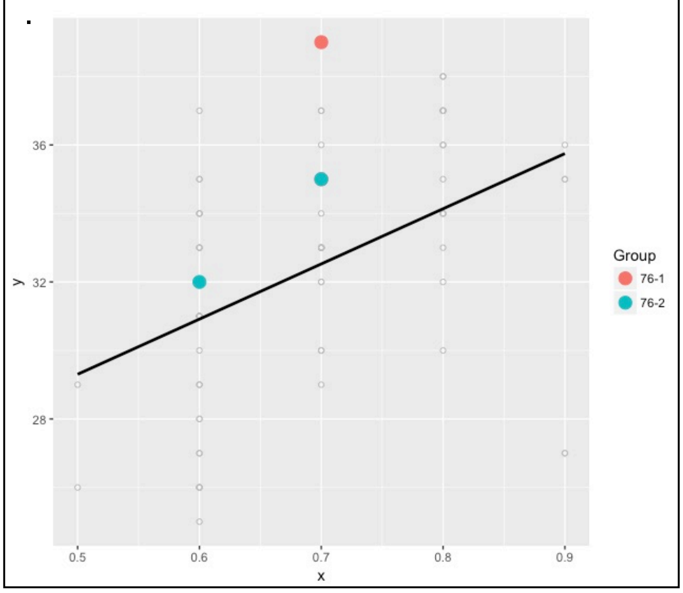

Representative cross section of segregating WT leaf

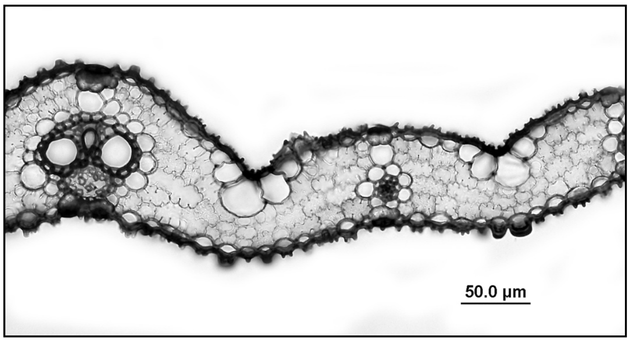

Representative cross section of line 76-2 leaf

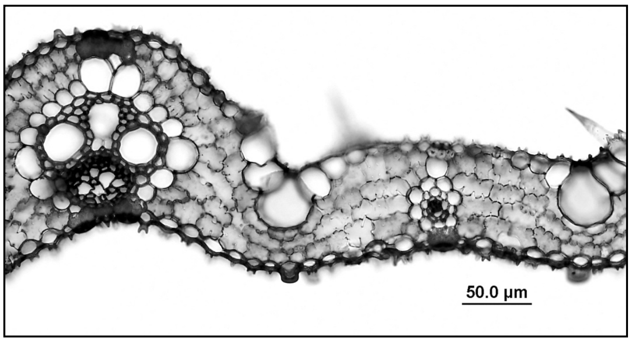

GRMZM2G126018 **SBP domain** **Gene ID: JL80**

Foliar expression

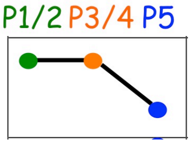

Husk expression

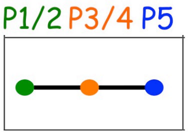

Construct: ZmUBI$_{pro}$:cDNA (ID# 954)

Two independent T1 lines:
80-1 (IRS954-3 – 1 individual)
80-2 (IRS954-10 – 1 individual)

Genomic PCR

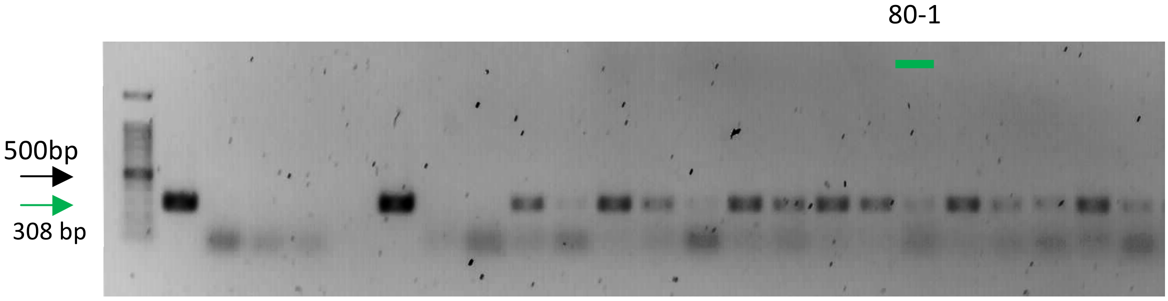

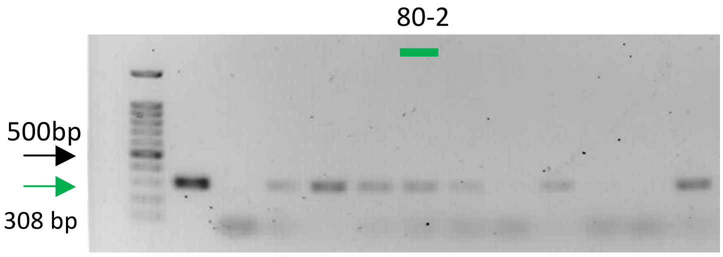

Number veins versus leaf width

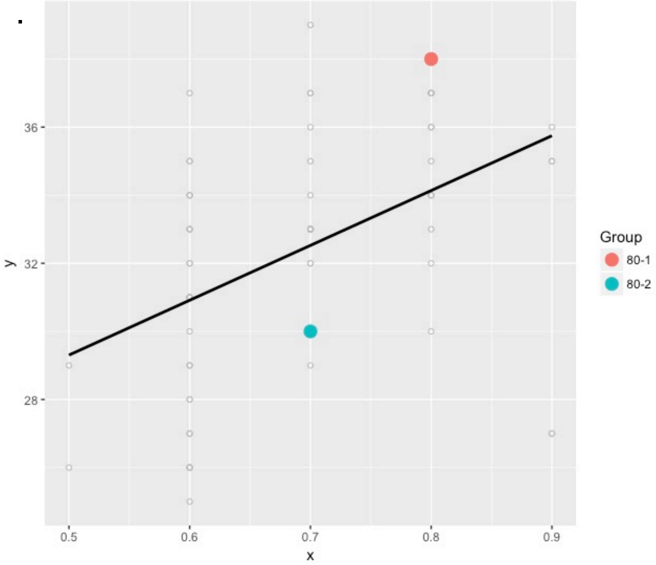

Representative cross section of segregating WT leaf

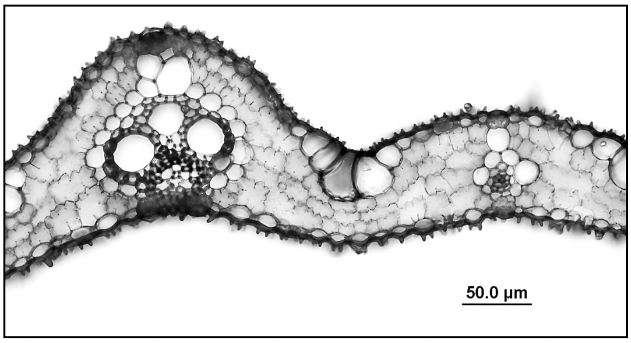

Representative cross section of line 80-2 leaf

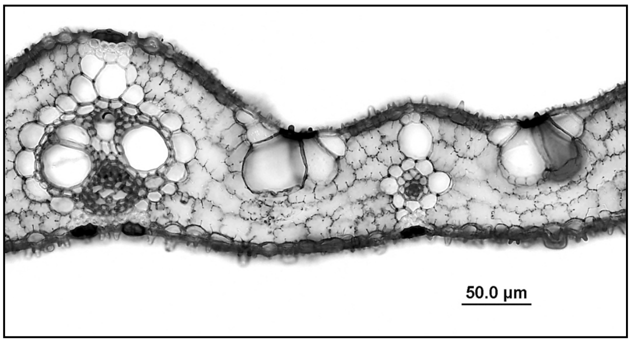

GRMZM2G312419 **R2R3 Myb** **Gene ID: JL81**

Foliar expression

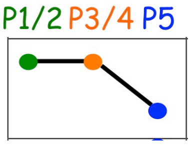

Husk expression

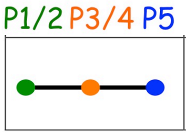

Construct: ZmUBI$_{pro}$:cDNA (ID# 961)

Three independent T1 lines:
81-1 (IRS961-1 – 1 individual)
81-2 (IRS961-6 – 3 individuals)
81-3 (IRS961-9 – 1 individual)

Southern blot

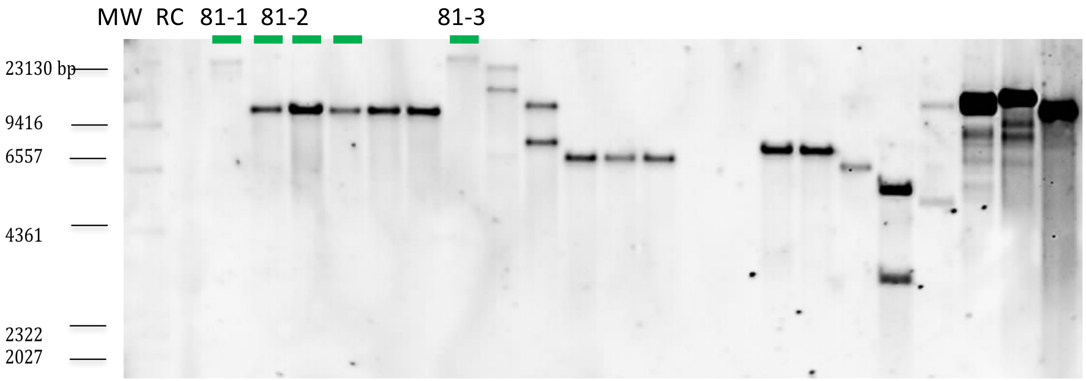

Number veins versus leaf width

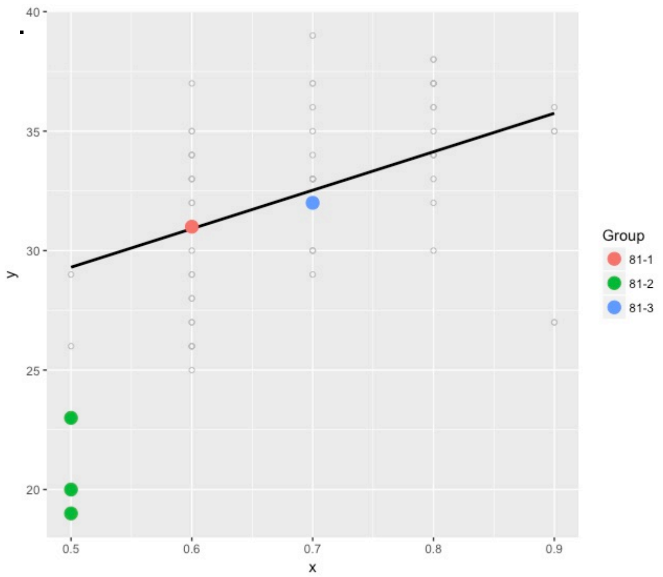

Representative cross section of segregating WT leaf

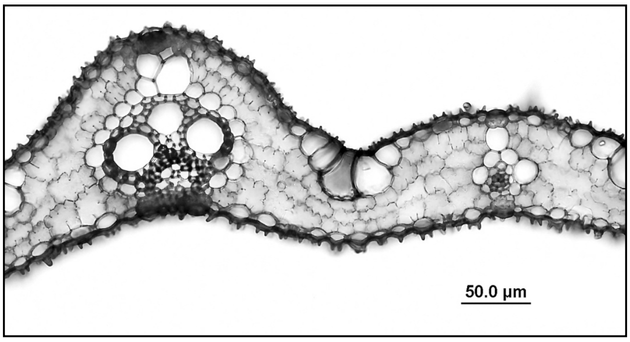

Representative cross section of line 81-3 leaf

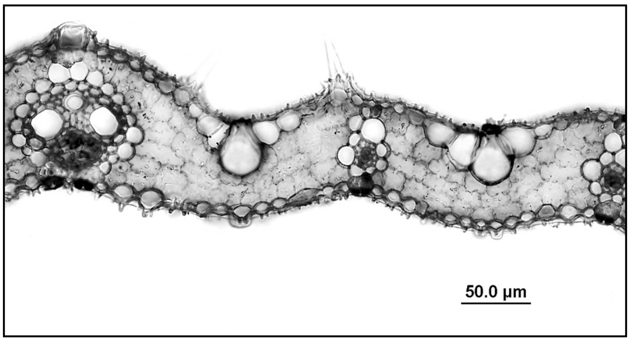

GRMZM2G478876 **Serine threonine kinase** **Gene ID: JL82**

Foliar expression

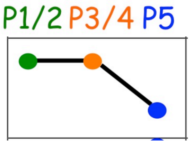

Husk expression

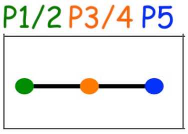

Construct: ZmUBI$_{pro}$:cDNA (ID# 940)

Nine independent T1 lines:
82-1 (IRS940-4–6 individuals) 82-4 (IRS940-10–7 individuals) 82-7 (IRS940-17–3 individuals)
82-2 (IRS940-5– 1 individual) 82-5 (IRS940-11–6 individuals) 82-8 (IRS940-19– 2 individuals)
82-3 (IRS940-7– 1 individual) 82-6 (IRS940-15 –2 individuals) 82-9 (IRS940-35– 6 individuals)

Southern blot

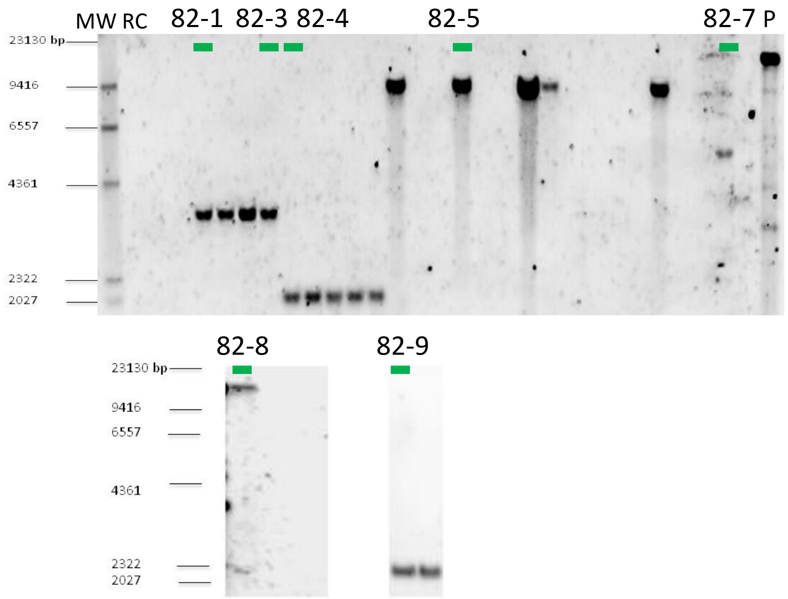

MW: DNA Molecular weight marker II, P- plasmid, RC- non-transgenic rice control

Number veins versus leaf width

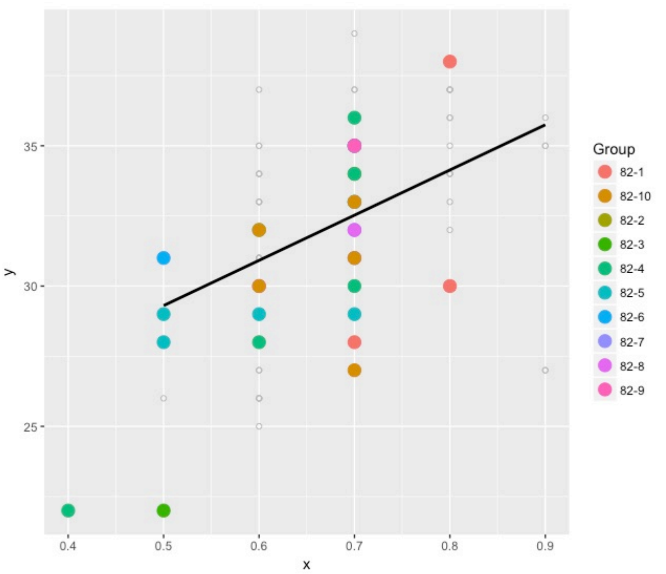

Representative cross section of segregating WT leaf

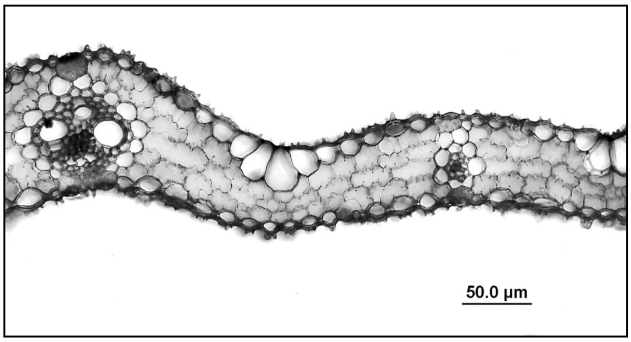

Representative cross section of line 82-8 leaf

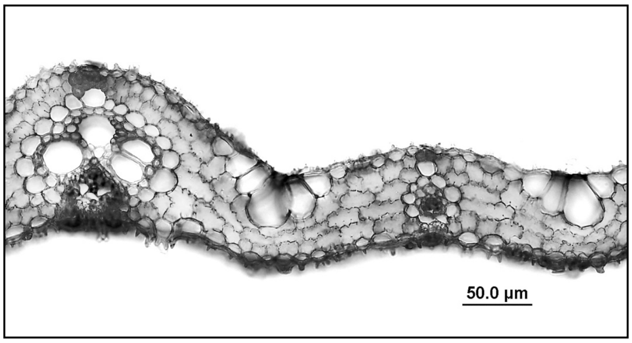

Figure S1. Phylogenetic tree of GRMZM2G074032 (*ZmIDD16*). Maximum-likelihood tree of the GRMZM2G074032 orthogroup (as defined by Orthofinder [2]). Numbers at each node are support values based on the consensus tree of 10 bootstrap replicates. Arabidopsis and rice proteins, plus the maize protein encoded by the gene analysed in this study are highlighted in yellow. Branch lengths reflect the number of amino acid substitutions per site.

Figure S2. Phylogenetic tree of GRMZM2G027068 (*ZmbHLH106*). Maximum-likelihood tree of the GRMZM2G027068 orthogroup (as defined by Orthofinder [2]). Numbers at each node are support values based on the consensus tree of 10 bootstrap replicates. Arabidopsis proteins, plus the maize protein encoded by the gene analysed in this study are highlighted in yellow. Branch lengths reflect the number of amino acid substitutions per site.

Figure S3. Phylogenetic tree of GRMZM2G140694 (*ZmHCA2*). Maximum-likelihood tree of one of the sub-groups of the GRMZM2G140694 orthogroup (as defined by Orthofinder [2]). Numbers at each node are support values based on the consensus tree of 10 bootstrap replicates. Arabidopsis HCA2, plus the maize protein encoded by the gene analysed in this study are highlighted in yellow. Branch lengths reflect the number of amino acid substitutions per site.

Figure S4. Phylogenetic tree of GRMZM2G480386 (YUCCA). Maximum-likelihood tree of the GRMZM2G480386 orthogroup (as defined by Orthofinder [2]). Numbers at each node are support values based on the consensus tree of 10 bootstrap replicates. Rice YUCCA8 plus the maize protein encoded by the gene analysed in this study are highlighted in yellow. Branch lengths reflect the number of amino acid substitutions per site.

Figure S5. Phylogenetic tree of GRMZM2G011463 (*ZmSAUR60*). Maximum-likelihood tree of the GRMZM2G011463 orthogroup (as defined by Orthofinder [2]). Numbers at each node

are support values based on the consensus tree of 10 bootstrap replicates. Rice and Arabidopsis proteins, plus the maize protein encoded by the gene analysed in this study are highlighted in yellow. Branch lengths reflect the number of amino acid substitutions per site.

Figure S6. Phylogenetic tree of GRMZM2G098988 (*ZmSACL3*). Maximum-likelihood tree of the GRMZM2G098988 orthogroup (as defined by Orthofinder [2]). Numbers at each node are support values based on the consensus tree of 10 bootstrap replicates. Arabidopsis SACL3, plus the maize protein encoded by the gene analysed in this study are highlighted in yellow. Branch lengths reflect the number of amino acid substitutions per site.

Figure S7. Phylogenetic tree of GRMZM2G163724 (LRR kinase). Maximum-likelihood tree of the GRMZM2G163724 orthogroup (as defined by Orthofinder [2]). Numbers at each node are support values based on the consensus tree of 10 bootstrap replicates. Arabidopsis RDK1, plus the maize protein encoded by the gene analysed in this study are highlighted in yellow. Branch lengths reflect the number of amino acid substitutions per site.

Figure S8. Phylogenetic tree of GRMZM2G111045 (R2R3 MYB). Maximum-likelihood tree of the GRMZM2G111045 orthogroup (as defined by Orthofinder [2]). Numbers at each node are support values based on the consensus tree of 10 bootstrap replicates. Arabidopsis LMI2, plus the maize protein encoded by the gene analysed in this study are highlighted in yellow. Branch lengths reflect the number of amino acid substitutions per site.

Figure S9. Phylogenetic tree of GRMZM2G114893 (Unknown). Maximum-likelihood tree of the GRMZM2G114893 orthogroup (as defined by Orthofinder [2]). Numbers at each node are support values based on the consensus tree of 10 bootstrap replicates. The maize protein encoded by the gene analysed in this study is highlighted in yellow. Branch lengths reflect the number of amino acid substitutions per site.

Figure S10. Phylogenetic tree of GRMZM2G377217 (*ZmWRKY12*). Maximum-likelihood tree of one of the sub-groups of the GRMZM2G377217 orthogroup (as defined by Orthofinder [2]). Numbers at each node are support values based on the consensus tree of 10 bootstrap replicates. Arabidopsis proteins plus the maize protein encoded by the gene analysed in this study are highlighted in yellow. Branch lengths reflect the number of amino acid substitutions per site.

Figure S11. Details of activation tagged line M0094935. **a)** Schematic of rice chromosome 3 showing insertion site of T-DNA (red arrow) upstream of Os03g50920 in line M0094935. **b)** Gel images of RT-PCR reactions showing transcript levels of 03g50920 relative to actin in 21 day old seedling shoots of wild-type (WT) and the insertion line (M0094935).

SUPPLEMENTARY FIGURE S1

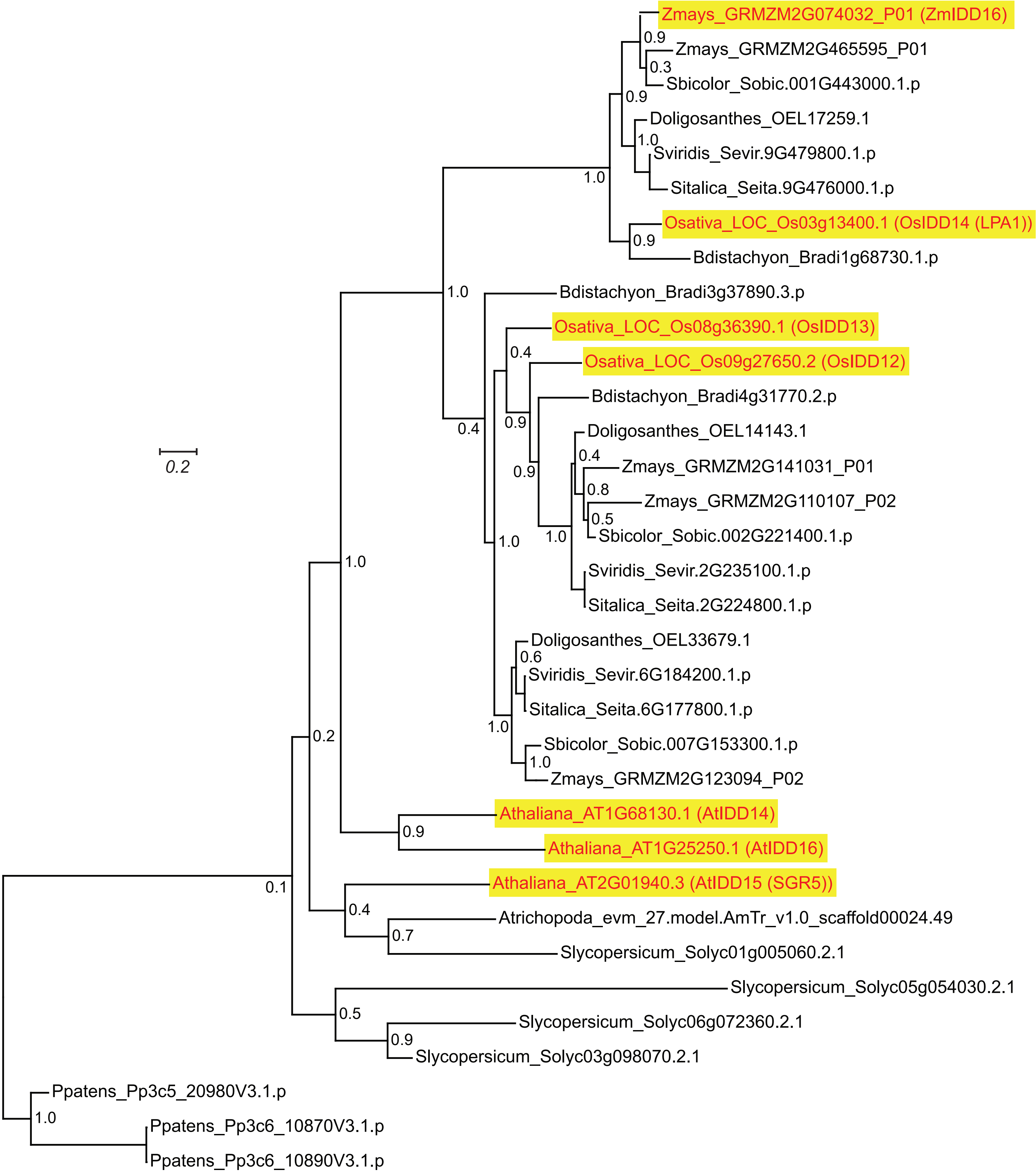

SUPPLEMENTARY FIGURE S2

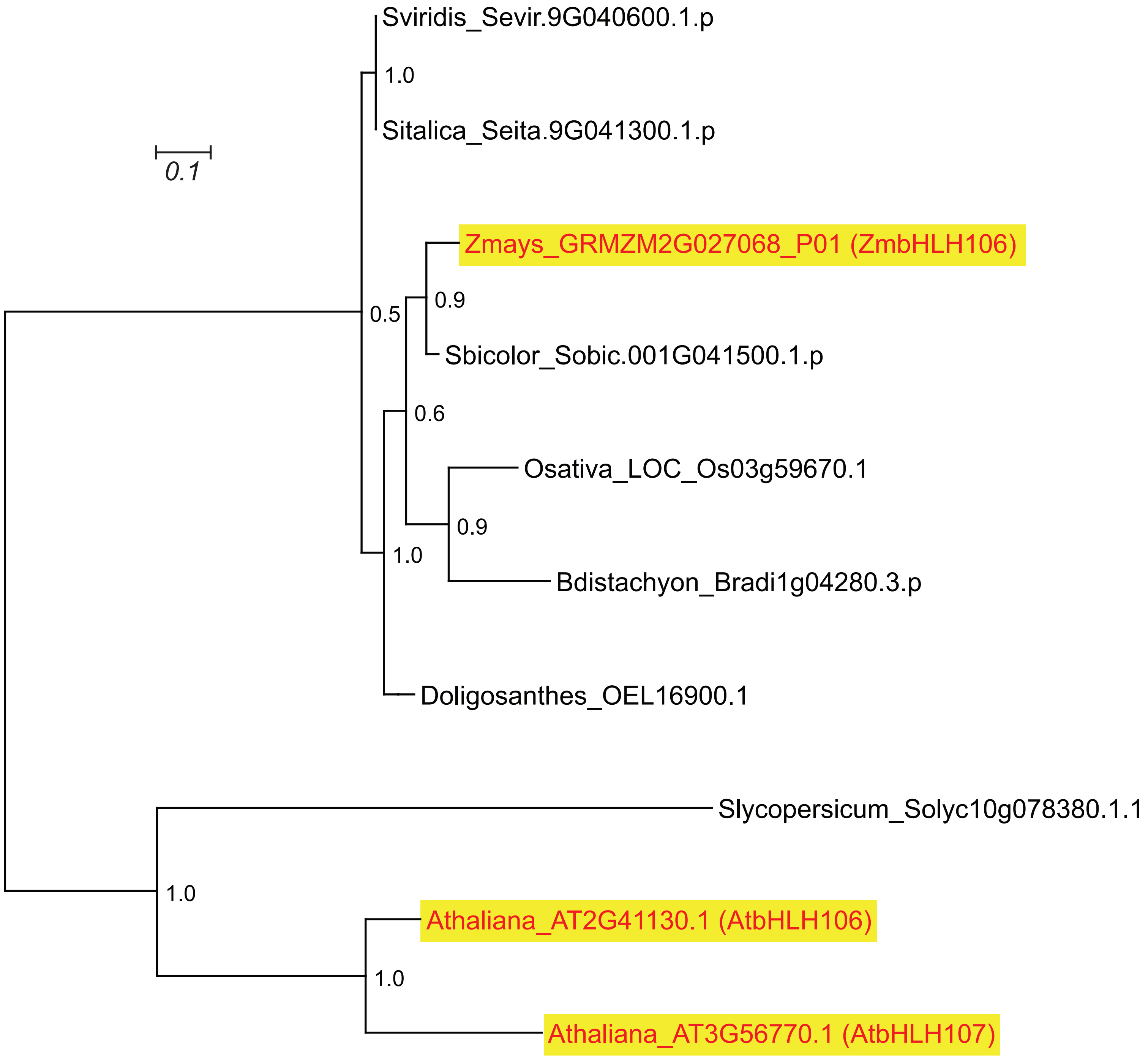

SUPPLEMENTARY FIGURE S3

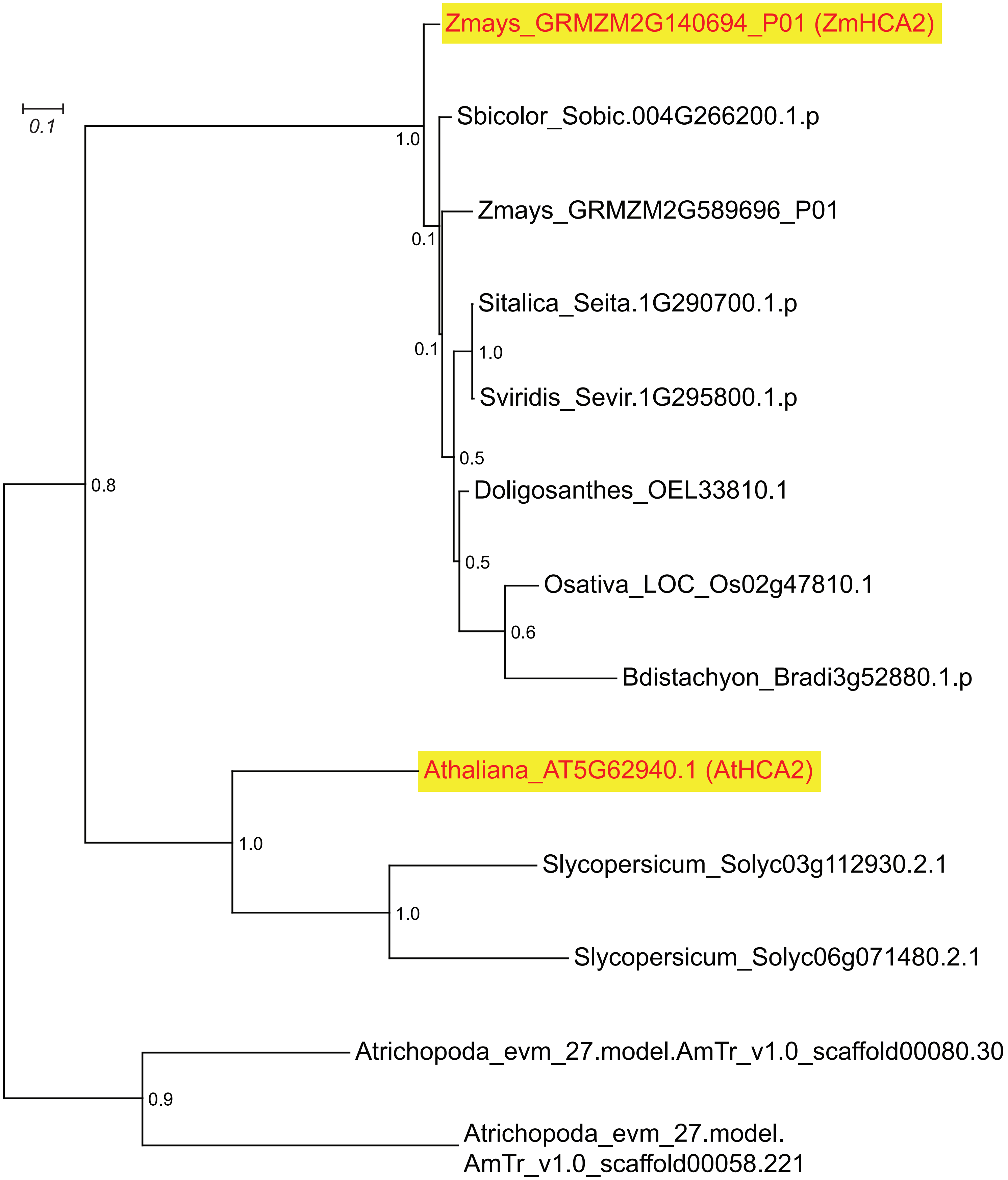

SUPPLEMENTARY FIGURE S4

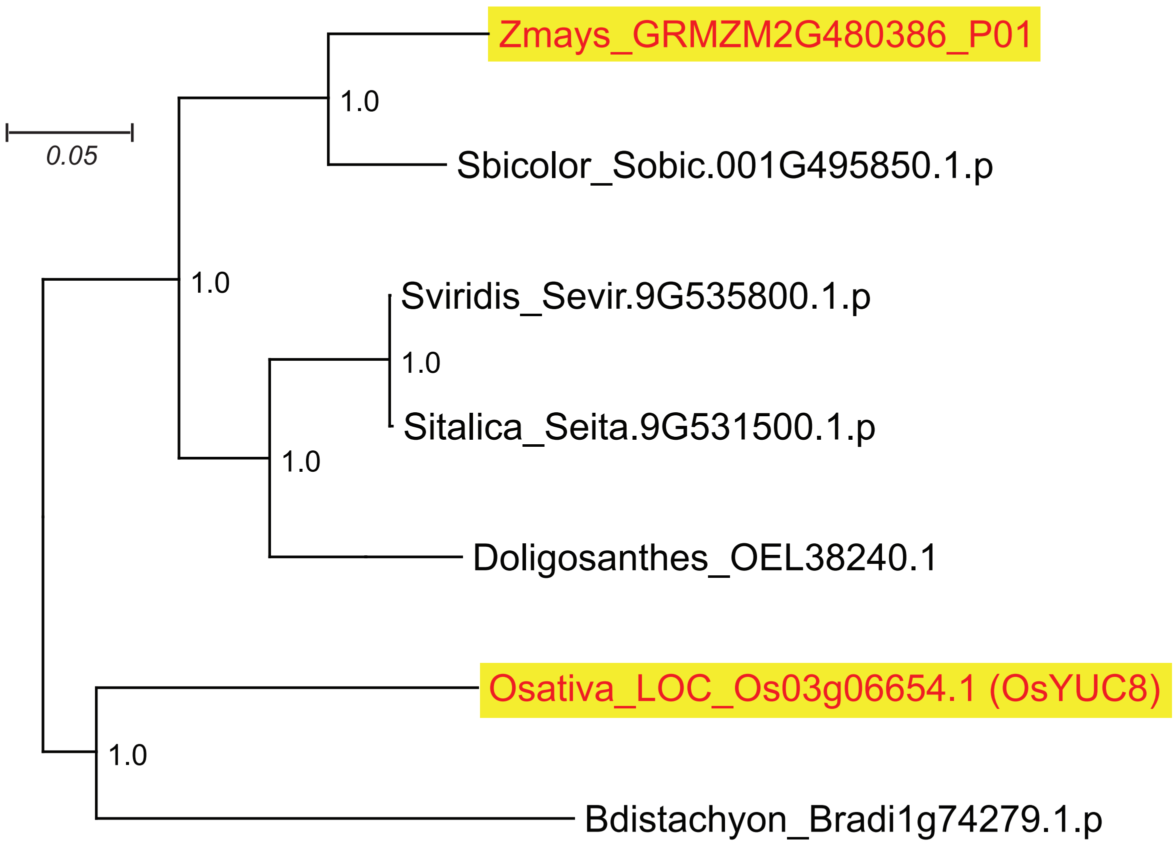

SUPPLEMENTARY FIGURE S5

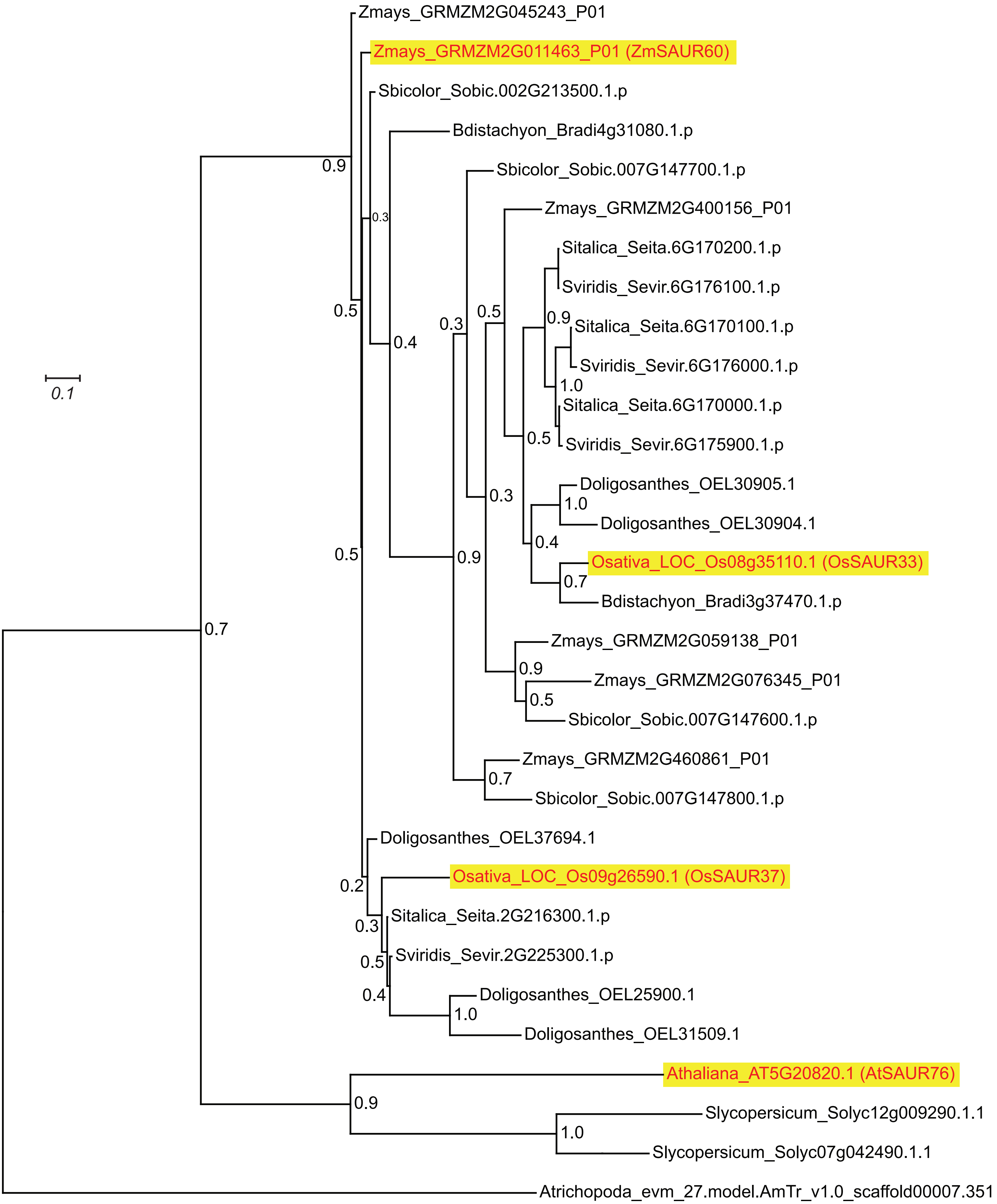

SUPPLEMENTARY FIGURE S6

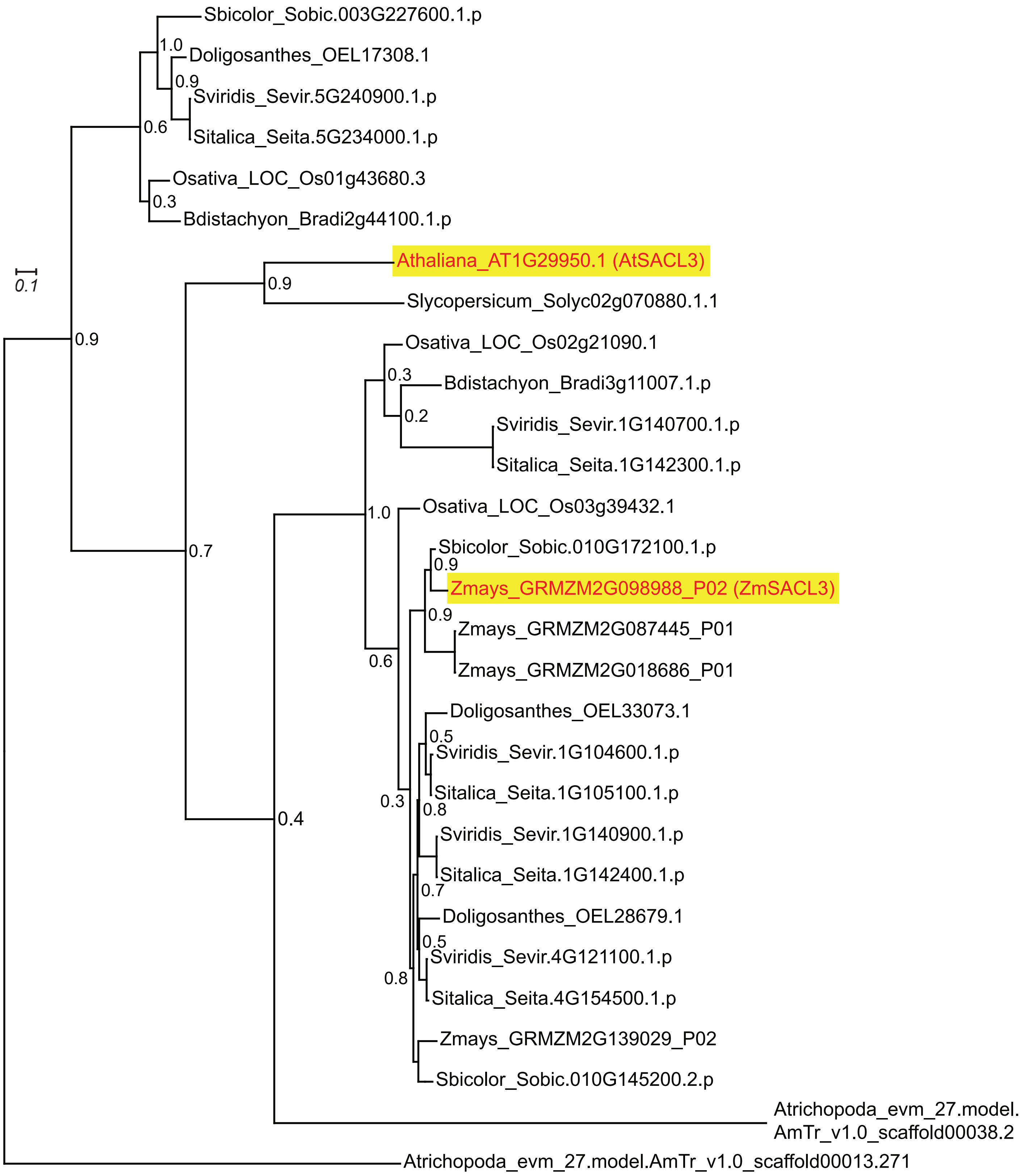

SUPPLEMENTARY FIGURE S7

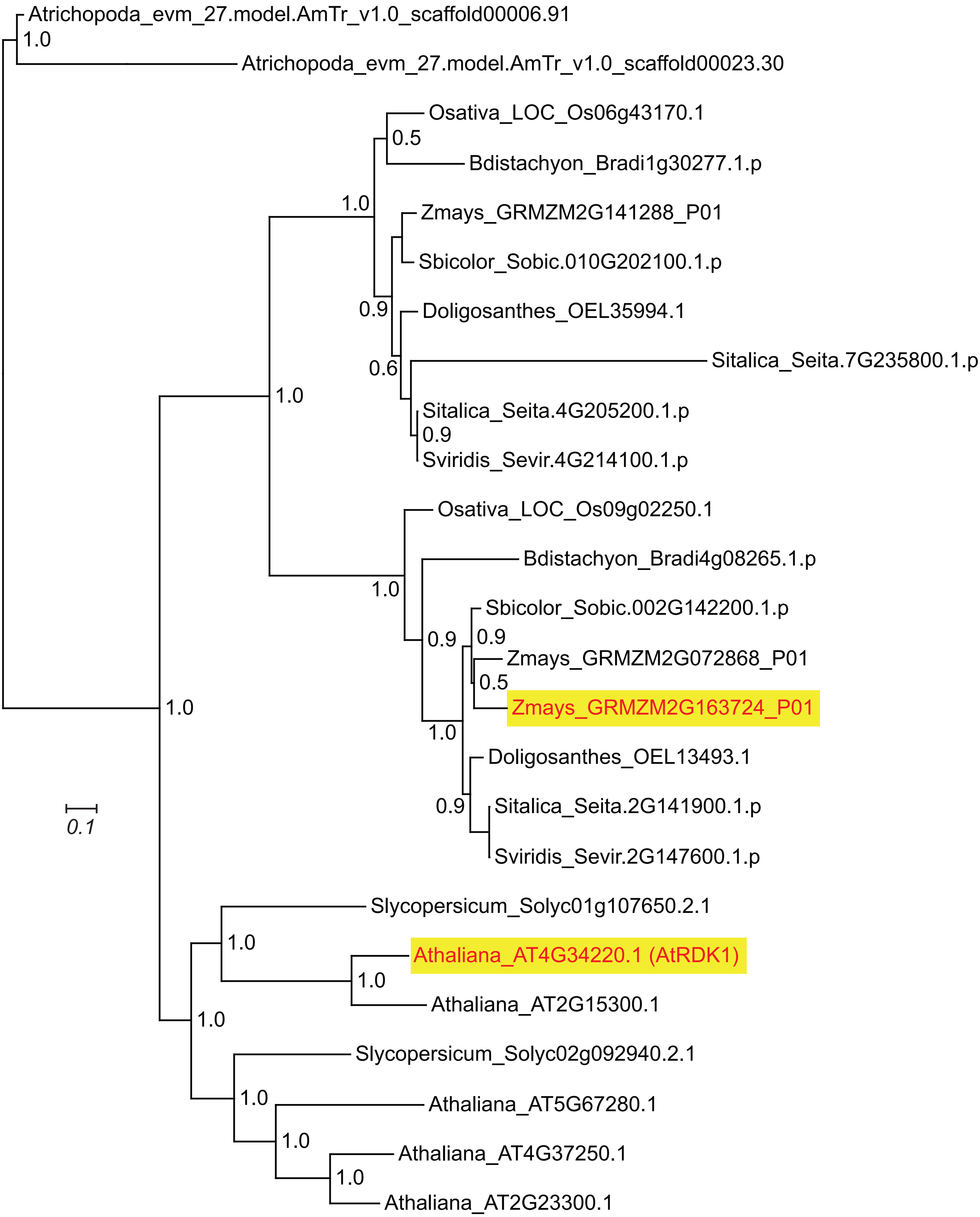

SUPPLEMENTARY FIGURE S8

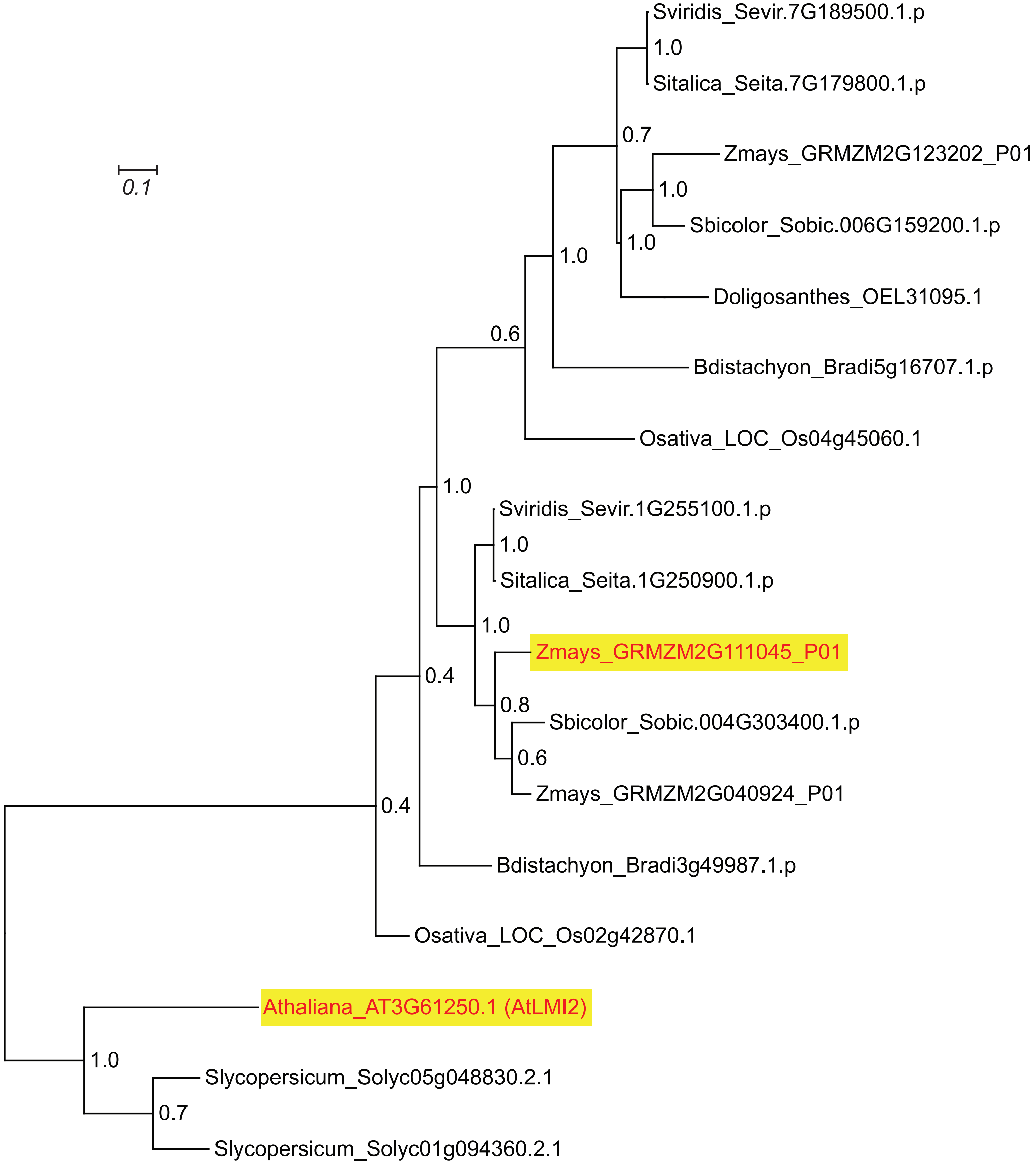

SUPPLEMENTARY FIGURE S9

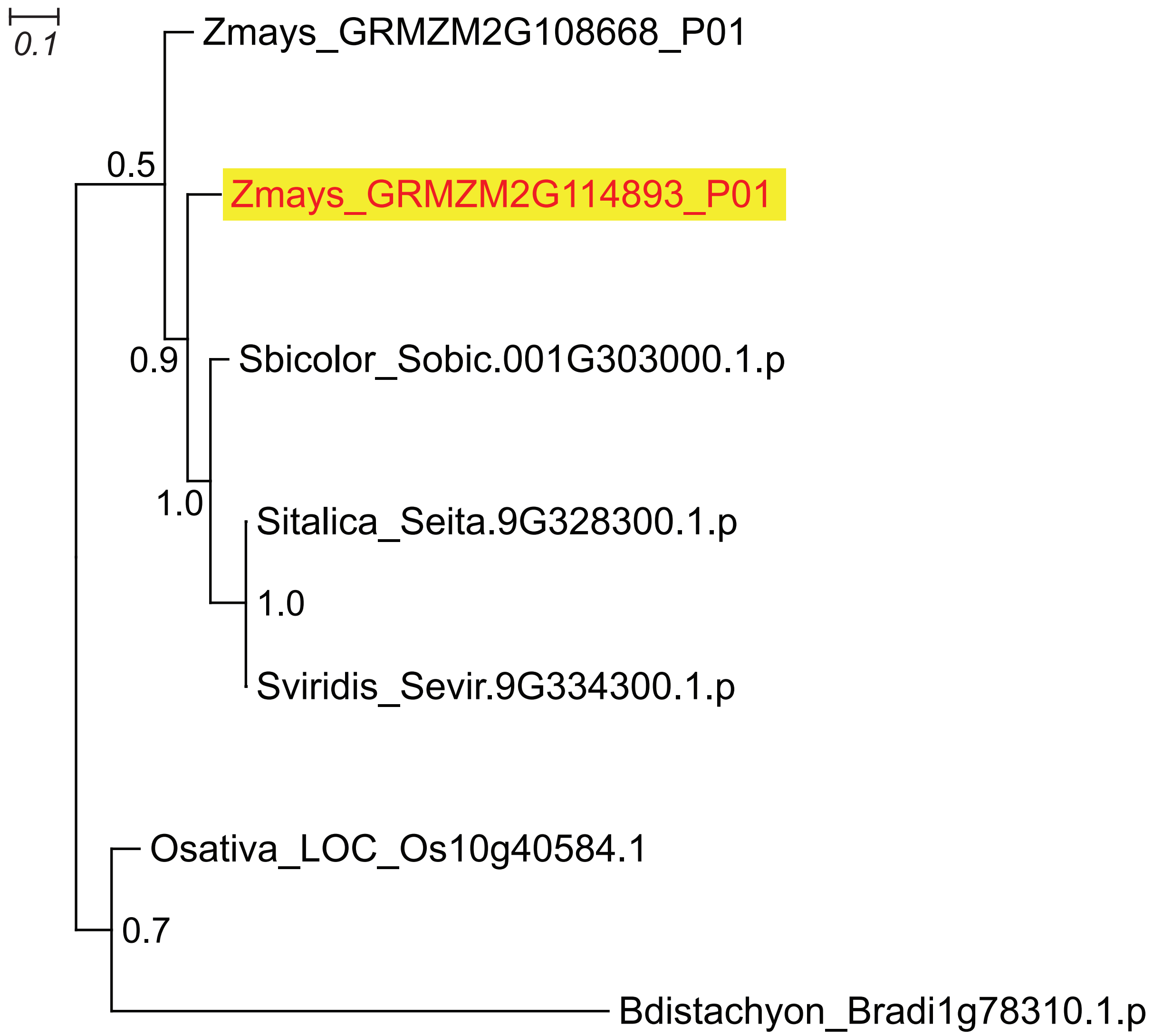

SUPPLEMENTARY FIGURE S10

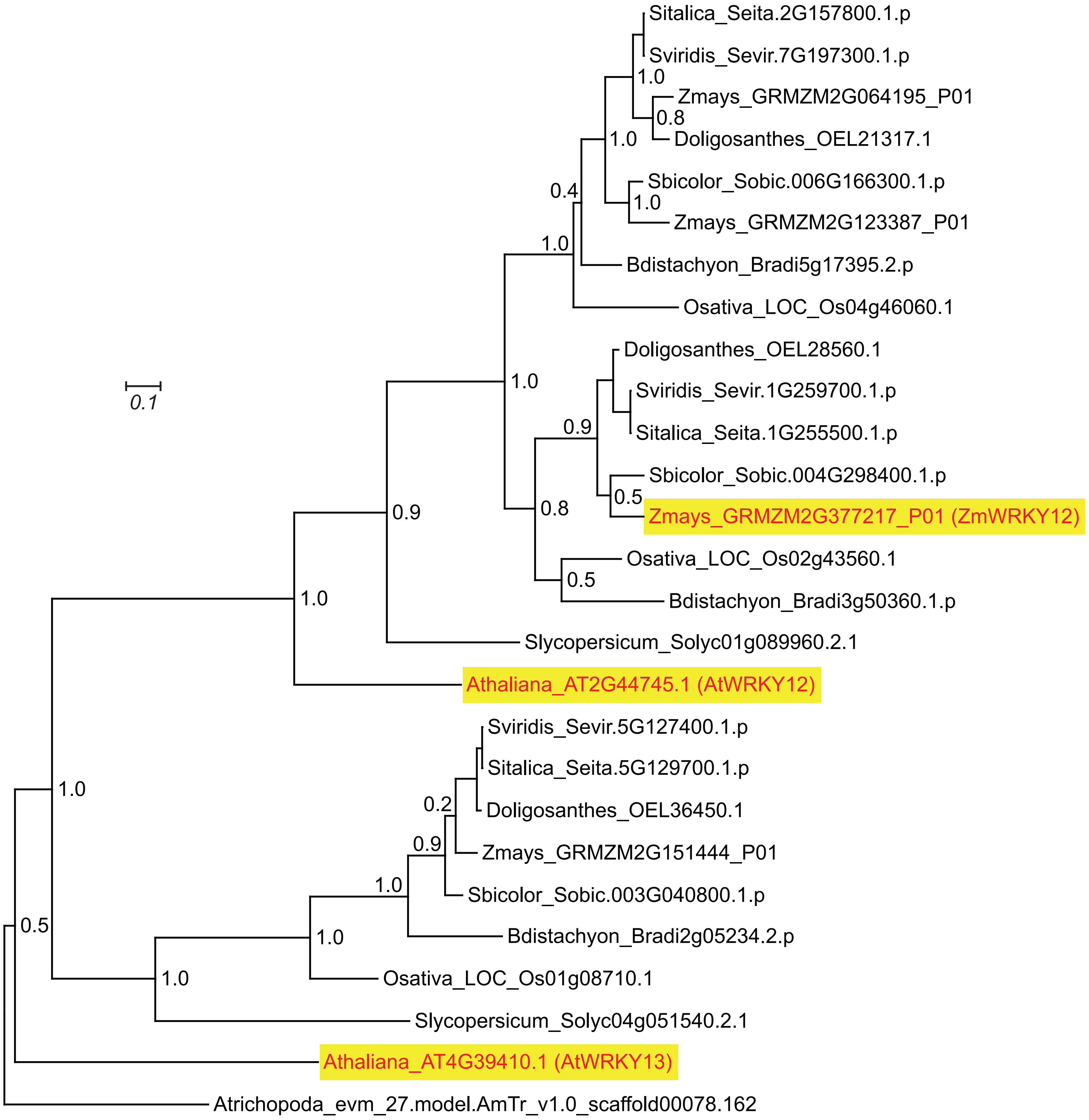

SUPPLEMENTARY FIGURE S11

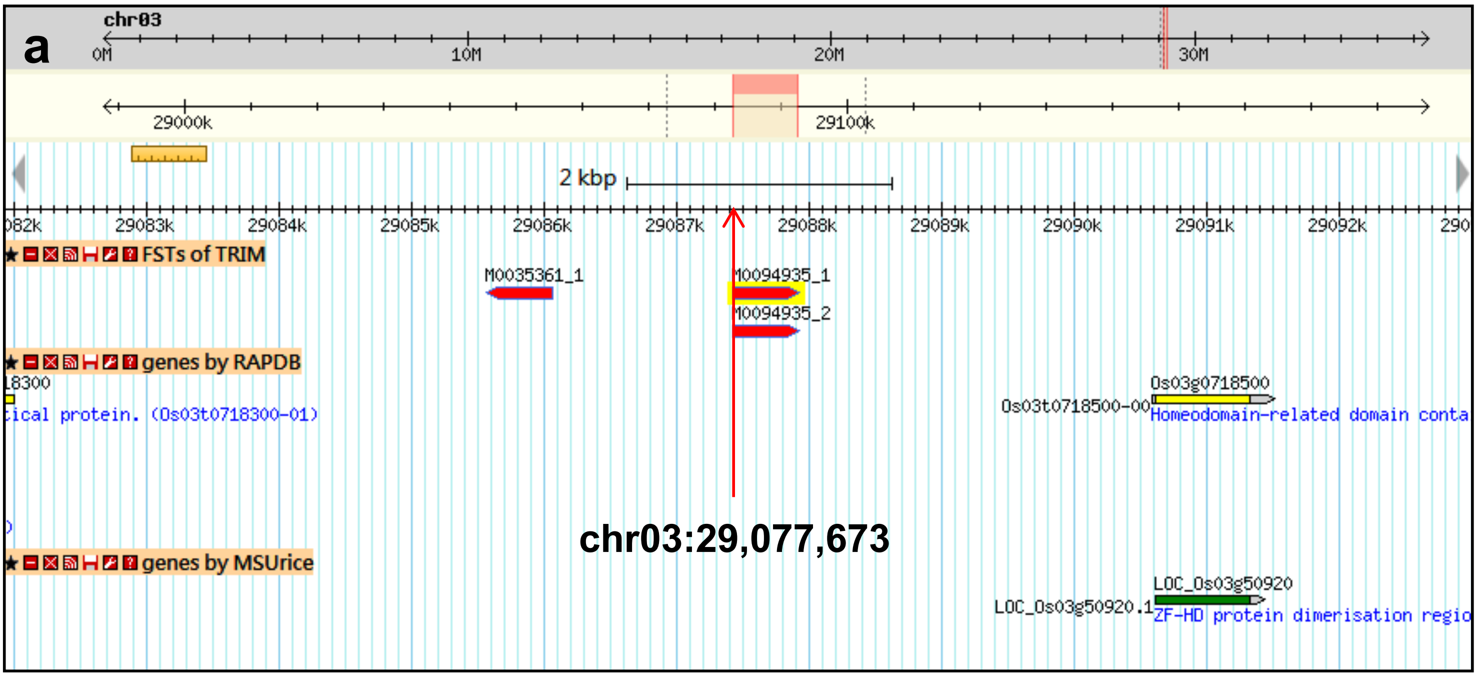

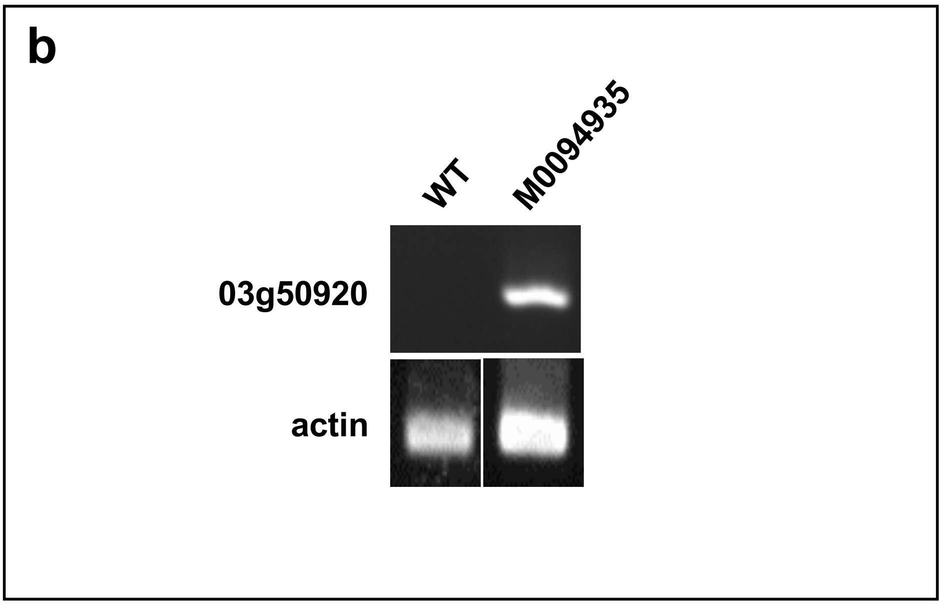

Supplement: Supplementary file 1 — Supplementary Information [file 41598_2017_4361_MOESM1_ESM.pdf]
